# Global, regional, and national burden of Hodgkin lymphoma from 1990 to 2017: estimates from the 2017 Global Burden of Disease study

**DOI:** 10.1186/s13045-019-0799-1

**Published:** 2019-10-22

**Authors:** Linghui Zhou, Yujiao Deng, Na Li, Yi Zheng, Tian Tian, Zhen Zhai, Si Yang, Qian Hao, Ying Wu, Dingli Song, Dai Zhang, Jun Lyu, Zhijun Dai

**Affiliations:** 1 Department of Breast Surgery, The First Affiliated Hospital, College of Medicine, Zhejiang University, Hangzhou, 310003 China; 2 Department of Oncology, The Second Affiliated Hospital of Xi’an Jiaotong University, Xi’an, 710004 China; 3 Clinical Research Center, The First Affiliated Hospital of Xi’an Jiaotong University, Xi’an, 710061 Shaanxi China

**Keywords:** Global Burden of Disease, Hodgkin lymphoma, Incidence, Death, Disability adjusted life-years

## Abstract

**Background:**

Hodgkin lymphoma (HL) is an uncommon B cell lymphoma. We assessed the global, regional, and national burden of HL from 1990 to 2017, by gender, age, and social-demographic index (SDI).

**Methods:**

Data on HL, including incidence, mortality, and disability adjusted life-years (DALY), from 1990 to 2017 were obtained from the 2017 Global Burden of Disease study. Estimated annual percentage changes (EAPCs) were calculated to assess incidence rate, mortality, and DALY trends.

**Results:**

HL incidences increased by 38.66%, from 72,937 in 1990 to 101,133 in 2017, while the age-standardized incidence rate (ASIR) was relatively stable. ASIR decreased in the low SDI regions (EAPC = − 2.58; 95% CI, from − 2.66 to − 2.49) and was stable in the other four SDI regions. Incidence showed a bimodal distribution with peak values in patients aged 20–39 years and patients aged 60 years or higher. The number of death cases and DALYs were stable. The age-standardized death rate decreased by 2.36% (95% CI, from − 2.43% to − 2.30%) per year. The annual age-standardized DALY rate decreased by 2.29% (95% CI, from − 2.36% to − 2.21%). The incidence and mortality in male subjects was higher than that in female subjects. The incidence in male and female subjects aged 15–30 years old was close, whereas the biggest difference existed in patients aged < 10 years old and 45–75 years old between genders.

**Conclusion:**

Globally, incidence of HL was stable, while mortality and DALY rate of HL had been decreasing from 1990 to 2017. Compared with lower and decreasing ASIR in the low SDI region, ASIR in the high SDI region was always high, indicating the need for HL treatment improvement and the establishment of more targeted and specific strategies in high SDI countries to reduce the incidence of HL.

## Background

Hodgkin lymphoma (HL) is a rare B cell lymphoma with 79,990 new cases (accounting for 0.4% of all new tumors) and 26,167 deaths (accounting for 0.3% of all cancer deaths) worldwide in 2018 [1]. HL incidence distribution varies with age, gender, and country [2]. Epidemiological studies had found that HL was rare in children under 5 years of age and was relatively rare in adults, but is the most common cancer among youngsters aged 15–19 years in the USA [3]. The incidence of HL showed a bimodal distribution and increased in subjects aged 20–34 years and subjects aged 55+ years in the USA [4, 5]. Significant advances in the treatment of HL mainly occurred in the 1960s and 1970s. During the mid-twentieth century, the status of HL changed from being an incurable disease to one of the most successfully curable diseases, leading to a sharp decline in mortality in the high-income countries [6]. Epidemiological investigations of HL have either been performed before the twenty-first century or were based mainly on the experience of the Caucasian population in developed countries, and comprehensive and latest studies on the distribution of HL in countries around the world are rare [7].

The Global Burden of Disease (GBD) study assessed the burden of 354 diseases and injuries in 194 countries and regions worldwide and provided an opportunity to comprehensively assess the distribution and development trends of HL in different countries. More detailed data on HL incidence, mortality, DALY, and the corresponding trends in different countries is necessary and could enable policymakers to allocate limited resources and formulate policies more rationally based on this information. Therefore, we conducted this study to reveal the incidence, mortality, disability adjusted life-years (DALY), and the corresponding trends of HL, according to sex, age, socio-demographic index (SDI), region, and country.

## Methods

### Study data

Information on the annual incidence rate, death rate, and DALY of Hodgkin lymphoma, from 1990 to 2017, was collected from the Global Health Data Exchange (GHDx) query tool (http://ghdx.healthdata.org/gbd-results-tool). We also obtained information on gender and age to assess the impact of age and gender on the burden of Hodgkin lymphoma. To further analyze the global burden of Hodgkin lymphoma, we classified location information according to three criteria. We used the social-demographic index (SDI) to divide the countries and regions into five categories (high SDI, high-middle SDI, middle SDI, low-middle SDI, and low SDI). SDI is the geometric average of total fertility, per capita income, and average years of education and ranges from zero to one [8, 9]. As shown in Tables 1, 2, and 3, the world was geographically divided into 21 GBD regions to observe differences. In addition, we drew world maps including 194 countries to observe the incidence rate, death rate and DALY of HL, and the corresponding trends in different countries from 1990 to 2017.

**Table 1:** The incident cases and ASIR in 1990 and 2017 and its temporal trends

	1990		2017		1990–2017
	Incident cases No. *10^2^ (95% UI)	ASIR per 100,000 No. (95% UI)	Incident cases No. *10^2^ (95% UI)	ASIR per 100,000 No. (95% UI)	EAPC No. (95% CI)
Overall	729.37 (558.01–793.7)	1.43 (1.1–1.56)	1011.33 (879.68–1187.46)	1.29 (1.12–1.51)	− 0.51 (from − 0.57 to − 0.45)
Sex					
Female	287.61 (212.52–321.94)	1.1 (0.82–1.23)	403.81 (344.46–483.68)	1.03 (0.87–1.23)	-0.4 (from − 0.46 to − 0.33)
Male	441.76 (321.52–492.58)	1.78 (1.31–2)	607.51 (504.7–748.82)	1.56 (1.29–1.91)	− 0.59 (from − 0.65 to − 0.52)
Socio-demographic index					
High SDI	272.62 (205.49–293.66)	2.57 (1.94–2.77)	349.61 (319.69–464.2)	2.82 (2.56–3.82)	0.19 (0.02–0.36)
High-middle SDI	159.29 (106.83–191.53)	1.43 (0.96–1.71)	266.55 (206.21–292.54)	1.79 (1.39–1.97)	0.84 (0.6–1.08)
Middle SDI	106.75 (74.85–122.02)	0.79 (0.56–0.9)	165.29 (135.05–194.99)	0.76 (0.62–0.89)	− 0.31 (− 0.66–0.04)
Low-middle SDI	113.41 (84.68–131.35)	1.28 (1–1.51)	151.78 (122.97–183.12)	0.95 (0.78–1.15)	− 1.22 (from − 1.27 to − 1.16)
Low SDI	75.32 (54.46–90.43)	1.34 (1–1.64)	74.47 (62.97–101.86)	0.7 (0.6–0.95)	− 2.58 (from − 2.66 to − 2.49)
Region					
Andean Latin America	2.27 (1.94–2.76)	0.74 (0.63–0.87)	3.23 (2.71–4.01)	0.55 (0.46–0.67)	− 0.96 (from − 1.08 to − 0.83)
Australasia	5.14 (3.82–5.97)	2.38 (1.76–2.78)	9.83 (7.85–11.79)	3.26 (2.6–3.92)	1.17 (0.94–1.4)
Caribbean	1.95 (1.65–2.55)	0.6 (0.51–0.79)	5.67 (2.96–7.07)	1.16 (0.61–1.45)	2.61 (1.95–3.27)
Central Asia	6.44 (5.07–7.57)	1.01 (0.8–1.19)	9.81 (8.15–11.44)	1.07 (0.89–1.24)	0.02 (− 0.14–0.18)
Central Europe	30.17 (23.4–33.29)	2.32 (1.8–2.57)	33.02 (29.14–40.17)	2.88 (2.52–3.51)	0.78 (0.73–0.83)
Central Latin America	12.51 (10.11–14.54)	0.98 (0.8–1.13)	22.26 (18.82–27.79)	0.88 (0.75–1.1)	− 0.47 (from − 0.66 to − 0.29)
Central Sub-Saharan Africa	2.78 (2.07–3.84)	0.73 (0.57–0.98)	4.39 (3.51–5.88)	0.54 (0.42–0.75)	− 1.17 (from − 1.26 to − 1.08)
East Asia	101.65 (55.81–126.45)	0.88 (0.48–1.09)	190.18 (135.5–216.38)	1.18 (0.86–1.34)	0.92 (0.32–1.52)
Eastern Europe	75.89 (47.6–96.16)	3.31 (2.03–4.25)	84.95 (74.1–107.26)	3.99 (3.43–5.19)	0.77 (0.53–1.01)
Eastern Sub-Saharan Africa	24.82 (18.06–31.49)	1.64 (1.25–2.09)	32.12 (23.46–44.47)	1.04 (0.77–1.41)	− 1.94 (from − 2.08 to − 1.8)
High-income Asia Pacific	7.59 (6.61–9.95)	0.4 (0.35–0.52)	19.12 (13.33–21.99)	0.86 (0.61–1)	3.71 (3.38–4.05)
High-income North America	104 (76.35–114.3)	3.37 (2.48–3.72)	124.45 (105.69–181.54)	3.23 (2.69–4.84)	− 0.66 (from − 0.98 to − 0.34)
North Africa and Middle East	35.99 (28.58–44.4)	1.32 (1.08–1.69)	84.81 (59.75–97.89)	1.45 (1.03–1.65)	0.58 (0.36–0.8)
Oceania	0.3 (0.24–0.38)	0.61 (0.49–0.78)	0.56 (0.39–0.7)	0.54 (0.38–0.68)	− 0.24 (from − 0.36 to − 0.12)
South Asia	110.08 (79.04–128.65)	1.16 (0.85–1.34)	115.1 (96.77–147.09)	0.68 (0.57–0.87)	− 2.1 (from − 2.18 to − 2.02)
Southeast Asia	28.26 (22.17–33.2)	0.75 (0.59–0.9)	34.96 (29.01–43.18)	0.53 (0.44–0.65)	− 1.39 (from − 1.46 to − 1.32)
Southern Latin America	4.66 (4.06–5.94)	0.96 (0.84–1.22)	6.81 (5.67–9.51)	0.95 (0.79–1.33)	− 0.16 (− 0.37–0.05)
Southern Sub-Saharan Africa	1.75 (1.34–2.03)	0.43 (0.34–0.52)	2.81 (1.96–3.26)	0.4 (0.28–0.46)	− 0.18 (− 0.83–0.48)
Tropical Latin America	9.6 (8.18–11.77)	0.75 (0.63–0.9)	15.1 (12.48–18.39)	0.65 (0.54–0.79)	− 0.38 (from − 0.47 to − 0.3)
Western Europe	134.79 (103.14–149.57)	3.14 (2.39–3.51)	167.97 (149–227.28)	3.58 (3.18–4.96)	0.53 (0.38–0.68)
Western Sub-Saharan Africa	28.72 (18.39–39.7)	1.79 (1.28–2.36)	44.19 (30.78–60.15)	1.24 (0.91–1.69)	− 1.63 (from − 1.76 to − 1.51)

*ASIR* age-standardized incidence rate

**Table 2:** The death cases and ASDR in 1990 and 2017 and its temporal trends

	1990		2017		1990–2017
	Death cases No. *10^2^ (95% UI)	ASDR per 100,000 No. (95% UI)	Death cases No. *10^2^ (95% UI)	ASDR per 100,000 No. (95% UI)	EAPC No. (95% CI)
Overall	359.46 (273.29–394.12)	0.75 (0.58–0.83)	325.6 (276.44–380.86)	0.41 (0.35–0.48)	− 2.36 (from − 2.43 to − 2.3)
Sex					
Female	126.22 (90.97–143.6)	0.51 (0.37–0.58)	118.4 (95.76–141.1)	0.29 (0.24–0.35)	− 2.24 (from − 2.34 to − 2.15)
Male	233.24 (173.14–269.48)	1.02 (0.76–1.18)	207.2 (168.22–257.67)	0.54 (0.44–0.67)	− 2.45 (from − 2.5 to − 2.39)
Socio-demographic index					
High SDI	66.23 (51.03–72.61)	0.56 (0.43–0.61)	48.06 (41.38–59.99)	0.27 (0.24–0.34)	− 2.75 (from − 2.86 to − 2.64)
High-middle SDI	69.27 (48.76–80.4)	0.67 (0.47–0.78)	50.38 (39.59–54.62)	0.3 (0.24–0.33)	− 3.28 (from − 3.44 to − 3.12)
Middle SDI	75.51 (54.1–85.85)	0.62 (0.45–0.7)	64.53 (52.33–73.79)	0.29 (0.24–0.34)	− 2.92 (from − 3.05 to − 2.79)
Low-middle SDI	87.84 (66.71–101.45)	1.07 (0.86–1.3)	103.76 (84.69–126.81)	0.7 (0.58–0.87)	− 1.72 (from − 1.8 to − 1.64)
Low SDI	59.46 (43.62–71.02)	1.15 (0.87–1.4)	58.19 (49.24–79.09)	0.59 (0.5–0.8)	− 2.62 (from − 2.69 to − 2.54)
Region					
Andean Latin America	1.82 (1.54–2.16)	0.65 (0.55–0.76)	2.01 (1.63–2.31)	0.36 (0.29–0.41)	− 2.07 (from − 2.18 to − 1.95)
Australasia	1.04 (0.79–1.14)	0.45 (0.34–0.49)	1.01 (0.79–1.17)	0.24 (0.19–0.29)	− 2.07 (from − 2.33 to − 1.81)
Caribbean	1.29 (1.09–1.72)	0.43 (0.36–0.57)	2.57 (1.39–3.18)	0.52 (0.28–0.64)	0.81 (0.23–1.39)
Central Asia	3.75 (2.94–4.4)	0.65 (0.51–0.77)	3.7 (3.16–4.37)	0.44 (0.37–0.52)	− 1.61 (from − 1.8 to − 1.43)
Central Europe	14.37 (10.94–15.45)	1.01 (0.77–1.08)	7.83 (6.81–9.36)	0.45 (0.39–0.53)	− 3.22 (from − 3.31 to − 3.12)
Central Latin America	9.09 (7.37–10.38)	0.81 (0.65–0.93)	10.74 (8.99–13.15)	0.45 (0.37–0.55)	− 2.26 (from − 2.43 to − 2.1)
Central Sub-Saharan Africa	2.27 (1.71–3.12)	0.66 (0.5–0.9)	3.61 (2.88–4.89)	0.49 (0.38–0.69)	− 1.13 (from − 1.23 to − 1.04)
East Asia	59.88 (34.3–73.07)	0.57 (0.32–0.69)	29 (20.03–32.98)	0.15 (0.11–0.17)	− 5.55 (from − 6 to − 5.09)
Eastern Europe	20.87 (14.19–24.65)	0.82 (0.55–0.96)	14.78 (12.48–18.53)	0.55 (0.46–0.71)	− 2 (from − 2.4 to − 1.6)
Eastern Sub-Saharan Africa	19.22 (14.18–24.31)	1.41 (1.08–1.79)	24.59 (18–33.92)	0.89 (0.66–1.2)	− 1.94 (from − 2.09 to − 1.8)
High-income Asia Pacific	2.22 (1.93–3.08)	0.11 (0.1–0.15)	2.98 (2.07–3.33)	0.08 (0.06–0.09)	− 0.54 (from − 0.71 to − 0.38)
High-income North America	18.32 (13.92–19.59)	0.55 (0.42–0.59)	15.12 (13.71–20.45)	0.3 (0.27–0.41)	− 2.35 (from − 2.48 to − 2.22)
North Africa and Middle East	26.51 (21.51–34)	1.08 (0.9–1.44)	31.96 (23.69–36.41)	0.62 (0.47–0.72)	− 1.91 (from − 1.96 to − 1.85)
Oceania	0.23 (0.18–0.29)	0.51 (0.41–0.66)	0.42 (0.3–0.53)	0.44 (0.31–0.56)	− 0.3 (from − 0.44 to − 0.17)
South Asia	86.49 (62.74–100.24)	0.99 (0.73–1.15)	85.36 (72.6–109.87)	0.53 (0.45–0.68)	− 2.38 (from − 2.46 to − 2.3)
Southeast Asia	22.18 (17.39–26.71)	0.64 (0.5–0.78)	21.86 (17.87–26.39)	0.35 (0.28–0.42)	− 2.29 (from − 2.38 to − 2.2)
Southern Latin America	3.6 (3.13–4.53)	0.75 (0.65–0.94)	3.22 (2.77–4.34)	0.42 (0.36–0.57)	− 2.23 (from − 2.38 to − 2.08)
Southern Sub-Saharan Africa	1.38 (1.08–1.65)	0.37 (0.29–0.45)	2.13 (1.52–2.44)	0.32 (0.23–0.37)	− 0.3 (− 0.97–0.37)
Tropical Latin America	7.05 (5.88–8.62)	0.6 (0.49–0.73)	7.47 (6.09–9.19)	0.32 (0.26–0.39)	− 2.27 (from − 2.34 to − 2.21)
Western Europe	35.86 (27.52–39.98)	0.7 (0.54–0.78)	24.41 (20.13–30.57)	0.34 (0.29–0.44)	− 2.62 (from − 2.77 to − 2.47)
Western Sub-Saharan Africa	21.98 (14.81–29.45)	1.53 (1.13–2)	30.83 (21.93–41.61)	1 (0.75–1.35)	− 1.85 (from − 1.97 to − 1.72)

*ASDR* age-standardized death rate

**Table 3:** The DALY and age-standardized DALY rate in 1990 and 2017 and its temporal trends

	1990		2017		1990–2017
	DALY No. *10^3^ (95% UI)	Age-standardized DALY Rate per 100,000 No. (95% UI)	DALY No. *10^3^ (95% UI)	Age-standardized DALY Rate per 100,000 No. (95% UI)	EAPC No. (95% CI)
Overall	1657.47 (1228.33–1843.22)	31.53 (23.56–34.73)	1378.17 (1155.06–1624.39)	17.77 (14.87–20.93)	− 2.29 (− 2.36 to − 2.21)
Sex					
Female	574.77 (393.26–677.46)	21.67 (14.99–25.31)	507.65 (400.13–605.67)	13.11 (10.28–15.63)	− 2.07 (from − 2.17 to − 1.98)
Male	1082.7 (797.07–1238.41)	41.46 (30.69–47.65)	870.52 (704.9–1092.74)	22.47 (18.14–28.18)	− 2.4 (from − 2.46 to − 2.34)
Socio-demographic index					
High SDI	234.61 (179.77–252.24)	21.48 (16.38–23.05)	149.39 (134.99–194.83)	10.57 (9.5–14.16)	− 2.64 (from − 2.75 to − 2.53)
High-middle SDI	292.4 (202.91–339.63)	26.5 (18.43–30.8)	190.67 (151.93–207.8)	12.19 (9.71–13.31)	− 3.24 (from − 3.42 to − 3.06)
Middle SDI	351.22 (245.53–401.7)	24.16 (17.1–27.57)	240.73 (198.08–276.18)	10.92 (9–12.57)	− 3.13 (from − 3.29 to − 2.97)
Low-middle SDI	455.57 (329.84–533.96)	45.68 (34.46–53.23)	508.84 (405.49–610.97)	30.23 (24.34–36.3)	− 1.65 (from − 1.74 to − 1.56)
Low SDI	318.61 (222.89–394.19)	49.23 (35.86–58.84)	286.14 (240.77–391.31)	24.16 (20.48–32.89)	− 2.82 (from − 2.91 to − 2.73)
Region					
Andean Latin America	8.58 (7.35–10.54)	24.62 (21–29.55)	7.1 (6.03–8.71)	12 (10.23–14.53)	− 2.52 (from − 2.63 to − 2.41)
Australasia	3.67 (2.78–4.05)	16.53 (12.57–18.27)	3.33 (2.65–3.95)	9.96 (8–11.98)	− 1.73 (from − 1.93 to − 1.53)
Caribbean	5.42 (4.61–7.13)	16.37 (13.89–21.53)	9.24 (5.31–11.48)	18.97 (10.98–23.56)	0.61 (0.07–1.15)
Central Asia	16.85 (13.05–19.68)	26.31 (20.44–30.73)	15.79 (13.24–18.3)	17.32 (14.51–20.01)	− 1.66 (from − 1.86 to − 1.45)
Central Europe	53.33 (40.14–57.48)	39.5 (29.85–42.49)	25.32 (21.97–30.07)	17.7 (15.35–20.87)	− 3.18 (from − 3.27 to − 3.1)
Central Latin America	40.8 (33.06–46.94)	28.79 (23.2–32.83)	38.03 (32.35–47.52)	15.1 (12.87–18.82)	− 2.42 (from − 2.61 to − 2.22)
Central Sub-Saharan Africa	11.13 (7.96–15.67)	24.99 (18.65–34.16)	16.85 (13.34–22.61)	17.8 (14.22–24.16)	− 1.32 (from − 1.43 to − 1.21)
East Asia	263.58 (146.58–322.9)	22.06 (12.37–27.04)	96.35 (68.89–111.83)	5.51 (4.01–6.39)	− 5.99 (from − 6.55 to − 5.42)
Eastern Europe	89.73 (58.59–106.43)	37.42 (24.32–44.37)	60.16 (51.2–78.41)	25.61 (21.91–34.32)	− 1.99 (from − 2.38 to − 1.59)
Eastern Sub-Saharan Africa	107.84 (76.09–137.03)	61.51 (45.35–77.92)	133.49 (96.81–187.35)	37.39 (27.47–51.34)	− 2.11 (from − 2.26 to − 1.96)
High-income Asia Pacific	7.41 (6.41–10.27)	3.82 (3.32–5.25)	8.16 (5.68–9.41)	3.17 (2.25–3.7)	− 0.16 (− 0.34–0.01)
High-income North America	70.8 (51.82–77.3)	22.69 (16.57–24.78)	51.21 (43.83–73.82)	12.23 (10.24–18.04)	− 2.44 (from − 2.57 to − 2.31)
North Africa and Middle East	130.28 (101.49–159.85)	42.68 (34.23–54.21)	144.86 (106.08–163.73)	24.9 (18.36–28.03)	− 1.86 (from − 1.93 to − 1.8)
Oceania	1.14 (0.88–1.42)	20.81 (16.29–26.12)	2 (1.39–2.49)	17.62 (12.42–22.07)	− 0.33 (from − 0.48 to − 0.19)
South Asia	454.42 (324.69–539.5)	42.57 (30.73–49.53)	393.94 (336.66–504.19)	22.16 (18.97–28.47)	− 2.49 (from − 2.6 to − 2.38)
Southeast Asia	101.9 (77.67–119.24)	24.49 (19.25–29.16)	83.02 (68.29–101.87)	12.39 (10.15–15.19)	− 2.58 (from − 2.65 to − 2.51)
Southern Latin America	12.61 (11.15–16.11)	25.86 (22.83–33.04)	10.49 (8.9–14.45)	14.5 (12.31–20.11)	− 2.17 (from − 2.32 to − 2.01)
Southern Sub-Saharan Africa	6.28 (4.77–7.26)	14.22 (10.89–16.96)	9.06 (6.12–10.6)	12.28 (8.5–14.24)	− 0.32 (− 1.08–0.45)
Tropical Latin America	30.89 (26.46–38.74)	22.44 (18.85–27.68)	28.48 (23.64–35.39)	12.19 (10.18–15.14)	− 2.16 (from − 2.21 to − 2.1)
Western Europe	120.41 (94–134.6)	26.73 (20.82–29.78)	71.62 (62.27–95.57)	13.06 (11.47–17.79)	− 2.55 (from − 2.69 to − 2.4)
Western Sub-Saharan Africa	120.41 (73.01–169.81)	64.68 (43.28–87.08)	169.69 (116.04–228.38)	41.46 (29.53–56.49)	− 1.99 (from − 2.14 to − 1.84)

*DALY* disability adjusted life-years

### Statistical analysis

The annual age-standardized incidence rate (ASIR), age-standardized death rate (ASDR), age-standardized DALY rate, and the corresponding estimated annual percentage changes (EAPCs) were calculated to assess trends of Hodgkin lymphoma incidence and mortality. DALY was estimated by adding the years lived with disability (YLDs) and the years of life lost (YLLs) [10]. Age-standardization of incidence/death rate was determined to compare the age structure of different groups or the age structure in the same population with changes over time. The purpose is to exclude the influence of age composition on population incidence or mortality because the incidence or mortality of cancer varies greatly in different age stages. ASR (age-standardized incidence/death/DALY rate) (per 100,000 population) is equal to the sum of the product of the specific age ratio (*a*_*i*_) in age group *i* and the number (or weight) (*w*_*i*_) of the selected reference standard population group *i* divided by the sum of number (or weight) of the standard population, i.e., $\mathrm{ASR}=\frac{\sum_{i=1}^A{a}_i{w}_i}{\sum_{i=1}^A{w}_i}\times \mathrm{100,000}$ [11]. EAPCs were calculated by using the following regression model to evaluate the trends of ASRs: *y* = *α* + *βx* + ε, where *y* represents ln (ASR) and *x* refers to the calendar year. EAPC = 100 × (exp(*β*) − 1) and its 95% confidence interval (CI) can be obtained from the regression model [12]. If the EAPC value and its lower limit of 95% CI are both positive, the ASR is considered to be in an increasing trend. Conversely, if the EAPC value and its upper limit of 95% CI are both negative, the ASR is in a downward trend. Otherwise, ASR is considered to be stable. If the EAPC is statistically significant but the uncertainty intervals of the GBD estimates overlap, ASR is still considered to be stable. In addition, we drew scatter plots to observe the links between EAPC and ASR and SDI, respectively. The HL ASR in 1990 is the baseline state of the disease pool. The human development index 2017 can be used as an alternative indicator of health care level in each country. *ρ* represents Pearson’s correlation coefficient. All calculations were performed using R software (version 3.5.1).

## Results

### The change in incidence of HL

**Fig. 1:**
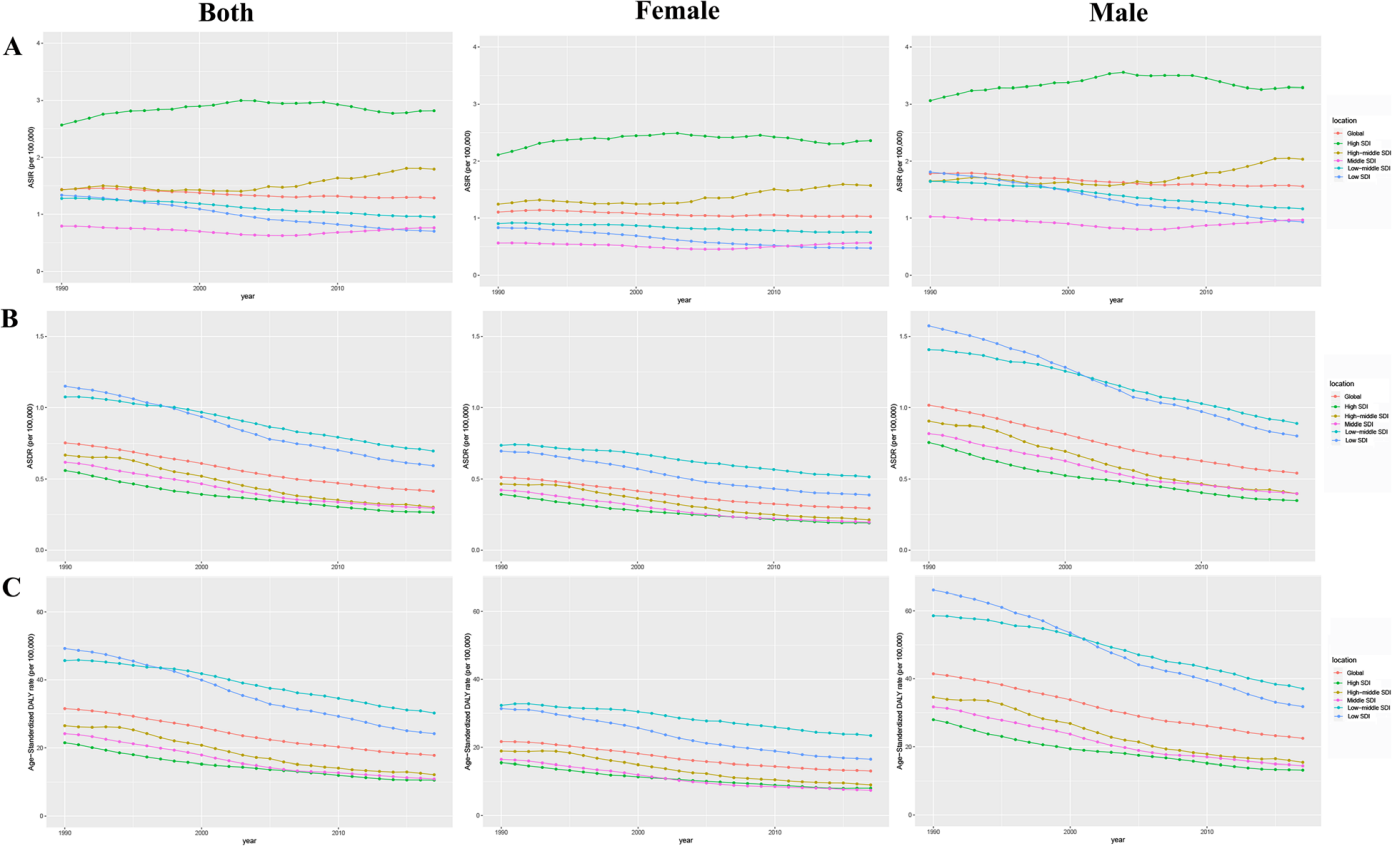
The change trends of age-standardized incidence, death, and DALY rate among different SDI quintiles. **a** Age-standardized incidence. **b** Age-standardized death rate. **c** Age-standardized DALY rate

**Fig. 2:**
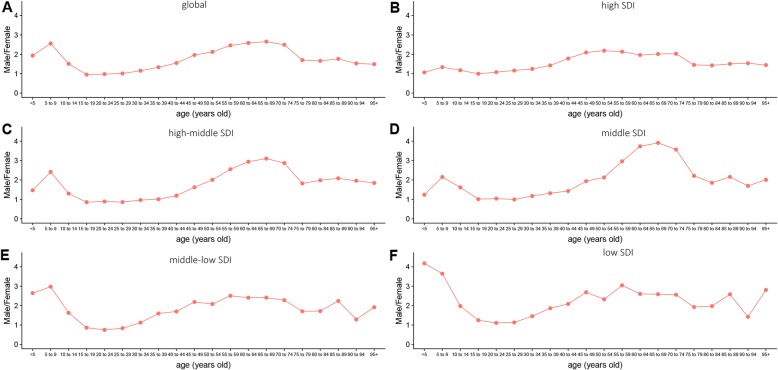
The ratio of male to female incidence among different age groups in 2017. **a** Global. **b** High SDI. **c** High-middle SDI. **d** Middle SDI. **e** Middle-low SDI. **f** Low SDI. SDI, socio-demographic index

At the global level, the annual incidence increased gradually and there were 101,133 (95% UI, 87,968–118,746) incidences in 2017 and 72,937 (95% UI, 55,801–79,370) incidences in 1990 (Table 1, Additional file 1: Figure S1A). Contrary to the 38.66% increase in incidences over the past 28 years, the ASIR was stable with 1.43/100,000 persons (95% UI, 1.1–1.56) in 1990 to 1.29/100,000 persons (95% UI,1.12–1.51) in 2017 (Additional file 1: Figure S1B). The ASIR in male subjects was slightly higher than that in female subjects over the past 28 years (Fig. 1a). The ratio of male to female incidence among different ages showed a bimodal distribution, with peaks in 5–9 years and 65–69 years age groups (Fig. 2).

**Fig. 3:**
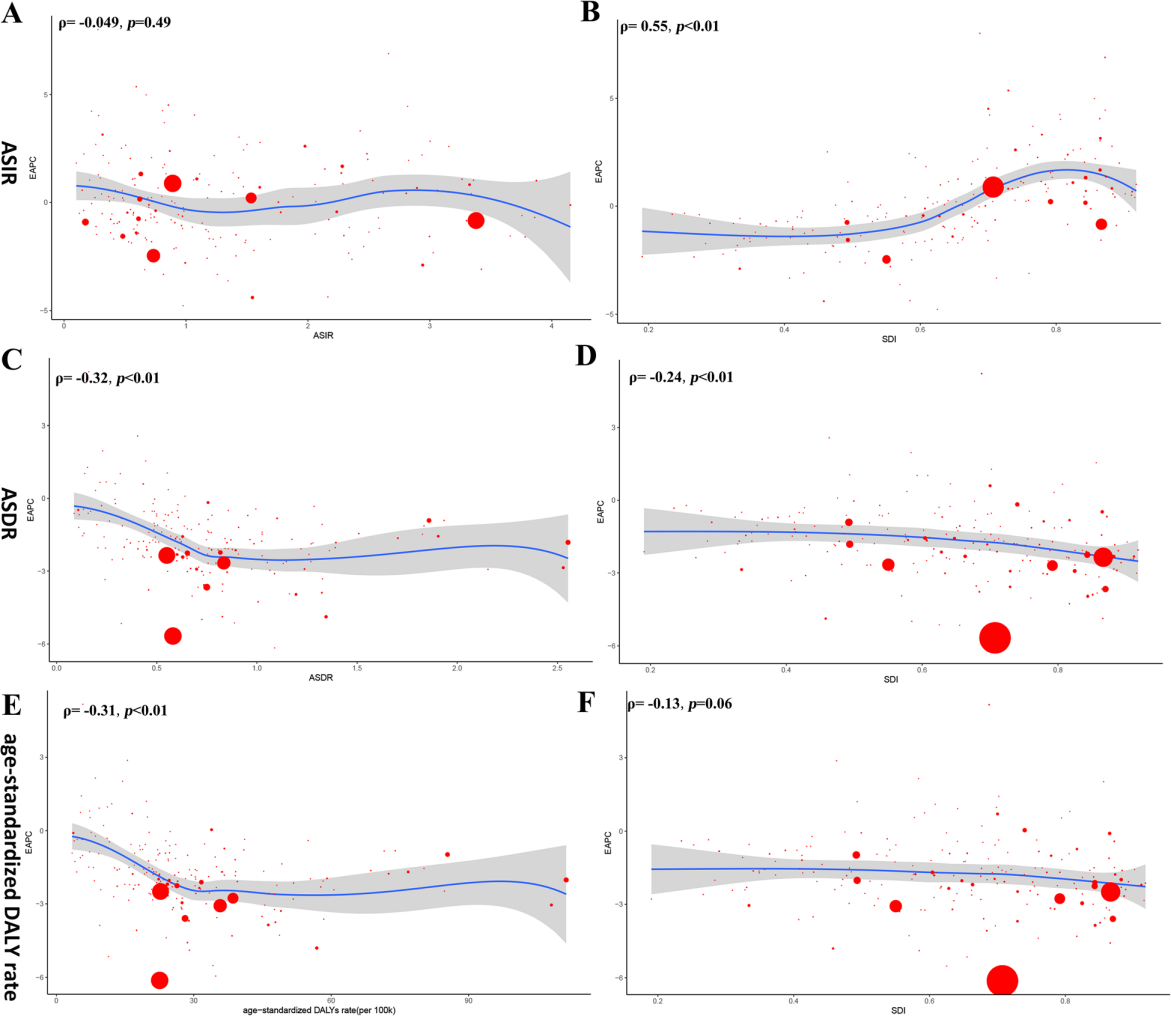
The correlation between EAPC and Hodgkin lymphoma ASR (incidence (**a**), death (**c**), and DALY (**e**)) in 1990 and HDI (incidence (**b**), death (**d**), and DALY (**f**)) in 2017. The circles represent countries that were available on SDI data. The size of circle represents the number of Hodgkin lymphoma patients. The *ρ* indices Pearson’s correlation coefficient and *p* values were derived from Pearson’s correlation analysis. ASR, age-standardized incidence/death/DALY rate; EAPC, estimated annual percentage change; SDI, socio-demographic index

**Fig. 4:**
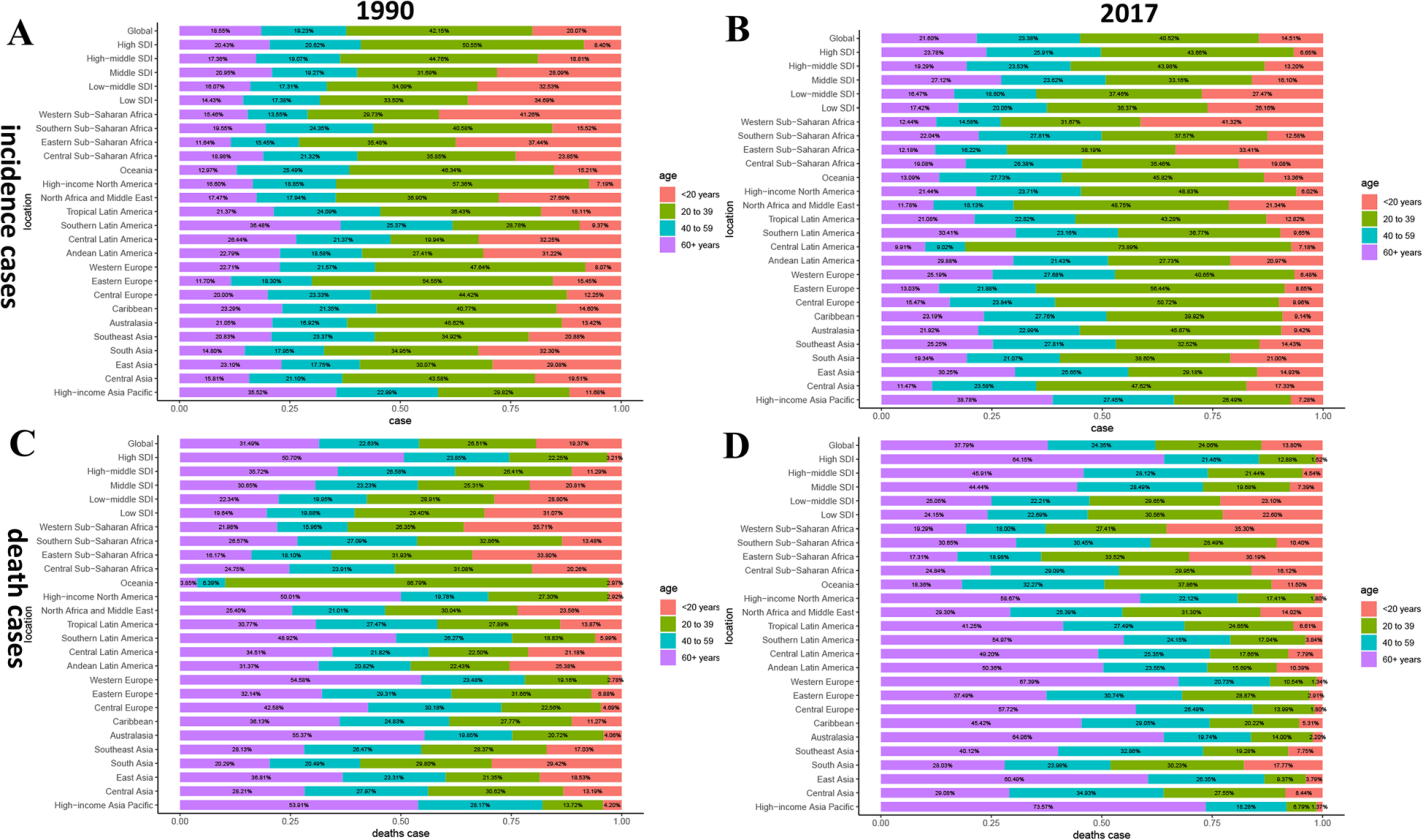
Distribution of different ages in Hodgkin lymphoma incidence/death cases by region. **a** Incidence in 1990. **b** Incidence in 2017. **c** Death rate in 1990. **d** Death rate in 2017

**Fig. 5:**
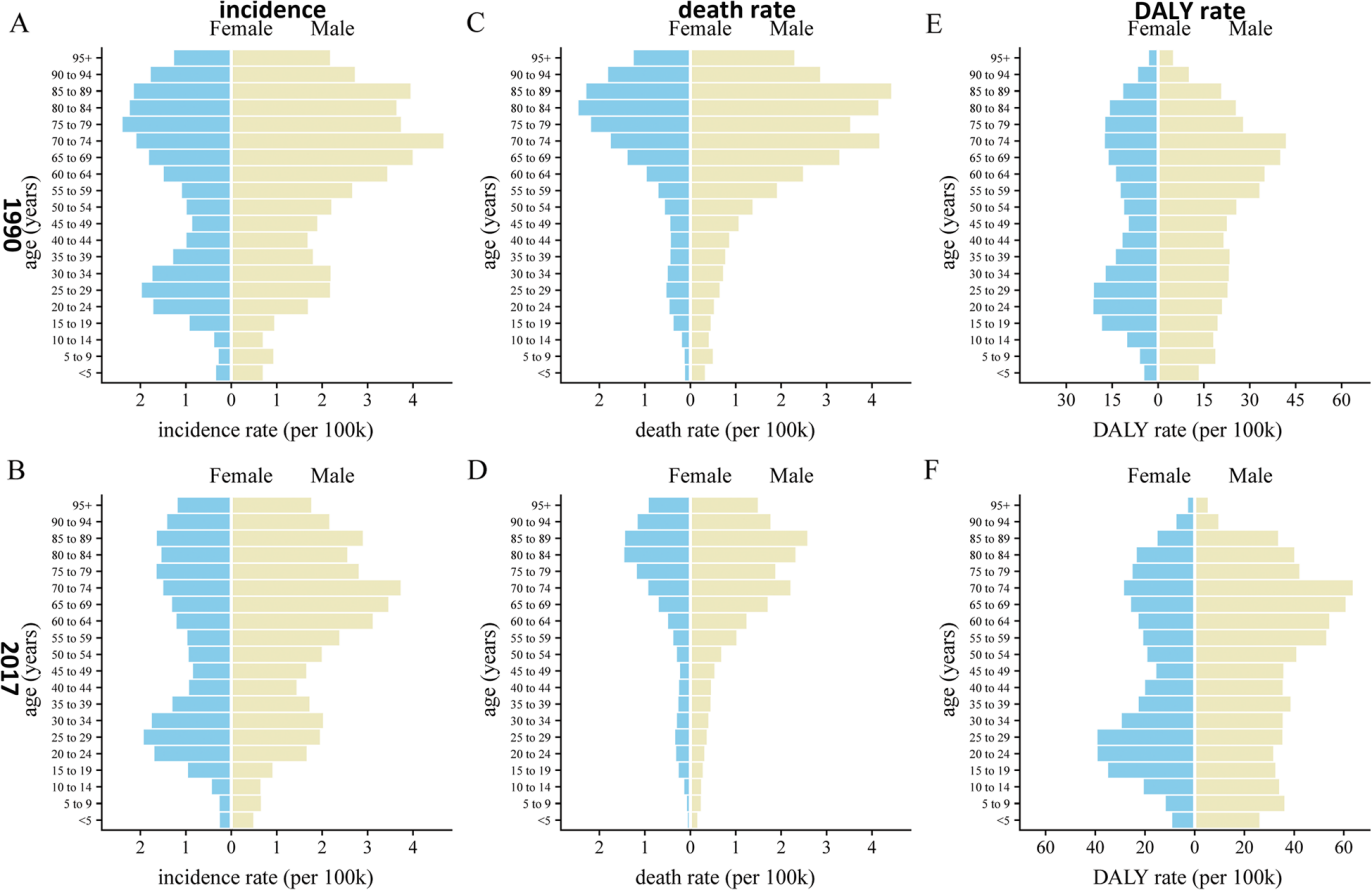
The incidence, death, and DALY rates of Hodgkin lymphoma in different age groups. **a** Incidence in 1990. **b** Incidence in 2017. **c** Death rate in 1990. **d** Death rate in 2017. **e** DALY rate in 1990. **f** DALY rate in 2017

Regarding SDI level analysis, as shown in Additional file 1: Figure S2 and Table 1, the ASIR in the low SDI region was on the decline with EAPCs of − 2.58 (95% CI, from − 2.66 to − 2.49). The ASIR in the other four SDI regions was stable. In addition, we found a positive correlation between EAPC and SDI (*ρ* = 0.55, *p* < 0.01, Fig. 3b) and a non-significant correlation between EAPC and the ASIR (Fig. 3a, Additional file 1: Figure S3A). We also found that the higher the SDI, the lower the proportion of young incidence cases among all HL incidence cases, while the proportion of elderly incident cases was relatively stable (Fig. 4a, b). The proportion of annual young incidence cases decreased year by year, while the proportion of elderly incident cases increased year by year, as shown in Additional file 1: Figure S4A. In 1990 and 2017, incidence showed a bimodal distribution and increased in people aged 20–39 years and 60+ years or higher (Fig. 5a). The peak of incidence of young incidence cases in the high SDI and high-middle SDI regions was higher than that of old patients, contrary to the peak of incidence in the middle, low-middle, and low SDI regions (Additional file 1: Figure S5).

**Fig. 6:**
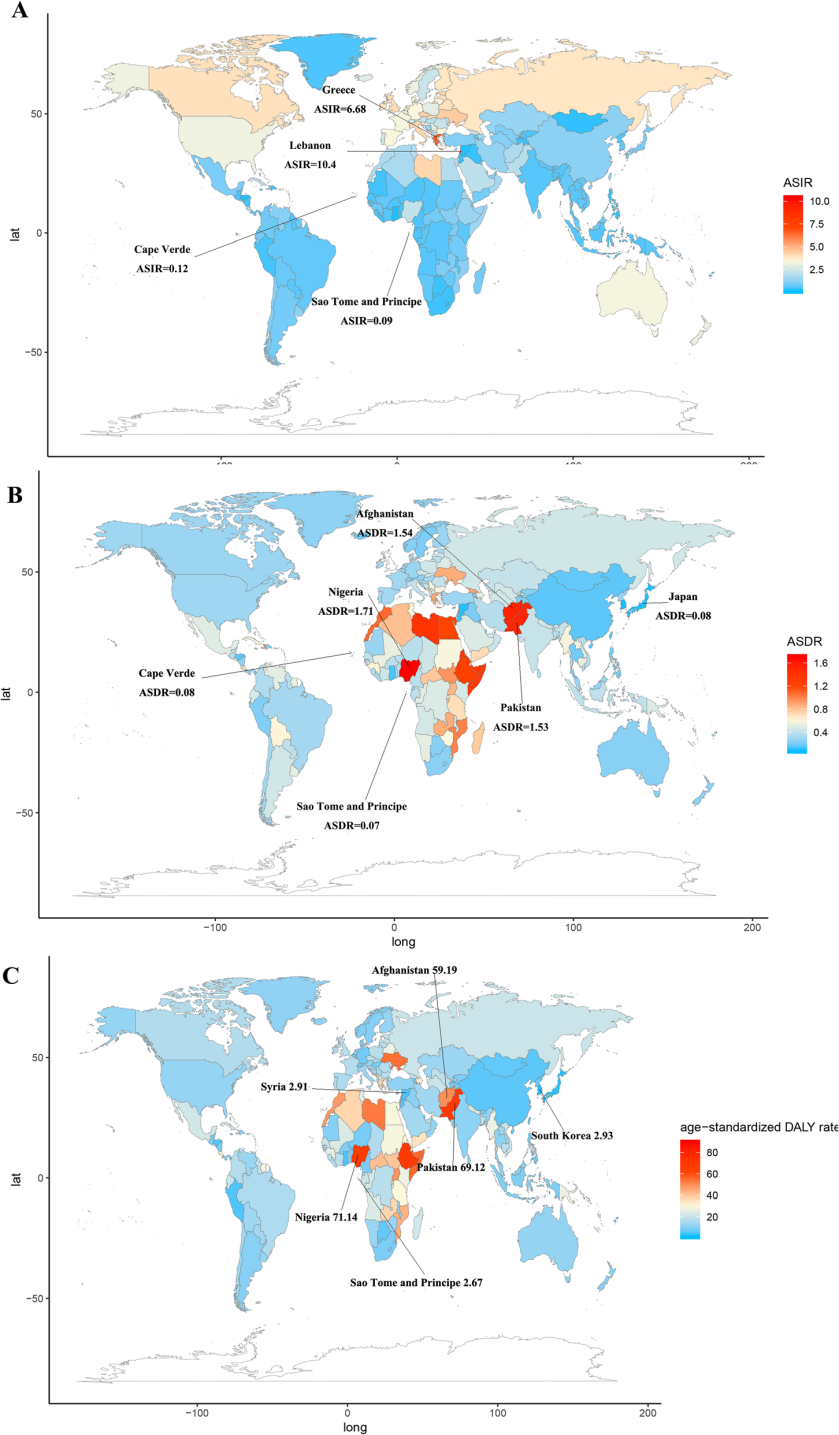
The global disease burden of Hodgkin lymphoma for both sexes in 194 countries. **a** The ASIR of Hodgkin lymphoma in 2017. **b** The ASDR of Hodgkin lymphoma in 2017. **c** The age-standardized DALY rate of Hodgkin lymphoma in 2017. ASIR, age-standardized incidence rate; ASDR, age-standardized death rate

On observation from the GBD regions and countries level, ASIR showed an upward trend in four countries (Bermuda, Singapore, Lebanon, and South Korea), a stable trend in 180 countries, and a downward trend in 10 countries (Equatorial Guinea, Bangladesh, Iraq, Rwanda, Myanmar, Cambodia, India, Mali, Niger, and Guatemala). Among female subjects, ASIR showed an upward trend in 10 countries (South Korea, Singapore, Lebanon, Virgin Islands, USA, Qatar, Mauritius, Japan, Ireland, Macedonia, and Ukraine), a stable trend in 181 countries, and a downward trend in three countries (Equatorial Guinea, Bangladesh, and Mozambique). Among male subjects, ASIR showed an upward trend in four countries (Equatorial Guinea, Bangladesh, Iraq, and Rwanda), a stable trend in 188 countries, and a downward trend in two countries (South Korea and Lebanon). The three countries with the highest ASIR were Lebanon, Greece, and Montenegro; the three countries with the lowest ASIR were Sao Tome and Principe, Cape Verde and Ghana; the three countries with the highest EAPC were Cuba, South Korea, and Lebanon; the three countries with the lowest ASIR were Equatorial Guinea, Bangladesh, and Iraq, and the details were listed in Additional file 1: Table S1–S3, Fig. 6 and Additional file 1: Figure S6–S10. As shown in Additional file 1: Table S6, most countries have a bimodal age distribution.

### The change in deaths due to HL

At the global level, the number of annual deaths was stable with 35,946 (95% UI, 27,329–39,412) deaths in 1990 and 32,560 (95% UI, 27,644–38,086) deaths in 2017. The number of deaths was stable over the past 28 years, while the ASDR decreased significantly with an EAPC of − 2.36 (95% CI, from − 2.43 to − 2.30), dropping from 0.75/100,000 persons (95% UI, 0.58–0.83) in 1990 to 0.41/100,000 persons (95% UI, 0.35–0.48) in 2017 (Table 2). The ASDR in male subjects was higher than that in female subjects over the past 28 years (Fig. 1b).

On analysis from the SDI level, the ASDR in all the SDI regions was found to have declined. In addition, we found a negative correlation between EAPC and SDI (*ρ* = − 0·24, *p* < 0.01, Fig. 3d) and a negative association between EAPC and ASDR (Fig. 3c, *ρ* = − 0.32, *p* < 0.01, Additional file 1: Figure S2B). Interestingly, the higher the SDI, the lower the proportion of young deaths, while the proportion of elderly deaths increased with SDI in 1990 and 2017 (Fig. 4c, d). The proportion of annual young deaths decreased year by year, while the proportion of elderly deaths increased year by year, as shown in Additional file 1: Figure S4B. As shown in Fig. 5b and Additional file 1: Figure S11, mortality showed a unimodal distribution and increased in people aged 50+ years. The difference between young male and female subjects was that female mortality was relatively stable and even slightly decreased with age, while young male mortality had been slowly increasing with age. The difference in deaths among different age groups between male and female subjects showed a bimodal distribution, with peaks on 5–9 years and 65–69 years (Additional file 1: Figure S12).

On observation from the GBD regions and countries level, ASDR in most regions declined and no country had a rising ASDR. The three countries with the highest ASDR were Nigeria, Afghanistan, and Pakistan; the three countries with the lowest ASDR were Sao Tome and Principe, Cape Verde, and Japan; details were shown in Additional file 1: Table S4, Additional file 1: Table S7, and Additional file 1: Figure S6–S10.

### The change in DALYs of HL

At the global level, there were 1,657,470 (95% UI, 1,228,330–1,843,220) DALYs in 1990 and 1,378,170 (95% UI, 1,155,060–1,624,390) DALYs in 2017. The number of DALYs was stable over the past 28 years, and the age-standardized DALY rate decreased significantly with an EAPC of − 2.29 (95% CI, from − 2.36 to − 2.21), dropping from 31.51/100,000 persons (95% UI, 23.56–34.73) in 1990 to 17.77/100,000 persons (95% UI, 14.87–20.93) in 2017 (Table 3). The age-standardized DALY rate of male subjects was higher than that of female subjects over the past 28 years (Fig. 1c). On analysis from the SDI level, the age-standardized DALY rate in all the SDI regions declined. In addition, we found a non-significant correlation between EAPC and SDI (*ρ* = − 0.13, *p* = 0.06, Fig. 3f) and a negative association between EAPC and the age-standardized DALY rate (Fig. 3e, *ρ* = − 0.31, *p* < 0.01, Additional file 1: Figure S3C). As shown in Fig. 5c and Additional file 1: Figure S13, the DALY rate showed a unimodal distribution in male subjects and a bimodal distribution in female subjects. The DALY difference among different age groups between male and female subjects showed a bimodal distribution, with peaks on 5–9 years and 55–59 years (Additional file 1: Figure S14).

On observation from the GBD regions and countries level, age-standardized DALY rate in most regions declined and no country had a rising trend. The three countries with the highest age-standardized DALY rate were Nigeria, Pakistan, and Afghanistan; the three countries with the lowest age-standardized DALY rate were Sao Tome and Principe, Syria, and South Korea; details were shown in Additional file 1: Table S5, Additional file 1: Table S8, and Additional file 1: Figure S5–S8.

## Discussion

To our knowledge, this study presents the latest trends and patterns of global incidence, mortality, and DALY of HL from 1990 to 2017, based on GBD 2017 results. Previous epidemiological studies of HL were conducted before the twenty-first century or focused on individual or several countries [7]. We believe that our research is more comprehensive and representative due to larger and updated data. Overall, the incidence of HL was higher in North America and Europe, and lower in Africa. The mortality of HL was higher in Africa and lower in East Asia and Australia. These conclusions were consistent with the data in the Global Cancer Observatory (GCO) (http://gco.iarc.fr). In GCO, we found that the incidence of HL in Australia, North America, and Europe was the highest and the mortality in Africa and Asia was the lowest. The mortality of HL was higher in Africa and lower in Eastern Asia, Europe, and Australia. For the high mortality rate of HL in Africa, African countries should pay more attention to the treatment of HL. Previous study has shown a significant increase in the incidence of HL in some populations, such as those with infectious mononucleosis or direct relatives with HD cases. For these high-risk people in high SDI countries, we believe that it is necessary to strengthen their screening for HL [5]. Although the major reason for the decline in mortality in recent decades was the improvement of treatment methods, these reductions were also due to certain human factors [13]: for instance, some histological types of HL have been reclassified as non-HL [14]; over time, pathological diagnosis of HL would tend to shift these categories to non-HL [15, 16]; and the decline in autopsy rates would lead to a decline in incidence, as many elderly HL cases were diagnosed only after death [17]. Interestingly, the countries with the highest or lowest incidence or mortality were mostly coastal or island countries. For instance, ASIR of Lebanon and Greece on the Mediterranean coast was much higher than that in other countries. The causes of this phenomenon are still unclear and require further study.

Incidence showed a bimodal distribution and increased in people aged 20–39 years and 60 years or higher. Incidence in most countries had a bimodal distribution of age. However, some countries like the Russian Federation and Canada showed a unimodal distribution in the recent years, peaking in young people, due to the increased incidence of nodular sclerosis subtypes in HL. Similar distribution was described in previous studies [18–20]. The peak was higher in adolescents in high SDI countries and for older people in low SDI countries. The lowest incidence of HL was in childhood, which was consistent with a previous study [21]. In addition, worldwide incidence, mortality, and DALY of male subjects were higher than those of female subjects. However, male and female subjects of different ages had different burdens. The incidence in female and male subjects aged 15–30 years old was close. The incidence in male subjects aged < 10 years old and 45–75 years old was twice or more than twice than that in female subjects. There were strong evidences, since the 1960s, showing the role of EBV in the etiology of HL in the childhood and in the elderly, which may explain the first peak in the childhood and the second peak in the elderly in some poor regions [22, 23]. EBV was usually found in 70% of HL mixed cellularity (morphological type common in childhood and in the elderly), and there was a male bias in EBV associated HL patients, which may explain the biggest difference in the proportion of boys and girls in the low SDI regions [24–26]. Previous study also found that childhood HL was associated with living conditions [27]. The incidence of children decreased over the past 28 years, probably due to the improved living conditions. As for the higher incidence in developed countries, it was likely more due to higher proportion of nodular sclerosis subtypes (accounting for about 70% of HL cases in the developed countries), for lacking explanatory factors [27, 28]. The mortality and DALY age distribution difference between male and female subjects was similar to that of incidence.

The proportion of annual young incidences and deaths in all HL cases decreased year by year, while the proportion of elderly incidences and deaths cases increased. This could be attributed to population growth and population aging. It is well known that aging and fewer births in developed countries has a serious concern in the recent years. We also observed a phenomenon that the higher the SDI, the lower the proportion of children and higher the proportion of the elderly, which confirmed this conjecture. Even different SDI regions in the same continent showed this difference. For instance, the incident and death HL population in high-income Asia Pacific tended to be aging, compared with other regions in Asia. Moreover, in recent years, new treatment schedule and the improvement in supportive nursing measures had led to an unprecedented long-term cure rate of HL treatment, which further promoted this trend [29, 30].

In addition, we found a significant negative correlation between the variation of ASDR between 1990 and 2017 and the baseline ASDR in 1990. For the countries with higher ASDR in 1990, the change in ASDR is more significant. The results could be explained as countries with higher ASDR were more likely to consider HL as a top priority in prevention plans, due to the public health factor. A similar trend of age-standardized DALY rate was also found. We also found a significant negative correlation between the variation of ASDR and SDI. For countries with higher SDI, the downward trend in ASDR was more obvious possibly due to better health care in countries with high SDI.

Some limitations were unavoidable in this study. First, the accuracy of the results depended on the quality and the quantity of data in GBD. Second, data quality in some underdeveloped areas, like Africa and Latin America, was not guaranteed which may cause data on some underdeveloped regions inaccuracy because many countries did not have reliable mortality information systems and population-based cancer registries are rare. Third, the GBD study took the country as its basic unit and neglected the influence of race. Last, due to the lack of relevant data, no further study was conducted on the etiology and risk information related to HL, which might better explain the changing trend and age distribution of HL and help local governments formulate more detailed policies to reduce the incidence of HL.

## Conclusion

In summary, developed areas had higher incidence, lower mortality rates, whereas underdeveloped areas had lower incidence rate and higher mortality rates. Globally, incidence of HL was stable, while mortality and DALY rate of HL had been decreasing from 1990 to 2017. Compared with lower and decreasing ASIR in the low SDI region, ASIR in the high SDI region was always high, signifying the need for larger investment in research to reduce the incidence of HL in developed countries. And the low SDI country should attach importance to improve HL treatment and prolong patient life. It was noteworthy that the change pattern was heterogeneous across sex, age, SDI, region, and country. The incidence was the lowest in childhood and the highest in youth and old ages, which highlighted the need to strengthen HL screening in these two age groups. The incidence and mortality in male subjects was always higher than that in female subjects. These differences have to be taken into account for policymakers to allocate limited resources and formulate relevant policies more rationally.

## Supplementary information

**Additional file 1.** Supplementary tables and figures.

## Data Availability

The datasets generated during and/or analyzed during the current study are available from the Global Health Data Exchange query tool (http://ghdx.healthdata.org/gbd-results-tool).

## Abbreviations

ASDR: Age-standardized death rate
ASIR: Age-standardized incidence rate
ASR: Age-standardized incidence/death/DALY rate
DALY: Disability adjusted life-years
EAPC: Estimated annual percentage changes
GBD: The Global Burden of Disease
GCO: Global Cancer Observatory
HL: Hodgkin lymphoma
SDI: Social-demographic index
YLDs: The years lived with disability
YLLs: Years of life lost

## References

[1] Bray F, Ferlay J, Soerjomataram I, Siegel RL, Torre LA, Jemal A (2018). Global cancer statistics 2018: Globocan estimates of incidence and mortality worldwide for 36 cancers in 185 countries. CA Cancer J Clin.

[2] Shin-ichi N (2006). Epidemiology and pathologic features of Hodgkin lymphoma. Int J Hematol.

[3] Punnett A, Tsang RW, Hodgson DC (2010). Hodgkin lymphoma across the age spectrum: epidemiology, therapy, and late effects. Semin Radiat Oncol.

[4] Ansell SM (2016). Hodgkin lymphoma: 2016 update on diagnosis, risk-stratification, and management. Am J Hematol.

[5] Cartwright RA, Watkins G (2004). Epidemiology of Hodgkin’s disease: a review. Hematol Oncol.

[6] Swerdlow AJ (2003). Epidemiology of Hodgkin’s disease and non-Hodgkin's lymphoma. Eur J Nucl Med Mol Imaging.

[7] Correa P, O'Conor GT (1971). Epidemiologic patterns of Hodgkin’s disease. Int J Cancer.

[8] Naghavi M, Abajobir AA, Abbafati C, Abbas KM, Abd-Allah F, Abera SF, et al. Global, regional, and national age-sex specific mortality for 264 causes of death, 1980–2016: a systematic analysis for the Global Burden of Disease study 2016. Lancet. 2017;390:1151–210. 10.1016/S0140-6736(17)32152-9 PMC5605883 28919116

[9] Ebrahimi Hedyeh, Amini Erfan, Pishgar Farhad, Moghaddam Sahar Saeedi, Nabavizadeh Behnam, Rostamabadi Yasna, Aminorroaya Arya, Fitzmaurice Christina, Farzadfar Farshad, Nowroozi Mohammad Reza, Black Peter C., Daneshmand Siamak (2019). Global, Regional and National Burden of Bladder Cancer, 1990 to 2016: Results from the GBD Study 2016. Journal of Urology.

[10] Global, regional, and national age-sex specific all-cause and cause-specific mortality for 240 causes of death, 1990–2013: a systematic analysis for the Global Burden of Disease study 2013. Lancet. 2015;385:117–71. 10.1016/S0140-6736(14)61682-2 PMC4340604 25530442

[11] Liu Z, Jiang Y, Yuan H, Fang Q, Cai N, Suo C (2019). The trends in incidence of primary liver cancer caused by specific etiologies: results from the Global Burden of Disease study 2016 and implications for liver cancer prevention. J Hepatol.

[12] Gao S, Yang WS, Bray F, Va P, Zhang W, Gao J (2012). Declining rates of hepatocellular carcinoma in urban shanghai: incidence trends in 1976-2005. Eur J Epidemiol.

[13] Swerdlow A, Silva IDS, Doll R, Swerdlow A, Silva IDS, Doll R (2003). Cancer incidence and mortality in England and Wales: trends and risk factors. J R Soc Med.

[14] Banks PM (1992). Changes in diagnosis of non-Hodgkin’s lymphomas over time. Cancer Res.

[15] Glaser SL, Swartz WG (1990). Time trends in Hodgkin’s disease incidence. The role of diagnostic accuracy. Cancer.

[16] Martinsson U, Glimelius B, Sundström C (1992). Lymphoma incidence in a Swedish county during 1969–1987. Acta Radiol Ther Phys Biol.

[17] Hasle H, Mellemgaard A (1993). Hodgkin’s disease diagnosed post mortem: a population based study. Br J Cancer.

[18] Grufferman S, Delzell E (1984). Epidemiology of Hodgkin’s disease. Epidemiol Rev.

[19] MacMahon B (1966). Epidemiology of Hodgkin’s disease. Cancer Res.

[20] Gutensohn N, Cole P (1980). Epidemiology of Hodgkin’s disease. Semin Oncol.

[21] Fraumeni JF, Li FP (1969). Hodgkin’s disease in childhood: an epidemiologic study. J Natl Cancer Inst.

[22] Jarrett AF, Armstrong AA, Alexander E (1996). Epidemiology of EBV and Hodgkin’s lymphoma. Ann Oncol.

[23] Glaser SL, Jarrett RF (1996). The epidemiology of Hodgkin’s disease. Baillieres Clin Haematol.

[24] Glaser SL, Lin RJ, Stewart SL, Ambinder RF, Jarrett RF, Brousset P (1997). Epstein-barr virus-associated hodgkin's disease: epidemiologic characteristics in international data. Int J Cancer.

[25] Sleckman BG, Mauch PM, Ambinder RF, Mann R, Pinkus GS, Kadin ME (1998). Epstein-barr virus in Hodgkin’s disease: correlation of risk factors and disease characteristics with molecular evidence of viral infection. Cancer Epidemiol Biomark Prev.

[26] Alexander FE, Jarrett RF, Lawrence D, Armstrong AA, Freeland J, Gokhale DA (2000). Risk factors for Hodgkin’s disease by epstein-barr virus (ebv) status: prior infection by EBV and other agents. Br J Cancer.

[27] Jarrett RF (2002). Viruses and Hodgkin’s lymphoma. Ann Oncol.

[28] Shanbhag S, Ambinder RF (2018). Hodgkin lymphoma: a review and update on recent progress. CA Cancer J Clin.

[29] Brenner H, Gondos A, Pulte D (2008). Ongoing improvement in long-term survival of patients with hodgkin disease at all ages and recent catch-up of older patients. Blood.

[30] Bouliotis G, Bessell EM (2015). Hodgkin disease (1973-2002): long-term survival and cure fractions. Leuk Lymphoma.

