# Supplementary material for: Global, regional, and national burden of Hodgkin lymphoma from 1990 to 2017: estimates from the 2017 Global Burden of Disease study
Source: J Hematol Oncol. 2019 Oct 22;12:107. doi: 10.1186/s13045-019-0799-1 (PMC6805485; doi:10.1186/s13045-019-0799-1)

Global, Regional, and National Burden of Hodgkin Lymphoma from 1990 to 2017: Estimates from the 2017 Global Burden of Disease study.

Linghui Zhou[1,2,†], Yujiao Deng[1,2,†], Na Li[1,2,†], Yi Zheng[1,2], Tian Tian[2], Zhen Zhai[1,2], Si Yang[1,2], Qian Hao[2], Ying Wu[1,2], Dingli Song[2], Dai Zhang[2], Jun Lyu[3], and Zhijun Dai[1,2]

1. Department of Breast Surgery, The First Affiliated Hospital, College of Medicine, Zhejiang University, Hangzhou 310003, China;

2. Department of Oncology, The Second Affiliated Hospital of Xi'an Jiaotong University, Xi'an 710004, China;

3. Clinical Research Center, The First Affiliated Hospital of Xi'an Jiaotong University, Xi'an, 710061, China.

Correspondence: Zhijun Dai, Department of Breast Surgery, The First Affiliated Hospital, College of Medicine, Zhejiang University, Hangzhou 310003, China; (E-Mail: dzj0911@126.com) or Jun Lyu, Clinical Research Center, First Affiliated Hospital of Xi'an Jiaotong University, Xi'an, Shaanxi, 710061, China (E-Mail: lujun2006@xjtu.edu.cn).

LH Z, YJ D and N L contributed equally to this work.

Supplementary Tables

Supplement Table S1 Three countries with the largest and lowest number of incidence, death, or DALY.

Supplement Table S2 Three regions with the largest and lowest number of incidence, death, or DALY.

Supplementary Table S3 The incident cases and age-standardized incidence rate of Hodgkin lymphoma in 1990 and 2017, and its temporal trends from 1990 to 2017.

Supplementary Table S4 The death cases and age-standardized death rate of Hodgkin lymphoma in 1990 and 2017, and its temporal trends from 1990 to 2017.

Supplementary Table S5 The DALY and age-standardized DALY rate of Hodgkin lymphoma in 1990 and 2017, and its temporal trends from 1990 to 2017.

Supplementary Table S6 Age distribution of incidence (per 100,000) for Hodgkin lymphoma in different countries in 2017.

Supplementary Table S7 Age distribution of death rate (per 100,000) for Hodgkin lymphoma in different countries in 2017.

Supplementary Table S8 Age distribution of DALYs rate (per 100,000) for Hodgkin lymphoma in different countries in 2017.

Supplementary Figures

Sup Figure 1 The incident cases (A), age standardized incidence (B), death (C) and DALY (D) rates of Hodgkin lymphoma between 1990 and 2017 among sexes.

Sup Figure 2 The EAPC of Hodgkin lymphoma ASR from 1990 to 2017, by sex and region. A: The EAPC of ASIR; B: The EAPC of ASDR; C: The EAPC of age-standardized DALY rate.

Sup Figure 3 The age standardized incidence (A), death (B) and DALY (C) rates of Hodgkin lymphoma per 100,000 population among regions based on SDI in 2017.

Sup Figure 4 The proportion of different ages in Hodgkin lymphoma incidence (A) and death (B) by years.

Sup Figure 5 Distribution of different ages in Hodgkin lymphoma incidence in global (A), high SDI (B), high-middle SDI (C), middle SDI (D), middle-low SDI (E), low SDI (F). SDI, socio-demographic index.

Sup Figure 6 The global EAPC of Hodgkin lymphoma for both sexes in 194 countries. A: The EAPC of ASIR; B: The EAPC of ASDR; C: The EAPC of age-standardized DALY rate.

Sup Figure 7 The global EAPC of Hodgkin lymphoma for female in 194 countries. A: The EAPC of ASIR; B: The EAPC of ASDR; C: The EAPC of age-standardized DALY rate.

Sup Figure 8 The global EAPC of Hodgkin lymphoma for male in 194 countries. A: The EAPC of ASIR; B: The EAPC of ASDR; C: The EAPC of age-standardized DALY rate.

Sup Figure 9 The global disease burden of Hodgkin lymphoma for female in 194 countries. (A) The ASIR of Hodgkin lymphoma in 2017; (B) The ASDR of Hodgkin lymphoma in 2017; (C) The age standardized DALY rate of Hodgkin lymphoma in 2017. ASIR: age-standardized incidence rate; ASDR: age-standardized death rate.

Sup Figure 10 The global disease burden of Hodgkin lymphoma for male in 194 countries. (A) The ASIR of Hodgkin lymphoma in 2017; (B) The ASDR of Hodgkin lymphoma in 2017; (C) The age standardized DALY rate of Hodgkin lymphoma in 2017. ASIR: age-standardized incidence rate; ASDR: age-standardized death rate.

Sup Figure 11 Distribution of different ages in Hodgkin lymphoma death rate in global (A), high SDI (B), high-middle SDI (C), middle SDI (D), middle-low SDI (E), low SDI (F). SDI, socio-demographic index.

Sup Figure 12 The ratio of male to female ASDR among different age groups in global (A), high SDI (B), high-middle SDI (C), middle SDI (D), middle-low SDI (E), low SDI (F). SDI, socio-demographic index. ASDR, age-standardized death rate.

Sup Figure 13 Distribution of different ages in Hodgkin lymphoma DALYs rate in global (A), high SDI (B), high-middle SDI (C), middle SDI (D), middle-low SDI (E), low SDI (F). SDI, socio-demographic index.

Sup Figure 14 The ratio of male to female age standardized DALY rate among different age groups in global (A), high SDI (B), high-middle SDI (C), middle SDI (D), middle-low SDI (E), low SDI (F). SDI, socio-demographic index.

Supplement Table S1 Three countries with the largest and lowest number of incidence, death, or DALY.

Measure	sex	Top three countries			Bottom three countries		
**2017ASR (per 100,000 people)**							
ASIR							
	both	Lebanon (10.4)	Greece (6.68)	Montenegro (4.71)	Sao Tome and Principe (0.09)	Cape Verde (0.12)	Ghana (0.14)
	female	Lebanon (10.33)	Greece (5.91)	Libya (5.04)	Honduras (0.05)	Sao Tome and Principe (0.06)	Cape Verde (0.08)
	male	Lebanon (10.47)	Greece (7.48)	Montenegro (5.36)	Sao Tome and Principe (0.12)	Cape Verde (0.15)	Ghana (0.18)
ASDR							
	both	Nigeria (1.71)	Afghanistan (1.54)	Pakistan (1.53)	Sao Tome and Principe (0.07)	Cape Verde (0.08)	Japan (0.08)
	female	Nigeria (1.62)	Libya (1.45)	Afghanistan (1.19)	Honduras (0.04)	Maldives (0.04)	Syria (0.04)
	male	Eritrea (2.08)	Pakistan (1.96)	Afghanistan (1.91)	Sao Tome and Principe (0.1)	Cape Verde (0.12)	Japan (0.12)
Age Standardized DALY Rate							
	both	Nigeria (71.74)	Pakistan (69.12)	Afghanistan (59.19)	Sao Tome and Principe (2.67)	Syria (2.91)	South Korea (2.93)
	female	Nigeria (67.39)	Libya (61.26)	Pakistan (51.59)	Honduras (1.26)	Maldives (1.56)	Syria (1.57)
	male	Eritrea (89.57)	Pakistan (85.4)	Nigeria (75.84)	Sao Tome and Principe (3.59)	South Korea (3.92)	Maldives (4.04)
**1990-2017 increase times**							
Incidence(cases)							
	both	Qatar (20.64)	United Arab Emirates (8.04)	Lebanon (7.76)	Bangladesh (-0.44)	Georgia (-0.40)	Greenland (-0.38)
	female	Qatar (15.27)	Cuba (11.07)	Lebanon (8.08)	Georgia (-0.43)	Hungary (-0.34)	Austria (-0.33)
	male	Qatar (21.07)	United Arab Emirates (8.34)	Lebanon (7.46)	Bangladesh (-0.51)	Greenland (-0.43)	Georgia (-0.38)
Death(cases)							
	both	Cuba (4.42)	Qatar (3.69)	United Arab Emirates (3.55)	Hungary (-0.62)	Austria (-0.59)	Estonia (-0.56)
	female	Cuba (6.88)	Dominican Republic (3.15)	Brunei (2.56)	Austria (-0.71)	Hungary (-0.67)	China (-0.56)
	male	United Arab Emirates (3.84)	Qatar (3.80)	Cuba (3.45)	Hungary (-0.58)	Estonia (-0.57)	Bangladesh (-0.54)
DALY(Year)							
	both	Qatar (3.99)	Cuba (3.56)	United Arab Emirates (3.10)	Hungary (-0.66)	China (-0.64)	Austria (-0.61)
	female	Cuba (5.33)	Brunei (2.60)	Dominican Republic (2.41)	Hungary (-0.71)	Austria (-0.70)	China (-0.68)
	male	Qatar (4.19)	United Arab Emirates (3.35)	Cuba (2.85))	Hungary (-0.64)	China (-0.63)	Estonia (-0.62)
**EAPC**							
Incidence							
	both	Cuba (8)	South Korea (6.88)	Lebanon (5.36)	Equatorial Guinea (-4.78)	Bangladesh (-4.39)	Iraq (-4.37)
	female	Cuba (9.89)	South Korea (8.51)	Brunei (6.51)	Iraq (-4.37)	Equatorial Guinea (-3.72)	Bangladesh (-3.46)
	male	Cuba (6.8)	South Korea (5.83)	Lebanon (5.08)	Equatorial Guinea (-5.34)	Bangladesh (-4.68)	Iraq (-4.41)
Death							
	both	Cuba (5.21)	Zimbabwe (2.58)	Guyana (1.97)	Bahrain (-6.16)	China (-5.67)	Maldives (-5.15)
	female	Cuba (6.92)	Guyana (2.92)	Brunei (2.56)	China (-6.09)	Austria (-5.99)	Bahrain (-5.65)
	male	Cuba (4.34)	Zimbabwe (2.97)	Guyana (1.49)	Bahrain (-6.47)	Equatorial Guinea (-5.54)	China (-5.42)
DALY							
	both	Cuba (5.17)	Zimbabwe (2.88)	Guyana (2.16)	China (-6.13)	Bahrain (-5.95)	Equatorial Guinea (-5.52)
	female	Cuba (6.83)	Brunei (3.22)	Guyana (3.12)	China (-6.51)	Bahrain (-5.54)	Austria (-5.38)
	male	Cuba (4.31)	Zimbabwe (3.33)	Guyana (1.67)	Bahrain (-6.32)	Equatorial Guinea (-6.05)	China (-5.91)

Supplement Table S2 Three regions with the largest and lowest number of incidence, death, or DALY.

Measure	sex	Top three regions			Bottom three regions		
**2017ASR (per 100,000 people)**							
ASIR							
	Both	Eastern Europe (3.99)	Western Europe (3.58)	Australasia (3.26)	Southern Sub-Saharan Africa (0.40)	Southeast Asia (0.53)	Oceania (0.54)
	female	Eastern Europe (4.33)	Western Europe (2.88)	Australasia (2.84)	Southern Sub-Saharan Africa (0.24)	Oceania (0.33)	Central Sub-Saharan Africa (0.36)
	male	Western Europe (4.29)	Eastern Europe (3.73)	Australasia (3.70)	Southern Sub-Saharan Africa (0.59)	Andean Latin America (0.69)	Southeast Asia (0.72)
ASDR							
	Both	Western Sub-Saharan Africa (1.00)	Eastern Sub-Saharan Africa (0.89)	North Africa and Middle East (0.62)	High-income Asia Pacific (0.08)	East Asia (0.15)	Australasia (0.24)
	female	Western Sub-Saharan Africa (0.89)	Eastern Sub-Saharan Africa (0.53)	North Africa and Middle East (0.46)	High-income Asia Pacific (0.05)	East Asia (0.10)	Southern Sub-Saharan Africa (0.18)
	male	Eastern Sub-Saharan Africa (1.28)	Western Sub-Saharan Africa (1.12)	North Africa and Middle East (0.79)	High-income Asia Pacific (0.12)	East Asia (0.20)	Australasia (0.31)
Age Standardized DALY Rate							
	Both	Western Sub-Saharan Africa (41.46)	Eastern Sub-Saharan Africa (37.39)	Eastern Europe (25.61)	High-income Asia Pacific (3.17)	East Asia (5.51)	Australasia (9.96)
	female	Western Sub-Saharan Africa (36.80)	Eastern Sub-Saharan Africa (23.50)	Eastern Europe (20.10)	High-income Asia Pacific (2.07)	East Asia (3.65)	Southern Sub-Saharan Africa (7.24)
	male	Eastern Sub-Saharan Africa (51.78)	Western Sub-Saharan Africa (46.09)	Eastern Europe (32.19)	High-income Asia Pacific (4.32)	East Asia (7.32)	Australasia (12.06)
**1990-2017 increase in the number of cases/years (-fold)**							
Incidence(cases)							
	both	Caribbean (1.91)	High-income Asia Pacific (1.52)	North Africa and Middle East (1.36)	South Asia (0.05)	Central Europe (0.10)	Eastern Europe (0.12)
	female	Caribbean (2.51)	High-income Asia Pacific (1.76)	North Africa and Middle East (1.68)	High-income North America (0.14)	Eastern Europe (0.17)	Central Europe (0.19)
	male	Caribbean (1.60)	High-income Asia Pacific (1.42)	North Africa and Middle East (1.17)	South Asia (-0.03)	Central Europe (0.02)	Eastern Europe (0.07)
Death(cases)							
	both	Caribbean (0.98)	Oceania (0.81)	Central Sub-Saharan Africa (0.59)	East Asia (-0.52)	Central Europe (-0.46)	Western Europe (-0.32)
	female	Caribbean (1.33)	Oceania (0.79)	Central Sub-Saharan Africa (0.63)	East Asia (-0.55)	Central Europe (-0.46)	Western Europe (-0.32)
	male	Caribbean (0.83)	Oceania (0.81)	Southern Sub-Saharan Africa (0.61)	East Asia (-0.49)	Central Europe (-0.45)	Western Europe (-0.32)
DALY(Year)							
	both	Oceania (0.76)	Caribbean (0.70)	Central Sub-Saharan Africa (0.51)	East Asia (-0.63)	Central Europe (-0.53)	Western Europe (-0.41)
	female	Caribbean (0.95)	Oceania (0.77)	Western Sub-Saharan Africa (0.58)	East Asia (-0.67)	Central Europe (-0.52)	Western Europe (-0.40)
	male	Oceania (0.75)	Caribbean (0.59)	Southern Sub-Saharan Africa (0.54)	East Asia (-0.62)	Central Europe (-0.53)	Western Europe (-0.41)
**EAPC**							
Incidence							
	both	High-income Asia Pacific (3.71)	Caribbean (2.61)	Australasia (1.17)	South Asia (-2.10)	Eastern Sub-Saharan Africa (1.94)	Western Sub-Saharan Africa (1.63)
	female	High-income Asia Pacific (4.47)	Caribbean (3.55)	Australasia (1.19)	Eastern Sub-Saharan Africa (-2.21)	South Asia (-1.66)	Western Sub-Saharan Africa (1.22)
	male	High-income Asia Pacific (3.20)	Caribbean (2.04)	Australasia (1.14)	South Asia (-2.23)	Western Sub-Saharan Africa (1.91)	Eastern Sub-Saharan Africa (1.76)
Death							
	both	Caribbean (0.81)	Oceania (-0.30)	Southern Sub-Saharan Africa (-0.30)	East Asia (-5.55)	Central Europe (-3.22)	Western Europe (-2.62)
	female	Caribbean (1.57)	High-income Asia Pacific (-0.12)	Oceania (-0.47)	East Asia (-5.98)	Central Europe (-3.28)	Western Europe (-2.55)
	male	Caribbean (0.44)	Southern Sub-Saharan Africa (-0.18)	Oceania (-0.22)	East Asia (-5.30)	Central Europe (-3.19)	Western Europe (-2.74)
DALY							
	both	Caribbean (0.61)	High-income Asia Pacific (-0.16)	Southern Sub-Saharan Africa (-0.32)	East Asia (-5.99)	Central Europe (-3.18)	Southeast Asia (-2.58)
	female	Caribbean (1.33)	High-income Asia Pacific (0.47)	Oceania (-0.47)	East Asia (-6.38)	Central Europe (-3.05)	Southeast Asia (-2.67)
	male	Caribbean (0.25)	Southern Sub-Saharan Africa (-0.16)	Oceania (-0.26)	East Asia (-5.77)	Central Europe (-3.27)	Western Europe (-2.67)

Supplementary Table S3. The incident cases and age-standardized incidence rate of Hodgkin lymphoma in 1990 and 2017, and its temporal trends from 1990 to 2017.

Nation	Sex	Incident Cases No. (95% UI)		Change in absolute number (%)	ASIR per 100,000 No. (95% UI)		1990-2017 EAPC No. (95 CI)
		1990	2017		1990	2017	
Afghanistan	Both	169.14 (64.94 - 268.1)	370.16 (263.32 - 511.98)	118.85	2.17 (0.85 - 3.46)	1.76 (1.27 - 2.46)	-0.8 (-1.14 - -0.45)
Albania	Both	37.86 (28.88 - 60.46)	59.48 (38.19 - 96.72)	57.11	1.23 (0.96 - 2.01)	2.13 (1.35 - 3.53)	2.17 (2.07 - 2.27)
Algeria	Both	377.83 (267.71 - 463.36)	693.4 (479.28 - 884.67)	83.52	1.76 (1.34 - 2.13)	1.72 (1.2 - 2.19)	0.01 (-0.03 - 0.06)
American Samoa	Both	0.07 (0.06 - 0.11)	0.12 (0.1 - 0.17)	71.43	0.21 (0.16 - 0.29)	0.25 (0.2 - 0.33)	1.03 (0.76 - 1.3)
Andorra	Both	1.95 (1.36 - 2.7)	3.3 (2.57 - 4.71)	69.23	3.06 (2.13 - 4.19)	3.81 (2.97 - 5.35)	0.57 (0.2 - 0.94)
Angola	Both	65.19 (40.44 - 94.12)	93.77 (73.05 - 125.36)	43.84	0.9 (0.61 - 1.25)	0.52 (0.39 - 0.7)	-2.15 (-2.36 - -1.94)
Antigua and Barbuda	Both	0.16 (0.13 - 0.23)	0.35 (0.26 - 0.47)	118.75	0.28 (0.23 - 0.4)	0.37 (0.28 - 0.51)	1.28 (1.1 - 1.46)
Argentina	Both	318.83 (274.36 - 420.23)	421.42 (330.87 - 630.4)	32.18	0.97 (0.83 - 1.27)	0.88 (0.69 - 1.32)	-0.64 (-0.84 - -0.44)
Armenia	Both	7.36 (5.41 - 13.48)	21.16 (11.64 - 27.49)	187.50	0.23 (0.17 - 0.4)	0.64 (0.35 - 0.85)	4.23 (3.08 - 5.39)
Australia	Both	408.91 (310.11 - 482.05)	842.69 (684.85 - 1042.5)	106.08	2.27 (1.72 - 2.71)	3.3 (2.66 - 4.07)	1.36 (1.09 - 1.64)
Austria	Both	272.02 (170.07 - 337.12)	220.77 (168.79 - 359.85)	-18.84	3 (1.91 - 3.74)	2.21 (1.64 - 3.71)	-1.58 (-1.96 - -1.2)
Azerbaijan	Both	85.63 (55.36 - 109.67)	169.15 (101.84 - 237.89)	97.54	1.19 (0.8 - 1.48)	1.56 (0.94 - 2.2)	0.81 (0.61 - 1.02)
Bahrain	Both	4.15 (2.03 - 5.67)	9.43 (6.79 - 12.29)	127.23	1.26 (0.58 - 1.71)	0.66 (0.47 - 0.86)	-3.32 (-3.83 - -2.81)
Bangladesh	Both	1339.48 (952.13 - 2035.59)	754.84 (518.74 - 1322.23)	-43.65	1.54 (1.13 - 2.43)	0.5 (0.34 - 0.86)	-4.39 (-4.5 - -4.28)
Barbados	Both	2.22 (1.78 - 2.77)	2.96 (2.39 - 3.82)	33.33	0.8 (0.65 - 1)	0.92 (0.71 - 1.21)	0.27 (0.06 - 0.49)
Belarus	Both	270.08 (172.32 - 343.73)	401.7 (274.98 - 527.45)	48.73	2.48 (1.57 - 3.22)	4.02 (2.67 - 5.38)	2.26 (1.92 - 2.6)
Belgium	Both	317.73 (246.79 - 392.28)	388.09 (304.58 - 588.09)	22.14	2.81 (2.19 - 3.56)	3.16 (2.4 - 4.93)	0.42 (0.25 - 0.59)
Belize	Both	1.01 (0.81 - 1.3)	2.44 (1.96 - 3.08)	141.58	0.71 (0.58 - 0.92)	0.67 (0.56 - 0.84)	-0.54 (-0.77 - -0.3)
Benin	Both	21.87 (16.15 - 27.54)	32.23 (25.12 - 41.2)	47.37	0.6 (0.46 - 0.73)	0.41 (0.32 - 0.51)	-1.48 (-1.59 - -1.37)
Bermuda	Both	0.51 (0.34 - 1.02)	1.78 (1.28 - 2.34)	249.02	0.81 (0.53 - 1.58)	2.63 (1.8 - 3.7)	4.25 (3.65 - 4.86)
Bhutan	Both	6.07 (4.32 - 9.19)	4.7 (2.59 - 8.97)	-22.57	1.34 (0.94 - 2.09)	0.53 (0.31 - 1.02)	-3.62 (-3.77 - -3.47)
Bolivia	Both	69.8 (46.19 - 85.57)	72.85 (52.03 - 99.18)	4.37	1.41 (0.96 - 1.68)	0.73 (0.53 - 0.98)	-2.47 (-2.51 - -2.44)
Bosnia and Herzegovina	Both	38.28 (28.92 - 44.66)	47.84 (28.28 - 62.37)	24.97	0.82 (0.62 - 0.96)	1.29 (0.78 - 1.75)	1.76 (1.53 - 2)
Botswana	Both	4.07 (2.94 - 5.84)	5.76 (4.54 - 8.02)	41.52	0.48 (0.36 - 0.68)	0.3 (0.25 - 0.41)	-1.47 (-1.7 - -1.24)
Brazil	Both	942.29 (798.68 - 1158.86)	1473.62 (1218.3 - 1803.51)	56.39	0.75 (0.63 - 0.91)	0.65 (0.54 - 0.8)	-0.38 (-0.47 - -0.3)
Brunei	Both	1.27 (0.8 - 2.32)	7.63 (5.27 - 9.86)	500.79	0.67 (0.44 - 1.23)	1.72 (1.19 - 2.21)	4.99 (4.43 - 5.55)
Bulgaria	Both	205.01 (171.83 - 266.02)	245.66 (196.86 - 308.25)	19.83	2.14 (1.76 - 2.68)	3.38 (2.57 - 4.55)	1.55 (1.29 - 1.81)
Burkina Faso	Both	50.11 (37.62 - 63.83)	68.09 (51.17 - 86.02)	35.88	0.68 (0.52 - 0.87)	0.45 (0.34 - 0.56)	-1.73 (-1.99 - -1.48)
Burundi	Both	68.73 (44.97 - 88.65)	76.26 (54.73 - 96.12)	10.96	1.56 (1.08 - 1.98)	0.93 (0.66 - 1.15)	-2.22 (-2.39 - -2.06)
Cambodia	Both	82.75 (59.22 - 101.97)	85.05 (64.71 - 114.11)	2.78	1.17 (0.85 - 1.51)	0.61 (0.47 - 0.81)	-2.57 (-2.66 - -2.48)
Cameroon	Both	44.47 (32.19 - 53.66)	81.68 (59.63 - 103.67)	83.67	0.57 (0.43 - 0.68)	0.43 (0.3 - 0.53)	-1.23 (-1.43 - -1.03)
Canada	Both	981.23 (737.87 - 1258.45)	1478.37 (1207.25 - 2069.62)	50.66	3.32 (2.46 - 4.36)	4.01 (3.17 - 5.57)	0.82 (0.1 - 1.55)
Cape Verde	Both	0.34 (0.24 - 0.41)	0.6 (0.4 - 0.72)	76.47	0.13 (0.08 - 0.16)	0.12 (0.08 - 0.14)	-0.51 (-0.63 - -0.39)
Central African Republic	Both	17.56 (11.56 - 24.88)	27.91 (21.23 - 37.93)	58.94	0.94 (0.67 - 1.29)	0.85 (0.65 - 1.15)	-0.57 (-0.67 - -0.47)
Chad	Both	25.7 (18.97 - 32.06)	48.28 (37.31 - 60.83)	87.86	0.55 (0.42 - 0.69)	0.49 (0.37 - 0.62)	-0.33 (-0.54 - -0.12)
Chile	Both	106.84 (86.9 - 130.12)	212.95 (170.18 - 285.15)	99.32	0.87 (0.72 - 1.06)	1.08 (0.85 - 1.47)	1.14 (0.79 - 1.5)
China	Both	9809.55 (5330.05 - 12229.82)	18267.64 (12908.15 - 20830.87)	86.22	0.89 (0.48 - 1.11)	1.19 (0.86 - 1.36)	0.88 (0.26 - 1.51)
Colombia	Both	245.89 (198.41 - 343.01)	412.13 (324.62 - 574.78)	67.61	0.91 (0.75 - 1.23)	0.79 (0.62 - 1.11)	-0.94 (-1.18 - -0.7)
Comoros	Both	5.64 (4.41 - 7.3)	6.04 (4.41 - 8.2)	7.09	1.49 (1.17 - 1.92)	0.95 (0.7 - 1.28)	-1.75 (-1.88 - -1.63)
Congo	Both	13.75 (10.4 - 18.97)	20.48 (14.73 - 28.43)	48.95	0.82 (0.65 - 1.09)	0.56 (0.4 - 0.78)	-1.72 (-1.94 - -1.5)
Costa Rica	Both	56.09 (26.38 - 74.34)	103.74 (64.84 - 136.39)	84.95	2.06 (1.06 - 2.62)	2.1 (1.34 - 2.76)	0.04 (-0.38 - 0.47)
Cote d'Ivoire	Both	56.95 (39.65 - 70.88)	98.24 (69.18 - 131.61)	72.50	0.63 (0.47 - 0.76)	0.51 (0.38 - 0.66)	-0.97 (-1.13 - -0.81)
Croatia	Both	171.66 (122.74 - 213.16)	146.4 (119.33 - 190.35)	-14.72	3.26 (2.3 - 4.19)	3.32 (2.64 - 4.43)	1.08 (0.65 - 1.5)
Cuba	Both	31.68 (19.22 - 75.44)	263.6 (71.07 - 377.36)	732.07	0.28 (0.17 - 0.65)	2.07 (0.56 - 3.04)	8 (5.97 - 10.06)
Cyprus	Both	11.83 (7.15 - 16.18)	38.81 (29.45 - 52.53)	228.06	1.44 (0.86 - 1.98)	2.75 (2.06 - 3.75)	3.03 (2.7 - 3.36)
Czech Republic	Both	442.96 (337.58 - 538.32)	443.34 (341.62 - 702.39)	0.09	4.15 (3.13 - 5.18)	4.38 (3.27 - 7.15)	-0.12 (-0.37 - 0.12)
Democratic Republic of the Congo	Both	174.31 (125.48 - 242.76)	288.09 (222.53 - 395.08)	65.27	0.67 (0.48 - 0.92)	0.54 (0.4 - 0.76)	-0.82 (-0.89 - -0.76)
Denmark	Both	143.9 (112.97 - 173.93)	207.42 (167.84 - 302.45)	44.14	2.53 (1.97 - 3.09)	3.3 (2.59 - 5.05)	1.01 (0.86 - 1.16)
Djibouti	Both	4.65 (2.84 - 6.51)	8.35 (5.54 - 12.62)	79.57	1.18 (0.81 - 1.63)	0.88 (0.59 - 1.27)	-1.36 (-1.58 - -1.13)
Dominica	Both	0.65 (0.51 - 0.92)	0.69 (0.56 - 0.92)	6.15	0.91 (0.72 - 1.27)	0.93 (0.74 - 1.24)	-0.03 (-0.14 - 0.08)
Dominican Republic	Both	9.21 (6.39 - 17.82)	38.97 (17.3 - 55.65)	323.13	0.16 (0.12 - 0.32)	0.38 (0.17 - 0.55)	2.25 (1.49 - 3.02)
Ecuador	Both	72.94 (61.5 - 102.57)	131.41 (101.64 - 205.63)	80.16	0.88 (0.77 - 1.22)	0.81 (0.64 - 1.23)	0.06 (-0.23 - 0.35)
Egypt	Both	997.45 (729.53 - 1259.02)	1648.24 (1090.98 - 2101.26)	65.25	2.24 (1.7 - 2.89)	1.89 (1.29 - 2.36)	-0.44 (-0.63 - -0.24)
El Salvador	Both	47.49 (38.07 - 58.62)	49.13 (35.61 - 83.96)	3.45	1.19 (0.92 - 1.49)	0.82 (0.6 - 1.36)	-1.68 (-1.89 - -1.48)
Equatorial Guinea	Both	2.92 (2.03 - 4.23)	2.92 (1.83 - 4.41)	0.00	0.98 (0.73 - 1.33)	0.32 (0.2 - 0.48)	-4.78 (-5.26 - -4.29)
Eritrea	Both	44.24 (22.83 - 62.52)	74.1 (49.3 - 108.64)	67.50	2.06 (1.27 - 2.75)	1.55 (1.05 - 2.24)	-1.47 (-1.74 - -1.2)
Estonia	Both	47.24 (31.53 - 60.04)	57.57 (41.17 - 86.74)	21.87	2.94 (1.97 - 3.81)	4.33 (3.07 - 6.93)	2.2 (1.66 - 2.74)
Ethiopia	Both	1149.2 (814.23 - 1496.47)	1126 (706.68 - 1866.91)	-2.02	2.94 (2.11 - 3.89)	1.45 (0.91 - 2.35)	-2.89 (-3.05 - -2.73)
Federated States of Micronesia	Both	0.37 (0.28 - 0.5)	0.34 (0.22 - 0.47)	-8.11	0.52 (0.38 - 0.71)	0.38 (0.26 - 0.52)	-1.16 (-1.24 - -1.07)
Fiji	Both	1.8 (1.36 - 2.19)	3.17 (1.74 - 4.34)	76.11	0.3 (0.23 - 0.37)	0.36 (0.21 - 0.49)	1.13 (0.88 - 1.39)
Finland	Both	140.07 (108.38 - 166.52)	227.45 (188.88 - 299.98)	62.38	2.49 (1.9 - 3)	3.9 (3.16 - 5.19)	2 (1.86 - 2.13)
France	Both	1436.95 (1130.86 - 1911.55)	2221.95 (1733.12 - 3195.6)	54.63	2.28 (1.79 - 3.06)	3.27 (2.47 - 4.72)	1.67 (1.33 - 2.02)
Gabon	Both	4.45 (3.47 - 5.89)	6.09 (4.53 - 8.49)	36.85	0.6 (0.47 - 0.8)	0.44 (0.33 - 0.62)	-1.04 (-1.28 - -0.81)
Georgia	Both	150.75 (101.86 - 184.89)	90.76 (72.97 - 118.86)	-39.79	2.58 (1.74 - 3.19)	2.18 (1.73 - 3)	-1.6 (-2.03 - -1.16)
Germany	Both	3428.54 (2286.73 - 4242.96)	3145.17 (2454.56 - 4965.78)	-8.27	3.78 (2.53 - 4.77)	3.4 (2.58 - 5.69)	-0.91 (-1.06 - -0.76)
Ghana	Both	18.47 (14.13 - 27.15)	31.33 (24.67 - 45.82)	69.63	0.17 (0.14 - 0.25)	0.14 (0.11 - 0.19)	-0.61 (-0.68 - -0.54)
Greece	Both	449.15 (358.56 - 700.63)	815.42 (676.38 - 1046.15)	81.55	3.76 (2.86 - 6.16)	6.68 (5.23 - 9.51)	2.38 (1.64 - 3.14)
Greenland	Both	0.45 (0.24 - 0.57)	0.28 (0.21 - 0.34)	-37.78	0.87 (0.44 - 1.12)	0.43 (0.34 - 0.54)	-3.1 (-3.34 - -2.87)
Grenada	Both	0.49 (0.4 - 0.63)	0.48 (0.38 - 0.7)	-2.04	0.66 (0.54 - 0.84)	0.38 (0.31 - 0.57)	-1.7 (-1.91 - -1.48)
Guam	Both	0.47 (0.33 - 0.71)	0.84 (0.64 - 1.16)	78.72	0.37 (0.27 - 0.55)	0.5 (0.38 - 0.69)	1.33 (0.85 - 1.82)
Guatemala	Both	51.55 (41.47 - 73.3)	66.77 (53.87 - 97.11)	29.52	0.84 (0.72 - 1.13)	0.48 (0.4 - 0.65)	-2.11 (-2.44 - -1.77)
Guinea	Both	51.08 (33.83 - 65.16)	65.66 (44.21 - 83.47)	28.54	0.92 (0.67 - 1.12)	0.72 (0.52 - 0.9)	-0.8 (-0.92 - -0.69)
Guinea-Bissau	Both	6.58 (4.46 - 8.59)	7.54 (5.82 - 10.02)	14.59	0.88 (0.63 - 1.13)	0.59 (0.47 - 0.74)	-1.48 (-1.52 - -1.44)
Guyana	Both	1.57 (0.85 - 4.02)	3.67 (2.7 - 4.41)	133.76	0.27 (0.15 - 0.67)	0.51 (0.38 - 0.62)	2.43 (1.28 - 3.59)
Haiti	Both	68.56 (53.17 - 99.33)	97.92 (62.64 - 144.72)	42.82	1.47 (1.1 - 2.24)	1.08 (0.68 - 1.62)	-1.12 (-1.19 - -1.05)
Honduras	Both	8.28 (5.58 - 10.9)	12.75 (8.59 - 16.94)	53.99	0.26 (0.17 - 0.33)	0.18 (0.11 - 0.25)	-1.37 (-1.42 - -1.33)
Hungary	Both	278.55 (195.78 - 339.3)	189.45 (147.59 - 267.62)	-31.99	2.5 (1.72 - 3.13)	1.99 (1.48 - 2.87)	-0.84 (-1.03 - -0.66)
Iceland	Both	8.81 (6.73 - 11.62)	10.31 (8.24 - 16.05)	17.03	3.3 (2.5 - 4.33)	2.86 (2.27 - 4.42)	-0.24 (-0.55 - 0.07)
India	Both	7321.82 (4696.73 - 8978.09)	6766.06 (5463.45 - 8438.35)	-7.59	0.98 (0.65 - 1.17)	0.52 (0.42 - 0.64)	-2.46 (-2.58 - -2.34)
Indonesia	Both	1108.51 (801.94 - 1375.09)	1209.81 (932.22 - 1552.04)	9.14	0.73 (0.54 - 0.93)	0.5 (0.38 - 0.63)	-1.41 (-1.48 - -1.34)
Iran	Both	274.07 (209.47 - 424.62)	1120.8 (674.43 - 1517.18)	308.95	0.59 (0.46 - 0.91)	1.34 (0.8 - 1.85)	4.51 (3.93 - 5.1)
Iraq	Both	109.95 (55.47 - 150.91)	105.39 (84.31 - 154.57)	-4.15	0.86 (0.46 - 1.14)	0.28 (0.23 - 0.42)	-4.37 (-5.32 - -3.41)
Ireland	Both	79.67 (62.29 - 98.03)	201.93 (158.21 - 257.55)	153.46	2.16 (1.69 - 2.67)	4.01 (3.1 - 5.15)	2.78 (2.43 - 3.13)
Israel	Both	78.01 (64.42 - 97.41)	211.03 (164.96 - 285.86)	170.52	1.61 (1.32 - 2)	2.38 (1.85 - 3.2)	1.02 (0.6 - 1.45)

Italy	Both	2133.49 (1636.27 - 2612.23)	2626.15 (2095.16 - 3544.32)	23.09	3.36 (2.53 - 4.19)	4.25 (3.27 - 5.72)	1.32 (1.08 - 1.56)
Jamaica	Both	9.27 (4.6 - 11.59)	14.76 (9.31 - 20.08)	59.22	0.44 (0.23 - 0.54)	0.5 (0.31 - 0.67)	0.54 (0.02 - 1.07)
Japan	Both	679.66 (585.57 - 863.38)	1420.23 (1030.95 - 1623.85)	108.96	0.48 (0.41 - 0.6)	0.92 (0.69 - 1.06)	3.14 (2.83 - 3.46)
Jordan	Both	8.58 (5.39 - 10.95)	25.42 (17.93 - 34.67)	196.27	0.32 (0.18 - 0.41)	0.26 (0.18 - 0.34)	-1.15 (-1.35 - -0.95)
Kazakhstan	Both	172.58 (129.36 - 233.96)	256.44 (204.73 - 329.1)	48.59	1.05 (0.79 - 1.42)	1.39 (1.1 - 1.8)	0.81 (0.65 - 0.97)
Kenya	Both	99.04 (78.14 - 132.19)	229.76 (169.25 - 270.3)	131.99	0.58 (0.47 - 0.78)	0.59 (0.43 - 0.7)	0.22 (-0.01 - 0.45)
Kiribati	Both	0.14 (0.12 - 0.2)	0.24 (0.18 - 0.31)	71.43	0.25 (0.21 - 0.36)	0.25 (0.2 - 0.32)	-0.23 (-0.39 - -0.06)
Kuwait	Both	11.54 (7.89 - 15.33)	29.41 (22.77 - 39.45)	154.85	0.69 (0.49 - 0.88)	0.65 (0.51 - 0.88)	0.67 (0.22 - 1.12)
Kyrgyzstan	Both	30.62 (18.8 - 38.02)	25.96 (20.56 - 35.74)	-15.22	0.77 (0.48 - 0.94)	0.44 (0.35 - 0.59)	-1.63 (-1.98 - -1.27)
Laos	Both	41.17 (27.81 - 50.62)	37.8 (26.57 - 52.45)	-8.19	1.28 (0.9 - 1.64)	0.65 (0.46 - 0.92)	-2.62 (-2.74 - -2.5)
Latvia	Both	66.28 (43.8 - 83.21)	79.29 (44.44 - 106.5)	19.63	2.36 (1.55 - 3.02)	3.98 (2.21 - 5.48)	2.59 (2.2 - 2.98)
Lebanon	Both	108.57 (79.57 - 144.57)	950.66 (627.29 - 1329.14)	775.62	3.15 (2.39 - 4.1)	10.4 (6.96 - 14.33)	5.36 (4.77 - 5.95)
Lesotho	Both	7.52 (6.09 - 10.19)	10.63 (7.88 - 13.95)	41.36	0.59 (0.48 - 0.81)	0.68 (0.5 - 0.88)	1.12 (0.62 - 1.61)
Liberia	Both	9.92 (7.08 - 12.34)	11.67 (9.06 - 14.98)	17.64	0.58 (0.44 - 0.71)	0.36 (0.28 - 0.46)	-2.05 (-2.36 - -1.73)
Libya	Both	81.06 (59.94 - 105.67)	301.8 (205.19 - 408.08)	272.32	2.46 (1.87 - 3.22)	4.27 (2.94 - 5.68)	2.84 (2.28 - 3.4)
Lithuania	Both	116.48 (83.6 - 145.89)	98.7 (76.56 - 138.86)	-15.26	3 (2.13 - 3.8)	3.36 (2.48 - 4.93)	0.43 (0.18 - 0.68)
Luxembourg	Both	11.42 (8.72 - 15.3)	21.98 (16.18 - 37.6)	92.47	2.62 (1.95 - 3.55)	3.36 (2.45 - 5.75)	0.41 (0.02 - 0.79)
Macedonia	Both	28.76 (17.24 - 37.33)	50.06 (30.65 - 67.98)	74.06	1.39 (0.84 - 1.8)	2.19 (1.32 - 3.05)	1.37 (1.13 - 1.61)
Madagascar	Both	147.93 (114.74 - 186.88)	178.56 (131.83 - 230.36)	20.71	1.39 (1.1 - 1.73)	0.88 (0.65 - 1.11)	-1.82 (-2.07 - -1.58)
Malawi	Both	57.39 (20.52 - 84.73)	75.73 (57.14 - 98.98)	31.96	0.72 (0.3 - 1)	0.54 (0.42 - 0.68)	-1.65 (-2 - -1.3)
Malaysia	Both	94.07 (53.77 - 126.44)	246.97 (118.82 - 375.36)	162.54	0.67 (0.4 - 0.88)	0.77 (0.38 - 1.17)	0.27 (-0.2 - 0.73)
Maldives	Both	0.52 (0.38 - 0.68)	1.07 (0.78 - 1.54)	105.77	0.37 (0.26 - 0.45)	0.24 (0.18 - 0.33)	-1.71 (-2.14 - -1.27)
Mali	Both	93.4 (59.15 - 119.48)	111.27 (79.06 - 146.38)	19.13	1.22 (0.88 - 1.47)	0.66 (0.51 - 0.84)	-2.35 (-2.58 - -2.12)
Malta	Both	9.11 (7.63 - 12.09)	21.35 (16.97 - 30.22)	134.36	2.33 (1.92 - 3.08)	4.45 (3.41 - 6.47)	2.66 (2.44 - 2.88)
Marshall Islands	Both	0.15 (0.11 - 0.18)	0.21 (0.13 - 0.27)	40.00	0.51 (0.38 - 0.61)	0.45 (0.29 - 0.57)	-0.33 (-0.48 - -0.18)
Mauritania	Both	8.54 (6.46 - 10.49)	9.83 (7.45 - 13.74)	15.11	0.55 (0.42 - 0.67)	0.34 (0.26 - 0.46)	-1.83 (-1.92 - -1.74)
Mauritius	Both	5.55 (3.87 - 6.76)	11.56 (6.42 - 15.48)	108.29	0.53 (0.38 - 0.63)	0.86 (0.47 - 1.18)	1.05 (0.49 - 1.61)
Mexico	Both	693.15 (550.49 - 785.08)	1180.99 (1012.75 - 1497.74)	70.38	1.06 (0.85 - 1.2)	0.95 (0.82 - 1.21)	-0.47 (-0.7 - -0.24)
Moldova	Both	105.27 (74.18 - 139.81)	89.46 (70.37 - 121.31)	-15.02	2.34 (1.64 - 3.11)	2.23 (1.67 - 3.09)	-0.52 (-0.73 - -0.31)
Mongolia	Both	4.41 (3.48 - 6.34)	7.07 (5.75 - 9.15)	60.32	0.31 (0.25 - 0.41)	0.23 (0.19 - 0.29)	-1.56 (-1.84 - -1.27)
Montenegro	Both	23.63 (18.74 - 30.74)	29.22 (22.1 - 41.72)	23.66	3.66 (2.89 - 4.76)	4.71 (3.43 - 6.92)	0.83 (0.69 - 0.97)
Morocco	Both	454.14 (312.31 - 553.92)	651.82 (438.13 - 849.01)	43.53	2.06 (1.52 - 2.42)	1.83 (1.25 - 2.37)	-0.46 (-0.53 - -0.38)
Mozambique	Both	214.77 (165.81 - 280.24)	274.8 (196.7 - 348.33)	27.95	1.78 (1.43 - 2.3)	1.18 (0.82 - 1.52)	-1.5 (-1.74 - -1.26)
Myanmar	Both	468.54 (321.3 - 587.9)	351.28 (268.98 - 452.17)	-25.03	1.41 (1 - 1.8)	0.69 (0.53 - 0.89)	-2.79 (-3 - -2.58)
Namibia	Both	10.19 (7.27 - 12.31)	12.04 (7.96 - 15.76)	18.16	0.99 (0.76 - 1.2)	0.62 (0.44 - 0.78)	-2.05 (-2.52 - -1.57)
Nepal	Both	188.78 (131.64 - 285.14)	145.76 (88.31 - 268.3)	-22.79	1.24 (0.89 - 1.86)	0.55 (0.34 - 1)	-3.08 (-3.39 - -2.77)
Netherlands	Both	571.67 (431.05 - 707.63)	730.66 (616.53 - 989.16)	27.81	3.42 (2.58 - 4.28)	3.92 (3.26 - 5.39)	0.67 (0.53 - 0.81)
New Zealand	Both	105.26 (60.93 - 139.95)	139.83 (86.11 - 170.11)	32.84	2.89 (1.7 - 3.87)	3.06 (1.87 - 3.75)	0.21 (0.08 - 0.34)
Nicaragua	Both	13.61 (9.33 - 16.44)	22.52 (14.39 - 29.46)	65.47	0.48 (0.35 - 0.58)	0.39 (0.25 - 0.5)	-0.72 (-0.85 - -0.58)
Niger	Both	46.93 (30.62 - 65.07)	57.36 (43.95 - 74.98)	22.22	0.71 (0.53 - 0.89)	0.42 (0.32 - 0.53)	-2.33 (-2.52 - -2.15)
Nigeria	Both	2366.79 (1462.2 - 3402.97)	3696.29 (2464.43 - 5237.37)	56.17	3.03 (2.02 - 4.2)	2.16 (1.5 - 3.08)	-1.57 (-1.71 - -1.43)
North Korea	Both	94.51 (60.01 - 129.39)	137.56 (93.4 - 174.28)	45.55	0.48 (0.31 - 0.65)	0.49 (0.33 - 0.63)	-0.05 (-0.12 - 0.01)
Northern Mariana Islands	Both	0.09 (0.07 - 0.14)	0.16 (0.12 - 0.21)	77.78	0.23 (0.17 - 0.32)	0.36 (0.28 - 0.48)	1.95 (1.53 - 2.37)
Norway	Both	131.94 (109.08 - 186.69)	175.86 (148.33 - 259.35)	33.29	2.83 (2.28 - 4.02)	3.09 (2.56 - 4.55)	0.51 (0.13 - 0.88)
Oman	Both	18.64 (11.03 - 25.78)	77.57 (44.61 - 113.98)	316.15	1.29 (0.79 - 1.71)	1.61 (0.91 - 2.3)	0.86 (0.33 - 1.4)
Pakistan	Both	2152.08 (1610.51 - 2749.8)	3838.94 (2910.94 - 5059.44)	78.38	2.29 (1.73 - 3.02)	1.96 (1.5 - 2.57)	-0.76 (-0.95 - -0.56)
Palestine	Both	8.66 (4.86 - 17.12)	24.53 (18.91 - 42.99)	183.26	0.61 (0.37 - 1.17)	0.63 (0.5 - 1.01)	0.06 (-0.24 - 0.35)
Panama	Both	7.58 (6.19 - 9.27)	23.76 (14.91 - 30.97)	213.46	0.37 (0.31 - 0.46)	0.61 (0.38 - 0.8)	2.07 (1.68 - 2.47)
Papua New Guinea	Both	22.22 (16.99 - 28.1)	43.04 (30.1 - 54.65)	93.70	0.72 (0.56 - 0.91)	0.58 (0.41 - 0.75)	-0.5 (-0.63 - -0.37)
Paraguay	Both	18.03 (13.84 - 24.9)	36.09 (25.93 - 45.97)	100.17	0.57 (0.45 - 0.77)	0.56 (0.4 - 0.71)	0.11 (-0.02 - 0.23)
Peru	Both	84.38 (69.8 - 115.14)	118.44 (88.32 - 150.48)	40.37	0.49 (0.39 - 0.65)	0.37 (0.27 - 0.47)	-0.98 (-1.1 - -0.86)
Philippines	Both	206.79 (172.2 - 307.5)	256.92 (170.96 - 535.19)	24.24	0.41 (0.35 - 0.58)	0.28 (0.19 - 0.55)	-1.54 (-1.76 - -1.31)
Poland	Both	939.68 (697.64 - 1081.23)	1101.31 (890.62 - 1439.45)	17.20	2.29 (1.7 - 2.67)	2.86 (2.27 - 3.84)	0.7 (0.49 - 0.91)
Portugal	Both	175.35 (143.05 - 259.92)	276.07 (214.81 - 404.72)	57.44	1.6 (1.29 - 2.39)	2.41 (1.82 - 3.53)	1.14 (0.67 - 1.61)
Puerto Rico	Both	52.64 (37.77 - 65.12)	98.52 (68.37 - 127.85)	87.16	1.43 (1.03 - 1.76)	2.6 (1.81 - 3.47)	2.18 (2.05 - 2.31)
Qatar	Both	0.98 (0.67 - 1.72)	21.21 (11.09 - 43.79)	2064.29	0.32 (0.24 - 0.51)	0.72 (0.4 - 1.4)	3.7 (3.37 - 4.03)
Romania	Both	409.62 (270.96 - 501.14)	425.89 (313.62 - 537.92)	3.97	1.69 (1.11 - 2.11)	2.31 (1.64 - 3.09)	1.01 (0.79 - 1.23)
Russian Federation	Both	5900.92 (3456.97 - 7697.19)	5799.84 (5045.62 - 7295.2)	-1.71	3.87 (2.23 - 5.15)	3.9 (3.31 - 5.01)	0.21 (-0.1 - 0.51)
Rwanda	Both	89.74 (61.18 - 114.37)	78.69 (57.71 - 105.82)	-12.31	1.53 (1.1 - 1.92)	0.77 (0.58 - 1.01)	-3.32 (-3.7 - -2.94)
Saint Lucia	Both	0.91 (0.77 - 1.3)	1.65 (1.27 - 2.17)	81.32	0.81 (0.69 - 1.17)	0.85 (0.65 - 1.11)	0.2 (-0.04 - 0.45)
Saint Vincent and the Grenadines	Both	0.37 (0.19 - 1.01)	1.06 (0.84 - 1.28)	186.49	0.41 (0.21 - 1.09)	0.86 (0.69 - 1.05)	1.83 (0.99 - 2.67)
Samoa	Both	0.87 (0.6 - 1.08)	0.86 (0.57 - 1.09)	-1.15	0.69 (0.5 - 0.84)	0.51 (0.35 - 0.64)	-1.21 (-1.31 - -1.11)
Sao Tome and Principe	Both	0.1 (0.07 - 0.17)	0.13 (0.09 - 0.22)	30.00	0.1 (0.08 - 0.17)	0.09 (0.06 - 0.13)	-0.59 (-0.65 - -0.52)
Saudi Arabia	Both	119.95 (39.52 - 186.77)	777.03 (197.25 - 1344.08)	547.79	0.97 (0.35 - 1.53)	2.08 (0.53 - 3.52)	3.32 (2.43 - 4.21)
Senegal	Both	32.97 (24.21 - 39.72)	46.27 (35.76 - 63.98)	40.34	0.57 (0.44 - 0.68)	0.44 (0.34 - 0.58)	-0.92 (-0.97 - -0.88)
Serbia	Both	240.78 (159.95 - 315.63)	270.47 (158.96 - 365.05)	12.33	2.43 (1.6 - 3.23)	2.85 (1.62 - 4.02)	1.08 (0.83 - 1.34)
Seychelles	Both	0.49 (0.37 - 0.8)	1.01 (0.79 - 1.38)	106.12	0.76 (0.58 - 1.2)	0.96 (0.75 - 1.3)	0.57 (0.34 - 0.79)
Sierra Leone	Both	18.53 (12.68 - 24.22)	23.24 (17.46 - 29.38)	25.42	0.56 (0.42 - 0.71)	0.41 (0.3 - 0.5)	-1.21 (-1.27 - -1.14)
Singapore	Both	13.3 (10.29 - 16.58)	62.73 (40.88 - 79.86)	371.65	0.42 (0.34 - 0.53)	0.99 (0.64 - 1.26)	4.45 (3.95 - 4.96)
Slovakia	Both	141.35 (107.72 - 174.71)	228.14 (177.04 - 297.48)	61.40	2.59 (1.98 - 3.22)	4.17 (3.19 - 5.54)	2.04 (1.73 - 2.35)
Slovenia	Both	58.85 (44.6 - 74.87)	64.78 (52.93 - 86.2)	10.08	2.82 (2.12 - 3.66)	3.37 (2.67 - 4.51)	0.73 (0.55 - 0.9)
Solomon Islands	Both	1.21 (0.89 - 1.58)	2.09 (1.46 - 2.88)	72.73	0.54 (0.41 - 0.73)	0.44 (0.3 - 0.61)	-0.54 (-0.67 - -0.41)
Somalia	Both	80.56 (27.41 - 131.1)	189.85 (112.36 - 301.91)	135.66	1.47 (0.66 - 2.3)	1.48 (0.92 - 2.25)	-0.37 (-0.62 - -0.13)
South Africa	Both	116.62 (90.35 - 138.13)	168.3 (123.37 - 201.9)	44.31	0.39 (0.3 - 0.47)	0.32 (0.23 - 0.38)	-0.96 (-1.6 - -0.32)
South Korea	Both	64.32 (49.27 - 117.98)	421.6 (244.13 - 555.93)	555.47	0.16 (0.13 - 0.29)	0.7 (0.42 - 0.93)	6.88 (6.31 - 7.45)
South Sudan	Both	51 (19.28 - 84.8)	81.75 (56.87 - 110.85)	60.29	1.13 (0.56 - 1.77)	1.09 (0.76 - 1.45)	-0.4 (-0.62 - -0.17)
Spain	Both	1145.71 (836.86 - 1412.16)	1683.58 (1286.29 - 2225.27)	46.95	2.66 (1.92 - 3.29)	3.39 (2.56 - 4.6)	1.09 (0.83 - 1.35)
Sri Lanka	Both	137.88 (115.55 - 184.01)	214.63 (160.66 - 309.27)	55.66	0.98 (0.81 - 1.29)	0.94 (0.7 - 1.38)	-0.68 (-1.01 - -0.35)
Sudan	Both	160.74 (106.52 - 243.23)	220.38 (167.24 - 297.67)	37.10	1.09 (0.76 - 1.63)	0.73 (0.54 - 0.99)	-1.43 (-1.47 - -1.39)
Suriname	Both	2.53 (2.11 - 3.57)	4.55 (3.56 - 5.65)	79.84	0.74 (0.62 - 1.05)	0.77 (0.61 - 0.96)	0.26 (-0.03 - 0.56)
Swaziland	Both	2.89 (2.3 - 3.9)	4.65 (3.43 - 6.08)	60.90	0.61 (0.49 - 0.83)	0.56 (0.41 - 0.73)	0.41 (-0.22 - 1.04)
Sweden	Both	184.87 (152.59 - 243.52)	253.11 (213.1 - 326.58)	36.91	1.88 (1.53 - 2.58)	2.31 (1.87 - 3.02)	0.56 (0.35 - 0.78)
Switzerland	Both	250.29 (184.71 - 324.61)	254.85 (201.76 - 381.68)	1.82	3.34 (2.38 - 4.5)	2.69 (2.07 - 4.22)	-0.9 (-1.12 - -0.67)
Syria	Both	11.94 (8.7 - 14.85)	26.31 (15.96 - 35.52)	120.35	0.15 (0.1 - 0.18)	0.17 (0.1 - 0.23)	0.39 (0.07 - 0.71)
Tajikistan	Both	11.52 (9.38 - 15.11)	21.94 (17.93 - 29.38)	90.45	0.31 (0.23 - 0.39)	0.3 (0.23 - 0.35)	-0.46 (-0.64 - -0.27)
Tanzania	Both	221.95 (104.92 - 303.45)	383.69 (275.25 - 527.74)	72.87	1.07 (0.61 - 1.47)	0.86 (0.63 - 1.14)	-1.07 (-1.23 - -0.92)
Thailand	Both	287.37 (225.93 - 426.8)	418.42 (285.23 - 762.25)	45.60	0.57 (0.46 - 0.83)	0.55 (0.35 - 1.06)	-0.78 (-1.05 - -0.51)
The Bahamas	Both	1.69 (1.4 - 2.05)	3.08 (2.4 - 4.09)	82.25	0.72 (0.59 - 0.86)	0.77 (0.6 - 1.03)	0.23 (0.09 - 0.36)

The Gambia	Both	4.51 (3.18 - 5.93)	7.68 (5.77 - 10.88)	70.29	0.62 (0.46 - 0.81)	0.48 (0.38 - 0.68)	-0.71 (-0.83 - -0.6)
Timor-Leste	Both	5.4 (3.74 - 7.41)	6.28 (3.5 - 9.69)	16.30	0.96 (0.64 - 1.42)	0.61 (0.35 - 0.95)	-1.88 (-2.01 - -1.74)
Togo	Both	14.78 (11.17 - 18.14)	21.37 (16.36 - 27.22)	44.59	0.55 (0.43 - 0.68)	0.39 (0.3 - 0.5)	-1.4 (-1.57 - -1.24)
Tonga	Both	0.19 (0.14 - 0.25)	0.24 (0.17 - 0.33)	26.32	0.26 (0.2 - 0.35)	0.27 (0.19 - 0.36)	0.19 (0.12 - 0.25)
Trinidad and Tobago	Both	4.12 (3.34 - 5.14)	9.28 (5.07 - 12.57)	125.24	0.38 (0.31 - 0.47)	0.61 (0.33 - 0.83)	2.69 (2.14 - 3.23)
Tunisia	Both	111.43 (87.29 - 142.88)	230.49 (158.69 - 309.09)	106.85	1.5 (1.21 - 1.97)	1.96 (1.34 - 2.64)	1.03 (0.91 - 1.14)
Turkey	Both	454.29 (326.19 - 750.7)	897.71 (664.75 - 1311.59)	97.61	0.91 (0.65 - 1.5)	1.08 (0.79 - 1.6)	0.81 (0.3 - 1.32)
Turkmenistan	Both	27.41 (18.92 - 32.89)	57.6 (35.44 - 75.14)	110.14	0.83 (0.61 - 0.96)	1.12 (0.71 - 1.45)	1.32 (1 - 1.63)
Uganda	Both	153.96 (115.35 - 202.26)	289.38 (203.69 - 398.27)	87.96	1.11 (0.85 - 1.5)	0.98 (0.71 - 1.29)	-0.7 (-0.86 - -0.53)
Ukraine	Both	1082.73 (770.74 - 1424.38)	1968.26 (1478.58 - 2891.45)	81.79	1.98 (1.39 - 2.62)	4.47 (3.22 - 6.67)	2.61 (1.97 - 3.25)
United Arab Emirates	Both	11.63 (7.62 - 19.08)	105.17 (71.48 - 157.57)	804.30	0.81 (0.59 - 1.25)	1.15 (0.78 - 1.74)	1.61 (1.45 - 1.78)
United Kingdom	Both	2483.09 (1783.25 - 2773.54)	3043.87 (2542.39 - 3680.15)	22.58	3.86 (2.71 - 4.35)	4.11 (3.44 - 5.02)	0.16 (0.06 - 0.25)
United States	Both	9417.93 (6919.53 - 10439.86)	10966.1 (9181.63 - 16238.65)	16.44	3.38 (2.48 - 3.75)	3.15 (2.59 - 4.79)	-0.84 (-1.13 - -0.55)
Uruguay	Both	40.78 (29.69 - 47.07)	46.89 (34.33 - 58.29)	14.98	1.18 (0.86 - 1.36)	1.21 (0.88 - 1.54)	0.21 (0.04 - 0.39)
Uzbekistan	Both	153.4 (119.23 - 207.85)	330.65 (258.1 - 440.34)	115.55	0.83 (0.62 - 1.2)	1.03 (0.82 - 1.36)	0.98 (0.8 - 1.17)
Vanuatu	Both	0.76 (0.4 - 1.33)	1.61 (0.75 - 2.96)	111.84	0.72 (0.38 - 1.28)	0.71 (0.33 - 1.32)	0.05 (-0.08 - 0.17)
Venezuela	Both	127.49 (97.5 - 156.67)	354.52 (238.35 - 463.71)	178.08	0.88 (0.67 - 1.06)	1.13 (0.77 - 1.46)	1.25 (1.05 - 1.46)
Vietnam	Both	383.68 (285.58 - 475.97)	650.28 (397.47 - 865.9)	69.48	0.7 (0.54 - 0.91)	0.66 (0.4 - 0.89)	-0.11 (-0.33 - 0.11)
Virgin Islands, U.S.	Both	0.28 (0.21 - 0.47)	0.76 (0.57 - 0.94)	171.43	0.28 (0.22 - 0.48)	0.62 (0.46 - 0.79)	4.02 (3.42 - 4.64)
Yemen	Both	101.83 (44.57 - 163.95)	185.89 (133.16 - 265.24)	82.55	1.16 (0.59 - 1.9)	0.85 (0.6 - 1.23)	-1.26 (-1.35 - -1.17)
Zambia	Both	92.2 (55.27 - 118.53)	137.41 (97.94 - 186.1)	49.03	1.48 (1.03 - 1.87)	1.02 (0.74 - 1.34)	-1.86 (-2.18 - -1.54)
Zimbabwe	Both	33.57 (21.62 - 41.78)	79.64 (43.65 - 108.57)	137.24	0.47 (0.36 - 0.59)	0.7 (0.43 - 0.9)	2.51 (1.49 - 3.55)
Afghanistan	Female	55.58 (21.22 - 94.3)	160.11 (102.97 - 236.57)	188.07	1.48 (0.62 - 2.53)	1.4 (0.92 - 1.99)	-0.2 (-0.46 - 0.06)
Albania	Female	17 (12.93 - 24.42)	26.47 (16.23 - 44.56)	55.71	1.09 (0.85 - 1.55)	1.92 (1.17 - 3.22)	2.58 (2.34 - 2.81)
Algeria	Female	153.6 (97.02 - 199.18)	335.21 (209.38 - 466.31)	118.24	1.45 (1.01 - 1.84)	1.65 (1.05 - 2.28)	0.57 (0.49 - 0.64)
American Samoa	Female	0.04 (0.03 - 0.06)	0.07 (0.05 - 0.11)	75.00	0.22 (0.16 - 0.35)	0.29 (0.2 - 0.45)	1.32 (0.95 - 1.69)
Andorra	Female	0.69 (0.46 - 1.02)	1.29 (0.95 - 1.9)	86.96	2.32 (1.52 - 3.47)	3.29 (2.38 - 4.86)	1.06 (0.65 - 1.47)
Angola	Female	19.51 (11.21 - 33.21)	32.98 (23.4 - 48.1)	69.04	0.54 (0.35 - 0.85)	0.33 (0.23 - 0.49)	-1.87 (-2.12 - -1.62)
Antigua and Barbuda	Female	0.03 (0.02 - 0.04)	0.07 (0.05 - 0.09)	133.33	0.08 (0.07 - 0.13)	0.15 (0.1 - 0.2)	2.43 (2.18 - 2.68)
Argentina	Female	117.38 (98.15 - 164.63)	154.59 (112.46 - 267.57)	31.70	0.68 (0.57 - 0.96)	0.62 (0.44 - 1.1)	-0.66 (-0.83 - -0.49)
Armenia	Female	2.78 (1.86 - 5.91)	8.73 (5.09 - 12.1)	214.03	0.16 (0.11 - 0.34)	0.54 (0.29 - 0.8)	5.23 (4.09 - 6.39)
Australia	Female	181.02 (136.55 - 227.51)	357.56 (276.96 - 456.19)	97.53	1.99 (1.49 - 2.56)	2.86 (2.19 - 3.68)	1.36 (1.14 - 1.58)
Austria	Female	133.85 (70.53 - 173.32)	90.3 (66.94 - 173.72)	-32.54	2.83 (1.56 - 3.75)	1.94 (1.35 - 3.94)	-2.08 (-2.45 - -1.7)
Azerbaijan	Female	33.12 (15.54 - 49.23)	71.44 (35.55 - 114.95)	115.70	0.86 (0.43 - 1.25)	1.27 (0.64 - 2.03)	1 (0.75 - 1.25)
Bahrain	Female	0.91 (0.36 - 1.38)	2.07 (1.32 - 2.86)	127.47	0.56 (0.23 - 0.84)	0.38 (0.25 - 0.52)	-1.92 (-2.37 - -1.48)
Bangladesh	Female	387.62 (281.02 - 538.86)	286.57 (191.66 - 485.42)	-26.07	0.87 (0.66 - 1.21)	0.36 (0.24 - 0.6)	-3.46 (-3.64 - -3.28)
Barbados	Female	0.86 (0.66 - 1.18)	1.3 (0.99 - 1.84)	51.16	0.58 (0.44 - 0.8)	0.81 (0.59 - 1.2)	1.19 (0.97 - 1.41)
Belarus	Female	124.36 (78.33 - 167.86)	208.1 (132.87 - 275.67)	67.34	2.2 (1.37 - 3.15)	4.21 (2.56 - 5.81)	2.94 (2.5 - 3.39)
Belgium	Female	118.26 (80.67 - 151.23)	143.54 (112.53 - 195.53)	21.38	2.07 (1.45 - 2.75)	2.4 (1.83 - 3.38)	0.68 (0.5 - 0.85)
Belize	Female	0.33 (0.26 - 0.44)	0.99 (0.79 - 1.36)	200.00	0.47 (0.38 - 0.63)	0.53 (0.43 - 0.73)	0.28 (0.04 - 0.53)
Benin	Female	6.32 (4.53 - 8.41)	10.97 (7.56 - 16.08)	73.58	0.34 (0.25 - 0.45)	0.26 (0.18 - 0.36)	-1.24 (-1.41 - -1.08)
Bermuda	Female	0.2 (0.11 - 0.54)	0.99 (0.57 - 1.35)	395.00	0.6 (0.34 - 1.6)	2.86 (1.59 - 4.29)	5.07 (4.06 - 6.08)
Bhutan	Female	1.8 (1.26 - 2.47)	1.54 (0.79 - 3)	-14.44	0.8 (0.57 - 1.11)	0.34 (0.19 - 0.65)	-3.42 (-3.66 - -3.18)
Bolivia	Female	22.3 (12.21 - 31.98)	26.67 (17.47 - 36.13)	19.60	0.89 (0.52 - 1.17)	0.52 (0.34 - 0.7)	-2.13 (-2.24 - -2.02)
Bosnia and Herzegovina	Female	16.81 (11.94 - 20.98)	25.33 (13.26 - 35.81)	50.68	0.71 (0.5 - 0.88)	1.4 (0.73 - 2.08)	2.6 (2.37 - 2.83)
Botswana	Female	1.3 (0.73 - 2.23)	2.51 (1.84 - 3.66)	93.08	0.27 (0.16 - 0.45)	0.23 (0.18 - 0.33)	0.33 (-0.16 - 0.82)
Brazil	Female	310.14 (266.57 - 381.93)	612.71 (468.27 - 696.81)	97.56	0.47 (0.4 - 0.56)	0.53 (0.4 - 0.61)	0.48 (0.39 - 0.56)
Brunei	Female	0.48 (0.24 - 1.02)	3.94 (2.42 - 5.75)	720.83	0.53 (0.27 - 1.16)	1.82 (1.14 - 2.65)	6.51 (5.83 - 7.2)
Bulgaria	Female	70.06 (58.06 - 89.59)	92.32 (68.58 - 123.26)	31.77	1.46 (1.18 - 1.88)	2.62 (1.82 - 3.72)	1.78 (1.38 - 2.17)
Burkina Faso	Female	17.01 (11.88 - 22.23)	21.78 (15.92 - 29.81)	28.04	0.46 (0.33 - 0.6)	0.28 (0.2 - 0.37)	-2.22 (-2.47 - -1.96)
Burundi	Female	23.01 (13.63 - 32.24)	23.68 (15.6 - 31.67)	2.91	1.01 (0.6 - 1.38)	0.57 (0.37 - 0.75)	-2.52 (-2.71 - -2.32)
Cambodia	Female	28.1 (17.39 - 36.74)	30.9 (21.1 - 42.39)	9.96	0.7 (0.46 - 0.89)	0.4 (0.27 - 0.55)	-2.33 (-2.46 - -2.2)
Cameroon	Female	13.85 (9.58 - 18.18)	26.04 (18.33 - 36.47)	88.01	0.35 (0.25 - 0.46)	0.26 (0.18 - 0.35)	-1.36 (-1.57 - -1.15)
Canada	Female	377 (232.75 - 507.55)	574.7 (449.44 - 724.95)	52.44	2.53 (1.51 - 3.51)	3.16 (2.39 - 4.03)	0.88 (0.73 - 1.03)
Cape Verde	Female	0.12 (0.09 - 0.16)	0.22 (0.16 - 0.28)	83.33	0.08 (0.06 - 0.1)	0.08 (0.06 - 0.1)	0.07 (-0.13 - 0.27)
Central African Republic	Female	5.79 (3.68 - 8.73)	8.45 (5.59 - 12.38)	45.94	0.6 (0.41 - 0.86)	0.51 (0.34 - 0.74)	-0.68 (-0.8 - -0.55)
Chad	Female	7.55 (5.11 - 10.44)	13.73 (9.66 - 19.13)	81.85	0.33 (0.23 - 0.45)	0.28 (0.2 - 0.38)	-0.52 (-0.7 - -0.34)
Chile	Female	39.95 (33.79 - 49.54)	93.25 (67.96 - 125.22)	133.42	0.62 (0.52 - 0.75)	0.94 (0.68 - 1.31)	1.85 (1.44 - 2.27)
China	Female	3879.15 (1750.27 - 5109.76)	6180.98 (4561.67 - 7675.14)	59.34	0.7 (0.32 - 0.92)	0.89 (0.66 - 1.1)	0.62 (0.09 - 1.15)
Colombia	Female	76.32 (64.87 - 108.73)	167.08 (127.93 - 233.5)	118.92	0.57 (0.49 - 0.83)	0.62 (0.47 - 0.89)	-0.18 (-0.43 - 0.07)
Comoros	Female	2.14 (1.46 - 2.91)	2.45 (1.62 - 3.67)	14.49	1.1 (0.76 - 1.48)	0.74 (0.49 - 1.09)	-1.53 (-1.61 - -1.46)
Congo	Female	4.66 (3.27 - 7.13)	8.14 (5.24 - 13.17)	74.68	0.54 (0.4 - 0.79)	0.43 (0.29 - 0.67)	-1.15 (-1.4 - -0.9)
Costa Rica	Female	21.95 (8.68 - 30.48)	47.66 (20.48 - 70.86)	117.13	1.55 (0.67 - 2.05)	1.85 (0.83 - 2.74)	0.89 (0.44 - 1.35)
Cote d'Ivoire	Female	17.49 (11.24 - 23.58)	31.86 (19.68 - 45.97)	82.16	0.39 (0.28 - 0.5)	0.33 (0.22 - 0.45)	-0.8 (-0.92 - -0.67)
Croatia	Female	80.8 (56.59 - 103.87)	73.69 (57.78 - 94.42)	-8.80	2.95 (2.05 - 3.91)	3.21 (2.43 - 4.15)	1.58 (1.09 - 2.08)
Cuba	Female	9.93 (5.71 - 29.15)	119.85 (26.34 - 175.19)	1106.95	0.17 (0.1 - 0.49)	1.9 (0.43 - 2.88)	9.89 (7.56 - 12.28)
Cyprus	Female	5.95 (2.86 - 9.28)	17.89 (12.11 - 25.78)	200.67	1.44 (0.69 - 2.3)	2.68 (1.69 - 3.99)	3.07 (2.74 - 3.41)
Czech Republic	Female	215.86 (155.03 - 289.94)	215.46 (161.12 - 367.2)	-0.19	4.09 (2.82 - 5.74)	4.45 (3.29 - 7.66)	-0.43 (-0.8 - -0.05)
Democratic Republic of the Congo	Female	60.59 (40.43 - 89.54)	95.98 (70.01 - 140.46)	58.41	0.45 (0.32 - 0.65)	0.35 (0.26 - 0.51)	-1.04 (-1.13 - -0.95)
Denmark	Female	55.62 (38.61 - 68.08)	78.66 (56.05 - 100.67)	41.42	1.94 (1.33 - 2.43)	2.62 (1.88 - 3.49)	1.04 (0.83 - 1.24)
Djibouti	Female	1.51 (0.94 - 2.2)	2.77 (1.58 - 4.54)	83.44	0.84 (0.55 - 1.19)	0.6 (0.34 - 0.97)	-1.41 (-1.57 - -1.26)
Dominica	Female	0.14 (0.11 - 0.18)	0.17 (0.13 - 0.24)	21.43	0.37 (0.3 - 0.48)	0.47 (0.35 - 0.65)	0.83 (0.7 - 0.95)
Dominican Republic	Female	2.76 (1.59 - 5.68)	16.7 (6.72 - 27.16)	505.07	0.09 (0.06 - 0.19)	0.32 (0.13 - 0.52)	3.79 (2.99 - 4.6)
Ecuador	Female	30.65 (24.96 - 43.37)	55.11 (41.22 - 86.94)	79.80	0.73 (0.61 - 0.99)	0.67 (0.51 - 1.02)	0.03 (-0.38 - 0.44)
Egypt	Female	318.78 (220.12 - 411.14)	579.01 (337.27 - 831.14)	81.63	1.4 (1.01 - 1.8)	1.3 (0.8 - 1.82)	-0.28 (-0.54 - -0.02)
El Salvador	Female	15.18 (11.57 - 19.76)	18.5 (12.6 - 29.8)	21.87	0.75 (0.54 - 0.96)	0.56 (0.38 - 0.9)	-1.45 (-1.68 - -1.23)
Equatorial Guinea	Female	0.89 (0.58 - 1.46)	1.12 (0.61 - 1.81)	25.84	0.57 (0.4 - 0.87)	0.24 (0.14 - 0.39)	-3.72 (-4.13 - -3.31)
Eritrea	Female	10.9 (6.28 - 15.44)	20.67 (12.01 - 31.86)	89.63	1.03 (0.66 - 1.4)	0.86 (0.51 - 1.31)	-1.07 (-1.23 - -0.92)
Estonia	Female	20.18 (13.15 - 27.06)	25.9 (18.09 - 35.01)	28.34	2.42 (1.57 - 3.4)	3.89 (2.7 - 5.47)	2.79 (2.23 - 3.36)
Ethiopia	Female	337.96 (231.05 - 463.68)	371.34 (197.93 - 636.82)	9.88	1.73 (1.18 - 2.42)	0.9 (0.48 - 1.5)	-2.73 (-2.87 - -2.58)
Federated States of Micronesia	Female	0.12 (0.08 - 0.16)	0.11 (0.07 - 0.16)	-8.33	0.34 (0.23 - 0.48)	0.26 (0.16 - 0.35)	-1.2 (-1.33 - -1.07)
Fiji	Female	0.54 (0.38 - 0.84)	0.84 (0.61 - 1.12)	55.56	0.19 (0.14 - 0.29)	0.2 (0.14 - 0.26)	0.19 (-0.01 - 0.38)
Finland	Female	61.47 (39.49 - 76.41)	90.32 (66.98 - 112.16)	46.93	2.12 (1.38 - 2.76)	3.25 (2.34 - 4.17)	1.72 (1.5 - 1.94)
France	Female	504.7 (394.83 - 666.21)	839.86 (639.42 - 1224.08)	66.41	1.57 (1.18 - 2.12)	2.53 (1.85 - 3.78)	2.11 (1.68 - 2.55)
Gabon	Female	1.34 (1 - 1.96)	1.88 (1.26 - 2.81)	40.30	0.35 (0.26 - 0.51)	0.26 (0.17 - 0.4)	-1.14 (-1.45 - -0.83)
Georgia	Female	55.97 (27.37 - 75.14)	31.74 (21.64 - 39.97)	-43.29	1.8 (0.89 - 2.46)	1.49 (1.02 - 1.98)	-1.96 (-2.77 - -1.16)
Germany	Female	1518.68 (847.43 - 1960.59)	1156.87 (893.77 - 1752.5)	-23.82	3.3 (1.87 - 4.38)	2.63 (1.96 - 4.35)	-1.51 (-1.71 - -1.31)
Ghana	Female	7.78 (5.6 - 11.24)	13.11 (9.72 - 19.44)	68.51	0.15 (0.11 - 0.2)	0.11 (0.08 - 0.15)	-1.22 (-1.34 - -1.11)

Greece	Female	168.56 (129.1 - 287.33)	358.16 (284.14 - 446.03)	112.48	2.77 (2 - 5.05)	5.91 (4.42 - 8.72)	3.07 (2.27 - 3.87)
Greenland	Female	0.08 (0.06 - 0.1)	0.07 (0.05 - 0.09)	-12.50	0.33 (0.21 - 0.42)	0.23 (0.17 - 0.33)	-1.61 (-1.93 - -1.28)
Grenada	Female	0.11 (0.09 - 0.16)	0.13 (0.1 - 0.21)	18.18	0.28 (0.21 - 0.39)	0.22 (0.16 - 0.34)	-0.66 (-0.83 - -0.49)
Guam	Female	0.07 (0.05 - 0.1)	0.15 (0.1 - 0.21)	114.29	0.11 (0.08 - 0.15)	0.19 (0.13 - 0.27)	1.62 (0.91 - 2.34)
Guatemala	Female	18.49 (12.81 - 30.03)	25.15 (18.64 - 42.12)	36.02	0.58 (0.45 - 0.96)	0.34 (0.26 - 0.54)	-1.84 (-2.22 - -1.46)
Guinea	Female	16.41 (10.15 - 22.15)	23.13 (13.95 - 34.29)	40.95	0.61 (0.42 - 0.78)	0.48 (0.31 - 0.65)	-0.88 (-0.95 - -0.81)
Guinea-Bissau	Female	1.92 (1.27 - 2.65)	2.48 (1.72 - 3.61)	29.17	0.5 (0.34 - 0.69)	0.36 (0.26 - 0.51)	-1.24 (-1.3 - -1.18)
Guyana	Female	0.48 (0.25 - 1.58)	1.48 (0.97 - 1.94)	208.33	0.15 (0.08 - 0.48)	0.4 (0.26 - 0.51)	3.45 (2.03 - 4.9)
Haiti	Female	19.71 (14.02 - 25.51)	31.98 (17.28 - 48.05)	62.25	0.82 (0.59 - 1.04)	0.65 (0.36 - 0.96)	-0.85 (-0.94 - -0.76)
Honduras	Female	0.8 (0.57 - 1)	1.99 (1.45 - 2.65)	148.75	0.05 (0.03 - 0.06)	0.05 (0.03 - 0.07)	0.24 (0.12 - 0.36)
Hungary	Female	128.35 (63.69 - 169.46)	84.45 (63.58 - 116.46)	-34.20	2.29 (1.09 - 3.17)	1.82 (1.28 - 2.61)	-0.88 (-1.04 - -0.71)
Iceland	Female	3.79 (2.8 - 5.05)	3.73 (2.89 - 6.57)	-1.58	2.84 (2.08 - 3.79)	2.16 (1.62 - 3.85)	-0.75 (-1.15 - -0.36)
India	Female	2394.85 (1298.75 - 3285.59)	2385.13 (1770.63 - 3147.37)	-0.41	0.64 (0.36 - 0.85)	0.36 (0.27 - 0.47)	-2.37 (-2.53 - -2.21)
Indonesia	Female	380 (231.03 - 454.31)	422.1 (292.32 - 525.44)	11.08	0.48 (0.31 - 0.58)	0.34 (0.24 - 0.42)	-1.4 (-1.46 - -1.35)
Iran	Female	106.59 (76.56 - 165.62)	478.68 (273.65 - 693.44)	349.09	0.46 (0.35 - 0.7)	1.16 (0.64 - 1.69)	4.94 (4.34 - 5.55)
Iraq	Female	41.2 (14.52 - 69.75)	39.68 (28.83 - 55.58)	-3.69	0.63 (0.23 - 1.03)	0.21 (0.16 - 0.29)	-4.37 (-5.43 - -3.3)
Ireland	Female	30.81 (23.9 - 41.37)	87.75 (64.35 - 114.13)	184.81	1.64 (1.25 - 2.23)	3.48 (2.55 - 4.62)	3.43 (3.04 - 3.82)
Israel	Female	38.47 (29.37 - 50)	103.36 (73.77 - 144.25)	168.68	1.54 (1.16 - 2.01)	2.34 (1.64 - 3.32)	1.18 (0.89 - 1.48)
Italy	Female	877.12 (606.53 - 1106.19)	1097.78 (840.79 - 1456.78)	25.16	2.72 (1.84 - 3.57)	3.72 (2.69 - 5.18)	1.43 (1.22 - 1.64)
Jamaica	Female	3.55 (1.65 - 4.56)	5.58 (2.84 - 8.24)	57.18	0.31 (0.15 - 0.39)	0.37 (0.19 - 0.54)	1.17 (0.44 - 1.91)
Japan	Female	203.28 (175.26 - 277.8)	448.72 (315 - 514.29)	120.74	0.29 (0.25 - 0.4)	0.64 (0.47 - 0.74)	3.58 (3.16 - 3.99)
Jordan	Female	2.97 (1.36 - 4.45)	8.97 (4.78 - 13.83)	202.02	0.23 (0.09 - 0.34)	0.19 (0.1 - 0.28)	-1.29 (-1.68 - -0.89)
Kazakhstan	Female	74.43 (52.82 - 102)	124.46 (91.17 - 166.66)	67.22	0.86 (0.62 - 1.18)	1.31 (0.96 - 1.77)	1.26 (1.04 - 1.48)
Kenya	Female	33.4 (23.27 - 45.14)	69.55 (46.39 - 87.36)	108.23	0.38 (0.27 - 0.52)	0.33 (0.22 - 0.41)	-0.69 (-0.81 - -0.57)
Kiribati	Female	0.04 (0.03 - 0.07)	0.07 (0.05 - 0.11)	75.00	0.16 (0.12 - 0.26)	0.16 (0.11 - 0.24)	-0.16 (-0.32 - 0.01)
Kuwait	Female	4.41 (2.99 - 5.91)	12.43 (8.78 - 16.92)	181.86	0.62 (0.43 - 0.79)	0.6 (0.43 - 0.83)	1.18 (0.35 - 2.01)
Kyrgyzstan	Female	12.74 (6.16 - 16.51)	9.78 (7.59 - 13.44)	-23.23	0.6 (0.3 - 0.77)	0.31 (0.25 - 0.42)	-1.62 (-2.14 - -1.1)
Laos	Female	12.16 (6.61 - 17.48)	13.42 (8.66 - 18.21)	10.36	0.74 (0.43 - 1.04)	0.43 (0.28 - 0.58)	-2.18 (-2.34 - -2.02)
Latvia	Female	27.96 (16.79 - 37.54)	35.93 (18.45 - 49.81)	28.51	1.91 (1.15 - 2.69)	3.57 (1.85 - 5.17)	3.16 (2.77 - 3.55)
Lebanon	Female	51.22 (33.44 - 71.17)	465.26 (291.92 - 685.45)	808.36	2.84 (1.91 - 3.81)	10.33 (6.54 - 15.15)	5.6 (5.1 - 6.09)
Lesotho	Female	2.17 (1.55 - 3.31)	3.13 (2.03 - 5.09)	44.24	0.31 (0.23 - 0.48)	0.36 (0.24 - 0.57)	1.31 (0.64 - 1.98)
Liberia	Female	2.8 (1.86 - 3.86)	3.93 (2.75 - 5.49)	40.36	0.36 (0.25 - 0.49)	0.24 (0.16 - 0.32)	-1.94 (-2.24 - -1.64)
Libya	Female	38.99 (25.75 - 54.49)	174.57 (109.63 - 255.09)	347.73	2.45 (1.68 - 3.41)	5.04 (3.21 - 7.25)	3.48 (2.92 - 4.04)
Lithuania	Female	57 (39.86 - 77.14)	44.42 (32.62 - 73.31)	-22.07	2.86 (1.99 - 4.01)	2.93 (1.95 - 5.45)	-0.1 (-0.44 - 0.25)
Luxembourg	Female	4.53 (3.46 - 6.37)	9.69 (6.95 - 16.61)	113.91	2.05 (1.48 - 3.08)	3.1 (2.17 - 5.43)	1.14 (0.71 - 1.57)
Macedonia	Female	9.45 (6.45 - 12.8)	24.79 (14.5 - 36.75)	162.33	0.92 (0.63 - 1.24)	2.32 (1.34 - 3.59)	3.3 (3.04 - 3.57)
Madagascar	Female	44.04 (29.38 - 56.96)	60.47 (40.67 - 81.33)	37.31	0.89 (0.6 - 1.13)	0.58 (0.39 - 0.78)	-1.7 (-1.89 - -1.52)
Malawi	Female	18.5 (7.11 - 28.34)	22.03 (14.03 - 32.52)	19.08	0.46 (0.21 - 0.65)	0.29 (0.19 - 0.42)	-2.68 (-3.11 - -2.24)
Malaysia	Female	33.28 (14 - 49.8)	93.36 (34.59 - 168.95)	180.53	0.47 (0.2 - 0.67)	0.6 (0.22 - 1.07)	0.77 (0.28 - 1.26)
Maldives	Female	0.12 (0.06 - 0.2)	0.34 (0.22 - 0.5)	183.33	0.16 (0.08 - 0.27)	0.17 (0.12 - 0.25)	0.23 (-0.35 - 0.81)
Mali	Female	27.09 (16.25 - 36.59)	34.17 (20.28 - 50.12)	26.14	0.74 (0.49 - 0.92)	0.41 (0.28 - 0.57)	-2.42 (-2.6 - -2.24)
Malta	Female	4.31 (3.43 - 5.75)	9.41 (7.17 - 13.68)	118.33	2.15 (1.67 - 3.02)	4.04 (2.89 - 6.27)	2.71 (2.43 - 2.98)
Marshall Islands	Female	0.04 (0.03 - 0.06)	0.07 (0.04 - 0.11)	75.00	0.3 (0.21 - 0.39)	0.29 (0.17 - 0.43)	-0.11 (-0.33 - 0.11)
Mauritania	Female	2.72 (1.93 - 3.58)	3.54 (2.64 - 4.89)	30.15	0.34 (0.25 - 0.46)	0.23 (0.17 - 0.32)	-1.52 (-1.6 - -1.44)
Mauritius	Female	1.58 (1.21 - 1.97)	6.26 (2.31 - 9.15)	296.20	0.29 (0.23 - 0.35)	0.95 (0.35 - 1.42)	3.79 (3.05 - 4.55)
Mexico	Female	248.32 (192.33 - 272.23)	467.36 (380.74 - 576.44)	88.21	0.75 (0.59 - 0.84)	0.73 (0.6 - 0.9)	-0.11 (-0.3 - 0.08)
Moldova	Female	41.77 (30.75 - 53.73)	42.32 (29.2 - 57.94)	1.32	1.76 (1.29 - 2.33)	2.1 (1.34 - 3.05)	0.4 (0.23 - 0.58)
Mongolia	Female	1.41 (0.8 - 2.96)	2.87 (2.02 - 4.68)	103.55	0.18 (0.11 - 0.36)	0.18 (0.13 - 0.28)	-0.12 (-0.5 - 0.26)
Montenegro	Female	8.59 (5.89 - 12.53)	12.32 (8.43 - 18.04)	43.42	2.68 (1.84 - 3.92)	4.07 (2.7 - 6.01)	1.6 (1.46 - 1.75)
Morocco	Female	200.2 (127.13 - 259.17)	318.51 (197.6 - 465.27)	59.10	1.81 (1.21 - 2.27)	1.77 (1.12 - 2.56)	-0.01 (-0.13 - 0.12)
Mozambique	Female	72.03 (50.87 - 95.42)	74.57 (49.31 - 102.78)	3.53	1.15 (0.83 - 1.5)	0.6 (0.39 - 0.82)	-2.92 (-3.3 - -2.53)
Myanmar	Female	178.78 (100.84 - 245.08)	139.36 (96.42 - 185.27)	-22.05	1.02 (0.61 - 1.4)	0.51 (0.35 - 0.67)	-2.82 (-3.08 - -2.55)
Namibia	Female	3.82 (2.19 - 5.26)	4.46 (2.67 - 6.62)	16.75	0.7 (0.42 - 0.93)	0.41 (0.26 - 0.59)	-2.47 (-3.06 - -1.87)
Nepal	Female	57.39 (38.41 - 82.72)	56.25 (32.36 - 105)	-1.99	0.74 (0.51 - 1.04)	0.38 (0.22 - 0.7)	-2.51 (-2.81 - -2.21)
Netherlands	Female	236.96 (170.35 - 314.18)	320.53 (261.57 - 434.06)	35.27	2.84 (2.03 - 3.84)	3.57 (2.83 - 5.1)	0.81 (0.71 - 0.92)
New Zealand	Female	48.67 (21.9 - 67.79)	61.77 (31.95 - 78.05)	26.92	2.6 (1.18 - 3.64)	2.73 (1.41 - 3.52)	0.42 (0.17 - 0.68)
Nicaragua	Female	3.99 (2.88 - 5.5)	9.46 (5.36 - 13.52)	137.09	0.28 (0.21 - 0.38)	0.32 (0.18 - 0.44)	0.9 (0.64 - 1.17)
Niger	Female	11.73 (7.14 - 17.21)	17.35 (12.05 - 24.25)	47.91	0.4 (0.28 - 0.54)	0.26 (0.18 - 0.35)	-2.05 (-2.23 - -1.88)
Nigeria	Female	1004.66 (501.99 - 1717.02)	1827 (1048.41 - 2864.33)	81.85	2.69 (1.49 - 4.33)	2.11 (1.28 - 3.21)	-1.13 (-1.29 - -0.97)
North Korea	Female	38.43 (20.8 - 60.88)	47.71 (28.36 - 66.91)	24.15	0.35 (0.19 - 0.55)	0.34 (0.2 - 0.5)	-0.27 (-0.43 - -0.12)
Northern Mariana Islands	Female	0.03 (0.02 - 0.05)	0.06 (0.04 - 0.08)	100.00	0.16 (0.11 - 0.22)	0.28 (0.19 - 0.41)	2.07 (1.57 - 2.57)
Norway	Female	49.76 (39.32 - 64.01)	66.86 (57.07 - 93.97)	34.36	2.09 (1.61 - 2.77)	2.44 (2.01 - 3.5)	0.58 (0.22 - 0.94)
Oman	Female	5.63 (2.5 - 8.88)	23.14 (10.69 - 37.46)	311.01	0.94 (0.43 - 1.45)	1.41 (0.65 - 2.24)	1.46 (0.91 - 2.01)
Pakistan	Female	664.81 (450.03 - 909.15)	1540.25 (963.43 - 2335.07)	131.68	1.47 (1.03 - 2)	1.49 (0.96 - 2.25)	-0.04 (-0.24 - 0.17)
Palestine	Female	2.58 (1.26 - 5.4)	8.8 (5.83 - 13.12)	241.09	0.34 (0.18 - 0.71)	0.45 (0.32 - 0.63)	0.86 (0.57 - 1.15)
Panama	Female	2.52 (2.06 - 3.62)	9.53 (5.49 - 12.43)	278.17	0.25 (0.21 - 0.35)	0.49 (0.28 - 0.64)	2.88 (2.42 - 3.35)
Papua New Guinea	Female	6.38 (4.32 - 8.82)	12.59 (7.86 - 17.72)	97.34	0.44 (0.3 - 0.59)	0.36 (0.23 - 0.5)	-0.56 (-0.66 - -0.46)
Paraguay	Female	5.57 (4.25 - 7.41)	11.55 (7.67 - 15.45)	107.36	0.34 (0.26 - 0.45)	0.36 (0.23 - 0.47)	0.45 (0.12 - 0.78)
Peru	Female	21.44 (16.3 - 28.3)	40.79 (24.48 - 55.52)	90.25	0.26 (0.18 - 0.33)	0.25 (0.15 - 0.34)	0.05 (-0.11 - 0.22)
Philippines	Female	64.94 (52.4 - 94.78)	91.85 (59.34 - 204.78)	41.44	0.25 (0.21 - 0.36)	0.19 (0.13 - 0.41)	-1.16 (-1.43 - -0.89)
Poland	Female	394.3 (282.53 - 480.58)	542.96 (413.86 - 746.2)	37.70	1.91 (1.36 - 2.4)	2.91 (2.13 - 4.03)	1.35 (1.16 - 1.53)
Portugal	Female	62.18 (44.88 - 77.32)	104.94 (68.66 - 135.94)	68.77	1.09 (0.77 - 1.38)	1.82 (1.22 - 2.47)	1.59 (1.1 - 2.08)
Puerto Rico	Female	24.02 (12.44 - 32.54)	41.87 (25.62 - 58.53)	74.31	1.25 (0.64 - 1.7)	2.2 (1.3 - 3.25)	1.71 (1.45 - 1.97)
Qatar	Female	0.11 (0.08 - 0.16)	1.79 (1.09 - 2.62)	1527.27	0.12 (0.09 - 0.17)	0.29 (0.18 - 0.42)	4.06 (3.75 - 4.37)
Romania	Female	168.17 (112.88 - 208.85)	193.7 (138.99 - 257.02)	15.18	1.39 (0.92 - 1.77)	2.19 (1.49 - 3.09)	1.65 (1.41 - 1.9)
Russian Federation	Female	3109.79 (1729.38 - 4339.65)	3258.08 (2651.04 - 4446.32)	4.77	4.08 (2.15 - 5.91)	4.43 (3.5 - 6.2)	0.67 (0.33 - 1.01)
Rwanda	Female	30.64 (19.16 - 41.46)	29.51 (18.19 - 43.57)	-3.69	1.01 (0.65 - 1.34)	0.53 (0.33 - 0.75)	-3.05 (-3.4 - -2.7)
Saint Lucia	Female	0.16 (0.13 - 0.25)	0.29 (0.23 - 0.37)	81.25	0.26 (0.21 - 0.4)	0.3 (0.23 - 0.39)	0.7 (0.5 - 0.91)
Saint Vincent and the Grenadines	Female	0.08 (0.04 - 0.32)	0.32 (0.25 - 0.4)	300.00	0.17 (0.07 - 0.65)	0.55 (0.43 - 0.69)	3.04 (1.84 - 4.25)
Samoa	Female	0.31 (0.18 - 0.44)	0.34 (0.17 - 0.51)	9.68	0.48 (0.31 - 0.64)	0.41 (0.22 - 0.6)	-0.73 (-0.79 - -0.67)
Sao Tome and Principe	Female	0.03 (0.02 - 0.05)	0.05 (0.03 - 0.07)	66.67	0.06 (0.05 - 0.1)	0.06 (0.04 - 0.08)	-0.28 (-0.39 - -0.16)
Saudi Arabia	Female	36.68 (8.6 - 69.76)	307.94 (62.32 - 627.75)	739.53	0.67 (0.16 - 1.24)	1.91 (0.39 - 3.82)	4.43 (3.43 - 5.44)
Senegal	Female	9.19 (6.52 - 12.56)	14.52 (10.81 - 20.95)	58.00	0.31 (0.23 - 0.43)	0.26 (0.19 - 0.37)	-0.84 (-0.93 - -0.74)
Serbia	Female	90.99 (60.36 - 126.75)	105.18 (57.58 - 151.66)	15.60	1.91 (1.26 - 2.72)	2.32 (1.22 - 3.51)	1.31 (0.93 - 1.7)
Seychelles	Female	0.21 (0.13 - 0.44)	0.56 (0.39 - 0.81)	166.67	0.61 (0.37 - 1.23)	1.13 (0.79 - 1.69)	2.16 (1.77 - 2.55)
Sierra Leone	Female	5.14 (3.46 - 7)	7.82 (5.31 - 10.78)	52.14	0.32 (0.23 - 0.44)	0.27 (0.18 - 0.37)	-0.63 (-0.76 - -0.51)
Singapore	Female	4.18 (2.6 - 5.62)	25.7 (12.46 - 36.03)	514.83	0.26 (0.17 - 0.34)	0.8 (0.4 - 1.1)	5.82 (5.13 - 6.51)
Slovakia	Female	69.89 (50.86 - 91.28)	122.05 (86.31 - 188.4)	74.63	2.56 (1.85 - 3.38)	4.72 (3.26 - 7.29)	2.24 (1.82 - 2.66)

Slovenia	Female	26.85 (17.97 - 35.67)	26.35 (20.94 - 34.01)	-1.86	2.54 (1.67 - 3.46)	2.69 (2.1 - 3.61)	0.39 (0.15 - 0.63)
Solomon Islands	Female	0.36 (0.24 - 0.49)	0.67 (0.44 - 0.93)	86.11	0.34 (0.24 - 0.47)	0.29 (0.19 - 0.4)	-0.49 (-0.57 - -0.41)
Somalia	Female	24.89 (9.93 - 40.12)	61.93 (32.74 - 103.85)	148.81	0.98 (0.48 - 1.51)	0.97 (0.53 - 1.59)	-0.4 (-0.6 - -0.21)
South Africa	Female	43.43 (31.18 - 55.97)	60.35 (40.49 - 75.76)	38.96	0.26 (0.19 - 0.32)	0.21 (0.14 - 0.26)	-0.83 (-1.75 - 0.09)
South Korea	Female	17.88 (12.56 - 38.87)	145.8 (84.22 - 188.34)	715.44	0.08 (0.06 - 0.18)	0.55 (0.33 - 0.74)	8.51 (7.83 - 9.2)
South Sudan	Female	16.03 (6.25 - 27.57)	23.3 (15.47 - 32.27)	45.35	0.74 (0.34 - 1.18)	0.62 (0.42 - 0.87)	-0.93 (-1.19 - -0.67)
Spain	Female	381.13 (250.36 - 482.97)	649.11 (461.27 - 818.08)	70.31	1.72 (1.12 - 2.21)	2.71 (1.92 - 3.53)	1.72 (1.36 - 2.08)
Sri Lanka	Female	35.51 (27.57 - 47.02)	76.7 (50.42 - 124.83)	116.00	0.51 (0.39 - 0.66)	0.65 (0.42 - 1.08)	0.04 (-0.44 - 0.53)
Sudan	Female	48.81 (30.62 - 74.96)	74.4 (51.54 - 101.9)	52.43	0.65 (0.43 - 0.95)	0.47 (0.33 - 0.64)	-1.14 (-1.19 - -1.09)
Suriname	Female	0.81 (0.64 - 1.24)	1.53 (1.19 - 1.94)	88.89	0.46 (0.37 - 0.7)	0.51 (0.4 - 0.65)	0.62 (0.36 - 0.88)
Swaziland	Female	0.96 (0.67 - 1.47)	1.36 (0.84 - 2.21)	41.67	0.34 (0.25 - 0.53)	0.29 (0.19 - 0.45)	-0.04 (-0.65 - 0.58)
Sweden	Female	74.9 (60.12 - 95.13)	105.09 (81.1 - 133.74)	40.31	1.52 (1.17 - 2.07)	1.98 (1.51 - 2.67)	0.61 (0.36 - 0.85)
Switzerland	Female	107.02 (69.65 - 146.69)	109.28 (86.65 - 156.79)	2.11	2.9 (1.8 - 4.2)	2.46 (1.89 - 3.61)	-0.72 (-1.02 - -0.41)
Syria	Female	2.76 (1.64 - 3.72)	8.37 (4.29 - 12.94)	203.26	0.07 (0.04 - 0.09)	0.1 (0.05 - 0.16)	1.83 (1.5 - 2.16)
Tajikistan	Female	5.15 (3.87 - 6.78)	10.32 (7.83 - 15.28)	100.39	0.26 (0.18 - 0.34)	0.26 (0.2 - 0.35)	-0.24 (-0.38 - -0.1)
Tanzania	Female	83.61 (44.23 - 116.31)	137.83 (87.06 - 201.04)	64.85	0.79 (0.49 - 1.08)	0.58 (0.38 - 0.82)	-1.58 (-1.81 - -1.36)
Thailand	Female	69.71 (45.33 - 118.56)	123.25 (66.69 - 247.27)	76.80	0.26 (0.17 - 0.44)	0.35 (0.18 - 0.69)	0.85 (0.6 - 1.1)
The Bahamas	Female	0.74 (0.59 - 0.97)	1.41 (1.04 - 1.92)	90.54	0.58 (0.47 - 0.74)	0.69 (0.51 - 0.94)	0.73 (0.61 - 0.85)
The Gambia	Female	1.24 (0.76 - 1.69)	2.46 (1.66 - 3.54)	98.39	0.33 (0.22 - 0.44)	0.29 (0.21 - 0.41)	-0.32 (-0.46 - -0.18)
Timor-Leste	Female	1.58 (0.91 - 2.28)	2.03 (1.06 - 2.85)	28.48	0.59 (0.37 - 0.78)	0.38 (0.22 - 0.53)	-1.79 (-1.94 - -1.65)
Togo	Female	4.99 (3.51 - 6.74)	7.3 (5.07 - 10.26)	46.29	0.36 (0.26 - 0.48)	0.24 (0.17 - 0.34)	-1.71 (-1.88 - -1.55)
Tonga	Female	0.07 (0.04 - 0.09)	0.08 (0.05 - 0.11)	14.29	0.18 (0.13 - 0.25)	0.17 (0.11 - 0.25)	-0.28 (-0.38 - -0.18)
Trinidad and Tobago	Female	1.12 (0.88 - 1.37)	3.29 (1.13 - 5.2)	193.75	0.2 (0.16 - 0.24)	0.46 (0.15 - 0.75)	4.62 (3.71 - 5.53)
Tunisia	Female	57.56 (42.35 - 76.01)	121.55 (76.86 - 180.75)	111.17	1.55 (1.17 - 2.02)	2.04 (1.28 - 3.03)	0.92 (0.87 - 0.98)
Turkey	Female	152.84 (110.29 - 239.84)	316.19 (219.43 - 463.14)	106.88	0.59 (0.43 - 0.92)	0.76 (0.52 - 1.15)	1.46 (1 - 1.93)
Turkmenistan	Female	13.01 (8.85 - 16.44)	24.75 (14.01 - 33.57)	90.24	0.76 (0.55 - 0.93)	0.99 (0.58 - 1.35)	1.09 (0.78 - 1.4)
Uganda	Female	65.72 (44.34 - 85.58)	102.99 (65.68 - 163.5)	56.71	0.98 (0.69 - 1.25)	0.64 (0.42 - 1)	-2 (-2.22 - -1.79)
Ukraine	Female	495.47 (334.63 - 671.53)	920.01 (597.22 - 1461.34)	85.68	1.77 (1.14 - 2.45)	4.36 (2.66 - 7.18)	2.96 (2.33 - 3.59)
United Arab Emirates	Female	2.22 (1.51 - 3.37)	17.3 (10.54 - 25.82)	679.28	0.46 (0.32 - 0.67)	0.73 (0.47 - 1.08)	1.88 (1.66 - 2.09)
United Kingdom	Female	777.1 (541.81 - 864.17)	1081.5 (873.85 - 1257.85)	39.17	2.32 (1.59 - 2.61)	2.97 (2.45 - 3.48)	0.84 (0.72 - 0.95)
United States	Female	4330.17 (3098.23 - 4779.99)	4797.86 (4053.5 - 7646.82)	10.80	3.07 (2.19 - 3.41)	2.77 (2.29 - 4.59)	-1.01 (-1.32 - -0.69)
Uruguay	Female	13.86 (9.34 - 16.78)	16.86 (11.62 - 22.26)	21.65	0.79 (0.52 - 0.98)	0.86 (0.57 - 1.19)	0.51 (0.18 - 0.83)
Uzbekistan	Female	50.41 (36.79 - 75.02)	143.13 (102.7 - 198.24)	183.93	0.54 (0.39 - 0.82)	0.86 (0.62 - 1.18)	1.93 (1.7 - 2.16)
Vanuatu	Female	0.24 (0.11 - 0.43)	0.52 (0.21 - 0.96)	116.67	0.46 (0.22 - 0.81)	0.46 (0.18 - 0.82)	-0.05 (-0.2 - 0.09)
Venezuela	Female	45.1 (30.92 - 56.01)	140.57 (64.98 - 199.76)	211.69	0.61 (0.42 - 0.73)	0.88 (0.42 - 1.24)	1.85 (1.57 - 2.12)
Vietnam	Female	130.27 (82.88 - 170.86)	202.93 (127.56 - 290.86)	55.78	0.43 (0.28 - 0.55)	0.4 (0.25 - 0.59)	-0.4 (-0.65 - -0.15)
Virgin Islands, U.S.	Female	0.07 (0.05 - 0.13)	0.22 (0.16 - 0.29)	214.29	0.14 (0.1 - 0.25)	0.38 (0.27 - 0.53)	5.14 (4.36 - 5.91)
Yemen	Female	29.38 (14.57 - 47.15)	64.46 (44.24 - 88.49)	119.40	0.67 (0.38 - 1.02)	0.56 (0.39 - 0.78)	-0.64 (-0.71 - -0.57)
Zambia	Female	34.64 (20.08 - 48.07)	45.43 (30.2 - 62.37)	31.15	1.1 (0.67 - 1.46)	0.64 (0.43 - 0.85)	-2.41 (-2.65 - -2.17)
Zimbabwe	Female	11.41 (6.58 - 15.31)	21.34 (11.45 - 31.82)	87.03	0.3 (0.19 - 0.38)	0.36 (0.21 - 0.5)	1.85 (1.01 - 2.7)
Afghanistan	Male	113.56 (39.51 - 202.27)	210.05 (125.48 - 338.46)	84.97	2.75 (0.94 - 5)	2.15 (1.33 - 3.43)	-0.95 (-1.37 - -0.54)
Albania	Male	20.86 (14.62 - 40.07)	33.01 (18.79 - 61.77)	58.25	1.37 (0.99 - 2.7)	2.32 (1.31 - 4.49)	1.85 (1.62 - 2.09)
Algeria	Male	224.23 (151.2 - 294.06)	358.19 (225.16 - 506.18)	59.74	2.07 (1.48 - 2.7)	1.78 (1.12 - 2.49)	-0.44 (-0.52 - -0.36)
American Samoa	Male	0.03 (0.03 - 0.05)	0.05 (0.03 - 0.07)	66.67	0.19 (0.15 - 0.28)	0.21 (0.13 - 0.26)	0.7 (0.45 - 0.94)
Andorra	Male	1.26 (0.78 - 1.92)	2.01 (1.42 - 3.12)	59.52	3.71 (2.33 - 5.59)	4.29 (3.03 - 6.49)	0.3 (-0.05 - 0.64)
Angola	Male	45.68 (26.23 - 67.86)	60.79 (44.24 - 85.47)	33.08	1.25 (0.8 - 1.8)	0.73 (0.51 - 1.05)	-2.1 (-2.29 - -1.9)
Antigua and Barbuda	Male	0.13 (0.11 - 0.2)	0.28 (0.2 - 0.4)	115.38	0.51 (0.41 - 0.75)	0.61 (0.44 - 0.88)	0.9 (0.66 - 1.14)
Argentina	Male	201.45 (166.45 - 290.25)	266.82 (202.47 - 411.17)	32.45	1.3 (1.07 - 1.87)	1.17 (0.89 - 1.8)	-0.65 (-0.88 - -0.42)
Armenia	Male	4.58 (3.25 - 8.72)	12.43 (5.51 - 17.54)	171.40	0.31 (0.22 - 0.55)	0.77 (0.35 - 1.1)	3.56 (2.43 - 4.71)
Australia	Male	227.89 (155.11 - 281.3)	485.13 (358.63 - 643.28)	112.88	2.58 (1.76 - 3.22)	3.75 (2.75 - 5.08)	1.35 (1.03 - 1.68)
Austria	Male	138.17 (78.35 - 182.13)	130.47 (94.33 - 235.22)	-5.57	3.24 (1.82 - 4.31)	2.52 (1.75 - 4.79)	-1.19 (-1.62 - -0.76)
Azerbaijan	Male	52.51 (35.79 - 70.2)	97.71 (58.22 - 143.48)	86.08	1.57 (1.12 - 2.12)	1.9 (1.13 - 2.78)	0.61 (0.37 - 0.86)
Bahrain	Male	3.24 (1.6 - 4.45)	7.37 (5.03 - 10.22)	127.47	1.82 (0.81 - 2.5)	0.86 (0.57 - 1.2)	-3.96 (-4.52 - -3.39)
Bangladesh	Male	951.85 (631.17 - 1581.49)	468.27 (298.1 - 926.78)	-50.80	2.12 (1.42 - 3.61)	0.63 (0.4 - 1.23)	-4.68 (-4.78 - -4.58)
Barbados	Male	1.35 (1.01 - 1.78)	1.66 (1.25 - 2.33)	22.96	1.07 (0.8 - 1.39)	1.04 (0.76 - 1.48)	-0.43 (-0.67 - -0.2)
Belarus	Male	145.73 (81.16 - 205.61)	193.6 (116.57 - 286.18)	32.85	2.86 (1.58 - 4.09)	3.91 (2.37 - 5.95)	1.6 (1.35 - 1.86)
Belgium	Male	199.47 (142.64 - 274.83)	244.55 (171.94 - 432.3)	22.60	3.58 (2.51 - 5.12)	3.91 (2.67 - 7.29)	0.25 (0.06 - 0.44)
Belize	Male	0.68 (0.46 - 0.97)	1.45 (1.05 - 1.93)	113.24	0.94 (0.65 - 1.35)	0.81 (0.6 - 1.05)	-0.99 (-1.28 - -0.71)
Benin	Male	15.55 (10.49 - 20.27)	21.25 (15.31 - 28.24)	36.66	0.87 (0.61 - 1.11)	0.58 (0.41 - 0.75)	-1.56 (-1.65 - -1.47)
Bermuda	Male	0.32 (0.21 - 0.54)	0.79 (0.57 - 1.23)	146.88	1.05 (0.7 - 1.79)	2.38 (1.56 - 3.98)	3.42 (3.16 - 3.67)
Bhutan	Male	4.27 (2.76 - 7.1)	3.16 (1.62 - 6.65)	-26.00	1.87 (1.15 - 3.16)	0.7 (0.37 - 1.48)	-3.73 (-3.86 - -3.6)
Bolivia	Male	47.5 (30.27 - 60.2)	46.18 (30.86 - 67.45)	-2.78	1.97 (1.29 - 2.52)	0.96 (0.64 - 1.39)	-2.66 (-2.73 - -2.59)
Bosnia and Herzegovina	Male	21.47 (14.83 - 26.2)	22.52 (12.91 - 31.41)	4.89	0.98 (0.66 - 1.2)	1.2 (0.68 - 1.8)	0.87 (0.5 - 1.25)
Botswana	Male	2.77 (2 - 4.1)	3.25 (2.37 - 4.79)	17.33	0.74 (0.54 - 1.09)	0.39 (0.29 - 0.56)	-2.43 (-2.63 - -2.23)
Brazil	Male	632.15 (505.71 - 838.26)	860.9 (614.37 - 1198.15)	36.19	1.05 (0.82 - 1.38)	0.79 (0.57 - 1.11)	-0.89 (-0.98 - -0.8)
Brunei	Male	0.79 (0.52 - 1.46)	3.69 (2.51 - 5.25)	367.09	0.8 (0.55 - 1.46)	1.65 (1.13 - 2.25)	3.86 (3.4 - 4.31)
Bulgaria	Male	134.95 (106.17 - 183.37)	153.35 (111.56 - 207.68)	13.63	2.84 (2.16 - 3.72)	4.14 (2.85 - 5.96)	1.38 (1.17 - 1.59)
Burkina Faso	Male	33.1 (22.81 - 44.84)	46.31 (31.75 - 61.63)	39.91	0.91 (0.63 - 1.27)	0.65 (0.43 - 0.83)	-1.42 (-1.69 - -1.16)
Burundi	Male	45.73 (30.14 - 61.8)	52.58 (36.27 - 70.68)	14.98	2.2 (1.51 - 2.99)	1.28 (0.87 - 1.69)	-2.27 (-2.43 - -2.11)
Cambodia	Male	54.66 (37.04 - 73.2)	54.15 (38.39 - 79.28)	-0.93	1.78 (1.21 - 2.51)	0.89 (0.63 - 1.27)	-2.66 (-2.73 - -2.59)
Cameroon	Male	30.62 (20.91 - 39.52)	55.64 (37.56 - 75.97)	81.71	0.8 (0.56 - 1.01)	0.6 (0.39 - 0.8)	-1.19 (-1.39 - -0.99)
Canada	Male	604.23 (416.97 - 851.25)	903.66 (666.9 - 1445.51)	49.56	4.16 (2.81 - 5.95)	4.87 (3.52 - 7.62)	0.76 (-0.25 - 1.8)
Cape Verde	Male	0.22 (0.14 - 0.28)	0.37 (0.21 - 0.48)	68.18	0.19 (0.11 - 0.26)	0.15 (0.08 - 0.2)	-1.05 (-1.22 - -0.89)
Central African Republic	Male	11.78 (7.35 - 17.43)	19.46 (13.57 - 27.66)	65.20	1.31 (0.89 - 1.93)	1.19 (0.85 - 1.74)	-0.56 (-0.66 - -0.46)
Chad	Male	18.14 (12.52 - 23.76)	34.55 (24.86 - 45.87)	90.46	0.78 (0.57 - 1.06)	0.68 (0.47 - 0.91)	-0.44 (-0.67 - -0.21)
Chile	Male	66.89 (48.45 - 88.87)	119.71 (87.27 - 185.26)	78.97	1.16 (0.86 - 1.54)	1.24 (0.89 - 1.91)	0.63 (0.29 - 0.96)
China	Male	5930.4 (3226.07 - 7255.07)	12086.66 (7751.45 - 14038.72)	103.81	1.08 (0.59 - 1.32)	1.5 (0.98 - 1.75)	1.05 (0.37 - 1.73)
Colombia	Male	169.58 (122.7 - 254.69)	245.05 (176.43 - 379.71)	44.50	1.26 (0.95 - 1.79)	0.97 (0.71 - 1.52)	-1.36 (-1.62 - -1.09)
Comoros	Male	3.5 (2.59 - 4.94)	3.59 (2.41 - 5.38)	2.57	1.89 (1.43 - 2.71)	1.19 (0.8 - 1.75)	-1.83 (-1.99 - -1.67)
Congo	Male	9.08 (6.72 - 12.64)	12.34 (7.94 - 19.37)	35.90	1.15 (0.87 - 1.59)	0.7 (0.45 - 1.09)	-2.17 (-2.38 - -1.96)
Costa Rica	Male	34.15 (15.78 - 47.73)	56.08 (31.11 - 78.07)	64.22	2.59 (1.29 - 3.41)	2.37 (1.35 - 3.28)	-0.54 (-0.98 - -0.11)
Cote d'Ivoire	Male	39.45 (26.18 - 50.55)	66.37 (44.34 - 91.81)	68.24	0.84 (0.59 - 1.07)	0.68 (0.47 - 0.93)	-1.02 (-1.22 - -0.83)
Croatia	Male	90.86 (52.14 - 128.23)	72.71 (53.09 - 105.67)	-19.98	3.63 (2.07 - 5.28)	3.43 (2.38 - 5.16)	0.57 (0.17 - 0.97)
Cuba	Male	21.75 (13.04 - 53.12)	143.76 (37.12 - 232.88)	560.97	0.38 (0.23 - 0.93)	2.25 (0.57 - 3.76)	6.8 (4.93 - 8.7)
Cyprus	Male	5.87 (3.68 - 8.01)	20.92 (14.51 - 31.66)	256.39	1.45 (0.91 - 1.98)	2.85 (1.92 - 4.43)	2.98 (2.58 - 3.38)
Czech Republic	Male	227.1 (151.67 - 300.44)	227.89 (156 - 417.62)	0.35	4.32 (2.86 - 5.83)	4.35 (2.82 - 8.25)	0.12 (-0.04 - 0.29)
Democratic Republic of the Congo	Male	113.73 (78.69 - 164.12)	192.11 (136.96 - 280.66)	68.92	0.91 (0.61 - 1.4)	0.74 (0.51 - 1.13)	-0.71 (-0.79 - -0.62)
Denmark	Male	88.28 (65.42 - 122.12)	128.76 (96.3 - 218.61)	45.85	3.15 (2.27 - 4.29)	3.98 (2.88 - 7.38)	0.97 (0.74 - 1.19)

Djibouti	Male	3.13 (1.73 - 4.68)	5.57 (3.61 - 8.6)	77.96	1.5 (0.96 - 2.15)	1.12 (0.73 - 1.69)	-1.37 (-1.62 - -1.12)
Dominica	Male	0.51 (0.38 - 0.78)	0.52 (0.4 - 0.72)	1.96	1.53 (1.14 - 2.34)	1.37 (1.04 - 1.91)	-0.54 (-0.7 - -0.39)
Dominican Republic	Male	6.45 (4.59 - 12.76)	22.27 (9.82 - 32.01)	245.27	0.23 (0.17 - 0.46)	0.44 (0.19 - 0.64)	1.32 (0.53 - 2.13)
Ecuador	Male	42.29 (34.38 - 67.07)	76.3 (55.35 - 130.91)	80.42	1.04 (0.87 - 1.64)	0.96 (0.71 - 1.58)	0.09 (-0.16 - 0.35)
Egypt	Male	678.66 (465.52 - 918.87)	1069.23 (674.03 - 1439.93)	57.55	3.03 (2.16 - 4.15)	2.44 (1.56 - 3.21)	-0.53 (-0.71 - -0.34)
El Salvador	Male	32.31 (23.94 - 42.15)	30.63 (19.88 - 58.23)	-5.20	1.68 (1.2 - 2.19)	1.14 (0.76 - 2.03)	-1.69 (-1.91 - -1.47)
Equatorial Guinea	Male	2.02 (1.35 - 3.03)	1.8 (1.17 - 2.83)	-10.89	1.46 (1.07 - 2.08)	0.43 (0.28 - 0.64)	-5.34 (-5.86 - -4.82)
Eritrea	Male	33.34 (15.37 - 50.68)	53.44 (32.98 - 84.01)	60.29	3.27 (1.9 - 4.74)	2.37 (1.48 - 3.65)	-1.59 (-1.96 - -1.21)
Estonia	Male	27.06 (15.74 - 37.34)	31.66 (19.34 - 58.37)	17.00	3.55 (2.06 - 5.02)	4.8 (2.86 - 9.45)	1.63 (1.05 - 2.21)
Ethiopia	Male	811.24 (539.3 - 1093.2)	754.66 (472.59 - 1285.85)	-6.97	4.15 (2.8 - 5.56)	1.97 (1.22 - 3.35)	-3 (-3.15 - -2.84)
Federated States of Micronesia	Male	0.25 (0.17 - 0.38)	0.23 (0.14 - 0.33)	-8.00	0.69 (0.46 - 1.06)	0.52 (0.32 - 0.74)	-1.07 (-1.13 - -1.01)
Fiji	Male	1.25 (0.79 - 1.61)	2.33 (0.99 - 3.46)	86.40	0.4 (0.27 - 0.51)	0.54 (0.23 - 0.78)	1.57 (1.22 - 1.92)
Finland	Male	78.6 (50.92 - 111.24)	137.12 (102.94 - 211.85)	74.45	2.88 (1.9 - 4.06)	4.54 (3.28 - 7.08)	2.15 (1.93 - 2.38)
France	Male	932.25 (684.55 - 1374.41)	1382.09 (986.3 - 2204.07)	48.25	3.04 (2.2 - 4.52)	4.04 (2.73 - 6.62)	1.4 (1.1 - 1.7)
Gabon	Male	3.11 (2.29 - 4.26)	4.21 (2.95 - 6.04)	35.37	0.88 (0.65 - 1.19)	0.64 (0.45 - 0.91)	-1.09 (-1.3 - -0.89)
Georgia	Male	94.79 (61.49 - 121.48)	59.02 (44.13 - 92.06)	-37.74	3.54 (2.27 - 4.58)	2.95 (2.16 - 4.69)	-1.48 (-1.79 - -1.17)
Germany	Male	1909.86 (1116.47 - 2558.3)	1988.3 (1403.58 - 3413.52)	4.11	4.32 (2.5 - 5.86)	4.17 (2.82 - 7.68)	-0.52 (-0.66 - -0.37)
Ghana	Male	10.69 (7.79 - 18.13)	18.22 (13.48 - 29.12)	70.44	0.2 (0.15 - 0.34)	0.18 (0.13 - 0.27)	-0.06 (-0.22 - 0.1)
Greece	Male	280.59 (211.29 - 467.18)	457.27 (347.59 - 657.35)	62.97	4.8 (3.48 - 8.26)	7.48 (5.39 - 12.07)	1.9 (1.19 - 2.63)
Greenland	Male	0.37 (0.18 - 0.49)	0.21 (0.15 - 0.27)	-43.24	1.37 (0.61 - 1.83)	0.6 (0.43 - 0.78)	-3.66 (-3.92 - -3.4)
Grenada	Male	0.38 (0.28 - 0.51)	0.34 (0.27 - 0.53)	-10.53	1.11 (0.81 - 1.43)	0.54 (0.42 - 0.84)	-2.32 (-2.55 - -2.1)
Guam	Male	0.4 (0.27 - 0.64)	0.69 (0.5 - 1)	72.50	0.6 (0.42 - 0.94)	0.79 (0.57 - 1.16)	1.33 (0.8 - 1.86)
Guatemala	Male	33.06 (26.4 - 47.95)	41.62 (32.72 - 62.63)	25.89	1.1 (0.91 - 1.53)	0.63 (0.5 - 0.89)	-2.14 (-2.46 - -1.82)
Guinea	Male	34.68 (21.75 - 46.12)	42.52 (26.07 - 55.26)	22.61	1.23 (0.85 - 1.59)	0.97 (0.63 - 1.31)	-0.77 (-0.9 - -0.64)
Guinea-Bissau	Male	4.66 (2.95 - 6.46)	5.07 (3.63 - 7.16)	8.80	1.28 (0.86 - 1.76)	0.84 (0.6 - 1.13)	-1.52 (-1.57 - -1.48)
Guyana	Male	1.09 (0.58 - 2.95)	2.19 (1.38 - 2.82)	100.92	0.39 (0.22 - 1.02)	0.63 (0.4 - 0.81)	1.9 (0.88 - 2.92)
Haiti	Male	48.85 (34.69 - 78.5)	65.94 (39.24 - 107.81)	34.98	2.15 (1.43 - 3.65)	1.56 (0.91 - 2.56)	-1.13 (-1.19 - -1.07)
Honduras	Male	7.48 (4.95 - 10.17)	10.76 (6.8 - 14.83)	43.85	0.48 (0.3 - 0.62)	0.32 (0.18 - 0.47)	-1.48 (-1.54 - -1.42)
Hungary	Male	150.2 (100.18 - 197.42)	105 (74.76 - 169.59)	-30.09	2.77 (1.8 - 3.67)	2.19 (1.45 - 3.67)	-0.85 (-1.08 - -0.62)
Iceland	Male	5.02 (3.3 - 7.43)	6.58 (5.08 - 11.09)	31.08	3.76 (2.46 - 5.57)	3.54 (2.67 - 5.97)	0.11 (-0.16 - 0.38)
India	Male	4926.98 (3148.46 - 5890.19)	4380.93 (3402.19 - 5837.43)	-11.08	1.3 (0.84 - 1.53)	0.68 (0.52 - 0.91)	-2.42 (-2.55 - -2.29)
Indonesia	Male	728.51 (500.79 - 985.68)	787.71 (571.45 - 1123.99)	8.13	0.99 (0.68 - 1.39)	0.66 (0.48 - 0.94)	-1.4 (-1.5 - -1.3)
Iran	Male	167.49 (121.97 - 281)	642.12 (334.45 - 969.8)	283.38	0.71 (0.54 - 1.2)	1.51 (0.79 - 2.34)	4.24 (3.67 - 4.82)
Iraq	Male	68.75 (38.18 - 91.56)	65.71 (49.56 - 107.77)	-4.42	1.08 (0.63 - 1.4)	0.35 (0.27 - 0.58)	-4.41 (-5.3 - -3.52)
Ireland	Male	48.86 (33.17 - 65.35)	114.17 (82.76 - 163.86)	133.67	2.71 (1.83 - 3.6)	4.55 (3.25 - 6.47)	2.32 (1.94 - 2.69)
Israel	Male	39.55 (29.86 - 55.69)	107.67 (76.23 - 174.72)	172.24	1.7 (1.29 - 2.41)	2.44 (1.71 - 3.98)	0.84 (0.27 - 1.4)
Italy	Male	1256.38 (857.87 - 1700.07)	1528.37 (1100.84 - 2368.7)	21.65	4.03 (2.65 - 5.54)	4.8 (3.26 - 7.51)	1.22 (0.95 - 1.49)
Jamaica	Male	5.71 (2.71 - 7.49)	9.19 (4.23 - 13.54)	60.95	0.57 (0.28 - 0.73)	0.63 (0.29 - 0.92)	0.15 (-0.35 - 0.66)
Japan	Male	476.38 (378.57 - 633.51)	971.52 (667.51 - 1156.16)	103.94	0.68 (0.54 - 0.92)	1.2 (0.83 - 1.46)	2.83 (2.55 - 3.11)
Jordan	Male	5.6 (3.75 - 7.5)	16.45 (11.33 - 24.99)	193.75	0.4 (0.25 - 0.52)	0.32 (0.22 - 0.45)	-1.09 (-1.34 - -0.84)
Kazakhstan	Male	98.15 (68.11 - 147.98)	131.97 (92.48 - 190.33)	34.46	1.31 (0.92 - 1.95)	1.5 (1.05 - 2.18)	0.33 (0.18 - 0.48)
Kenya	Male	65.64 (49.28 - 95.96)	160.21 (112.4 - 199.61)	144.07	0.78 (0.62 - 1.16)	0.87 (0.6 - 1.07)	0.68 (0.37 - 0.98)
Kiribati	Male	0.1 (0.08 - 0.14)	0.17 (0.11 - 0.22)	70.00	0.36 (0.29 - 0.52)	0.35 (0.25 - 0.45)	-0.24 (-0.42 - -0.07)
Kuwait	Male	7.12 (4.01 - 10.65)	16.98 (11.64 - 25.64)	138.48	0.76 (0.45 - 1.09)	0.69 (0.48 - 1.1)	0.27 (-0.19 - 0.73)
Kyrgyzstan	Male	17.88 (10.05 - 22.99)	16.18 (11.89 - 25.75)	-9.51	0.98 (0.57 - 1.22)	0.57 (0.43 - 0.87)	-1.64 (-1.95 - -1.32)
Laos	Male	29 (18.96 - 37.53)	24.38 (15.59 - 38.42)	-15.93	1.87 (1.24 - 2.57)	0.88 (0.57 - 1.41)	-2.85 (-2.95 - -2.76)
Latvia	Male	38.32 (22.6 - 52.3)	43.35 (22.02 - 64.15)	13.13	2.9 (1.68 - 4.01)	4.45 (2.25 - 6.81)	2.09 (1.68 - 2.5)
Lebanon	Male	57.35 (38.86 - 86.6)	485.39 (267.6 - 798.38)	746.36	3.53 (2.49 - 5.03)	10.47 (6 - 16.77)	5.08 (4.41 - 5.75)
Lesotho	Male	5.34 (4.16 - 7.53)	7.5 (4.85 - 10.42)	40.45	0.92 (0.72 - 1.29)	1.08 (0.72 - 1.48)	1.17 (0.75 - 1.6)
Liberia	Male	7.13 (4.91 - 9.3)	7.74 (5.47 - 10.89)	8.56	0.78 (0.55 - 1.01)	0.48 (0.34 - 0.67)	-2.02 (-2.35 - -1.7)
Libya	Male	42.07 (27.52 - 61.49)	127.23 (73.52 - 189.15)	202.42	2.46 (1.7 - 3.51)	3.54 (2.11 - 5.21)	2.14 (1.58 - 2.71)
Lithuania	Male	59.48 (37.47 - 83.91)	54.28 (36.45 - 76.78)	-8.74	3.21 (2 - 4.56)	3.83 (2.45 - 5.71)	0.84 (0.58 - 1.09)
Luxembourg	Male	6.9 (4.63 - 9.95)	12.29 (8.22 - 25.74)	78.12	3.21 (2.12 - 4.71)	3.62 (2.39 - 8.05)	-0.17 (-0.55 - 0.21)
Macedonia	Male	19.31 (9.22 - 27.26)	25.26 (13.11 - 37.25)	30.81	1.87 (0.92 - 2.61)	2.09 (1.06 - 3.24)	-0.07 (-0.44 - 0.3)
Madagascar	Male	103.89 (77.91 - 140.26)	118.09 (81.22 - 165.62)	13.67	1.87 (1.43 - 2.55)	1.19 (0.82 - 1.64)	-1.82 (-2.09 - -1.55)
Malawi	Male	38.89 (12.51 - 59.92)	53.7 (37.8 - 72.79)	38.08	0.99 (0.41 - 1.47)	0.83 (0.59 - 1.08)	-1.09 (-1.43 - -0.75)
Malaysia	Male	60.79 (36.92 - 81.27)	153.6 (73.7 - 239.11)	152.67	0.87 (0.57 - 1.15)	0.94 (0.46 - 1.45)	-0.07 (-0.52 - 0.39)
Maldives	Male	0.41 (0.3 - 0.56)	0.74 (0.49 - 1.16)	80.49	0.52 (0.4 - 0.7)	0.29 (0.21 - 0.43)	-2.29 (-2.61 - -1.97)
Mali	Male	66.32 (39.68 - 88.03)	77.09 (52.1 - 104.65)	16.24	1.71 (1.18 - 2.16)	0.89 (0.66 - 1.21)	-2.36 (-2.63 - -2.08)
Malta	Male	4.81 (3.66 - 7.22)	11.94 (8.64 - 18.67)	148.23	2.55 (1.93 - 3.85)	4.85 (3.33 - 7.87)	2.56 (2.29 - 2.84)
Marshall Islands	Male	0.1 (0.08 - 0.13)	0.14 (0.09 - 0.19)	40.00	0.71 (0.52 - 0.91)	0.6 (0.38 - 0.77)	-0.42 (-0.55 - -0.3)
Mauritania	Male	5.82 (4.15 - 7.48)	6.29 (4.26 - 9.61)	8.08	0.76 (0.55 - 0.97)	0.44 (0.3 - 0.66)	-2.03 (-2.16 - -1.9)
Mauritius	Male	3.97 (2.48 - 5.15)	5.3 (3.19 - 7.51)	33.50	0.79 (0.52 - 0.98)	0.78 (0.47 - 1.15)	-0.9 (-1.35 - -0.45)
Mexico	Male	444.83 (322.94 - 540.97)	713.63 (563.62 - 1008.76)	60.43	1.39 (1.02 - 1.71)	1.19 (0.94 - 1.69)	-0.68 (-0.94 - -0.42)
Moldova	Male	63.5 (39.24 - 94.63)	47.13 (35.02 - 75.06)	-25.78	3.01 (1.87 - 4.49)	2.4 (1.71 - 3.79)	-1.26 (-1.52 - -1)
Mongolia	Male	3 (2.04 - 4.02)	4.2 (3.01 - 5.44)	40.00	0.46 (0.26 - 0.61)	0.3 (0.2 - 0.37)	-2.21 (-2.49 - -1.93)
Montenegro	Male	15.05 (10.43 - 21.66)	16.9 (11.53 - 26.66)	12.29	4.69 (3.28 - 6.72)	5.36 (3.46 - 8.8)	0.3 (0.15 - 0.45)
Morocco	Male	253.94 (167.13 - 325.76)	333.31 (212.89 - 472.33)	31.26	2.31 (1.57 - 2.94)	1.89 (1.21 - 2.64)	-0.84 (-0.9 - -0.78)
Mozambique	Male	142.74 (104.46 - 205.66)	200.23 (140.27 - 265.03)	40.28	2.44 (1.9 - 3.58)	1.86 (1.25 - 2.5)	-0.78 (-0.99 - -0.58)
Myanmar	Male	289.76 (197.58 - 390.5)	211.92 (148.61 - 300.23)	-26.86	1.83 (1.26 - 2.53)	0.92 (0.64 - 1.28)	-2.66 (-2.83 - -2.49)
Namibia	Male	6.38 (4.48 - 8.15)	7.58 (4.68 - 10.18)	18.81	1.33 (1.03 - 1.83)	0.87 (0.61 - 1.12)	-1.72 (-2.13 - -1.31)
Nepal	Male	131.39 (86.11 - 215.6)	89.51 (51.17 - 179.06)	-31.87	1.74 (1.15 - 2.92)	0.73 (0.41 - 1.47)	-3.27 (-3.6 - -2.94)
Netherlands	Male	334.71 (219.15 - 463.64)	410.14 (310.65 - 607.5)	22.54	4.03 (2.62 - 5.6)	4.26 (3.18 - 6.64)	0.54 (0.33 - 0.75)
New Zealand	Male	56.59 (22.98 - 82.5)	78.06 (41.89 - 100.08)	37.94	3.21 (1.3 - 4.7)	3.42 (1.83 - 4.43)	0.04 (-0.14 - 0.22)
Nicaragua	Male	9.62 (5.99 - 12.12)	13.06 (7.98 - 18.04)	35.76	0.69 (0.45 - 0.87)	0.48 (0.29 - 0.63)	-1.56 (-1.71 - -1.41)
Niger	Male	35.19 (21.69 - 51.22)	40 (28.74 - 56.96)	13.67	1.01 (0.71 - 1.33)	0.59 (0.42 - 0.81)	-2.35 (-2.55 - -2.14)
Nigeria	Male	1362.13 (893.11 - 1870.9)	1869.29 (1259.81 - 2594.43)	37.23	3.34 (2.32 - 4.55)	2.2 (1.58 - 3.16)	-1.96 (-2.16 - -1.76)
North Korea	Male	56.07 (34.99 - 81.12)	89.85 (58.95 - 121.88)	60.25	0.68 (0.42 - 0.95)	0.68 (0.45 - 0.91)	-0.05 (-0.11 - 0)
Northern Mariana Islands	Male	0.06 (0.04 - 0.1)	0.11 (0.07 - 0.15)	83.33	0.28 (0.19 - 0.46)	0.44 (0.31 - 0.62)	2.08 (1.67 - 2.5)
Norway	Male	82.18 (61.97 - 130.96)	109 (85.55 - 187.96)	32.64	3.56 (2.65 - 5.7)	3.72 (2.86 - 6.41)	0.43 (0.04 - 0.83)
Oman	Male	13.01 (7.69 - 18.36)	54.44 (30.22 - 86.71)	318.45	1.59 (0.99 - 2.16)	1.8 (0.97 - 2.75)	0.56 (0.01 - 1.12)
Pakistan	Male	1487.28 (1049.84 - 2069.09)	2298.68 (1657.97 - 3161.92)	54.56	3.01 (2.19 - 4.31)	2.39 (1.73 - 3.28)	-1.09 (-1.28 - -0.89)
Palestine	Male	6.08 (3.4 - 12.69)	15.73 (11.34 - 31.3)	158.72	0.91 (0.55 - 1.78)	0.8 (0.61 - 1.48)	-0.45 (-0.76 - -0.13)
Panama	Male	5.06 (3.72 - 6.54)	14.23 (7.9 - 20.88)	181.23	0.49 (0.37 - 0.63)	0.72 (0.4 - 1.06)	1.64 (1.28 - 2)
Papua New Guinea	Male	15.84 (11.49 - 21.28)	30.46 (19.61 - 41.18)	92.30	0.98 (0.71 - 1.33)	0.8 (0.52 - 1.07)	-0.48 (-0.61 - -0.34)
Paraguay	Male	12.46 (8.59 - 19.15)	24.54 (16.36 - 34.45)	96.95	0.81 (0.57 - 1.21)	0.77 (0.52 - 1.07)	-0.07 (-0.16 - 0.02)
Peru	Male	62.95 (50.65 - 90.6)	77.65 (53.78 - 110.46)	23.35	0.73 (0.57 - 1.03)	0.49 (0.34 - 0.68)	-1.46 (-1.58 - -1.35)
Philippines	Male	141.85 (113.6 - 228.33)	165.07 (101.39 - 368.76)	16.37	0.57 (0.47 - 0.87)	0.37 (0.23 - 0.79)	-1.68 (-1.89 - -1.47)

Poland	Male	545.38 (347.27 - 649.16)	558.35 (421.73 - 798.3)	2.38	2.76 (1.78 - 3.32)	2.85 (2.02 - 4.37)	0.06 (-0.19 - 0.31)
Portugal	Male	113.17 (82.1 - 200.54)	171.13 (118.93 - 296.32)	51.21	2.16 (1.55 - 3.77)	3.05 (2.03 - 5.29)	0.87 (0.41 - 1.33)
Puerto Rico	Male	28.62 (19.34 - 38.41)	56.65 (32.81 - 79.87)	97.94	1.64 (1.1 - 2.2)	3.05 (1.77 - 4.49)	2.5 (2.28 - 2.72)
Qatar	Male	0.88 (0.57 - 1.59)	19.42 (9.31 - 41.88)	2106.82	0.43 (0.3 - 0.72)	0.85 (0.44 - 1.72)	3.27 (2.95 - 3.59)
Romania	Male	241.45 (137.31 - 322.66)	232.19 (147.44 - 321.91)	-3.84	2.01 (1.14 - 2.71)	2.44 (1.48 - 3.64)	0.48 (0.27 - 0.69)
Russian Federation	Male	2791.13 (1493.23 - 4021.33)	2541.77 (2016.98 - 3483.78)	-8.93	3.78 (2.01 - 5.57)	3.43 (2.72 - 4.7)	-0.39 (-0.71 - -0.08)
Rwanda	Male	59.1 (39.66 - 79.01)	49.18 (33.58 - 71.22)	-16.79	2.12 (1.51 - 2.83)	1.08 (0.75 - 1.48)	-3.31 (-3.72 - -2.9)
Saint Lucia	Male	0.75 (0.62 - 1.12)	1.36 (0.99 - 1.87)	81.33	1.45 (1.21 - 2.17)	1.41 (1.02 - 1.92)	-0.09 (-0.35 - 0.18)
Saint Vincent and the Grenadines	Male	0.29 (0.15 - 0.82)	0.74 (0.55 - 0.96)	155.17	0.67 (0.35 - 1.84)	1.16 (0.87 - 1.53)	1.2 (0.49 - 1.91)
Samoa	Male	0.56 (0.38 - 0.72)	0.51 (0.35 - 0.71)	-8.93	0.89 (0.63 - 1.16)	0.62 (0.43 - 0.82)	-1.46 (-1.58 - -1.34)
Sao Tome and Principe	Male	0.07 (0.05 - 0.13)	0.08 (0.05 - 0.16)	14.29	0.14 (0.1 - 0.26)	0.12 (0.08 - 0.2)	-0.76 (-0.82 - -0.69)
Saudi Arabia	Male	83.27 (29.2 - 133.44)	469.09 (119.01 - 863.64)	463.34	1.2 (0.46 - 1.89)	2.19 (0.57 - 4.08)	2.73 (1.93 - 3.54)
Senegal	Male	23.79 (16.75 - 30.07)	31.75 (22.54 - 44.97)	33.46	0.82 (0.6 - 1.03)	0.64 (0.44 - 0.88)	-0.92 (-0.98 - -0.86)
Serbia	Male	149.79 (86.13 - 206)	165.29 (89.22 - 239.12)	10.35	2.99 (1.7 - 4.22)	3.39 (1.75 - 5.15)	0.88 (0.69 - 1.07)
Seychelles	Male	0.28 (0.22 - 0.41)	0.45 (0.35 - 0.68)	60.71	0.94 (0.74 - 1.35)	0.81 (0.63 - 1.23)	-0.97 (-1.18 - -0.76)
Sierra Leone	Male	13.39 (8.53 - 18.53)	15.42 (10.6 - 20.2)	15.16	0.8 (0.54 - 1.07)	0.55 (0.37 - 0.7)	-1.46 (-1.52 - -1.4)
Singapore	Male	9.11 (6.61 - 12.76)	37.03 (24.74 - 49.15)	306.48	0.59 (0.44 - 0.85)	1.19 (0.78 - 1.6)	3.74 (3.28 - 4.21)
Slovakia	Male	71.46 (50.68 - 93.07)	106.08 (68.1 - 148.62)	48.45	2.67 (1.88 - 3.45)	3.67 (2.31 - 5.32)	1.77 (1.52 - 2.02)
Slovenia	Male	32 (22.48 - 46.88)	38.44 (28.43 - 56.91)	20.13	3.15 (2.18 - 4.71)	4.02 (2.85 - 6.08)	0.86 (0.56 - 1.16)
Solomon Islands	Male	0.85 (0.58 - 1.22)	1.42 (0.89 - 2.12)	67.06	0.72 (0.5 - 1.04)	0.58 (0.37 - 0.88)	-0.47 (-0.63 - -0.32)
Somalia	Male	55.68 (16.27 - 94.28)	127.91 (74.31 - 216.7)	129.72	1.94 (0.79 - 3.22)	1.99 (1.21 - 3.19)	-0.31 (-0.6 - -0.02)
South Africa	Male	73.19 (53.5 - 92.44)	107.95 (73.2 - 134.4)	47.49	0.56 (0.41 - 0.71)	0.46 (0.31 - 0.56)	-0.98 (-1.5 - -0.46)
South Korea	Male	46.45 (35.43 - 84.79)	275.8 (147.2 - 388.07)	493.76	0.26 (0.2 - 0.46)	0.86 (0.47 - 1.22)	5.83 (5.31 - 6.35)
South Sudan	Male	34.96 (12.65 - 59.57)	58.45 (38.37 - 83)	67.19	1.45 (0.68 - 2.37)	1.52 (1.03 - 2.13)	-0.07 (-0.29 - 0.16)
Spain	Male	764.58 (500.07 - 1012.22)	1034.48 (753.63 - 1571.96)	35.30	3.63 (2.29 - 4.83)	4.07 (2.85 - 6.47)	0.69 (0.47 - 0.91)
Sri Lanka	Male	102.37 (81.43 - 144.7)	137.93 (94.96 - 207.37)	34.74	1.45 (1.13 - 2)	1.26 (0.86 - 1.93)	-0.84 (-1.1 - -0.58)
Sudan	Male	111.93 (66.63 - 182.97)	145.99 (99.32 - 213.55)	30.43	1.5 (0.92 - 2.48)	0.96 (0.63 - 1.43)	-1.61 (-1.66 - -1.56)
Suriname	Male	1.72 (1.37 - 2.66)	3.02 (2.19 - 3.91)	75.58	1.02 (0.83 - 1.55)	1.05 (0.77 - 1.36)	0.14 (-0.19 - 0.48)
Swaziland	Male	1.93 (1.48 - 2.65)	3.29 (2.12 - 4.5)	70.47	0.93 (0.72 - 1.3)	0.9 (0.59 - 1.22)	0.66 (0.01 - 1.32)
Sweden	Male	109.98 (84.13 - 164)	148.02 (113.27 - 215.44)	34.59	2.25 (1.69 - 3.5)	2.62 (1.94 - 3.88)	0.5 (0.29 - 0.72)
Switzerland	Male	143.27 (87.31 - 211.59)	145.57 (103.81 - 262.72)	1.61	3.79 (2.22 - 5.62)	2.93 (2.02 - 5.62)	-1.05 (-1.23 - -0.87)
Syria	Male	9.18 (6.79 - 12.14)	17.94 (10.65 - 25.92)	95.42	0.22 (0.15 - 0.28)	0.22 (0.13 - 0.32)	-0.09 (-0.4 - 0.23)
Tajikistan	Male	6.37 (4.92 - 9.53)	11.62 (8.98 - 16.32)	82.42	0.37 (0.25 - 0.5)	0.33 (0.23 - 0.42)	-0.76 (-0.99 - -0.53)
Tanzania	Male	138.34 (57.27 - 211.01)	245.86 (166.81 - 369.6)	77.72	1.35 (0.68 - 2.07)	1.15 (0.8 - 1.67)	-0.74 (-0.87 - -0.62)
Thailand	Male	217.65 (171.66 - 327.81)	295.16 (200.07 - 565)	35.61	0.91 (0.73 - 1.32)	0.77 (0.49 - 1.59)	-1.39 (-1.69 - -1.09)
The Bahamas	Male	0.95 (0.71 - 1.23)	1.66 (1.13 - 2.44)	74.74	0.88 (0.68 - 1.14)	0.87 (0.59 - 1.28)	-0.21 (-0.4 - -0.02)
The Gambia	Male	3.28 (2.21 - 4.63)	5.22 (3.48 - 8.23)	59.15	0.89 (0.62 - 1.25)	0.68 (0.48 - 1.06)	-0.7 (-0.81 - -0.59)
Timor-Leste	Male	3.82 (2.46 - 5.75)	4.25 (2.26 - 7.27)	11.26	1.32 (0.79 - 2.19)	0.83 (0.45 - 1.42)	-1.85 (-1.99 - -1.71)
Togo	Male	9.79 (6.87 - 12.71)	14.07 (9.85 - 19)	43.72	0.76 (0.55 - 0.99)	0.58 (0.39 - 0.76)	-1.09 (-1.27 - -0.91)
Tonga	Male	0.12 (0.08 - 0.17)	0.16 (0.11 - 0.24)	33.33	0.34 (0.24 - 0.49)	0.36 (0.24 - 0.53)	0.42 (0.3 - 0.55)
Trinidad and Tobago	Male	3 (2.33 - 3.97)	5.99 (3.32 - 8.44)	99.67	0.57 (0.45 - 0.76)	0.76 (0.42 - 1.07)	1.72 (1.26 - 2.19)
Tunisia	Male	53.87 (39 - 75.03)	108.94 (63.31 - 163.2)	102.23	1.45 (1.08 - 2.12)	1.87 (1.07 - 2.81)	1.12 (0.9 - 1.35)
Turkey	Male	301.44 (206.15 - 552.94)	581.51 (389.55 - 957.71)	92.91	1.25 (0.86 - 2.24)	1.41 (0.93 - 2.35)	0.43 (-0.12 - 0.99)
Turkmenistan	Male	14.4 (8.35 - 18.52)	32.85 (16.08 - 47.06)	128.13	0.92 (0.59 - 1.15)	1.26 (0.65 - 1.78)	1.45 (1.12 - 1.78)
Uganda	Male	88.24 (60.43 - 128.85)	186.39 (119.07 - 265.97)	111.23	1.23 (0.85 - 1.95)	1.37 (0.9 - 1.84)	0.29 (0.03 - 0.54)
Ukraine	Male	587.26 (385.39 - 871.6)	1048.25 (734.35 - 1755.96)	78.50	2.28 (1.48 - 3.41)	4.69 (3.17 - 8.16)	2.25 (1.61 - 2.89)
United Arab Emirates	Male	9.41 (5.66 - 16.47)	87.87 (55.31 - 138.92)	833.79	1.01 (0.7 - 1.66)	1.37 (0.84 - 2.25)	1.46 (1.27 - 1.66)
United Kingdom	Male	1705.99 (1135.66 - 1981.68)	1962.37 (1506.39 - 2581.47)	15.03	5.45 (3.59 - 6.37)	5.26 (4.09 - 7)	-0.22 (-0.32 - -0.11)
United States	Male	5087.76 (3172.91 - 5761.91)	6168.23 (4975.53 - 9831.92)	21.24	3.74 (2.35 - 4.24)	3.55 (2.8 - 5.81)	-0.73 (-1.01 - -0.46)
Uruguay	Male	26.91 (18.37 - 31.89)	30.03 (19.69 - 40.02)	11.59	1.62 (1.11 - 1.93)	1.62 (1.06 - 2.19)	0.04 (-0.09 - 0.17)
Uzbekistan	Male	102.99 (75 - 152.65)	187.53 (130.9 - 285.97)	82.09	1.16 (0.83 - 1.95)	1.23 (0.88 - 1.86)	0.36 (0.19 - 0.54)
Vanuatu	Male	0.52 (0.27 - 0.98)	1.08 (0.48 - 2.16)	107.69	0.95 (0.49 - 1.83)	0.96 (0.43 - 1.92)	0.17 (0.03 - 0.32)
Venezuela	Male	82.39 (60.67 - 109.42)	213.95 (119.86 - 345.32)	159.68	1.17 (0.85 - 1.5)	1.39 (0.81 - 2.23)	0.86 (0.68 - 1.04)
Vietnam	Male	253.4 (183.75 - 345.46)	447.35 (245.27 - 626.61)	76.54	1.03 (0.78 - 1.47)	0.96 (0.53 - 1.34)	-0.07 (-0.3 - 0.16)
Virgin Islands, U.S.	Male	0.21 (0.15 - 0.37)	0.54 (0.37 - 0.71)	157.14	0.45 (0.34 - 0.8)	0.89 (0.59 - 1.2)	3.57 (3.01 - 4.14)
Yemen	Male	72.45 (28.04 - 127.7)	121.43 (82.51 - 193.35)	67.61	1.67 (0.79 - 3)	1.15 (0.77 - 1.8)	-1.53 (-1.62 - -1.44)
Zambia	Male	57.56 (31.38 - 81.94)	91.98 (61.14 - 133.27)	59.80	1.84 (1.18 - 2.62)	1.41 (0.96 - 1.93)	-1.48 (-1.91 - -1.04)
Zimbabwe	Male	22.16 (13.47 - 28.93)	58.31 (28.4 - 81.28)	163.13	0.65 (0.46 - 0.91)	1.11 (0.64 - 1.47)	2.99 (1.87 - 4.12)

ASIR, age standardized incidence rate; CI, confidence interval; EAPC, estimated annual percentage change; UI, uncertainty interval.

Supplementary Table S4. The death cases and age-standardized death rate of Hodgkin lymphoma in 1990 and 2017, and its temporal trends from 1990 to 2017.

Nation	Sex	Death Cases No. (95% UI)		Change in absolute number (%)	ASDR per 100,000 No. (95% UI)		1990-2017 EAPC No. (95 CI)
		1990	2017		1990	2017	
Afghanistan	Both	148.04 (59.97 - 234.8)	289.46 (207.81 - 398.93)	95.53	1.93 (0.84 - 3.07)	1.54 (1.1 - 2.17)	-0.91 (-1.27 - -0.56)
Albania	Both	20.68 (16.84 - 34.58)	16 (11.5 - 26.62)	-22.63	0.76 (0.62 - 1.29)	0.45 (0.32 - 0.75)	-2.06 (-2.23 - -1.88)
Algeria	Both	269.87 (199.94 - 327.46)	305.5 (230.61 - 360.62)	13.20	1.39 (1.12 - 1.77)	0.81 (0.62 - 0.95)	-1.82 (-1.96 - -1.69)
American Samoa	Both	0.05 (0.04 - 0.07)	0.06 (0.05 - 0.08)	20.00	0.17 (0.13 - 0.23)	0.14 (0.11 - 0.17)	-0.54 (-0.78 - -0.29)
Andorra	Both	0.3 (0.23 - 0.39)	0.35 (0.28 - 0.47)	16.67	0.48 (0.38 - 0.62)	0.3 (0.24 - 0.41)	-1.72 (-1.96 - -1.49)
Angola	Both	52.23 (33.15 - 73.56)	74.84 (57.51 - 101.15)	43.29	0.81 (0.56 - 1.12)	0.46 (0.34 - 0.63)	-2.12 (-2.33 - -1.91)
Antigua and Barbuda	Both	0.11 (0.09 - 0.16)	0.14 (0.12 - 0.19)	27.27	0.2 (0.17 - 0.29)	0.15 (0.12 - 0.19)	-1.08 (-1.29 - -0.88)
Argentina	Both	255.01 (222.66 - 330.76)	229.7 (189.05 - 328.87)	-9.93	0.77 (0.68 - 1)	0.45 (0.37 - 0.65)	-2.12 (-2.27 - -1.97)
Armenia	Both	4.67 (3.56 - 8.04)	8.05 (4.37 - 9.8)	72.38	0.15 (0.12 - 0.25)	0.21 (0.11 - 0.25)	1.28 (0.53 - 2.03)
Australia	Both	87.31 (67.24 - 98.4)	84.56 (69.5 - 101.33)	-3.15	0.45 (0.35 - 0.51)	0.24 (0.2 - 0.29)	-2.13 (-2.4 - -1.85)
Austria	Both	90.11 (48.98 - 105.04)	36.77 (31.62 - 53.02)	-59.19	0.83 (0.47 - 0.96)	0.25 (0.21 - 0.38)	-4.87 (-5.44 - -4.29)
Azerbaijan	Both	51.44 (35.8 - 63.26)	58 (40.16 - 72.66)	12.75	0.77 (0.56 - 0.96)	0.55 (0.39 - 0.69)	-1.41 (-1.7 - -1.12)
Bahrain	Both	2.99 (1.44 - 4)	3.32 (2.27 - 4.06)	11.04	1.09 (0.47 - 1.47)	0.28 (0.18 - 0.34)	-6.16 (-6.68 - -5.64)
Bangladesh	Both	1060.33 (759.94 - 1613.9)	536.64 (374.54 - 924.86)	-49.39	1.35 (0.97 - 2.12)	0.37 (0.26 - 0.64)	-4.88 (-4.96 - -4.79)
Barbados	Both	1.44 (1.21 - 1.78)	1.33 (1.11 - 1.74)	-7.64	0.51 (0.43 - 0.63)	0.33 (0.28 - 0.43)	-1.8 (-1.88 - -1.73)
Belarus	Both	101.38 (64.76 - 118.16)	64.66 (47.23 - 77.19)	-36.22	0.85 (0.54 - 0.99)	0.5 (0.36 - 0.6)	-1.83 (-2.15 - -1.51)
Belgium	Both	91.63 (67.76 - 102.24)	54.79 (47.3 - 74.05)	-40.21	0.67 (0.5 - 0.74)	0.3 (0.26 - 0.42)	-2.89 (-2.99 - -2.8)
Belize	Both	0.73 (0.59 - 0.94)	1.47 (1.22 - 1.83)	101.37	0.57 (0.47 - 0.75)	0.45 (0.38 - 0.56)	-1.24 (-1.51 - -0.96)
Benin	Both	17.24 (12.99 - 21.48)	25.83 (20.18 - 33.26)	49.83	0.52 (0.41 - 0.64)	0.37 (0.28 - 0.46)	-1.39 (-1.49 - -1.3)
Bermuda	Both	0.31 (0.22 - 0.59)	0.44 (0.34 - 0.52)	41.94	0.49 (0.34 - 0.91)	0.42 (0.32 - 0.5)	-1.13 (-1.68 - -0.59)
Bhutan	Both	4.78 (3.34 - 7.24)	3.31 (1.88 - 6.4)	-30.75	1.16 (0.8 - 1.82)	0.41 (0.25 - 0.8)	-3.94 (-4.03 - -3.85)
Bolivia	Both	56.92 (38.09 - 68.18)	58.77 (42.8 - 78.62)	3.25	1.27 (0.89 - 1.53)	0.63 (0.46 - 0.84)	-2.61 (-2.63 - -2.59)
Bosnia and Herzegovina	Both	26.91 (19.82 - 31.02)	21.64 (12.25 - 27.01)	-19.58	0.6 (0.44 - 0.69)	0.42 (0.24 - 0.52)	-1.59 (-1.85 - -1.34)
Botswana	Both	3.55 (2.61 - 5.04)	4.11 (3.36 - 5.52)	15.77	0.45 (0.34 - 0.63)	0.24 (0.2 - 0.32)	-1.99 (-2.24 - -1.74)
Brazil	Both	690.7 (575.41 - 848.76)	723.53 (589.01 - 897)	4.75	0.6 (0.49 - 0.73)	0.32 (0.26 - 0.39)	-2.32 (-2.38 - -2.25)
Brunei	Both	0.66 (0.43 - 1.21)	1.92 (1.44 - 2.26)	190.91	0.45 (0.29 - 0.82)	0.52 (0.39 - 0.6)	1.55 (1.1 - 2)
Bulgaria	Both	95.58 (84.2 - 134.84)	63.97 (54.92 - 83.65)	-33.07	0.87 (0.77 - 1.18)	0.59 (0.5 - 0.73)	-1.71 (-1.87 - -1.56)
Burkina Faso	Both	38.25 (29.4 - 48.58)	51.46 (38.71 - 64.5)	34.54	0.57 (0.44 - 0.75)	0.39 (0.28 - 0.47)	-1.69 (-1.93 - -1.44)
Burundi	Both	54.52 (36.4 - 69.64)	61.04 (43.4 - 76.75)	11.96	1.36 (0.95 - 1.71)	0.83 (0.59 - 1.02)	-2.16 (-2.32 - -2)
Cambodia	Both	68.02 (48.8 - 85.76)	69.34 (52.19 - 92.42)	1.94	1.05 (0.76 - 1.37)	0.53 (0.4 - 0.7)	-2.65 (-2.74 - -2.56)
Cameroon	Both	35.43 (26.1 - 42.5)	66.64 (48.14 - 83.93)	88.09	0.5 (0.38 - 0.6)	0.39 (0.27 - 0.48)	-1.11 (-1.31 - -0.9)
Canada	Both	176.21 (142.06 - 210.96)	154.34 (133.68 - 211.36)	-12.41	0.56 (0.44 - 0.66)	0.29 (0.25 - 0.4)	-2.32 (-2.86 - -1.79)
Cape Verde	Both	0.29 (0.19 - 0.36)	0.41 (0.24 - 0.51)	41.38	0.11 (0.07 - 0.14)	0.08 (0.05 - 0.11)	-1.2 (-1.36 - -1.03)
Central African Republic	Both	14.69 (9.92 - 20.46)	24.05 (18.3 - 32.7)	63.72	0.86 (0.63 - 1.17)	0.79 (0.59 - 1.08)	-0.5 (-0.59 - -0.41)
Chad	Both	20.64 (15.57 - 25.79)	38.78 (29.78 - 49.05)	87.89	0.48 (0.38 - 0.61)	0.45 (0.33 - 0.56)	-0.22 (-0.43 - -0.01)
Chile	Both	74.58 (61.55 - 91.25)	68.77 (58.02 - 87.09)	-7.79	0.65 (0.55 - 0.79)	0.32 (0.27 - 0.4)	-2.48 (-2.74 - -2.22)
China	Both	5809.17 (3311.22 - 7097.77)	2742.23 (1878.83 - 3131.05)	-52.79	0.58 (0.33 - 0.71)	0.15 (0.11 - 0.17)	-5.67 (-6.14 - -5.2)
Colombia	Both	174.46 (143.5 - 221.84)	188.41 (158.66 - 251.72)	8.00	0.73 (0.61 - 0.93)	0.36 (0.3 - 0.48)	-3.02 (-3.22 - -2.82)
Comoros	Both	4.49 (3.51 - 5.81)	4.82 (3.59 - 6.46)	7.35	1.28 (1.01 - 1.66)	0.8 (0.6 - 1.07)	-1.84 (-1.97 - -1.71)
Congo	Both	11.53 (8.79 - 15.47)	17 (12.23 - 23.37)	47.44	0.75 (0.6 - 0.99)	0.51 (0.36 - 0.71)	-1.71 (-1.92 - -1.5)
Costa Rica	Both	29.19 (15.79 - 34.7)	34.79 (24.83 - 41.27)	19.18	1.26 (0.75 - 1.46)	0.71 (0.51 - 0.84)	-2.31 (-2.56 - -2.06)
Cote d'Ivoire	Both	42.95 (30.18 - 53.05)	76.36 (53.73 - 101.21)	77.79	0.54 (0.41 - 0.65)	0.44 (0.33 - 0.58)	-0.91 (-1.08 - -0.75)
Croatia	Both	48.3 (34.84 - 54.05)	24.36 (20.37 - 29.54)	-49.57	0.8 (0.58 - 0.88)	0.34 (0.29 - 0.42)	-2.67 (-2.9 - -2.45)
Cuba	Both	17.62 (11.66 - 39.21)	95.46 (27.86 - 131.66)	441.77	0.16 (0.11 - 0.36)	0.59 (0.17 - 0.81)	5.21 (3.18 - 7.29)
Cyprus	Both	5.98 (3.77 - 7.68)	4.98 (3.78 - 6.06)	-16.72	0.71 (0.45 - 0.91)	0.29 (0.22 - 0.35)	-3.67 (-4.05 - -3.29)
Czech Republic	Both	169.85 (126.9 - 185.58)	80.74 (69.2 - 108.94)	-52.46	1.32 (1.01 - 1.44)	0.46 (0.39 - 0.66)	-3.9 (-4.05 - -3.74)
Democratic Republic of the Congo	Both	142.42 (104.36 - 197.36)	237.99 (182.25 - 331.74)	67.10	0.61 (0.43 - 0.86)	0.49 (0.36 - 0.69)	-0.79 (-0.85 - -0.72)
Denmark	Both	42.1 (31.7 - 49.18)	33.32 (25.17 - 39.06)	-20.86	0.59 (0.45 - 0.7)	0.35 (0.27 - 0.43)	-2.06 (-2.2 - -1.91)
Djibouti	Both	3.58 (2.24 - 4.99)	6.52 (4.38 - 9.64)	82.12	1.03 (0.71 - 1.42)	0.75 (0.51 - 1.09)	-1.39 (-1.62 - -1.17)
Dominica	Both	0.48 (0.39 - 0.68)	0.41 (0.34 - 0.55)	-14.58	0.67 (0.55 - 0.95)	0.5 (0.42 - 0.67)	-1.29 (-1.48 - -1.1)
Dominican Republic	Both	7.21 (5.14 - 13.8)	23.08 (9.52 - 33.58)	220.11	0.14 (0.1 - 0.27)	0.24 (0.1 - 0.35)	1.03 (0.26 - 1.8)
Ecuador	Both	56.96 (49.03 - 79)	72.78 (61.86 - 100.27)	27.77	0.76 (0.67 - 1.04)	0.47 (0.4 - 0.64)	-1.4 (-1.68 - -1.12)
Egypt	Both	773.24 (578.14 - 989.3)	880.71 (584.87 - 1093.04)	13.90	1.91 (1.48 - 2.48)	1.17 (0.79 - 1.44)	-1.56 (-1.7 - -1.42)
El Salvador	Both	38.9 (29.79 - 48.56)	29.62 (22.32 - 41.62)	-23.86	1.06 (0.76 - 1.35)	0.51 (0.39 - 0.7)	-2.97 (-3.13 - -2.81)
Equatorial Guinea	Both	2.45 (1.8 - 3.47)	1.96 (1.26 - 2.93)	-20.00	0.89 (0.68 - 1.2)	0.26 (0.17 - 0.4)	-5.13 (-5.56 - -4.7)
Eritrea	Both	35.59 (18.5 - 50.41)	58.54 (38.8 - 86.34)	64.48	1.83 (1.17 - 2.47)	1.34 (0.9 - 1.92)	-1.48 (-1.75 - -1.21)
Estonia	Both	15.19 (9.64 - 17.47)	6.73 (4.61 - 8.39)	-55.69	0.83 (0.54 - 0.94)	0.34 (0.24 - 0.46)	-3.83 (-4.19 - -3.47)
Ethiopia	Both	892.43 (633.26 - 1156.54)	871.23 (545.78 - 1433.67)	-2.38	2.53 (1.8 - 3.38)	1.25 (0.78 - 2.03)	-2.86 (-3.03 - -2.69)
Federated States of Micronesia	Both	0.3 (0.22 - 0.41)	0.26 (0.17 - 0.35)	-13.33	0.46 (0.33 - 0.64)	0.32 (0.21 - 0.43)	-1.41 (-1.49 - -1.33)
Fiji	Both	1.32 (1.01 - 1.63)	2.17 (1.22 - 2.86)	64.39	0.25 (0.2 - 0.31)	0.26 (0.15 - 0.34)	0.68 (0.42 - 0.94)
Finland	Both	38.88 (29.91 - 43.44)	26.69 (22.2 - 32.93)	-31.35	0.6 (0.47 - 0.68)	0.29 (0.25 - 0.37)	-2.58 (-2.88 - -2.27)
France	Both	457.2 (395.12 - 584.71)	323.32 (278.91 - 448.17)	-29.28	0.63 (0.54 - 0.8)	0.31 (0.27 - 0.44)	-2.42 (-2.55 - -2.3)
Gabon	Both	3.83 (2.99 - 5.14)	5.05 (3.77 - 7)	31.85	0.55 (0.43 - 0.72)	0.4 (0.29 - 0.55)	-1.13 (-1.39 - -0.87)
Georgia	Both	85.13 (53.94 - 100.12)	46.48 (37.22 - 57.55)	-45.40	1.39 (0.88 - 1.63)	0.91 (0.75 - 1.16)	-2.03 (-2.3 - -1.77)
Germany	Both	845.76 (533.01 - 943.83)	431.7 (364.44 - 572.68)	-48.96	0.75 (0.48 - 0.83)	0.28 (0.24 - 0.4)	-3.67 (-4.11 - -3.22)
Ghana	Both	14.82 (11.61 - 21.23)	24.62 (19.77 - 34.25)	66.13	0.16 (0.13 - 0.21)	0.13 (0.1 - 0.16)	-0.62 (-0.71 - -0.52)
Greece	Both	156.75 (137.39 - 202.27)	177.44 (124.16 - 204.23)	13.20	1.1 (0.99 - 1.47)	0.88 (0.68 - 1.01)	-0.82 (-1.32 - -0.32)
Greenland	Both	0.31 (0.16 - 0.4)	0.17 (0.12 - 0.2)	-45.16	0.68 (0.32 - 0.88)	0.26 (0.18 - 0.3)	-4.02 (-4.18 - -3.86)
Grenada	Both	0.39 (0.32 - 0.47)	0.34 (0.28 - 0.48)	-12.82	0.53 (0.44 - 0.65)	0.25 (0.21 - 0.37)	-2.43 (-2.67 - -2.18)
Guam	Both	0.21 (0.16 - 0.31)	0.32 (0.26 - 0.42)	52.38	0.19 (0.15 - 0.3)	0.18 (0.15 - 0.24)	0.33 (-0.1 - 0.75)
Guatemala	Both	40.53 (34.05 - 55.98)	47.32 (39.35 - 64.26)	16.75	0.76 (0.66 - 1.01)	0.38 (0.31 - 0.49)	-2.67 (-2.98 - -2.37)
Guinea	Both	39.38 (27.37 - 48.53)	52.01 (36.39 - 64.73)	32.07	0.78 (0.59 - 0.96)	0.63 (0.46 - 0.78)	-0.7 (-0.81 - -0.58)
Guinea-Bissau	Both	5.06 (3.52 - 6.55)	5.95 (4.65 - 7.78)	17.59	0.75 (0.55 - 0.96)	0.52 (0.41 - 0.65)	-1.35 (-1.39 - -1.31)
Guyana	Both	1.24 (0.69 - 3.14)	2.65 (1.95 - 3.16)	113.71	0.23 (0.13 - 0.57)	0.38 (0.29 - 0.46)	1.97 (0.85 - 3.1)
Haiti	Both	57.29 (43.29 - 85.26)	82.22 (52.23 - 123.52)	43.52	1.33 (0.96 - 2.03)	0.98 (0.62 - 1.49)	-1.07 (-1.15 - -0.99)
Honduras	Both	6.75 (4.43 - 8.41)	10.09 (5.92 - 14.11)	49.48	0.24 (0.14 - 0.31)	0.15 (0.08 - 0.22)	-1.59 (-1.62 - -1.56)
Hungary	Both	133.65 (97.16 - 147.85)	51.08 (43.61 - 72.28)	-61.78	1.01 (0.73 - 1.11)	0.33 (0.28 - 0.46)	-4.26 (-4.38 - -4.14)
Iceland	Both	1.22 (1.03 - 1.46)	0.99 (0.86 - 1.43)	-18.85	0.44 (0.37 - 0.53)	0.21 (0.18 - 0.31)	-2.44 (-2.62 - -2.26)
India	Both	5797.83 (3796.93 - 7032.01)	5152.56 (4216.34 - 6476.57)	-11.13	0.84 (0.56 - 0.98)	0.42 (0.34 - 0.53)	-2.66 (-2.78 - -2.54)
Indonesia	Both	874.85 (636.17 - 1107.85)	934.43 (713.87 - 1217.57)	6.81	0.63 (0.46 - 0.81)	0.4 (0.31 - 0.52)	-1.57 (-1.71 - -1.44)
Iran	Both	167.66 (129.41 - 279.41)	279.46 (186.76 - 316.75)	66.68	0.43 (0.34 - 0.7)	0.37 (0.24 - 0.42)	0.6 (0.02 - 1.18)
Iraq	Both	84.84 (43.86 - 115.17)	67.76 (55.09 - 100.96)	-20.13	0.72 (0.4 - 0.96)	0.21 (0.17 - 0.31)	-4.99 (-5.92 - -4.04)

Ireland	Both	27.98 (21.69 - 31.55)	20.8 (15.9 - 26.24)	-25.66	0.71 (0.55 - 0.8)	0.32 (0.25 - 0.41)	-2.84 (-3 - -2.68)
Israel	Both	31.72 (27.77 - 40.02)	38.04 (32.22 - 51.67)	19.92	0.65 (0.57 - 0.82)	0.38 (0.32 - 0.51)	-2.38 (-2.62 - -2.15)
Italy	Both	632.65 (514.26 - 734.88)	445.46 (358.83 - 573.84)	-29.59	0.82 (0.66 - 0.94)	0.41 (0.33 - 0.53)	-2.23 (-2.5 - -1.97)
Jamaica	Both	6.45 (3.42 - 7.81)	7.41 (4.84 - 9.71)	14.88	0.32 (0.18 - 0.39)	0.25 (0.17 - 0.33)	-0.82 (-1.3 - -0.34)
Japan	Both	175.21 (149.23 - 226.72)	228.34 (163.72 - 251.59)	30.32	0.11 (0.09 - 0.14)	0.08 (0.06 - 0.09)	-0.48 (-0.65 - -0.31)
Jordan	Both	5.64 (3.42 - 7.19)	7.94 (5.67 - 9.55)	40.78	0.24 (0.14 - 0.31)	0.11 (0.07 - 0.13)	-3.35 (-3.51 - -3.19)
Kazakhstan	Both	95.16 (72.66 - 126.71)	75.45 (64.38 - 93.72)	-20.71	0.62 (0.47 - 0.83)	0.41 (0.35 - 0.52)	-1.79 (-2.32 - -1.26)
Kenya	Both	74.03 (58.76 - 98.83)	160.3 (117.52 - 188.08)	116.53	0.49 (0.4 - 0.66)	0.47 (0.34 - 0.55)	-0.03 (-0.27 - 0.21)
Kiribati	Both	0.12 (0.1 - 0.16)	0.19 (0.15 - 0.25)	58.33	0.22 (0.19 - 0.32)	0.21 (0.17 - 0.27)	-0.31 (-0.45 - -0.16)
Kuwait	Both	3.94 (2.73 - 4.51)	4.74 (3.95 - 6.04)	20.30	0.33 (0.24 - 0.37)	0.14 (0.12 - 0.18)	-2.5 (-3.06 - -1.94)
Kyrgyzstan	Both	19.29 (11.71 - 22.81)	12.69 (10.6 - 16.87)	-34.21	0.53 (0.32 - 0.63)	0.24 (0.2 - 0.31)	-2.5 (-2.79 - -2.2)
Laos	Both	33.75 (22.94 - 42.31)	29.87 (21.18 - 42.15)	-11.50	1.13 (0.81 - 1.49)	0.56 (0.39 - 0.81)	-2.69 (-2.83 - -2.54)
Latvia	Both	22.72 (14.56 - 26.42)	12.35 (7.53 - 15.15)	-45.64	0.71 (0.46 - 0.82)	0.43 (0.26 - 0.53)	-2.07 (-2.31 - -1.84)
Lebanon	Both	64.7 (49.55 - 82.3)	73.88 (52.56 - 86.97)	14.19	2.15 (1.68 - 2.76)	1 (0.72 - 1.17)	-2.93 (-3.13 - -2.73)
Lesotho	Both	6.7 (5.44 - 9.18)	9.08 (6.77 - 11.97)	35.52	0.55 (0.45 - 0.75)	0.62 (0.46 - 0.8)	1.05 (0.57 - 1.53)
Liberia	Both	7.81 (5.79 - 9.55)	9.56 (7.36 - 12.36)	22.41	0.49 (0.38 - 0.61)	0.33 (0.25 - 0.42)	-1.8 (-2.1 - -1.51)
Libya	Both	52.32 (39.43 - 67.38)	83.42 (59.29 - 105.41)	59.44	1.83 (1.42 - 2.43)	1.38 (1 - 1.75)	-1.14 (-1.32 - -0.95)
Lithuania	Both	32.46 (22.7 - 36.82)	16.64 (13.49 - 19.95)	-48.74	0.77 (0.54 - 0.87)	0.39 (0.33 - 0.49)	-3.07 (-3.45 - -2.69)
Luxembourg	Both	2.8 (2.18 - 3.4)	2.2 (1.74 - 3.36)	-21.43	0.55 (0.43 - 0.68)	0.26 (0.2 - 0.41)	-3.01 (-3.24 - -2.78)
Macedonia	Both	15.35 (9.53 - 18.97)	14.22 (9.64 - 17.23)	-7.36	0.75 (0.48 - 0.92)	0.49 (0.32 - 0.59)	-2.29 (-2.65 - -1.93)
Madagascar	Both	116.32 (91.37 - 145.14)	142.48 (104.1 - 182.56)	22.49	1.17 (0.94 - 1.46)	0.77 (0.56 - 0.97)	-1.69 (-1.9 - -1.47)
Malawi	Both	44.03 (16.5 - 63.72)	58.17 (44.52 - 74.31)	32.11	0.62 (0.3 - 0.85)	0.46 (0.36 - 0.57)	-1.68 (-2.04 - -1.33)
Malaysia	Both	69.61 (41.36 - 91.99)	81.26 (41.17 - 108.28)	16.74	0.55 (0.34 - 0.72)	0.28 (0.14 - 0.38)	-2.5 (-2.73 - -2.26)
Maldives	Both	0.41 (0.3 - 0.52)	0.31 (0.24 - 0.4)	-24.39	0.32 (0.23 - 0.4)	0.09 (0.07 - 0.11)	-5.15 (-5.32 - -4.98)
Mali	Both	70.44 (47.07 - 87.27)	80.49 (59.43 - 101.72)	14.27	1.03 (0.78 - 1.25)	0.54 (0.43 - 0.71)	-2.37 (-2.59 - -2.15)
Malta	Both	3.53 (3 - 4.29)	3.45 (3.02 - 4.51)	-2.27	0.84 (0.72 - 1.04)	0.48 (0.42 - 0.63)	-2.07 (-2.19 - -1.95)
Marshall Islands	Both	0.11 (0.08 - 0.14)	0.16 (0.1 - 0.2)	45.45	0.44 (0.33 - 0.53)	0.37 (0.24 - 0.45)	-0.54 (-0.72 - -0.36)
Mauritania	Both	6.66 (5.08 - 8.15)	7.39 (5.62 - 10.25)	10.96	0.46 (0.37 - 0.57)	0.28 (0.21 - 0.37)	-1.82 (-1.9 - -1.75)
Mauritius	Both	3.32 (2.44 - 3.74)	3.9 (2.51 - 4.53)	17.47	0.35 (0.26 - 0.4)	0.25 (0.16 - 0.29)	-1.69 (-2.06 - -1.31)
Mexico	Both	513.26 (404.99 - 581.51)	589.77 (496.11 - 746.69)	14.91	0.89 (0.71 - 1.01)	0.5 (0.42 - 0.63)	-2.14 (-2.39 - -1.89)
Moldova	Both	52.23 (39.96 - 69.53)	30.46 (26.58 - 39.67)	-41.68	1.15 (0.88 - 1.51)	0.64 (0.55 - 0.84)	-2.61 (-2.89 - -2.33)
Mongolia	Both	3.63 (2.97 - 4.97)	4.55 (3.59 - 5.56)	25.34	0.28 (0.21 - 0.35)	0.16 (0.12 - 0.2)	-2.53 (-2.9 - -2.17)
Montenegro	Both	7.9 (6.6 - 10.46)	6.81 (5.65 - 9.34)	-13.80	1.22 (1.03 - 1.62)	0.78 (0.65 - 1.07)	-2.08 (-2.4 - -1.76)
Morocco	Both	348.58 (247.08 - 416.04)	369.68 (269.25 - 461.94)	6.05	1.7 (1.29 - 2.01)	1.08 (0.8 - 1.34)	-1.64 (-1.72 - -1.56)
Mozambique	Both	166.14 (130.36 - 216.65)	212.14 (151.17 - 271.66)	27.69	1.51 (1.24 - 2)	1.01 (0.7 - 1.29)	-1.45 (-1.67 - -1.23)
Myanmar	Both	385.92 (269.54 - 490)	282.52 (214.18 - 367.13)	-26.79	1.24 (0.89 - 1.62)	0.58 (0.44 - 0.75)	-2.92 (-3.18 - -2.65)
Namibia	Both	8.82 (6.42 - 10.57)	9.46 (6.69 - 12.07)	7.26	0.91 (0.72 - 1.13)	0.52 (0.39 - 0.65)	-2.3 (-2.8 - -1.79)
Nepal	Both	152.89 (108.39 - 230.27)	119.05 (73.08 - 213.35)	-22.13	1.09 (0.78 - 1.64)	0.47 (0.3 - 0.85)	-3.11 (-3.39 - -2.83)
Netherlands	Both	113.65 (86.72 - 124.18)	90.07 (71.35 - 108.04)	-20.75	0.6 (0.46 - 0.66)	0.32 (0.26 - 0.39)	-2.32 (-2.45 - -2.2)
New Zealand	Both	16.95 (10.79 - 19.47)	15.97 (9.73 - 18.76)	-5.78	0.43 (0.28 - 0.49)	0.25 (0.16 - 0.29)	-1.77 (-2.01 - -1.53)
Nicaragua	Both	9.95 (7.02 - 11.81)	12.79 (8.06 - 15.71)	28.54	0.41 (0.3 - 0.5)	0.25 (0.16 - 0.31)	-1.79 (-1.91 - -1.66)
Niger	Both	34.88 (23.85 - 45.43)	45.13 (34.87 - 58.9)	29.39	0.61 (0.46 - 0.79)	0.38 (0.29 - 0.48)	-2.13 (-2.3 - -1.96)
Nigeria	Both	1808.82 (1181.13 - 2536.44)	2516.58 (1731.32 - 3547.13)	39.13	2.55 (1.78 - 3.49)	1.71 (1.22 - 2.42)	-1.82 (-1.96 - -1.67)
North Korea	Both	53.99 (35.58 - 70.99)	81.23 (55.83 - 100.83)	50.45	0.29 (0.19 - 0.38)	0.27 (0.18 - 0.34)	-0.21 (-0.45 - 0.03)
Northern Mariana Islands	Both	0.04 (0.03 - 0.06)	0.05 (0.04 - 0.06)	25.00	0.13 (0.1 - 0.19)	0.11 (0.08 - 0.13)	-0.45 (-0.76 - -0.14)
Norway	Both	27.68 (24.08 - 35.19)	18.91 (17.05 - 25.9)	-31.68	0.47 (0.41 - 0.61)	0.24 (0.21 - 0.34)	-2.42 (-2.57 - -2.27)
Oman	Both	11.63 (6.96 - 15.66)	14.19 (8.45 - 18.65)	22.01	0.97 (0.61 - 1.28)	0.43 (0.25 - 0.56)	-2.92 (-3.31 - -2.52)
Pakistan	Both	1633.42 (1221.92 - 2117.89)	2724.87 (2077.89 - 3587.55)	66.82	1.86 (1.42 - 2.5)	1.53 (1.19 - 2.04)	-0.91 (-1.11 - -0.71)
Palestine	Both	6.28 (3.65 - 12.29)	13.91 (11.23 - 22.67)	121.50	0.5 (0.31 - 0.95)	0.42 (0.35 - 0.63)	-0.64 (-0.91 - -0.37)
Panama	Both	5.31 (4.49 - 6.6)	10.04 (6.72 - 11.69)	89.08	0.29 (0.25 - 0.37)	0.26 (0.17 - 0.3)	-0.04 (-0.43 - 0.36)
Papua New Guinea	Both	17.15 (13.32 - 22.02)	32.64 (22.9 - 41.56)	90.32	0.6 (0.47 - 0.78)	0.49 (0.35 - 0.63)	-0.51 (-0.64 - -0.37)
Paraguay	Both	14.2 (10.73 - 19.17)	23.48 (16.33 - 29.6)	65.35	0.49 (0.38 - 0.65)	0.4 (0.27 - 0.5)	-0.54 (-0.69 - -0.39)
Peru	Both	67.84 (55.66 - 92.23)	69.4 (46.22 - 85.98)	2.30	0.43 (0.33 - 0.56)	0.22 (0.15 - 0.28)	-2.38 (-2.5 - -2.25)
Philippines	Both	150.95 (128.08 - 216.16)	167.39 (116.28 - 323.76)	10.89	0.34 (0.29 - 0.47)	0.2 (0.14 - 0.37)	-2.01 (-2.18 - -1.84)
Poland	Both	519.92 (380.84 - 567.82)	259.87 (219.73 - 329.24)	-50.02	1.19 (0.88 - 1.3)	0.45 (0.39 - 0.58)	-3.96 (-4.17 - -3.75)
Portugal	Both	80.86 (70.15 - 117.77)	58.08 (48.42 - 85.13)	-28.17	0.65 (0.57 - 0.95)	0.31 (0.26 - 0.46)	-3.03 (-3.17 - -2.89)
Puerto Rico	Both	24.57 (17.64 - 28.23)	21.03 (14.39 - 24.29)	-14.41	0.66 (0.48 - 0.76)	0.39 (0.27 - 0.45)	-2.35 (-2.64 - -2.05)
Qatar	Both	0.55 (0.4 - 0.91)	2.58 (1.53 - 4.04)	369.09	0.24 (0.19 - 0.37)	0.14 (0.09 - 0.2)	-2.05 (-2.33 - -1.77)
Romania	Both	222.86 (155.41 - 246.22)	119.39 (96.08 - 143.36)	-46.43	0.85 (0.59 - 0.94)	0.42 (0.32 - 0.48)	-3.05 (-3.23 - -2.86)
Russian Federation	Both	1396.92 (912.13 - 1628.61)	864.9 (732.86 - 1039.99)	-38.09	0.83 (0.54 - 0.97)	0.45 (0.39 - 0.55)	-2.7 (-3.17 - -2.22)
Rwanda	Both	70.12 (48.6 - 88.9)	60.25 (45.1 - 80.53)	-14.08	1.33 (0.96 - 1.66)	0.65 (0.5 - 0.85)	-3.32 (-3.69 - -2.96)
Saint Lucia	Both	0.69 (0.59 - 0.99)	0.93 (0.75 - 1.2)	34.78	0.65 (0.56 - 0.96)	0.46 (0.37 - 0.59)	-1.26 (-1.48 - -1.03)
Saint Vincent and the Grenadines	Both	0.27 (0.14 - 0.74)	0.72 (0.57 - 0.86)	166.67	0.32 (0.17 - 0.85)	0.56 (0.45 - 0.67)	1.02 (0.19 - 1.85)
Samoa	Both	0.66 (0.47 - 0.8)	0.6 (0.43 - 0.74)	-9.09	0.57 (0.44 - 0.71)	0.38 (0.28 - 0.48)	-1.56 (-1.7 - -1.42)
Sao Tome and Principe	Both	0.08 (0.06 - 0.13)	0.1 (0.07 - 0.16)	25.00	0.09 (0.07 - 0.14)	0.07 (0.05 - 0.11)	-0.62 (-0.69 - -0.56)
Saudi Arabia	Both	78.32 (27.21 - 121.58)	129.01 (35.4 - 198.36)	64.72	0.76 (0.29 - 1.18)	0.49 (0.14 - 0.74)	-0.87 (-1.26 - -0.49)
Senegal	Both	26.07 (19.52 - 31.47)	39.2 (30.01 - 52.72)	50.36	0.5 (0.39 - 0.61)	0.41 (0.31 - 0.53)	-0.73 (-0.76 - -0.69)
Serbia	Both	107.63 (74.35 - 135.64)	76.52 (49.93 - 90.9)	-28.90	0.99 (0.68 - 1.23)	0.58 (0.37 - 0.7)	-1.87 (-2.06 - -1.68)
Seychelles	Both	0.39 (0.3 - 0.6)	0.47 (0.35 - 0.57)	20.51	0.63 (0.49 - 0.95)	0.43 (0.32 - 0.53)	-1.61 (-1.76 - -1.45)
Sierra Leone	Both	14.55 (10.37 - 18.55)	18.92 (14.13 - 23.48)	30.03	0.49 (0.36 - 0.61)	0.37 (0.27 - 0.45)	-1.03 (-1.1 - -0.97)
Singapore	Both	5.69 (4.75 - 7.47)	6.83 (5.21 - 8.08)	20.04	0.21 (0.18 - 0.28)	0.1 (0.08 - 0.12)	-2.04 (-2.3 - -1.77)
Slovakia	Both	51.23 (39.21 - 59.68)	36.79 (29.49 - 44.79)	-28.19	0.88 (0.68 - 1.03)	0.47 (0.38 - 0.57)	-2.11 (-2.2 - -2.02)
Slovenia	Both	17.34 (13.02 - 19.34)	11.61 (8.45 - 13.55)	-33.04	0.73 (0.55 - 0.81)	0.33 (0.25 - 0.4)	-3.17 (-3.27 - -3.07)
Solomon Islands	Both	0.97 (0.73 - 1.3)	1.67 (1.16 - 2.31)	72.16	0.48 (0.36 - 0.65)	0.38 (0.26 - 0.54)	-0.53 (-0.68 - -0.37)
Somalia	Both	62.98 (22.97 - 102.5)	151.35 (90.69 - 238.15)	140.31	1.29 (0.62 - 2)	1.3 (0.82 - 1.98)	-0.33 (-0.57 - -0.09)
South Africa	Both	90.78 (70.44 - 109.54)	122.99 (90.84 - 146.03)	35.48	0.33 (0.26 - 0.39)	0.25 (0.18 - 0.29)	-1.13 (-1.79 - -0.47)
South Korea	Both	40.83 (32.33 - 73.54)	61.41 (36.09 - 75.34)	50.40	0.11 (0.09 - 0.2)	0.08 (0.05 - 0.1)	-0.68 (-0.86 - -0.49)
South Sudan	Both	40.37 (16.34 - 66.63)	64.29 (44.74 - 85.9)	59.25	0.99 (0.53 - 1.54)	0.95 (0.67 - 1.27)	-0.44 (-0.65 - -0.22)
Spain	Both	345.4 (265.65 - 396.51)	246.39 (183.45 - 308.96)	-28.67	0.7 (0.53 - 0.78)	0.31 (0.24 - 0.4)	-2.92 (-3.03 - -2.81)
Sri Lanka	Both	106.79 (89.44 - 138.38)	87.06 (66.09 - 108.73)	-18.48	0.83 (0.67 - 1.05)	0.36 (0.27 - 0.44)	-3.54 (-3.92 - -3.16)
Sudan	Both	129.82 (87.67 - 200.3)	155.66 (116.53 - 215.13)	19.90	0.96 (0.67 - 1.45)	0.59 (0.43 - 0.82)	-1.7 (-1.77 - -1.63)
Suriname	Both	1.94 (1.63 - 2.78)	3.22 (2.6 - 3.96)	65.98	0.61 (0.51 - 0.87)	0.55 (0.44 - 0.67)	-0.26 (-0.62 - 0.11)
Swaziland	Both	2.41 (1.93 - 3.26)	3.86 (2.86 - 5.04)	60.17	0.56 (0.45 - 0.75)	0.5 (0.37 - 0.65)	0.36 (-0.3 - 1.03)
Sweden	Both	48.12 (38.39 - 54.79)	35.75 (27.71 - 41.99)	-25.71	0.37 (0.31 - 0.44)	0.21 (0.18 - 0.26)	-1.84 (-2.01 - -1.68)
Switzerland	Both	37.46 (28.51 - 42.52)	31.7 (23.52 - 39.59)	-15.38	0.41 (0.31 - 0.46)	0.22 (0.17 - 0.3)	-2.08 (-2.17 - -2)
Syria	Both	9.27 (6.47 - 11.13)	12.41 (6.37 - 17.15)	33.87	0.13 (0.08 - 0.16)	0.09 (0.04 - 0.12)	-1.54 (-1.76 - -1.33)
Tajikistan	Both	9.03 (6.9 - 11.24)	14.35 (11.05 - 17.02)	58.91	0.26 (0.18 - 0.34)	0.22 (0.15 - 0.27)	-0.93 (-1.1 - -0.76)
Tanzania	Both	169.78 (86.13 - 232.31)	282.26 (204.09 - 383.81)	66.25	0.91 (0.56 - 1.26)	0.71 (0.52 - 0.93)	-1.19 (-1.35 - -1.04)

Thailand	Both	213.27 (171.58 - 313.28)	174.66 (137.9 - 260.71)	-18.10	0.46 (0.38 - 0.67)	0.19 (0.15 - 0.29)	-3.78 (-3.99 - -3.56)
The Bahamas	Both	1.03 (0.86 - 1.24)	1.35 (1.12 - 1.75)	31.07	0.49 (0.41 - 0.59)	0.34 (0.29 - 0.44)	-1.6 (-1.79 - -1.4)
The Gambia	Both	3.5 (2.52 - 4.62)	6.2 (4.67 - 8.67)	77.14	0.54 (0.41 - 0.71)	0.43 (0.33 - 0.6)	-0.66 (-0.77 - -0.56)
Timor-Leste	Both	4.26 (2.91 - 6.11)	4.89 (2.87 - 7.63)	14.79	0.86 (0.56 - 1.3)	0.51 (0.31 - 0.8)	-2.08 (-2.19 - -1.98)
Togo	Both	11.44 (8.73 - 13.91)	17.48 (13.33 - 22.35)	52.80	0.48 (0.38 - 0.6)	0.35 (0.26 - 0.44)	-1.29 (-1.46 - -1.13)
Tonga	Both	0.15 (0.11 - 0.2)	0.17 (0.12 - 0.23)	13.33	0.22 (0.17 - 0.31)	0.2 (0.14 - 0.27)	-0.29 (-0.37 - -0.21)
Trinidad and Tobago	Both	2.96 (2.47 - 3.72)	4.88 (2.8 - 6.48)	64.86	0.29 (0.25 - 0.36)	0.3 (0.17 - 0.39)	0.61 (0.24 - 0.98)
Tunisia	Both	66.8 (53.81 - 88.89)	75.48 (56.69 - 96.39)	12.99	1.02 (0.84 - 1.4)	0.63 (0.47 - 0.8)	-1.77 (-1.9 - -1.63)
Turkey	Both	336.82 (241.27 - 562.26)	254.75 (199.85 - 382.62)	-24.37	0.74 (0.52 - 1.23)	0.3 (0.23 - 0.44)	-3.58 (-3.82 - -3.34)
Turkmenistan	Both	17.06 (12.31 - 19.79)	21.84 (15.19 - 25.52)	28.02	0.58 (0.45 - 0.66)	0.46 (0.33 - 0.53)	-0.57 (-0.98 - -0.16)
Uganda	Both	116.47 (88.02 - 152.32)	219.47 (157.26 - 295.39)	88.43	0.95 (0.73 - 1.32)	0.84 (0.61 - 1.1)	-0.71 (-0.88 - -0.53)
Ukraine	Both	466.22 (353.99 - 598.07)	482.16 (400.15 - 667.75)	3.42	0.76 (0.57 - 0.96)	0.88 (0.71 - 1.27)	-0.17 (-0.67 - 0.33)
United Arab Emirates	Both	7.39 (5.02 - 11.95)	33.59 (22.9 - 50.99)	354.53	0.64 (0.47 - 1)	0.46 (0.3 - 0.74)	-1.14 (-1.23 - -1.05)
United Kingdom	Both	501.21 (401.64 - 580.43)	357.47 (283.75 - 411.31)	-28.68	0.65 (0.51 - 0.73)	0.35 (0.28 - 0.41)	-2.26 (-2.43 - -2.08)
United States	Both	1655.11 (1244.81 - 1766.42)	1357.42 (1225.08 - 1834.6)	-17.99	0.55 (0.41 - 0.59)	0.3 (0.27 - 0.42)	-2.36 (-2.48 - -2.23)
Uruguay	Both	30.68 (22.39 - 35)	23.05 (17.44 - 27.62)	-24.87	0.84 (0.61 - 0.96)	0.51 (0.38 - 0.61)	-1.66 (-1.84 - -1.47)
Uzbekistan	Both	89.57 (69.25 - 131.58)	128.91 (107.92 - 170.75)	43.92	0.56 (0.41 - 0.88)	0.46 (0.38 - 0.63)	-0.69 (-0.84 - -0.54)
Vanuatu	Both	0.62 (0.32 - 1.11)	1.35 (0.61 - 2.5)	117.74	0.64 (0.33 - 1.14)	0.64 (0.29 - 1.18)	0.09 (-0.06 - 0.23)
Venezuela	Both	90.41 (69.65 - 108.02)	151.36 (106.52 - 184.29)	67.42	0.71 (0.55 - 0.84)	0.51 (0.37 - 0.62)	-1.06 (-1.2 - -0.92)
Vietnam	Both	303.84 (232.38 - 391.56)	346.75 (214.16 - 435.42)	14.12	0.59 (0.47 - 0.8)	0.36 (0.22 - 0.45)	-1.66 (-1.77 - -1.56)
Virgin Islands, U.S.	Both	0.2 (0.16 - 0.34)	0.37 (0.27 - 0.46)	85.00	0.22 (0.17 - 0.36)	0.23 (0.17 - 0.28)	0.66 (0.24 - 1.07)
Yemen	Both	81.06 (37.87 - 127.94)	135.19 (98.72 - 194)	66.78	1.03 (0.55 - 1.66)	0.71 (0.51 - 1.05)	-1.48 (-1.59 - -1.36)
Zambia	Both	70.61 (43.69 - 90.14)	104.97 (75.98 - 140.69)	48.66	1.27 (0.91 - 1.62)	0.87 (0.64 - 1.13)	-1.87 (-2.21 - -1.54)
Zimbabwe	Both	26.01 (18.49 - 31.82)	63.78 (37.31 - 83.4)	145.21	0.4 (0.33 - 0.54)	0.61 (0.4 - 0.75)	2.58 (1.54 - 3.62)
Afghanistan	Female	47.36 (19.66 - 78.4)	123.74 (80.05 - 179.95)	161.28	1.29 (0.58 - 2.1)	1.19 (0.8 - 1.65)	-0.34 (-0.61 - -0.07)
Albania	Female	7.98 (6.36 - 11.25)	5.87 (4.03 - 9.23)	-26.44	0.57 (0.46 - 0.82)	0.32 (0.22 - 0.51)	-1.95 (-2.06 - -1.85)
Algeria	Female	110.78 (73.45 - 141.36)	133.1 (93.98 - 169.88)	20.15	1.14 (0.83 - 1.46)	0.7 (0.51 - 0.89)	-1.64 (-1.74 - -1.54)
American Samoa	Female	0.03 (0.02 - 0.04)	0.03 (0.02 - 0.05)	0.00	0.17 (0.12 - 0.27)	0.14 (0.11 - 0.21)	-0.71 (-0.97 - -0.44)
Andorra	Female	0.09 (0.06 - 0.12)	0.12 (0.09 - 0.17)	33.33	0.3 (0.21 - 0.42)	0.21 (0.16 - 0.3)	-1.35 (-1.64 - -1.05)
Angola	Female	15.4 (9.14 - 25.21)	25.75 (18.03 - 37.74)	67.21	0.47 (0.32 - 0.73)	0.29 (0.2 - 0.43)	-1.84 (-2.1 - -1.58)
Antigua and Barbuda	Female	0.02 (0.01 - 0.02)	0.03 (0.02 - 0.03)	50.00	0.05 (0.04 - 0.07)	0.05 (0.04 - 0.06)	0.17 (-0.02 - 0.36)
Argentina	Female	91.42 (78.01 - 123.3)	80.15 (62.31 - 128.97)	-12.33	0.52 (0.44 - 0.7)	0.29 (0.23 - 0.47)	-2.34 (-2.48 - -2.21)
Armenia	Female	1.53 (1.14 - 3.11)	2.59 (1.56 - 3.07)	69.28	0.09 (0.07 - 0.19)	0.12 (0.08 - 0.15)	1.43 (0.77 - 2.1)
Australia	Female	33.49 (25.81 - 38.2)	32.28 (26.07 - 40.53)	-3.61	0.32 (0.25 - 0.36)	0.18 (0.15 - 0.23)	-1.97 (-2.29 - -1.66)
Austria	Female	42.89 (17.86 - 51.38)	12.45 (10.17 - 19.36)	-70.97	0.65 (0.3 - 0.76)	0.15 (0.12 - 0.26)	-5.99 (-6.6 - -5.39)
Azerbaijan	Female	17.98 (9.58 - 24.83)	18.44 (10.81 - 25.37)	2.56	0.5 (0.28 - 0.67)	0.33 (0.2 - 0.44)	-1.97 (-2.38 - -1.56)
Bahrain	Female	0.6 (0.24 - 0.91)	0.52 (0.33 - 0.66)	-13.33	0.44 (0.18 - 0.64)	0.11 (0.08 - 0.14)	-5.65 (-6.09 - -5.21)
Bangladesh	Female	298.4 (221.51 - 411.18)	187.55 (129.12 - 311.57)	-37.15	0.73 (0.57 - 1.04)	0.25 (0.17 - 0.41)	-3.97 (-4.07 - -3.88)
Barbados	Female	0.54 (0.44 - 0.71)	0.51 (0.41 - 0.68)	-5.56	0.34 (0.28 - 0.44)	0.24 (0.19 - 0.32)	-1.28 (-1.38 - -1.19)
Belarus	Female	40.27 (24.64 - 47.35)	24.98 (17.68 - 29.56)	-37.97	0.59 (0.37 - 0.7)	0.36 (0.24 - 0.43)	-1.76 (-2.01 - -1.5)
Belgium	Female	32.09 (19.94 - 36.88)	18.49 (14.86 - 22.46)	-42.38	0.42 (0.27 - 0.48)	0.19 (0.16 - 0.23)	-2.75 (-2.87 - -2.64)
Belize	Female	0.24 (0.2 - 0.32)	0.56 (0.47 - 0.77)	133.33	0.38 (0.31 - 0.51)	0.34 (0.28 - 0.48)	-0.58 (-0.86 - -0.3)
Benin	Female	4.99 (3.64 - 6.64)	8.7 (5.99 - 12.63)	74.35	0.3 (0.22 - 0.39)	0.23 (0.16 - 0.32)	-1.17 (-1.32 - -1.02)
Bermuda	Female	0.11 (0.07 - 0.3)	0.23 (0.14 - 0.28)	109.09	0.32 (0.21 - 0.84)	0.39 (0.25 - 0.48)	-0.74 (-1.74 - 0.27)
Bhutan	Female	1.41 (0.99 - 1.94)	1 (0.55 - 1.89)	-29.08	0.68 (0.49 - 0.96)	0.25 (0.14 - 0.45)	-3.97 (-4.13 - -3.81)
Bolivia	Female	18.15 (10.39 - 24.62)	20.64 (13.26 - 27.81)	13.72	0.79 (0.48 - 1.03)	0.43 (0.27 - 0.58)	-2.41 (-2.5 - -2.31)
Bosnia and Herzegovina	Female	10.68 (7.54 - 13)	9.78 (5.09 - 12.98)	-8.43	0.45 (0.32 - 0.55)	0.36 (0.19 - 0.48)	-1.27 (-1.57 - -0.97)
Botswana	Female	1.02 (0.61 - 1.74)	1.64 (1.21 - 2.34)	60.78	0.23 (0.15 - 0.38)	0.17 (0.13 - 0.24)	-0.32 (-0.82 - 0.19)
Brazil	Female	219.24 (184.73 - 260.02)	266.68 (216.11 - 314.77)	21.64	0.37 (0.3 - 0.42)	0.22 (0.18 - 0.26)	-1.92 (-1.98 - -1.85)
Brunei	Female	0.25 (0.13 - 0.53)	0.89 (0.59 - 1.11)	256.00	0.35 (0.18 - 0.75)	0.48 (0.32 - 0.59)	2.56 (1.99 - 3.12)
Bulgaria	Female	29.06 (25.67 - 38.02)	20.47 (17.35 - 28.17)	-29.56	0.52 (0.45 - 0.66)	0.37 (0.31 - 0.47)	-1.72 (-1.98 - -1.45)
Burkina Faso	Female	13.22 (9.42 - 17.09)	16.87 (12.26 - 22.56)	27.61	0.39 (0.29 - 0.51)	0.24 (0.17 - 0.31)	-2.12 (-2.36 - -1.87)
Burundi	Female	18.2 (10.69 - 25.21)	18.88 (12.43 - 25.3)	3.74	0.86 (0.52 - 1.16)	0.5 (0.32 - 0.66)	-2.42 (-2.61 - -2.23)
Cambodia	Female	22.81 (14.31 - 29.3)	24.17 (16.59 - 33.27)	5.96	0.62 (0.41 - 0.78)	0.33 (0.23 - 0.46)	-2.5 (-2.63 - -2.37)
Cameroon	Female	10.95 (7.79 - 14.27)	20.57 (14.22 - 28.2)	87.85	0.31 (0.23 - 0.4)	0.23 (0.16 - 0.31)	-1.27 (-1.48 - -1.06)
Canada	Female	62.97 (43.08 - 70.05)	55.33 (46.27 - 69.2)	-12.13	0.37 (0.25 - 0.41)	0.2 (0.17 - 0.25)	-2.29 (-2.53 - -2.06)
Cape Verde	Female	0.1 (0.07 - 0.12)	0.14 (0.09 - 0.18)	40.00	0.07 (0.04 - 0.08)	0.06 (0.04 - 0.07)	-0.82 (-0.94 - -0.69)
Central African Republic	Female	4.74 (3.1 - 6.96)	7.51 (5.03 - 10.97)	58.44	0.54 (0.38 - 0.76)	0.49 (0.33 - 0.72)	-0.44 (-0.54 - -0.34)
Chad	Female	6.2 (4.3 - 8.52)	10.99 (7.81 - 15.04)	77.26	0.29 (0.21 - 0.4)	0.26 (0.18 - 0.34)	-0.42 (-0.58 - -0.25)
Chile	Female	27.66 (23.15 - 33)	28.08 (21.51 - 34.63)	1.52	0.45 (0.37 - 0.53)	0.24 (0.19 - 0.3)	-2.22 (-2.56 - -1.88)
China	Female	2146.75 (1024.4 - 2750.07)	936.14 (638.93 - 1124.42)	-56.39	0.43 (0.21 - 0.55)	0.1 (0.07 - 0.12)	-6.09 (-6.59 - -5.59)
Colombia	Female	53.65 (46.62 - 76.84)	72.47 (59.41 - 103.7)	35.08	0.45 (0.39 - 0.68)	0.26 (0.21 - 0.36)	-2.55 (-2.8 - -2.31)
Comoros	Female	1.67 (1.15 - 2.27)	1.9 (1.27 - 2.8)	13.77	0.91 (0.63 - 1.23)	0.6 (0.4 - 0.87)	-1.68 (-1.76 - -1.6)
Congo	Female	3.95 (2.84 - 5.88)	6.62 (4.41 - 10.27)	67.59	0.49 (0.37 - 0.71)	0.38 (0.26 - 0.59)	-1.21 (-1.46 - -0.97)
Costa Rica	Female	10.46 (4.85 - 12.65)	13.49 (7.16 - 16.85)	28.97	0.86 (0.44 - 1.01)	0.52 (0.28 - 0.65)	-1.86 (-2.09 - -1.63)
Cote d'Ivoire	Female	13.47 (8.74 - 18.01)	24.81 (15.52 - 34.86)	84.19	0.34 (0.24 - 0.43)	0.28 (0.19 - 0.38)	-0.79 (-0.92 - -0.67)
Croatia	Female	17.32 (12.89 - 19.77)	9.25 (7.66 - 11.39)	-46.59	0.53 (0.39 - 0.6)	0.24 (0.2 - 0.29)	-2.33 (-2.61 - -2.04)
Cuba	Female	4.95 (3.22 - 13.24)	39.03 (8.13 - 54.01)	688.48	0.09 (0.06 - 0.23)	0.47 (0.1 - 0.65)	6.92 (4.54 - 9.35)
Cyprus	Female	2.72 (1.42 - 3.86)	1.67 (1.26 - 2.21)	-38.60	0.62 (0.33 - 0.88)	0.2 (0.15 - 0.26)	-4.65 (-5.06 - -4.24)
Czech Republic	Female	66.18 (43.53 - 73.44)	28.93 (24.12 - 41.49)	-56.29	0.91 (0.64 - 1.01)	0.32 (0.26 - 0.49)	-4.3 (-4.5 - -4.1)
Democratic Republic of the Congo	Female	49.07 (34.21 - 71.01)	80.51 (58.39 - 116.54)	64.07	0.41 (0.28 - 0.58)	0.32 (0.23 - 0.47)	-0.9 (-0.98 - -0.82)
Denmark	Female	13.77 (9.22 - 15.62)	11 (7.27 - 12.96)	-20.12	0.36 (0.24 - 0.41)	0.23 (0.16 - 0.27)	-1.91 (-2.07 - -1.74)
Djibouti	Female	1.17 (0.73 - 1.67)	2.14 (1.23 - 3.49)	82.91	0.72 (0.47 - 1.01)	0.5 (0.3 - 0.81)	-1.52 (-1.7 - -1.34)
Dominica	Female	0.1 (0.08 - 0.13)	0.09 (0.07 - 0.12)	-10.00	0.25 (0.21 - 0.33)	0.21 (0.18 - 0.29)	-0.84 (-1 - -0.68)
Dominican Republic	Female	2.13 (1.26 - 4.37)	8.85 (3.08 - 14.29)	315.49	0.08 (0.05 - 0.16)	0.18 (0.06 - 0.29)	2.19 (1.4 - 2.99)
Ecuador	Female	23.74 (19.97 - 32.58)	29.22 (23.95 - 40.52)	23.08	0.62 (0.54 - 0.82)	0.37 (0.31 - 0.5)	-1.61 (-2 - -1.22)
Egypt	Female	239.84 (169.18 - 305.85)	267.24 (167.2 - 355.04)	11.42	1.14 (0.86 - 1.52)	0.69 (0.47 - 0.9)	-1.75 (-1.92 - -1.58)
El Salvador	Female	12.82 (9.34 - 16.3)	11.61 (8.41 - 16.72)	-9.44	0.69 (0.48 - 0.89)	0.35 (0.25 - 0.51)	-2.81 (-3 - -2.61)
Equatorial Guinea	Female	0.75 (0.51 - 1.2)	0.73 (0.4 - 1.19)	-2.67	0.52 (0.37 - 0.78)	0.19 (0.11 - 0.3)	-4.28 (-4.62 - -3.94)
Eritrea	Female	9.04 (5.29 - 12.73)	16.28 (9.55 - 24.8)	80.09	0.93 (0.61 - 1.27)	0.74 (0.45 - 1.11)	-0.91 (-0.99 - -0.84)
Estonia	Female	5.62 (3.24 - 6.54)	2.59 (1.55 - 3.3)	-53.91	0.53 (0.32 - 0.6)	0.22 (0.15 - 0.28)	-3.6 (-3.93 - -3.26)
Ethiopia	Female	263.37 (179.53 - 365.1)	281.08 (151.54 - 473.67)	6.72	1.47 (1 - 2.08)	0.75 (0.41 - 1.23)	-2.79 (-2.95 - -2.64)
Federated States of Micronesia	Female	0.1 (0.06 - 0.13)	0.08 (0.05 - 0.12)	-20.00	0.31 (0.2 - 0.43)	0.21 (0.13 - 0.28)	-1.58 (-1.69 - -1.46)
Fiji	Female	0.4 (0.29 - 0.61)	0.56 (0.4 - 0.74)	40.00	0.16 (0.12 - 0.24)	0.14 (0.1 - 0.19)	-0.34 (-0.54 - -0.15)
Finland	Female	15.46 (9.35 - 17.79)	9.83 (6.88 - 11.52)	-36.42	0.41 (0.25 - 0.47)	0.2 (0.15 - 0.24)	-2.67 (-3.03 - -2.31)
France	Female	148.72 (128.97 - 185.42)	110.58 (92.85 - 151.34)	-25.65	0.36 (0.31 - 0.44)	0.19 (0.16 - 0.27)	-2.17 (-2.35 - -2)
Gabon	Female	1.16 (0.87 - 1.7)	1.53 (1.03 - 2.32)	31.90	0.32 (0.23 - 0.47)	0.23 (0.15 - 0.35)	-1.3 (-1.62 - -0.97)
Georgia	Female	27.5 (13.23 - 34.28)	13.97 (8.68 - 16.53)	-49.20	0.8 (0.39 - 1)	0.48 (0.32 - 0.57)	-2.6 (-3.24 - -1.95)

Germany	Female	358.85 (182.68 - 408.18)	157.27 (126.18 - 194.55)	-56.17	0.54 (0.29 - 0.6)	0.19 (0.15 - 0.25)	-4.08 (-4.55 - -3.6)
Ghana	Female	6.39 (4.66 - 8.67)	10.29 (7.97 - 14.23)	61.03	0.14 (0.09 - 0.18)	0.1 (0.07 - 0.13)	-1.3 (-1.42 - -1.17)
Greece	Female	53.83 (47.72 - 71.11)	74.31 (47.95 - 88.62)	38.05	0.71 (0.63 - 1.01)	0.67 (0.51 - 0.77)	-0.14 (-0.76 - 0.48)
Greenland	Female	0.06 (0.04 - 0.07)	0.03 (0.03 - 0.04)	-50.00	0.25 (0.15 - 0.33)	0.12 (0.09 - 0.15)	-2.97 (-3.25 - -2.68)
Grenada	Female	0.09 (0.07 - 0.12)	0.09 (0.07 - 0.14)	0.00	0.21 (0.17 - 0.29)	0.13 (0.1 - 0.2)	-1.67 (-1.88 - -1.46)
Guam	Female	0.03 (0.02 - 0.03)	0.05 (0.03 - 0.06)	66.67	0.05 (0.04 - 0.07)	0.06 (0.04 - 0.07)	0.46 (0.11 - 0.81)
Guatemala	Female	14.12 (10.35 - 22.92)	17.51 (13.63 - 28.29)	24.01	0.52 (0.42 - 0.85)	0.26 (0.21 - 0.41)	-2.47 (-2.83 - -2.11)
Guinea	Female	12.83 (8.49 - 16.65)	18.12 (11.29 - 25.99)	41.23	0.52 (0.37 - 0.67)	0.41 (0.28 - 0.56)	-0.86 (-0.95 - -0.77)
Guinea-Bissau	Female	1.46 (0.97 - 2.02)	1.93 (1.35 - 2.76)	32.19	0.42 (0.29 - 0.58)	0.32 (0.23 - 0.44)	-1.05 (-1.11 - -1)
Guyana	Female	0.36 (0.19 - 1.18)	1.01 (0.68 - 1.29)	180.56	0.12 (0.07 - 0.4)	0.28 (0.19 - 0.36)	2.92 (1.53 - 4.33)
Haiti	Female	16.35 (11.8 - 20.96)	26.76 (14.56 - 39.95)	63.67	0.73 (0.53 - 0.94)	0.59 (0.33 - 0.87)	-0.79 (-0.9 - -0.69)
Honduras	Female	0.63 (0.43 - 0.79)	1.34 (0.92 - 1.83)	112.70	0.04 (0.03 - 0.05)	0.04 (0.02 - 0.05)	-0.31 (-0.4 - -0.21)
Hungary	Female	53.43 (27.03 - 62.38)	17.45 (14.33 - 22.35)	-67.34	0.73 (0.37 - 0.85)	0.2 (0.17 - 0.26)	-4.92 (-5.08 - -4.76)
Iceland	Female	0.5 (0.39 - 0.58)	0.32 (0.27 - 0.51)	-36.00	0.34 (0.26 - 0.39)	0.14 (0.11 - 0.22)	-3.12 (-3.21 - -3.04)
India	Female	1850.27 (1006.25 - 2504.77)	1751.49 (1311.41 - 2327.7)	-5.34	0.53 (0.3 - 0.69)	0.28 (0.21 - 0.37)	-2.61 (-2.75 - -2.47)
Indonesia	Female	293.04 (181.69 - 350.58)	298.4 (206.37 - 366.21)	1.83	0.41 (0.26 - 0.49)	0.25 (0.17 - 0.31)	-1.76 (-1.9 - -1.63)
Iran	Female	60.01 (45.48 - 100.79)	105.51 (66.87 - 121.74)	75.82	0.31 (0.24 - 0.51)	0.28 (0.17 - 0.33)	0.82 (0.21 - 1.44)
Iraq	Female	30.68 (11.06 - 51.32)	23.66 (17.93 - 32.88)	-22.88	0.51 (0.2 - 0.82)	0.14 (0.11 - 0.2)	-5.12 (-6.16 - -4.06)
Ireland	Female	9.88 (7.41 - 11.2)	8.4 (5.81 - 10.12)	-14.98	0.47 (0.36 - 0.53)	0.25 (0.18 - 0.3)	-2.23 (-2.53 - -1.93)
Israel	Female	13.92 (12.05 - 17.35)	16.64 (13.6 - 21.27)	19.54	0.54 (0.47 - 0.67)	0.31 (0.25 - 0.4)	-2.32 (-2.49 - -2.16)
Italy	Female	251.97 (189.46 - 274.44)	177.07 (144.39 - 218.46)	-29.73	0.6 (0.44 - 0.65)	0.3 (0.24 - 0.38)	-2.36 (-2.61 - -2.1)
Jamaica	Female	2.36 (1.18 - 2.87)	2.6 (1.46 - 3.53)	10.17	0.22 (0.11 - 0.27)	0.17 (0.1 - 0.23)	-0.39 (-0.98 - 0.2)
Japan	Female	50.56 (46.44 - 70.25)	72.12 (48.25 - 80.64)	42.64	0.06 (0.05 - 0.08)	0.05 (0.03 - 0.05)	-0.21 (-0.38 - -0.03)
Jordan	Female	1.84 (0.84 - 2.71)	2.32 (1.26 - 3.18)	26.09	0.16 (0.07 - 0.24)	0.07 (0.03 - 0.09)	-3.89 (-4.39 - -3.38)
Kazakhstan	Female	36.63 (28.31 - 48.12)	29.62 (24.87 - 36.15)	-19.14	0.44 (0.34 - 0.57)	0.3 (0.25 - 0.37)	-1.8 (-2.43 - -1.16)
Kenya	Female	24.62 (16.97 - 33.2)	46.09 (29.63 - 56.88)	87.21	0.32 (0.22 - 0.42)	0.25 (0.16 - 0.31)	-1.02 (-1.15 - -0.89)
Kiribati	Female	0.04 (0.03 - 0.06)	0.06 (0.04 - 0.1)	50.00	0.15 (0.11 - 0.24)	0.14 (0.1 - 0.22)	-0.2 (-0.35 - -0.06)
Kuwait	Female	1.34 (0.96 - 1.58)	1.44 (1.14 - 1.85)	7.46	0.27 (0.19 - 0.31)	0.1 (0.08 - 0.12)	-2.74 (-3.74 - -1.73)
Kyrgyzstan	Female	7.55 (3.66 - 9.23)	4.17 (3.41 - 5.04)	-44.77	0.38 (0.19 - 0.46)	0.15 (0.12 - 0.18)	-2.81 (-3.2 - -2.41)
Laos	Female	9.87 (5.56 - 13.94)	9.92 (6.43 - 13.4)	0.51	0.65 (0.38 - 0.88)	0.35 (0.23 - 0.47)	-2.4 (-2.6 - -2.2)
Latvia	Female	8.3 (4.69 - 9.73)	4.46 (2.29 - 5.79)	-46.27	0.45 (0.27 - 0.52)	0.27 (0.15 - 0.36)	-2.05 (-2.29 - -1.81)
Lebanon	Female	29.59 (20.03 - 39.08)	33.28 (23.51 - 41.27)	12.47	1.83 (1.26 - 2.41)	0.86 (0.61 - 1.07)	-3.08 (-3.29 - -2.87)
Lesotho	Female	1.85 (1.35 - 2.84)	2.64 (1.74 - 4.11)	42.70	0.28 (0.21 - 0.43)	0.33 (0.21 - 0.5)	1.32 (0.68 - 1.96)
Liberia	Female	2.15 (1.45 - 2.93)	3.14 (2.22 - 4.3)	46.05	0.3 (0.21 - 0.4)	0.21 (0.14 - 0.28)	-1.6 (-1.88 - -1.33)
Libya	Female	24.09 (16.59 - 33.29)	43.07 (29.65 - 58.79)	78.79	1.75 (1.23 - 2.47)	1.45 (1.03 - 1.97)	-0.86 (-1.09 - -0.63)
Lithuania	Female	13.33 (9.2 - 15.1)	6.12 (5.19 - 8.05)	-54.09	0.56 (0.4 - 0.64)	0.24 (0.2 - 0.36)	-3.91 (-4.41 - -3.41)
Luxembourg	Female	1.08 (0.9 - 1.32)	0.9 (0.68 - 1.47)	-16.67	0.38 (0.32 - 0.48)	0.2 (0.15 - 0.34)	-2.51 (-2.78 - -2.24)
Macedonia	Female	4.04 (2.97 - 5.01)	4.85 (2.96 - 6.21)	20.05	0.4 (0.29 - 0.49)	0.34 (0.21 - 0.44)	-1.12 (-1.44 - -0.79)
Madagascar	Female	34.11 (22.68 - 43.65)	47.32 (31.7 - 63.57)	38.73	0.74 (0.5 - 0.93)	0.49 (0.33 - 0.66)	-1.63 (-1.79 - -1.46)
Malawi	Female	14.27 (5.77 - 21.37)	16.74 (10.82 - 24.39)	17.31	0.38 (0.19 - 0.54)	0.24 (0.16 - 0.35)	-2.7 (-3.12 - -2.29)
Malaysia	Female	23.93 (9.97 - 34.79)	25.35 (9.37 - 40.57)	5.93	0.38 (0.16 - 0.55)	0.18 (0.07 - 0.29)	-2.68 (-2.98 - -2.39)
Maldives	Female	0.08 (0.04 - 0.14)	0.07 (0.05 - 0.08)	-12.50	0.13 (0.06 - 0.22)	0.04 (0.03 - 0.05)	-4.74 (-4.95 - -4.53)
Mali	Female	20.64 (13.01 - 26.9)	24.73 (15.44 - 35.09)	19.82	0.61 (0.43 - 0.76)	0.34 (0.24 - 0.46)	-2.46 (-2.63 - -2.28)
Malta	Female	1.57 (1.26 - 1.82)	1.42 (1.21 - 1.81)	-9.55	0.7 (0.57 - 0.82)	0.38 (0.32 - 0.51)	-2.19 (-2.34 - -2.05)
Marshall Islands	Female	0.03 (0.02 - 0.04)	0.05 (0.03 - 0.07)	66.67	0.25 (0.18 - 0.32)	0.22 (0.13 - 0.32)	-0.48 (-0.7 - -0.25)
Mauritania	Female	2.19 (1.57 - 2.92)	2.59 (1.86 - 3.52)	18.26	0.3 (0.22 - 0.41)	0.19 (0.14 - 0.25)	-1.71 (-1.78 - -1.64)
Mauritius	Female	0.87 (0.7 - 1.01)	1.74 (0.7 - 2.19)	100.00	0.18 (0.14 - 0.21)	0.22 (0.09 - 0.28)	0.44 (-0.15 - 1.04)
Mexico	Female	183.2 (143.85 - 202.94)	222.35 (185.83 - 275.61)	21.37	0.63 (0.49 - 0.71)	0.36 (0.3 - 0.45)	-2.03 (-2.23 - -1.84)
Moldova	Female	18.6 (14.61 - 21.66)	11.74 (9.01 - 13.69)	-36.88	0.75 (0.59 - 0.88)	0.46 (0.34 - 0.55)	-2.16 (-2.48 - -1.84)
Mongolia	Female	1.08 (0.64 - 2.18)	1.62 (1.21 - 2.46)	50.00	0.15 (0.09 - 0.28)	0.11 (0.09 - 0.16)	-1.48 (-1.99 - -0.97)
Montenegro	Female	2.24 (1.72 - 3.2)	2.05 (1.61 - 2.99)	-8.48	0.66 (0.51 - 0.93)	0.46 (0.36 - 0.66)	-1.77 (-2.13 - -1.41)
Morocco	Female	153.91 (101.53 - 195.97)	168.18 (111.64 - 229.2)	9.27	1.49 (1.05 - 1.87)	0.97 (0.65 - 1.31)	-1.47 (-1.55 - -1.4)
Mozambique	Female	56.01 (39.61 - 73.7)	60.05 (39.24 - 83.02)	7.21	0.96 (0.69 - 1.24)	0.52 (0.34 - 0.72)	-2.58 (-2.93 - -2.23)
Myanmar	Female	144.85 (84.52 - 197.37)	106.5 (72.63 - 141.53)	-26.48	0.88 (0.54 - 1.2)	0.4 (0.27 - 0.53)	-3.08 (-3.42 - -2.74)
Namibia	Female	3.18 (1.84 - 4.35)	3.33 (2.09 - 4.78)	4.72	0.62 (0.38 - 0.82)	0.33 (0.22 - 0.46)	-2.79 (-3.41 - -2.18)
Nepal	Female	45.49 (30.87 - 64.74)	43.32 (25.71 - 80.54)	-4.77	0.63 (0.44 - 0.9)	0.31 (0.19 - 0.57)	-2.66 (-2.93 - -2.39)
Netherlands	Female	42.64 (31.64 - 47.36)	36.46 (27.21 - 42.96)	-14.49	0.41 (0.31 - 0.46)	0.25 (0.2 - 0.3)	-2.02 (-2.24 - -1.8)
New Zealand	Female	7.06 (3.42 - 8.31)	6.26 (3.24 - 7.54)	-11.33	0.33 (0.17 - 0.38)	0.19 (0.1 - 0.23)	-1.45 (-1.84 - -1.05)
Nicaragua	Female	2.9 (2.15 - 3.84)	5.17 (2.98 - 6.91)	78.28	0.24 (0.18 - 0.32)	0.2 (0.11 - 0.26)	-0.37 (-0.59 - -0.15)
Niger	Female	9.04 (5.8 - 12.75)	13.73 (9.74 - 19.07)	51.88	0.34 (0.25 - 0.47)	0.23 (0.16 - 0.31)	-1.89 (-2.05 - -1.72)
Nigeria	Female	775.18 (398.67 - 1297.17)	1216.23 (731.94 - 1863.77)	56.90	2.28 (1.32 - 3.55)	1.62 (1.01 - 2.46)	-1.51 (-1.67 - -1.34)
North Korea	Female	19.88 (11.17 - 28.71)	26.75 (16.07 - 36.61)	34.56	0.19 (0.11 - 0.27)	0.16 (0.1 - 0.22)	-0.6 (-0.82 - -0.38)
Northern Mariana Islands	Female	0.01 (0.01 - 0.01)	0.01 (0.01 - 0.02)	0.00	0.08 (0.06 - 0.1)	0.06 (0.04 - 0.07)	-1.01 (-1.3 - -0.71)
Norway	Female	10.29 (8.09 - 11.88)	6.81 (6.08 - 8.76)	-33.82	0.32 (0.26 - 0.37)	0.17 (0.15 - 0.22)	-2.33 (-2.52 - -2.14)
Oman	Female	3.29 (1.5 - 5.01)	3.57 (1.74 - 5.06)	8.51	0.64 (0.3 - 0.95)	0.3 (0.15 - 0.42)	-2.93 (-3.4 - -2.46)
Pakistan	Female	459.77 (315.74 - 627.63)	996.28 (626.86 - 1497.19)	116.69	1.09 (0.78 - 1.48)	1.05 (0.69 - 1.62)	-0.23 (-0.45 - -0.02)
Palestine	Female	1.75 (0.89 - 3.54)	4.66 (3.42 - 6.5)	166.29	0.26 (0.14 - 0.53)	0.28 (0.22 - 0.38)	0.29 (0.04 - 0.55)
Panama	Female	1.73 (1.48 - 2.38)	3.83 (2.39 - 4.6)	121.39	0.19 (0.17 - 0.27)	0.19 (0.12 - 0.23)	0.57 (0.07 - 1.08)
Papua New Guinea	Female	4.7 (3.23 - 6.41)	8.93 (5.8 - 12.66)	90.00	0.36 (0.25 - 0.49)	0.29 (0.19 - 0.41)	-0.58 (-0.69 - -0.47)
Paraguay	Female	4.16 (3.15 - 5.49)	7.06 (4.44 - 9.47)	69.71	0.28 (0.21 - 0.36)	0.23 (0.14 - 0.32)	-0.25 (-0.61 - 0.11)
Peru	Female	17.33 (12.84 - 22.59)	23.74 (11.75 - 34.11)	36.99	0.23 (0.15 - 0.3)	0.15 (0.07 - 0.21)	-1.46 (-1.65 - -1.27)
Philippines	Female	46.1 (38.72 - 66.21)	54.2 (37.24 - 108.96)	17.57	0.21 (0.18 - 0.28)	0.13 (0.09 - 0.24)	-1.94 (-2.15 - -1.73)
Poland	Female	180.83 (137.17 - 199.08)	89.85 (76.02 - 124.12)	-50.31	0.77 (0.58 - 0.85)	0.29 (0.25 - 0.4)	-3.98 (-4.2 - -3.76)
Portugal	Female	27.56 (19.65 - 31.09)	20.63 (12.93 - 24.79)	-25.15	0.4 (0.29 - 0.46)	0.19 (0.12 - 0.23)	-3.09 (-3.32 - -2.85)
Puerto Rico	Female	9.8 (5.55 - 11.73)	7.53 (5.13 - 8.93)	-23.16	0.5 (0.28 - 0.59)	0.26 (0.18 - 0.31)	-3.2 (-3.61 - -2.79)
Qatar	Female	0.06 (0.04 - 0.08)	0.18 (0.1 - 0.25)	200.00	0.09 (0.07 - 0.12)	0.05 (0.03 - 0.07)	-2.23 (-2.64 - -1.81)
Romania	Female	78.92 (57.36 - 87.57)	42.01 (34.21 - 49.65)	-46.77	0.58 (0.42 - 0.65)	0.28 (0.22 - 0.33)	-3.1 (-3.31 - -2.88)
Russian Federation	Female	545.11 (356.37 - 611.2)	345.12 (283.17 - 405.3)	-36.69	0.59 (0.37 - 0.67)	0.33 (0.28 - 0.41)	-2.69 (-3.18 - -2.21)
Rwanda	Female	23.95 (15.29 - 31.67)	22.18 (13.86 - 32.05)	-7.39	0.86 (0.55 - 1.11)	0.43 (0.27 - 0.6)	-3.13 (-3.46 - -2.81)
Saint Lucia	Female	0.12 (0.09 - 0.18)	0.15 (0.12 - 0.2)	25.00	0.2 (0.16 - 0.3)	0.15 (0.12 - 0.19)	-0.91 (-1.07 - -0.74)
Saint Vincent and the Grenadines	Female	0.06 (0.03 - 0.23)	0.19 (0.16 - 0.23)	216.67	0.13 (0.06 - 0.48)	0.31 (0.25 - 0.38)	1.9 (0.74 - 3.08)
Samoa	Female	0.22 (0.14 - 0.3)	0.22 (0.13 - 0.31)	0.00	0.38 (0.27 - 0.49)	0.27 (0.16 - 0.39)	-1.27 (-1.41 - -1.14)
Sao Tome and Principe	Female	0.02 (0.02 - 0.04)	0.03 (0.02 - 0.05)	50.00	0.05 (0.04 - 0.08)	0.05 (0.03 - 0.06)	-0.41 (-0.51 - -0.31)
Saudi Arabia	Female	22.75 (5.53 - 42.11)	39.82 (8.3 - 69.79)	75.03	0.49 (0.12 - 0.89)	0.35 (0.08 - 0.58)	-0.71 (-1.06 - -0.36)
Senegal	Female	7.31 (5.26 - 9.89)	11.98 (8.98 - 17.03)	63.89	0.28 (0.2 - 0.37)	0.23 (0.17 - 0.32)	-0.72 (-0.8 - -0.63)
Serbia	Female	32.44 (22.53 - 40.92)	26.76 (16.67 - 33.11)	-17.51	0.6 (0.41 - 0.76)	0.39 (0.24 - 0.49)	-1.41 (-1.6 - -1.21)
Seychelles	Female	0.16 (0.1 - 0.31)	0.23 (0.15 - 0.29)	43.75	0.47 (0.3 - 0.91)	0.42 (0.28 - 0.54)	-0.54 (-0.86 - -0.22)
Sierra Leone	Female	4.08 (2.84 - 5.57)	6.29 (4.29 - 8.63)	54.17	0.28 (0.21 - 0.38)	0.24 (0.16 - 0.33)	-0.49 (-0.59 - -0.39)

Singapore	Female	1.58 (1.14 - 1.8)	2 (1.2 - 2.45)	26.58	0.11 (0.08 - 0.13)	0.06 (0.04 - 0.07)	-1.6 (-2.03 - -1.17)
Slovakia	Female	21.06 (15.5 - 27.02)	15.09 (11.95 - 21.87)	-28.35	0.66 (0.49 - 0.86)	0.37 (0.29 - 0.53)	-2.25 (-2.35 - -2.16)
Slovenia	Female	6.51 (4.1 - 7.52)	4.02 (2.64 - 4.87)	-38.25	0.49 (0.32 - 0.56)	0.2 (0.15 - 0.24)	-3.41 (-3.74 - -3.09)
Solomon Islands	Female	0.28 (0.19 - 0.38)	0.52 (0.34 - 0.73)	85.71	0.3 (0.21 - 0.4)	0.25 (0.16 - 0.35)	-0.48 (-0.6 - -0.37)
Somalia	Female	19.5 (8.06 - 31.19)	48.61 (25.9 - 79.55)	149.28	0.84 (0.44 - 1.27)	0.83 (0.46 - 1.31)	-0.38 (-0.57 - -0.19)
South Africa	Female	31.71 (23.43 - 40.1)	39.85 (28.09 - 49.93)	25.67	0.2 (0.15 - 0.25)	0.14 (0.1 - 0.18)	-1.17 (-2.1 - -0.24)
South Korea	Female	10.67 (7.83 - 23.33)	19.41 (11.23 - 23.44)	81.91	0.05 (0.04 - 0.12)	0.05 (0.03 - 0.06)	0.12 (-0.07 - 0.3)
South Sudan	Female	12.18 (5.02 - 20.59)	18.38 (12.16 - 25.45)	50.90	0.62 (0.3 - 0.97)	0.54 (0.36 - 0.74)	-0.85 (-1.1 - -0.59)
Spain	Female	115.02 (78.2 - 127.75)	92.22 (57.85 - 109.23)	-19.82	0.42 (0.29 - 0.47)	0.21 (0.14 - 0.24)	-2.57 (-2.68 - -2.47)
Sri Lanka	Female	27.04 (20.51 - 34.94)	25.91 (18.27 - 36.38)	-4.18	0.43 (0.32 - 0.54)	0.2 (0.14 - 0.28)	-3.64 (-4.12 - -3.16)
Sudan	Female	39.12 (25.45 - 58.75)	50.14 (34.95 - 68.48)	28.17	0.57 (0.39 - 0.82)	0.37 (0.25 - 0.5)	-1.53 (-1.57 - -1.5)
Suriname	Female	0.6 (0.49 - 0.9)	1.01 (0.8 - 1.24)	68.33	0.37 (0.29 - 0.55)	0.34 (0.27 - 0.41)	-0.07 (-0.41 - 0.28)
Swaziland	Female	0.76 (0.53 - 1.15)	1.06 (0.67 - 1.65)	39.47	0.3 (0.22 - 0.45)	0.25 (0.16 - 0.37)	-0.07 (-0.71 - 0.58)
Sweden	Female	18.63 (13.08 - 21.05)	14.64 (10.33 - 17.16)	-21.42	0.26 (0.2 - 0.29)	0.16 (0.12 - 0.19)	-1.73 (-1.87 - -1.58)
Switzerland	Female	14.41 (10.18 - 16.33)	11.9 (9.11 - 14.56)	-17.42	0.29 (0.2 - 0.33)	0.16 (0.13 - 0.21)	-2.08 (-2.22 - -1.94)
Syria	Female	2.02 (1.15 - 2.71)	2.89 (1.32 - 4.46)	43.07	0.06 (0.03 - 0.08)	0.04 (0.02 - 0.06)	-1.18 (-1.4 - -0.97)
Tajikistan	Female	4.02 (2.69 - 5.28)	6.32 (4.47 - 8.01)	57.21	0.22 (0.13 - 0.3)	0.18 (0.11 - 0.23)	-0.99 (-1.14 - -0.85)
Tanzania	Female	64.14 (35.68 - 88.61)	100.7 (64.21 - 143.95)	57.00	0.66 (0.42 - 0.9)	0.47 (0.31 - 0.65)	-1.71 (-1.93 - -1.5)
Thailand	Female	47.11 (31.14 - 81.93)	38.26 (24.55 - 70.53)	-18.79	0.19 (0.13 - 0.33)	0.08 (0.05 - 0.15)	-3.38 (-3.56 - -3.21)
The Bahamas	Female	0.42 (0.35 - 0.53)	0.56 (0.45 - 0.74)	33.33	0.37 (0.31 - 0.46)	0.28 (0.22 - 0.36)	-1.18 (-1.34 - -1.01)
The Gambia	Female	0.93 (0.59 - 1.25)	1.95 (1.35 - 2.74)	109.68	0.28 (0.2 - 0.37)	0.25 (0.18 - 0.36)	-0.27 (-0.38 - -0.15)
Timor-Leste	Female	1.26 (0.73 - 1.73)	1.46 (0.86 - 2.01)	15.87	0.53 (0.34 - 0.7)	0.3 (0.19 - 0.41)	-2.27 (-2.39 - -2.16)
Togo	Female	3.85 (2.77 - 5.07)	5.81 (4.13 - 8.06)	50.91	0.31 (0.23 - 0.42)	0.21 (0.15 - 0.29)	-1.63 (-1.8 - -1.47)
Tonga	Female	0.05 (0.04 - 0.07)	0.05 (0.03 - 0.08)	0.00	0.16 (0.11 - 0.21)	0.12 (0.08 - 0.18)	-0.88 (-0.97 - -0.78)
Trinidad and Tobago	Female	0.77 (0.6 - 0.89)	1.49 (0.57 - 2.21)	93.51	0.14 (0.11 - 0.17)	0.19 (0.07 - 0.28)	2.07 (1.35 - 2.8)
Tunisia	Female	31.89 (24.09 - 41.64)	34.91 (24.2 - 49.26)	9.47	0.99 (0.77 - 1.34)	0.57 (0.4 - 0.8)	-2.14 (-2.21 - -2.08)
Turkey	Female	110.95 (79.41 - 173.94)	77.1 (59.8 - 109.78)	-30.51	0.46 (0.33 - 0.73)	0.17 (0.13 - 0.25)	-3.82 (-3.97 - -3.67)
Turkmenistan	Female	7.67 (5.64 - 9.19)	8.23 (5.61 - 9.95)	7.30	0.49 (0.39 - 0.59)	0.34 (0.24 - 0.42)	-1.11 (-1.52 - -0.7)
Uganda	Female	50.11 (34.31 - 64.55)	76.59 (48.9 - 120.55)	52.84	0.83 (0.6 - 1.05)	0.53 (0.35 - 0.81)	-2.1 (-2.31 - -1.89)
Ukraine	Female	185.91 (139.31 - 233)	177.49 (132.86 - 258.3)	-4.53	0.55 (0.39 - 0.7)	0.63 (0.44 - 0.96)	-0.3 (-0.82 - 0.22)
United Arab Emirates	Female	1.31 (0.91 - 1.94)	4.17 (2.62 - 6.34)	218.32	0.35 (0.24 - 0.51)	0.24 (0.15 - 0.39)	-1.33 (-1.42 - -1.24)
United Kingdom	Female	181.32 (135.19 - 196.64)	135.17 (102.22 - 147.7)	-25.45	0.43 (0.31 - 0.46)	0.25 (0.2 - 0.28)	-1.77 (-2.04 - -1.5)
United States	Female	711.05 (526.07 - 758.3)	585.81 (531.66 - 825.88)	-17.61	0.43 (0.32 - 0.46)	0.24 (0.22 - 0.36)	-2.29 (-2.4 - -2.18)
Uruguay	Female	9.63 (6.89 - 11.12)	7.59 (5.69 - 9.43)	-21.18	0.5 (0.35 - 0.59)	0.31 (0.22 - 0.38)	-1.54 (-1.88 - -1.19)
Uzbekistan	Female	27.35 (20.3 - 42.75)	47.08 (36.76 - 59.15)	72.14	0.33 (0.24 - 0.53)	0.31 (0.25 - 0.39)	-0.2 (-0.38 - -0.01)
Vanuatu	Female	0.18 (0.09 - 0.32)	0.41 (0.16 - 0.73)	127.78	0.39 (0.19 - 0.68)	0.39 (0.15 - 0.68)	-0.03 (-0.18 - 0.13)
Venezuela	Female	31.45 (21.87 - 37.5)	57.85 (29.95 - 76.25)	83.94	0.48 (0.34 - 0.57)	0.38 (0.2 - 0.5)	-0.59 (-0.82 - -0.36)
Vietnam	Female	100.5 (66.23 - 130.27)	91.4 (60.41 - 122.22)	-9.05	0.35 (0.24 - 0.45)	0.18 (0.12 - 0.24)	-2.6 (-2.64 - -2.56)
Virgin Islands, U.S.	Female	0.05 (0.04 - 0.09)	0.08 (0.06 - 0.1)	60.00	0.1 (0.07 - 0.17)	0.1 (0.08 - 0.13)	0.71 (0.22 - 1.21)
Yemen	Female	23.47 (12.13 - 36.53)	45.57 (31.73 - 64.02)	94.16	0.58 (0.34 - 0.9)	0.45 (0.32 - 0.64)	-1.03 (-1.14 - -0.93)
Zambia	Female	26.37 (15.52 - 36.15)	34.7 (22.75 - 46.67)	31.59	0.92 (0.57 - 1.19)	0.54 (0.36 - 0.71)	-2.43 (-2.68 - -2.17)
Zimbabwe	Female	8.73 (5.35 - 11.42)	17.92 (9.91 - 26.48)	105.27	0.25 (0.17 - 0.32)	0.32 (0.2 - 0.44)	2.18 (1.29 - 3.08)
Afghanistan	Male	100.69 (36.83 - 181.45)	165.72 (100.31 - 266.64)	64.58	2.47 (0.88 - 4.5)	1.91 (1.2 - 3.05)	-1.01 (-1.43 - -0.6)
Albania	Male	12.7 (9.63 - 24.94)	10.12 (6.71 - 18.59)	-20.31	0.95 (0.72 - 1.88)	0.58 (0.39 - 1.04)	-2.08 (-2.35 - -1.82)
Algeria	Male	159.1 (111.58 - 208.62)	172.4 (117.93 - 211.97)	8.36	1.65 (1.25 - 2.31)	0.92 (0.63 - 1.13)	-1.98 (-2.15 - -1.82)
American Samoa	Male	0.02 (0.02 - 0.04)	0.03 (0.02 - 0.04)	50.00	0.16 (0.12 - 0.24)	0.14 (0.09 - 0.17)	-0.31 (-0.62 - 0)
Andorra	Male	0.21 (0.16 - 0.28)	0.23 (0.18 - 0.33)	9.52	0.64 (0.49 - 0.86)	0.39 (0.29 - 0.55)	-1.84 (-2.06 - -1.63)
Angola	Male	36.83 (21.57 - 53.21)	49.1 (35.07 - 69.41)	33.32	1.13 (0.73 - 1.65)	0.66 (0.46 - 0.95)	-2.05 (-2.24 - -1.86)
Antigua and Barbuda	Male	0.09 (0.08 - 0.14)	0.12 (0.09 - 0.16)	33.33	0.38 (0.31 - 0.55)	0.25 (0.2 - 0.34)	-1.45 (-1.73 - -1.17)
Argentina	Male	163.6 (136.01 - 230.39)	149.55 (117.79 - 223.97)	-8.59	1.07 (0.89 - 1.5)	0.64 (0.51 - 0.96)	-2.01 (-2.17 - -1.84)
Armenia	Male	3.14 (2.31 - 5.59)	5.46 (2.33 - 7)	73.89	0.23 (0.17 - 0.39)	0.31 (0.13 - 0.4)	1.18 (0.39 - 1.97)
Australia	Male	53.82 (36.26 - 65.99)	52.28 (39.04 - 65.02)	-2.86	0.59 (0.4 - 0.73)	0.31 (0.23 - 0.39)	-2.26 (-2.51 - -2.01)
Austria	Male	47.22 (24.96 - 55.73)	24.31 (19.37 - 36.82)	-48.52	1.06 (0.56 - 1.24)	0.36 (0.29 - 0.57)	-4.3 (-4.85 - -3.74)
Azerbaijan	Male	33.46 (24.2 - 43.62)	39.56 (27.24 - 51.05)	18.23	1.11 (0.83 - 1.56)	0.84 (0.59 - 1.07)	-1.16 (-1.43 - -0.9)
Bahrain	Male	2.39 (1.16 - 3.19)	2.8 (1.87 - 3.5)	17.15	1.63 (0.68 - 2.21)	0.39 (0.24 - 0.49)	-6.47 (-7.02 - -5.92)
Bangladesh	Male	761.93 (511.84 - 1297.32)	349.09 (225.57 - 672.64)	-54.18	1.87 (1.26 - 3.18)	0.49 (0.32 - 0.94)	-5.13 (-5.23 - -5.02)
Barbados	Male	0.91 (0.71 - 1.19)	0.82 (0.65 - 1.14)	-9.89	0.73 (0.57 - 0.95)	0.43 (0.34 - 0.59)	-2.18 (-2.3 - -2.07)
Belarus	Male	61.11 (36.17 - 75.72)	39.68 (26.58 - 50.47)	-35.07	1.2 (0.71 - 1.46)	0.69 (0.46 - 0.88)	-1.85 (-2.19 - -1.5)
Belgium	Male	59.54 (41.67 - 71.73)	36.31 (29.7 - 55.99)	-39.02	0.96 (0.68 - 1.17)	0.42 (0.34 - 0.68)	-3.04 (-3.14 - -2.93)
Belize	Male	0.49 (0.35 - 0.7)	0.91 (0.68 - 1.16)	85.71	0.76 (0.54 - 1.08)	0.56 (0.43 - 0.73)	-1.59 (-1.9 - -1.28)
Benin	Male	12.25 (8.46 - 15.81)	17.13 (12.38 - 22.86)	39.84	0.77 (0.55 - 0.99)	0.52 (0.37 - 0.69)	-1.46 (-1.54 - -1.38)
Bermuda	Male	0.2 (0.14 - 0.33)	0.22 (0.16 - 0.28)	10.00	0.69 (0.48 - 1.11)	0.46 (0.35 - 0.62)	-1.47 (-1.66 - -1.28)
Bhutan	Male	3.37 (2.13 - 5.59)	2.3 (1.21 - 4.85)	-31.75	1.63 (0.99 - 2.8)	0.57 (0.31 - 1.19)	-3.95 (-4.02 - -3.87)
Bolivia	Male	38.76 (25.44 - 48.99)	38.13 (25.64 - 54.97)	-1.63	1.79 (1.19 - 2.31)	0.85 (0.56 - 1.22)	-2.72 (-2.75 - -2.68)
Bosnia and Herzegovina	Male	16.23 (10.66 - 19.57)	11.86 (6.47 - 15.18)	-26.93	0.79 (0.51 - 0.95)	0.49 (0.27 - 0.62)	-1.93 (-2.32 - -1.55)
Botswana	Male	2.52 (1.83 - 3.7)	2.48 (1.86 - 3.57)	-1.59	0.71 (0.52 - 1.04)	0.34 (0.26 - 0.47)	-2.79 (-3 - -2.58)
Brazil	Male	471.46 (371.89 - 631.4)	456.85 (333.34 - 633.23)	-3.10	0.85 (0.66 - 1.12)	0.43 (0.31 - 0.59)	-2.49 (-2.57 - -2.42)
Brunei	Male	0.42 (0.29 - 0.77)	1.03 (0.73 - 1.3)	145.24	0.53 (0.38 - 0.99)	0.55 (0.39 - 0.69)	0.92 (0.56 - 1.28)
Bulgaria	Male	66.52 (56.92 - 100.44)	43.5 (34.82 - 60.29)	-34.61	1.25 (1.06 - 1.8)	0.84 (0.66 - 1.13)	-1.66 (-1.81 - -1.51)
Burkina Faso	Male	25.03 (17.68 - 34.06)	34.6 (23.7 - 45.32)	38.23	0.77 (0.53 - 1.09)	0.56 (0.37 - 0.7)	-1.39 (-1.65 - -1.14)
Burundi	Male	36.32 (24.29 - 49.04)	42.16 (29.01 - 56.21)	16.08	1.96 (1.35 - 2.66)	1.15 (0.78 - 1.52)	-2.24 (-2.39 - -2.09)
Cambodia	Male	45.21 (30.61 - 62.37)	45.17 (31.83 - 65.11)	-0.09	1.62 (1.08 - 2.35)	0.8 (0.57 - 1.13)	-2.67 (-2.74 - -2.6)
Cameroon	Male	24.48 (16.94 - 31.19)	46.07 (30.35 - 62.16)	88.19	0.71 (0.5 - 0.89)	0.55 (0.35 - 0.73)	-1.04 (-1.24 - -0.84)
Canada	Male	113.24 (89.16 - 151.18)	99.01 (81.47 - 154.02)	-12.57	0.77 (0.61 - 1.03)	0.39 (0.32 - 0.6)	-2.42 (-3.25 - -1.59)
Cape Verde	Male	0.19 (0.11 - 0.25)	0.26 (0.13 - 0.34)	36.84	0.17 (0.09 - 0.24)	0.12 (0.06 - 0.16)	-1.6 (-1.83 - -1.36)
Central African Republic	Male	9.95 (6.31 - 14.88)	16.53 (11.58 - 23.76)	66.13	1.21 (0.83 - 1.75)	1.1 (0.77 - 1.59)	-0.56 (-0.65 - -0.46)
Chad	Male	14.44 (10.2 - 18.97)	27.78 (19.82 - 36.82)	92.38	0.69 (0.5 - 0.94)	0.61 (0.42 - 0.83)	-0.35 (-0.58 - -0.12)
Chile	Male	46.92 (34.77 - 63.35)	40.69 (32.03 - 57.44)	-13.28	0.88 (0.66 - 1.17)	0.4 (0.31 - 0.56)	-2.66 (-2.91 - -2.42)
China	Male	3662.42 (2049.83 - 4428.46)	1806.09 (1176.96 - 2138.28)	-50.69	0.73 (0.4 - 0.89)	0.2 (0.13 - 0.24)	-5.42 (-5.87 - -4.97)
Colombia	Male	120.81 (90.69 - 156.37)	115.94 (89.74 - 166.59)	-4.03	1.01 (0.78 - 1.31)	0.47 (0.36 - 0.67)	-3.2 (-3.42 - -2.98)
Comoros	Male	2.81 (2.11 - 4.01)	2.92 (2.01 - 4.33)	3.91	1.66 (1.26 - 2.38)	1.04 (0.72 - 1.5)	-1.86 (-2.02 - -1.7)
Congo	Male	7.58 (5.75 - 10.43)	10.38 (6.65 - 16.33)	36.94	1.05 (0.78 - 1.44)	0.64 (0.41 - 1.01)	-2.12 (-2.32 - -1.92)
Costa Rica	Male	18.73 (9.5 - 22.85)	21.3 (12.21 - 26.46)	13.72	1.68 (0.91 - 2)	0.92 (0.53 - 1.15)	-2.52 (-2.8 - -2.23)
Cote d'Ivoire	Male	29.48 (19.47 - 37.78)	51.55 (34.61 - 71.56)	74.86	0.71 (0.5 - 0.92)	0.58 (0.41 - 0.8)	-0.94 (-1.14 - -0.74)
Croatia	Male	30.98 (19.03 - 35.96)	15.11 (11.46 - 20.08)	-51.23	1.15 (0.7 - 1.33)	0.46 (0.36 - 0.62)	-2.97 (-3.19 - -2.75)
Cuba	Male	12.68 (8.25 - 28.7)	56.43 (14.62 - 82.82)	345.03	0.23 (0.15 - 0.53)	0.72 (0.19 - 1.05)	4.34 (2.46 - 6.25)
Cyprus	Male	3.27 (2.12 - 4.29)	3.31 (2.26 - 4.21)	1.22	0.81 (0.53 - 1.06)	0.39 (0.27 - 0.49)	-3 (-3.38 - -2.62)
Czech Republic	Male	103.67 (66.35 - 124.69)	51.81 (42.45 - 75.71)	-50.02	1.84 (1.17 - 2.2)	0.63 (0.52 - 0.96)	-3.73 (-3.92 - -3.54)

Democratic Republic of the Congo	Male	93.35 (63.92 - 138.24)	157.48 (110.63 - 231.82)	68.70	0.83 (0.55 - 1.29)	0.68 (0.46 - 1.05)	-0.69 (-0.78 - -0.61)
Denmark	Male	28.32 (18.72 - 37.32)	22.32 (16.59 - 28.51)	-21.19	0.86 (0.57 - 1.13)	0.48 (0.37 - 0.63)	-2.21 (-2.37 - -2.06)
Djibouti	Male	2.42 (1.35 - 3.58)	4.38 (2.89 - 6.72)	80.99	1.31 (0.85 - 1.9)	0.97 (0.64 - 1.46)	-1.38 (-1.61 - -1.14)
Dominica	Male	0.38 (0.29 - 0.59)	0.32 (0.26 - 0.45)	-15.79	1.18 (0.9 - 1.81)	0.78 (0.64 - 1.09)	-1.74 (-1.98 - -1.51)
Dominican Republic	Male	5.08 (3.73 - 9.94)	14.23 (5.91 - 20.3)	180.12	0.2 (0.15 - 0.4)	0.3 (0.12 - 0.43)	0.38 (-0.43 - 1.19)
Ecuador	Male	33.22 (27.54 - 52.58)	43.57 (35.1 - 66.93)	31.16	0.9 (0.76 - 1.41)	0.58 (0.47 - 0.87)	-1.24 (-1.51 - -0.98)
Egypt	Male	533.4 (378.32 - 733.42)	613.47 (385.09 - 794.15)	15.01	2.63 (1.93 - 3.68)	1.62 (1.01 - 2.08)	-1.5 (-1.64 - -1.35)
El Salvador	Male	26.08 (18.76 - 33.9)	18.01 (12.23 - 27.67)	-30.94	1.48 (0.98 - 1.97)	0.71 (0.48 - 1.06)	-2.92 (-3.1 - -2.73)
Equatorial Guinea	Male	1.69 (1.18 - 2.48)	1.23 (0.81 - 1.85)	-27.22	1.33 (0.98 - 1.91)	0.36 (0.24 - 0.54)	-5.54 (-6.02 - -5.07)
Eritrea	Male	26.55 (12.22 - 39.84)	42.26 (25.83 - 66.72)	59.17	2.92 (1.77 - 4.24)	2.08 (1.31 - 3.19)	-1.64 (-2.02 - -1.26)
Estonia	Male	9.57 (5.79 - 11.36)	4.14 (2.77 - 5.61)	-56.74	1.24 (0.74 - 1.46)	0.49 (0.32 - 0.69)	-3.99 (-4.4 - -3.58)
Ethiopia	Male	629.06 (417.95 - 835.83)	590.16 (360.06 - 1002.84)	-6.18	3.6 (2.43 - 4.82)	1.73 (1.05 - 2.94)	-2.94 (-3.09 - -2.79)
Federated States of Micronesia	Male	0.21 (0.14 - 0.32)	0.18 (0.11 - 0.26)	-14.29	0.61 (0.4 - 0.95)	0.43 (0.27 - 0.62)	-1.25 (-1.31 - -1.18)
Fiji	Male	0.93 (0.6 - 1.18)	1.61 (0.73 - 2.3)	73.12	0.34 (0.23 - 0.43)	0.4 (0.18 - 0.55)	1.18 (0.85 - 1.52)
Finland	Male	23.42 (15.36 - 31.03)	16.86 (13.86 - 23.68)	-28.01	0.83 (0.54 - 1.09)	0.38 (0.32 - 0.55)	-2.65 (-2.95 - -2.35)
France	Male	308.48 (252.89 - 426.79)	212.74 (175.36 - 318.48)	-31.04	0.94 (0.77 - 1.3)	0.44 (0.36 - 0.67)	-2.6 (-2.71 - -2.48)
Gabon	Male	2.67 (1.98 - 3.63)	3.52 (2.47 - 5.02)	31.84	0.81 (0.6 - 1.11)	0.57 (0.41 - 0.81)	-1.17 (-1.4 - -0.95)
Georgia	Male	57.62 (35.65 - 68.85)	32.51 (23.78 - 46.07)	-43.58	2.18 (1.32 - 2.6)	1.44 (1.08 - 2.06)	-1.89 (-2.06 - -1.73)
Germany	Male	486.92 (282.02 - 548.79)	274.43 (220.07 - 409.11)	-43.64	1.02 (0.59 - 1.15)	0.39 (0.31 - 0.62)	-3.58 (-4.02 - -3.14)
Ghana	Male	8.43 (6.22 - 14.07)	14.33 (10.61 - 21.68)	69.99	0.18 (0.13 - 0.29)	0.16 (0.11 - 0.23)	-0.01 (-0.2 - 0.18)
Greece	Male	102.91 (82.51 - 139.84)	103.13 (67.35 - 122.11)	0.21	1.54 (1.27 - 2.15)	1.11 (0.81 - 1.37)	-1.26 (-1.68 - -0.83)
Greenland	Male	0.26 (0.12 - 0.34)	0.14 (0.08 - 0.17)	-46.15	1.11 (0.46 - 1.5)	0.38 (0.25 - 0.46)	-4.53 (-4.74 - -4.32)
Grenada	Male	0.3 (0.22 - 0.38)	0.25 (0.2 - 0.38)	-16.67	0.92 (0.68 - 1.16)	0.37 (0.3 - 0.57)	-3.02 (-3.26 - -2.77)
Guam	Male	0.18 (0.13 - 0.29)	0.27 (0.22 - 0.38)	50.00	0.33 (0.25 - 0.54)	0.31 (0.25 - 0.43)	0.31 (-0.13 - 0.75)
Guatemala	Male	26.41 (21.79 - 37.22)	29.81 (23.16 - 40.47)	12.87	0.99 (0.81 - 1.36)	0.51 (0.38 - 0.65)	-2.65 (-2.95 - -2.36)
Guinea	Male	26.55 (17.86 - 34.56)	33.89 (21.54 - 44.43)	27.65	1.04 (0.74 - 1.37)	0.85 (0.56 - 1.14)	-0.63 (-0.75 - -0.51)
Guinea-Bissau	Male	3.6 (2.33 - 4.96)	4.02 (2.92 - 5.6)	11.67	1.11 (0.75 - 1.5)	0.75 (0.52 - 1)	-1.39 (-1.44 - -1.35)
Guyana	Male	0.88 (0.48 - 2.33)	1.63 (1.04 - 2.09)	85.23	0.34 (0.2 - 0.87)	0.49 (0.32 - 0.62)	1.49 (0.5 - 2.49)
Haiti	Male	40.93 (27.94 - 69.04)	55.46 (32.19 - 90)	35.50	1.94 (1.26 - 3.36)	1.42 (0.82 - 2.34)	-1.08 (-1.15 - -1.01)
Honduras	Male	6.12 (3.96 - 7.76)	8.74 (4.85 - 12.77)	42.81	0.44 (0.25 - 0.58)	0.28 (0.14 - 0.42)	-1.64 (-1.69 - -1.59)
Hungary	Male	80.21 (57.97 - 103.76)	33.63 (27.35 - 52.75)	-58.07	1.35 (0.97 - 1.72)	0.48 (0.4 - 0.76)	-3.84 (-3.97 - -3.71)
Iceland	Male	0.72 (0.55 - 0.97)	0.67 (0.57 - 1.02)	-6.94	0.54 (0.41 - 0.73)	0.29 (0.25 - 0.45)	-2.06 (-2.31 - -1.81)
India	Male	3947.56 (2548.37 - 4703.72)	3401.06 (2664.54 - 4596.75)	-13.84	1.13 (0.73 - 1.32)	0.56 (0.43 - 0.75)	-2.58 (-2.72 - -2.44)
Indonesia	Male	581.82 (396.97 - 797.73)	636.03 (454.59 - 910.94)	9.32	0.86 (0.58 - 1.24)	0.56 (0.4 - 0.8)	-1.46 (-1.6 - -1.31)
Iran	Male	107.65 (80.9 - 196.81)	173.95 (107.12 - 200.51)	61.59	0.54 (0.41 - 0.96)	0.45 (0.27 - 0.52)	0.53 (-0.05 - 1.11)
Iraq	Male	54.16 (30.74 - 71.29)	44.1 (34.32 - 70.68)	-18.57	0.94 (0.57 - 1.21)	0.27 (0.21 - 0.43)	-4.98 (-5.85 - -4.09)
Ireland	Male	18.1 (12.67 - 21.52)	12.4 (9.34 - 17.91)	-31.49	0.97 (0.68 - 1.15)	0.4 (0.3 - 0.57)	-3.26 (-3.38 - -3.14)
Israel	Male	17.8 (14.66 - 24.65)	21.41 (16.96 - 33.23)	20.28	0.78 (0.64 - 1.08)	0.45 (0.35 - 0.69)	-2.49 (-2.82 - -2.15)
Italy	Male	380.68 (288.46 - 485.43)	268.39 (195.21 - 375.06)	-29.50	1.07 (0.8 - 1.35)	0.53 (0.39 - 0.76)	-2.19 (-2.46 - -1.92)
Jamaica	Male	4.1 (2.03 - 5.19)	4.81 (2.37 - 6.76)	17.32	0.44 (0.23 - 0.55)	0.34 (0.17 - 0.47)	-1.12 (-1.6 - -0.63)
Japan	Male	124.65 (98.9 - 168.81)	156.21 (105.84 - 179.43)	25.32	0.17 (0.13 - 0.23)	0.12 (0.09 - 0.14)	-0.72 (-0.89 - -0.55)
Jordan	Male	3.79 (2.45 - 5.02)	5.62 (4 - 7.1)	48.28	0.32 (0.19 - 0.42)	0.14 (0.1 - 0.18)	-3.11 (-3.23 - -2.98)
Kazakhstan	Male	58.52 (41.6 - 84.93)	45.83 (36.25 - 62.48)	-21.68	0.88 (0.62 - 1.3)	0.56 (0.44 - 0.76)	-1.86 (-2.31 - -1.4)
Kenya	Male	49.41 (37.77 - 72.93)	114.21 (80.51 - 141.72)	131.15	0.66 (0.53 - 0.98)	0.71 (0.49 - 0.87)	0.48 (0.17 - 0.79)
Kiribati	Male	0.08 (0.06 - 0.12)	0.13 (0.09 - 0.17)	62.50	0.31 (0.25 - 0.45)	0.29 (0.21 - 0.38)	-0.34 (-0.49 - -0.18)
Kuwait	Male	2.6 (1.6 - 3.11)	3.29 (2.64 - 4.46)	26.54	0.38 (0.25 - 0.44)	0.17 (0.14 - 0.24)	-2.52 (-3.01 - -2.03)
Kyrgyzstan	Male	11.74 (6.91 - 14.22)	8.52 (6.77 - 12.24)	-27.43	0.73 (0.43 - 0.87)	0.35 (0.28 - 0.48)	-2.34 (-2.62 - -2.06)
Laos	Male	23.89 (15.78 - 32.15)	19.95 (12.95 - 32)	-16.49	1.65 (1.08 - 2.34)	0.79 (0.5 - 1.28)	-2.83 (-2.94 - -2.72)
Latvia	Male	14.43 (9.02 - 17.44)	7.89 (4.55 - 10.06)	-45.32	1.06 (0.66 - 1.27)	0.64 (0.37 - 0.82)	-2.11 (-2.37 - -1.86)
Lebanon	Male	35.11 (25.31 - 49.09)	40.6 (26.44 - 50.24)	15.64	2.55 (1.86 - 3.55)	1.16 (0.75 - 1.41)	-2.83 (-3.04 - -2.62)
Lesotho	Male	4.85 (3.78 - 6.82)	6.44 (4.23 - 8.98)	32.78	0.87 (0.68 - 1.23)	1 (0.68 - 1.36)	1.09 (0.67 - 1.5)
Liberia	Male	5.66 (3.94 - 7.28)	6.42 (4.48 - 8.91)	13.43	0.67 (0.47 - 0.89)	0.45 (0.3 - 0.61)	-1.8 (-2.1 - -1.49)
Libya	Male	28.23 (19.48 - 39.89)	40.35 (24.65 - 55.23)	42.93	1.91 (1.34 - 2.77)	1.32 (0.82 - 1.78)	-1.36 (-1.55 - -1.18)
Lithuania	Male	19.13 (12.25 - 22.79)	10.52 (7.76 - 13.02)	-45.01	1.03 (0.66 - 1.22)	0.58 (0.43 - 0.72)	-2.57 (-2.89 - -2.24)
Luxembourg	Male	1.72 (1.15 - 2.27)	1.3 (0.99 - 2.28)	-24.42	0.76 (0.51 - 1)	0.32 (0.24 - 0.58)	-3.44 (-3.66 - -3.23)
Macedonia	Male	11.31 (5.86 - 14.69)	9.37 (5.97 - 11.82)	-17.15	1.13 (0.61 - 1.45)	0.63 (0.4 - 0.8)	-2.85 (-3.28 - -2.42)
Madagascar	Male	82.21 (62.78 - 110.91)	95.16 (65.41 - 131.97)	15.75	1.6 (1.23 - 2.19)	1.06 (0.73 - 1.44)	-1.63 (-1.87 - -1.4)
Malawi	Male	29.77 (10.17 - 45.2)	41.44 (29.19 - 54.92)	39.20	0.86 (0.39 - 1.27)	0.72 (0.51 - 0.91)	-1.11 (-1.46 - -0.76)
Malaysia	Male	45.69 (28.93 - 59.94)	55.91 (30.52 - 74.46)	22.37	0.73 (0.49 - 0.94)	0.38 (0.21 - 0.51)	-2.47 (-2.69 - -2.26)
Maldives	Male	0.32 (0.25 - 0.45)	0.24 (0.18 - 0.33)	-25.00	0.46 (0.35 - 0.61)	0.12 (0.1 - 0.17)	-5.08 (-5.23 - -4.94)
Mali	Male	49.8 (31.68 - 63.91)	55.75 (39.19 - 75.01)	11.95	1.44 (1.02 - 1.82)	0.74 (0.55 - 1.04)	-2.38 (-2.64 - -2.12)
Malta	Male	1.96 (1.59 - 2.69)	2.03 (1.67 - 2.88)	3.57	1.01 (0.82 - 1.38)	0.58 (0.49 - 0.84)	-2.06 (-2.19 - -1.93)
Marshall Islands	Male	0.08 (0.06 - 0.1)	0.11 (0.07 - 0.14)	37.50	0.62 (0.47 - 0.79)	0.51 (0.33 - 0.65)	-0.58 (-0.73 - -0.42)
Mauritania	Male	4.47 (3.22 - 5.7)	4.81 (3.27 - 7.29)	7.61	0.64 (0.47 - 0.82)	0.37 (0.26 - 0.56)	-1.94 (-2.06 - -1.82)
Mauritius	Male	2.46 (1.62 - 2.87)	2.15 (1.34 - 2.68)	-12.60	0.54 (0.37 - 0.65)	0.28 (0.18 - 0.35)	-2.83 (-3.12 - -2.55)
Mexico	Male	330.06 (240.22 - 405.35)	367.42 (282.79 - 518.04)	11.32	1.17 (0.86 - 1.45)	0.66 (0.5 - 0.92)	-2.17 (-2.45 - -1.89)
Moldova	Male	33.64 (23.33 - 50.87)	18.72 (15.7 - 28.35)	-44.35	1.64 (1.15 - 2.47)	0.86 (0.72 - 1.3)	-2.88 (-3.16 - -2.59)
Mongolia	Male	2.55 (1.59 - 3.38)	2.93 (1.95 - 3.7)	14.90	0.42 (0.22 - 0.56)	0.23 (0.14 - 0.29)	-2.88 (-3.21 - -2.55)
Montenegro	Male	5.65 (4.48 - 7.7)	4.76 (3.67 - 6.89)	-15.75	1.86 (1.49 - 2.55)	1.14 (0.89 - 1.65)	-2.23 (-2.54 - -1.93)
Morocco	Male	194.67 (128.59 - 246.65)	201.5 (135.33 - 272.17)	3.51	1.91 (1.33 - 2.51)	1.19 (0.81 - 1.59)	-1.77 (-1.88 - -1.66)
Mozambique	Male	110.13 (83.29 - 160.72)	152.09 (104.22 - 200.4)	38.10	2.1 (1.65 - 3.16)	1.59 (1.05 - 2.11)	-0.84 (-1.02 - -0.66)
Myanmar	Male	241.07 (166.08 - 330.48)	176.02 (122.05 - 251.3)	-26.98	1.63 (1.11 - 2.29)	0.79 (0.55 - 1.14)	-2.69 (-2.9 - -2.48)
Namibia	Male	5.64 (4.19 - 7.31)	6.13 (4.1 - 8)	8.69	1.24 (0.98 - 1.75)	0.76 (0.55 - 1.01)	-1.93 (-2.36 - -1.49)
Nepal	Male	107.41 (70.68 - 179.1)	75.73 (43.84 - 149.1)	-29.49	1.53 (1.01 - 2.57)	0.65 (0.38 - 1.28)	-3.2 (-3.51 - -2.9)
Netherlands	Male	71.01 (48.9 - 82.98)	53.61 (40.35 - 70.15)	-24.50	0.83 (0.57 - 0.97)	0.4 (0.3 - 0.53)	-2.6 (-2.7 - -2.5)
New Zealand	Male	9.9 (4.7 - 11.6)	9.71 (5.25 - 11.78)	-1.92	0.56 (0.26 - 0.66)	0.32 (0.18 - 0.38)	-2.02 (-2.19 - -1.86)
Nicaragua	Male	7.05 (4.49 - 8.69)	7.62 (4.68 - 9.63)	8.09	0.59 (0.37 - 0.76)	0.32 (0.19 - 0.41)	-2.48 (-2.64 - -2.31)
Niger	Male	25.85 (16.66 - 35.25)	31.4 (22.63 - 44.23)	21.47	0.86 (0.61 - 1.15)	0.53 (0.37 - 0.72)	-2.12 (-2.3 - -1.93)
Nigeria	Male	1033.64 (703.63 - 1392.36)	1300.35 (900 - 1836.49)	25.80	2.81 (2.04 - 3.82)	1.8 (1.32 - 2.64)	-2.06 (-2.23 - -1.88)
North Korea	Male	34.11 (21.68 - 47.81)	54.48 (35.73 - 71.59)	59.72	0.45 (0.29 - 0.62)	0.41 (0.27 - 0.54)	-0.21 (-0.49 - 0.08)
Northern Mariana Islands	Male	0.03 (0.02 - 0.05)	0.04 (0.03 - 0.05)	33.33	0.18 (0.13 - 0.26)	0.15 (0.1 - 0.19)	0.02 (-0.3 - 0.33)
Norway	Male	17.39 (14.08 - 25.29)	12.11 (10.47 - 18.67)	-30.36	0.64 (0.53 - 0.94)	0.31 (0.27 - 0.48)	-2.56 (-2.7 - -2.42)
Oman	Male	8.35 (5.08 - 11.25)	10.62 (6.28 - 14.41)	27.19	1.26 (0.8 - 1.67)	0.54 (0.3 - 0.72)	-2.95 (-3.33 - -2.57)
Pakistan	Male	1173.65 (835.93 - 1618.29)	1728.58 (1252.65 - 2349.53)	47.28	2.53 (1.84 - 3.61)	1.96 (1.43 - 2.67)	-1.16 (-1.36 - -0.96)
Palestine	Male	4.53 (2.62 - 9.17)	9.25 (7.08 - 16.72)	104.19	0.77 (0.47 - 1.5)	0.56 (0.44 - 0.92)	-1.17 (-1.46 - -0.88)
Panama	Male	3.58 (2.78 - 4.64)	6.2 (3.66 - 7.53)	73.18	0.39 (0.31 - 0.51)	0.32 (0.19 - 0.39)	-0.33 (-0.68 - 0.03)
Papua New Guinea	Male	12.45 (9.04 - 16.74)	23.7 (15.51 - 31.83)	90.36	0.83 (0.6 - 1.15)	0.67 (0.44 - 0.91)	-0.48 (-0.62 - -0.34)
Paraguay	Male	10.03 (6.94 - 14.88)	16.43 (10.88 - 21.9)	63.81	0.71 (0.51 - 1.03)	0.56 (0.36 - 0.76)	-0.68 (-0.78 - -0.58)

Peru	Male	50.51 (40.19 - 71.83)	45.66 (31.4 - 59.95)	-9.60	0.64 (0.49 - 0.9)	0.3 (0.2 - 0.39)	-2.8 (-2.9 - -2.69)
Philippines	Male	104.84 (86.22 - 164.01)	113.19 (73.33 - 240.36)	7.96	0.47 (0.39 - 0.69)	0.28 (0.19 - 0.56)	-1.98 (-2.14 - -1.82)
Poland	Male	339.08 (214.8 - 378.98)	170.01 (132.21 - 221.06)	-49.86	1.71 (1.09 - 1.9)	0.64 (0.5 - 0.83)	-3.98 (-4.2 - -3.76)
Portugal	Male	53.3 (42.64 - 92.79)	37.44 (29.59 - 64.1)	-29.76	0.94 (0.76 - 1.61)	0.45 (0.36 - 0.76)	-3 (-3.11 - -2.89)
Puerto Rico	Male	14.77 (10.05 - 17.82)	13.5 (7.76 - 16.18)	-8.60	0.85 (0.58 - 1.03)	0.54 (0.32 - 0.65)	-1.85 (-2.13 - -1.57)
Qatar	Male	0.5 (0.35 - 0.85)	2.4 (1.38 - 3.85)	380.00	0.33 (0.24 - 0.53)	0.17 (0.11 - 0.24)	-2.41 (-2.66 - -2.16)
Romania	Male	143.94 (88.57 - 163.81)	77.38 (57.92 - 98.47)	-46.24	1.14 (0.71 - 1.29)	0.57 (0.41 - 0.71)	-2.99 (-3.17 - -2.8)
Russian Federation	Male	851.8 (501.9 - 1043.54)	519.79 (401.81 - 687.98)	-38.98	1.17 (0.7 - 1.43)	0.62 (0.48 - 0.82)	-2.75 (-3.2 - -2.3)
Rwanda	Male	46.16 (31.73 - 61.98)	38.06 (26.33 - 53.61)	-17.55	1.86 (1.35 - 2.53)	0.95 (0.68 - 1.28)	-3.22 (-3.61 - -2.82)
Saint Lucia	Male	0.57 (0.48 - 0.87)	0.78 (0.59 - 1.05)	36.84	1.19 (0.99 - 1.79)	0.79 (0.6 - 1.06)	-1.53 (-1.77 - -1.28)
Saint Vincent and the Grenadines	Male	0.21 (0.11 - 0.58)	0.52 (0.39 - 0.67)	147.62	0.53 (0.28 - 1.43)	0.79 (0.59 - 1.02)	0.48 (-0.23 - 1.19)
Samoa	Male	0.44 (0.3 - 0.56)	0.38 (0.26 - 0.5)	-13.64	0.76 (0.55 - 1.01)	0.5 (0.36 - 0.67)	-1.64 (-1.78 - -1.51)
Sao Tome and Principe	Male	0.05 (0.04 - 0.1)	0.07 (0.04 - 0.12)	40.00	0.13 (0.1 - 0.22)	0.1 (0.07 - 0.16)	-0.74 (-0.8 - -0.69)
Saudi Arabia	Male	55.57 (20.56 - 88.01)	89.19 (25.41 - 140.24)	60.50	0.95 (0.39 - 1.51)	0.6 (0.18 - 0.9)	-0.97 (-1.36 - -0.58)
Senegal	Male	18.75 (13.4 - 23.8)	27.23 (19.03 - 38.44)	45.23	0.72 (0.53 - 0.91)	0.59 (0.41 - 0.81)	-0.68 (-0.74 - -0.61)
Serbia	Male	75.19 (47.05 - 99.41)	49.76 (30.17 - 61.17)	-33.82	1.41 (0.89 - 1.84)	0.78 (0.48 - 0.98)	-2.14 (-2.36 - -1.92)
Seychelles	Male	0.23 (0.18 - 0.33)	0.24 (0.18 - 0.31)	4.35	0.82 (0.64 - 1.13)	0.44 (0.32 - 0.57)	-2.6 (-2.72 - -2.47)
Sierra Leone	Male	10.47 (6.99 - 14.22)	12.63 (8.64 - 16.42)	20.63	0.69 (0.47 - 0.92)	0.49 (0.33 - 0.64)	-1.26 (-1.33 - -1.2)
Singapore	Male	4.11 (3.29 - 6.05)	4.83 (3.76 - 6.37)	17.52	0.31 (0.25 - 0.47)	0.15 (0.12 - 0.2)	-2.15 (-2.39 - -1.91)
Slovakia	Male	30.17 (21.96 - 37.18)	21.7 (14.88 - 26.76)	-28.07	1.14 (0.82 - 1.4)	0.6 (0.41 - 0.72)	-2 (-2.14 - -1.87)
Slovenia	Male	10.84 (7.62 - 13.41)	7.59 (5.4 - 9.55)	-29.98	1.06 (0.73 - 1.28)	0.47 (0.34 - 0.6)	-3.25 (-3.4 - -3.09)
Solomon Islands	Male	0.69 (0.47 - 1.01)	1.15 (0.72 - 1.72)	66.67	0.63 (0.43 - 0.91)	0.51 (0.32 - 0.78)	-0.44 (-0.61 - -0.27)
Somalia	Male	43.48 (13.76 - 75.15)	102.74 (60.4 - 171.09)	136.29	1.72 (0.77 - 2.83)	1.78 (1.1 - 2.8)	-0.24 (-0.51 - 0.04)
South Africa	Male	59.07 (43.92 - 75.33)	83.14 (57.02 - 101.25)	40.75	0.49 (0.35 - 0.64)	0.38 (0.26 - 0.46)	-1.06 (-1.63 - -0.49)
South Korea	Male	30.15 (23.98 - 54.42)	42 (23.51 - 54.03)	39.30	0.19 (0.15 - 0.34)	0.12 (0.07 - 0.15)	-1.23 (-1.43 - -1.03)
South Sudan	Male	28.19 (11.02 - 47.52)	45.91 (30.57 - 64.73)	62.86	1.3 (0.66 - 2.07)	1.33 (0.9 - 1.85)	-0.17 (-0.38 - 0.05)
Spain	Male	230.38 (171.8 - 287.34)	154.17 (119.27 - 216.06)	-33.08	1.01 (0.73 - 1.24)	0.43 (0.34 - 0.61)	-3.12 (-3.23 - -3.01)
Sri Lanka	Male	79.75 (63.49 - 108.69)	61.15 (42.52 - 80.8)	-23.32	1.22 (0.95 - 1.62)	0.54 (0.38 - 0.71)	-3.23 (-3.6 - -2.85)
Sudan	Male	90.7 (55.44 - 151.4)	105.52 (71.36 - 157.88)	16.34	1.34 (0.83 - 2.22)	0.79 (0.51 - 1.22)	-1.84 (-1.91 - -1.77)
Suriname	Male	1.33 (1.08 - 2.01)	2.21 (1.63 - 2.89)	66.17	0.85 (0.7 - 1.28)	0.78 (0.58 - 1.02)	-0.28 (-0.66 - 0.11)
Swaziland	Male	1.66 (1.27 - 2.28)	2.81 (1.83 - 3.76)	69.28	0.87 (0.67 - 1.21)	0.83 (0.56 - 1.11)	0.6 (-0.08 - 1.29)
Sweden	Male	29.49 (21.96 - 38.31)	21.1 (16.24 - 26.95)	-28.45	0.49 (0.38 - 0.65)	0.26 (0.21 - 0.36)	-1.99 (-2.19 - -1.79)
Switzerland	Male	23.05 (14.65 - 29.63)	19.8 (13.28 - 26.75)	-14.10	0.54 (0.34 - 0.7)	0.29 (0.2 - 0.42)	-2.15 (-2.25 - -2.06)
Syria	Male	7.25 (5.12 - 9.16)	9.53 (4.89 - 13.48)	31.45	0.2 (0.13 - 0.25)	0.13 (0.06 - 0.19)	-1.61 (-1.83 - -1.39)
Tajikistan	Male	5.01 (3.75 - 6.99)	8.03 (5.96 - 10.21)	60.28	0.32 (0.2 - 0.44)	0.26 (0.17 - 0.33)	-1.08 (-1.29 - -0.86)
Tanzania	Male	105.65 (46.34 - 160.03)	181.56 (125.51 - 268.95)	71.85	1.16 (0.64 - 1.78)	0.96 (0.67 - 1.37)	-0.86 (-0.99 - -0.73)
Thailand	Male	166.16 (133.32 - 240.2)	136.4 (104.74 - 205.09)	-17.91	0.76 (0.61 - 1.09)	0.31 (0.24 - 0.48)	-3.86 (-4.1 - -3.63)
The Bahamas	Male	0.61 (0.46 - 0.78)	0.79 (0.58 - 1.11)	29.51	0.64 (0.49 - 0.83)	0.42 (0.31 - 0.59)	-1.93 (-2.16 - -1.7)
The Gambia	Male	2.57 (1.76 - 3.61)	4.25 (2.88 - 6.71)	65.37	0.78 (0.55 - 1.11)	0.61 (0.44 - 0.93)	-0.63 (-0.74 - -0.53)
Timor-Leste	Male	3 (1.86 - 4.78)	3.43 (1.91 - 5.99)	14.33	1.17 (0.68 - 2)	0.72 (0.4 - 1.26)	-1.92 (-2.03 - -1.81)
Togo	Male	7.59 (5.4 - 9.86)	11.67 (7.92 - 15.8)	53.75	0.67 (0.49 - 0.87)	0.53 (0.35 - 0.7)	-0.93 (-1.11 - -0.75)
Tonga	Male	0.09 (0.07 - 0.14)	0.12 (0.08 - 0.17)	33.33	0.3 (0.21 - 0.43)	0.28 (0.18 - 0.4)	0.02 (-0.1 - 0.14)
Trinidad and Tobago	Male	2.2 (1.77 - 2.97)	3.38 (1.96 - 4.66)	53.64	0.45 (0.36 - 0.61)	0.41 (0.24 - 0.55)	-0.04 (-0.35 - 0.27)
Tunisia	Male	34.91 (26.3 - 50.92)	40.57 (25.91 - 55.98)	16.21	1.05 (0.81 - 1.59)	0.68 (0.44 - 0.93)	-1.42 (-1.65 - -1.19)
Turkey	Male	225.88 (155.27 - 404.16)	177.65 (130.68 - 295.19)	-21.35	1.02 (0.69 - 1.85)	0.43 (0.32 - 0.73)	-3.4 (-3.69 - -3.1)
Turkmenistan	Male	9.38 (5.83 - 11.55)	13.61 (7.71 - 16.89)	45.10	0.68 (0.47 - 0.82)	0.58 (0.35 - 0.71)	-0.27 (-0.68 - 0.14)
Uganda	Male	66.36 (45.82 - 98.67)	142.88 (92.43 - 199.26)	115.31	1.07 (0.73 - 1.76)	1.21 (0.8 - 1.62)	0.33 (0.07 - 0.6)
Ukraine	Male	280.31 (194.66 - 390.35)	304.67 (245.8 - 456.3)	8.69	1.04 (0.73 - 1.43)	1.19 (0.95 - 1.86)	-0.14 (-0.62 - 0.34)
United Arab Emirates	Male	6.08 (3.89 - 10.63)	29.42 (19.23 - 45.92)	383.88	0.81 (0.57 - 1.34)	0.56 (0.34 - 0.95)	-1.24 (-1.36 - -1.11)
United Kingdom	Male	319.89 (238.91 - 403.33)	222.3 (165.42 - 275.44)	-30.51	0.9 (0.66 - 1.1)	0.45 (0.34 - 0.57)	-2.6 (-2.73 - -2.47)
United States	Male	944.06 (624.25 - 1056.56)	771.61 (655.92 - 1112.08)	-18.27	0.69 (0.46 - 0.77)	0.36 (0.31 - 0.54)	-2.44 (-2.59 - -2.29)
Uruguay	Male	21.05 (14.43 - 24.82)	15.45 (10.2 - 19.48)	-26.60	1.23 (0.85 - 1.45)	0.76 (0.5 - 0.95)	-1.69 (-1.82 - -1.55)
Uzbekistan	Male	62.23 (46.87 - 100.93)	81.83 (64.66 - 125.18)	31.50	0.84 (0.59 - 1.52)	0.64 (0.5 - 1.02)	-0.94 (-1.08 - -0.8)
Vanuatu	Male	0.44 (0.23 - 0.86)	0.94 (0.41 - 1.91)	113.64	0.86 (0.44 - 1.65)	0.87 (0.38 - 1.76)	0.23 (0.07 - 0.39)
Venezuela	Male	58.96 (44.13 - 74.56)	93.5 (57.21 - 121.93)	58.58	0.96 (0.72 - 1.2)	0.66 (0.41 - 0.86)	-1.35 (-1.49 - -1.22)
Vietnam	Male	203.34 (150.13 - 286.23)	255.35 (147.37 - 335.38)	25.58	0.91 (0.69 - 1.34)	0.59 (0.34 - 0.76)	-1.32 (-1.48 - -1.16)
Virgin Islands, U.S.	Male	0.16 (0.12 - 0.27)	0.29 (0.2 - 0.37)	81.25	0.35 (0.27 - 0.61)	0.38 (0.26 - 0.48)	0.67 (0.26 - 1.08)
Yemen	Male	57.6 (23.93 - 100.81)	89.61 (62.6 - 142.68)	55.57	1.48 (0.73 - 2.68)	0.98 (0.66 - 1.57)	-1.68 (-1.78 - -1.58)
Zambia	Male	44.23 (25.35 - 62.84)	70.27 (47.29 - 100.96)	58.87	1.6 (1.04 - 2.27)	1.22 (0.84 - 1.66)	-1.46 (-1.91 - -1.01)
Zimbabwe	Male	17.28 (11.73 - 22.67)	45.86 (24.32 - 61.73)	165.39	0.56 (0.43 - 0.84)	0.96 (0.6 - 1.23)	2.97 (1.86 - 4.09)

ASDR, age standardized death rate; CI, confidence interval; EAPC, estimated annual percentage change; UI, uncertainty interval.

Supplementary Table S5. The DALY and age-standardized DALY rate of Hodgkin lymphoma in 1990 and 2017, and its temporal trends from 1990 to 2017.

Nation	Sex	DALY No. (95% UI) 1990	DALY No. (95% UI) 2017	Change in absolute number(%)	Age Standardized DALY Rate per 100,000 No. (9 1990	Age Standardized DALY Rate per 100,000 No. (9 2017	1990-2017EAPC No. (95 CI)
Afghanistan	Both	6200.71 (2041.21 - 9979.04)	14963.85 (10626.83 - 20519.05)	141.32	73.97 (25.69 - 117.66)	59.19 (42.46 - 81.21)	-0.82 (-1.2 - -0.45)
Albania	Both	934.79 (750.19 - 1498.91)	571.91 (406.67 - 956)	-38.82	30.4 (24.66 - 50.17)	18.31 (13.14 - 29.78)	-1.99 (-2.16 - -1.81)
Algeria	Both	14115.55 (9646.81 - 17602.77)	13626.72 (9710.42 - 16279.34)	-3.46	59.1 (43.34 - 71.86)	33.73 (24.26 - 40.29)	-1.96 (-2.09 - -1.83)
American Samoa	Both	2.18 (1.68 - 3.12)	2.45 (2.04 - 3.18)	12.39	5.64 (4.38 - 8.03)	4.78 (3.98 - 6.18)	-0.36 (-0.61 - -0.11)
Andorra	Both	12.11 (9.28 - 15.41)	12.12 (9.78 - 16.76)	0.08	19.07 (14.73 - 24.2)	12.38 (9.96 - 17.17)	-1.58 (-1.74 - -1.42)
Angola	Both	2691.71 (1538.38 - 4068.92)	3602.81 (2808.9 - 4798.32)	33.85	31.5 (20.42 - 44.65)	16.89 (12.91 - 22.81)	-2.4 (-2.64 - -2.15)
Antigua and Barbuda	Both	4.14 (3.44 - 6.07)	5.33 (4.38 - 6.98)	28.74	7.27 (6.05 - 10.54)	5.54 (4.55 - 7.22)	-0.87 (-1.05 - -0.69)
Argentina	Both	8702.25 (7503.6 - 11541.08)	7527.95 (6103.72 - 10977.21)	-13.49	26.47 (22.78 - 35.03)	15.73 (12.74 - 23.05)	-2.07 (-2.22 - -1.92)
Armenia	Both	180.21 (134.31 - 326)	275.46 (151.09 - 335.91)	52.86	5.46 (4.13 - 9.69)	7.73 (4.31 - 9.38)	1.43 (0.57 - 2.29)
Australia	Both	3077.65 (2369.59 - 3437.83)	2803.99 (2266.86 - 3408.9)	-8.89	16.67 (12.84 - 18.55)	9.91 (7.97 - 12.2)	-1.77 (-1.99 - -1.56)
Austria	Both	2705.9 (1674.63 - 3109.92)	1063.21 (867.25 - 1659.78)	-60.71	28.62 (18.27 - 32.71)	9.2 (7.32 - 14.9)	-4.57 (-5.09 - -4.06)
Azerbaijan	Both	2545.79 (1635.23 - 3184.56)	2591.2 (1678.3 - 3354.13)	1.78	34.95 (23.22 - 43.06)	23.8 (15.36 - 30.44)	-1.48 (-1.76 - -1.2)
Bahrain	Both	129.36 (64.24 - 176.23)	133.29 (95.23 - 164.46)	3.04	34.79 (16.25 - 47.05)	9.32 (6.52 - 11.23)	-5.95 (-6.46 - -5.44)
Bangladesh	Both	57267.08 (39925.34 - 85684.12)	25073.14 (17293 - 43168.72)	-56.22	56.85 (40.76 - 87.51)	15.87 (11 - 27.45)	-4.8 (-4.88 - -4.71)
Barbados	Both	51.89 (43.13 - 63.75)	43.32 (36.32 - 56.35)	-16.52	19.28 (15.99 - 23.51)	12.59 (10.64 - 16.22)	-1.77 (-1.85 - -1.7)
Belarus	Both	4023.26 (2588.17 - 4798.5)	2429.38 (1725.02 - 2981.92)	-39.62	36.08 (23.35 - 43.01)	21.65 (15.21 - 26.78)	-1.69 (-2.02 - -1.36)
Belgium	Both	2921.09 (2271.42 - 3333.39)	1650.6 (1378.18 - 2444.16)	-43.49	24.36 (19.37 - 28.58)	11.59 (9.5 - 17.63)	-2.66 (-2.75 - -2.57)
Belize	Both	33.76 (26.58 - 43.42)	64.61 (50.66 - 78.93)	91.38	21.75 (17.39 - 27.9)	17.24 (13.99 - 21.36)	-1.2 (-1.46 - -0.94)
Benin	Both	919.4 (655.22 - 1180.3)	1254.52 (968.9 - 1661.6)	36.45	21.13 (15.92 - 25.98)	13.43 (10.49 - 17.26)	-1.79 (-1.93 - -1.66)
Bermuda	Both	10.96 (7.38 - 21.36)	12.61 (9.63 - 15.38)	15.05	17.09 (11.59 - 33)	15.37 (11.41 - 19.56)	-0.93 (-1.39 - -0.45)
Bhutan	Both	253.65 (182.21 - 380.13)	150.27 (81.88 - 293.63)	-40.76	48.72 (34.34 - 73.46)	16.04 (9.06 - 31.15)	-4.28 (-4.39 - -4.16)
Bolivia	Both	2730.43 (1754.11 - 3428.27)	2117.01 (1511.68 - 2894.42)	-22.47	47.63 (31.52 - 57.64)	20.21 (14.6 - 27.45)	-3.23 (-3.26 - -3.19)
Bosnia and Herzegovina	Both	1011.4 (758 - 1168.18)	650.09 (384.11 - 806.09)	-35.72	21.27 (15.8 - 24.52)	14.76 (8.98 - 18.33)	-1.67 (-1.93 - -1.41)
Botswana	Both	154.85 (110.43 - 224.21)	167.14 (131.83 - 231.92)	7.94	15.97 (11.57 - 22.98)	8.22 (6.64 - 11.19)	-2.22 (-2.46 - -1.97)
Brazil	Both	30244.87 (25854.53 - 38078.77)	27567.3 (22736.8 - 34508.44)	-8.85	22.54 (18.91 - 27.86)	12.14 (10.04 - 15.24)	-2.19 (-2.24 - -2.13)
Brunei	Both	28.95 (18.96 - 51.94)	81.4 (59.63 - 97.96)	181.17	14.45 (9.47 - 26.28)	18.65 (13.63 - 22.16)	2.03 (1.56 - 2.5)
Bulgaria	Both	3580.53 (3007.96 - 4649.82)	2121.95 (1770.29 - 2613.69)	-40.74	36.26 (29.63 - 44.93)	24.74 (20.11 - 29.43)	-1.69 (-1.83 - -1.55)
Burkina Faso	Both	2005.99 (1475.46 - 2555.43)	2557.15 (1914.94 - 3297.98)	27.48	23.33 (17.83 - 29.66)	14.58 (11 - 18.22)	-2.01 (-2.28 - -1.75)
Burundi	Both	3080.25 (2016.76 - 3982.16)	3322.37 (2370.53 - 4239.74)	7.86	60.59 (40.56 - 77.14)	34.17 (24.15 - 42.95)	-2.42 (-2.59 - -2.25)
Cambodia	Both	3132.15 (2198.26 - 3853.24)	2685.19 (2024.76 - 3629.91)	-14.27	38.93 (27.94 - 49.29)	18.19 (13.76 - 24.35)	-2.96 (-3.06 - -2.86)
Cameroon	Both	1836.65 (1305.19 - 2250.76)	3207.97 (2343.96 - 4131.17)	74.66	20.23 (14.82 - 24.22)	14.17 (10.26 - 17.79)	-1.51 (-1.73 - -1.3)
Canada	Both	6745.7 (5290.92 - 7788.02)	5322.4 (4571.79 - 7303.47)	-21.10	22.32 (17.31 - 25.41)	12.78 (10.94 - 17.33)	-1.99 (-2.48 - -1.49)
Cape Verde	Both	11.68 (9.35 - 14.46)	15.85 (10.95 - 19.1)	35.70	4.06 (2.94 - 4.84)	3.05 (2.04 - 3.69)	-1.26 (-1.41 - -1.11)
Central African Republic	Both	703.56 (422.15 - 1019.5)	1134.06 (848.05 - 1553.2)	61.19	32.35 (21.84 - 44.92)	29.57 (22.54 - 40.11)	-0.54 (-0.65 - -0.44)
Chad	Both	1061.68 (754.13 - 1345.42)	1979.78 (1524.97 - 2524.32)	86.48	19.43 (14.85 - 23.99)	16.43 (12.46 - 20.79)	-0.56 (-0.78 - -0.35)
Chile	Both	2880.48 (2324.77 - 3563.58)	2228.27 (1870.01 - 2883.69)	-22.64	22.83 (18.47 - 28.19)	10.97 (9.17 - 14.16)	-2.44 (-2.73 - -2.15)
China	Both	255898.87 (141523.67 - 313742.16)	90898.14 (64616.49 - 105927.28)	-64.48	22.55 (12.58 - 27.66)	5.46 (3.98 - 6.37)	-6.13 (-6.71 - -5.55)
Colombia	Both	8030.51 (6437.03 - 10218.32)	6614.79 (5577.52 - 8893.71)	-17.63	27.39 (22.3 - 34.21)	12.68 (10.68 - 17.13)	-3.21 (-3.41 - -3.01)
Comoros	Both	251.37 (193.86 - 331.66)	235.65 (173.08 - 325.78)	-6.25	58.23 (45.09 - 75.47)	34.11 (25.21 - 46.47)	-2.06 (-2.19 - -1.94)
Congo	Both	543.28 (404.89 - 761.66)	754.07 (533.82 - 1044.4)	38.80	28.2 (21.41 - 37.7)	18.23 (13.11 - 24.94)	-1.97 (-2.22 - -1.72)
Costa Rica	Both	1313.35 (606.01 - 1621.02)	1218.95 (818.06 - 1479.32)	-7.19	47.68 (24 - 57.6)	24.59 (16.74 - 29.7)	-2.62 (-2.92 - -2.32)
Cote d'Ivoire	Both	2383.75 (1605.66 - 3027.74)	4015.66 (2687.59 - 5524.93)	68.46	22.21 (15.91 - 27.3)	17.87 (12.66 - 23.71)	-0.99 (-1.16 - -0.82)
Croatia	Both	1725.64 (1251.88 - 1932.55)	737.78 (624.73 - 917.85)	-57.25	30.44 (22.03 - 34.09)	13.29 (11.33 - 17.1)	-2.57 (-2.83 - -2.31)
Cuba	Both	654.05 (416.85 - 1497.53)	2981.49 (856.42 - 4103.54)	355.85	5.78 (3.74 - 13.11)	21.39 (6.13 - 29.4)	5.17 (3.1 - 7.29)
Cyprus	Both	208.39 (130.24 - 272.23)	173.08 (139.86 - 222.2)	-16.94	25.32 (15.85 - 33.21)	11.33 (9.16 - 14.74)	-3.24 (-3.58 - -2.89)
Czech Republic	Both	5581.69 (4334.24 - 6185.97)	2402.07 (2002.46 - 3584.44)	-56.97	47.83 (37.46 - 53.1)	17.66 (14.31 - 27.63)	-3.75 (-3.9 - -3.6)
Democratic Republic of the Congo	Both	6905.42 (4782.28 - 9719.5)	11049.06 (8418.49 - 15156.52)	60.01	22.54 (16.42 - 31.17)	17.57 (13.45 - 24.57)	-0.93 (-1.01 - -0.85)
Denmark	Both	1341.1 (1043.07 - 1617.15)	952.63 (778.02 - 1238.22)	-28.97	21.9 (17.09 - 26.1)	12.73 (10.47 - 17.8)	-2.13 (-2.28 - -1.98)
Djibouti	Both	209.93 (118.7 - 294.12)	328.84 (218.76 - 495.16)	56.64	45.26 (29.13 - 63.03)	31.15 (20.93 - 46.05)	-1.67 (-1.93 - -1.4)
Dominica	Both	18.54 (14.7 - 25.97)	14.79 (12.46 - 19.58)	-20.23	26.03 (20.89 - 36.33)	19.94 (16.71 - 26.17)	-1.13 (-1.32 - -0.95)
Dominican Republic	Both	332.53 (228.8 - 624.78)	851.21 (379.57 - 1179.98)	155.98	5.27 (3.72 - 10.03)	8.32 (3.61 - 11.7)	0.75 (0 - 1.51)
Ecuador	Both	2753.64 (2281.75 - 3907.59)	2689.07 (2185 - 4079.58)	-2.34	29.45 (24.94 - 41.55)	16.51 (13.56 - 24.7)	-1.82 (-2.09 - -1.56)
Egypt	Both	38883.21 (27848 - 48900.23)	40606.36 (26778.34 - 50802.65)	4.43	76.81 (56.94 - 98.32)	45.16 (29.73 - 56.04)	-1.69 (-1.84 - -1.54)
El Salvador	Both	1625.3 (1302.95 - 2032.93)	963.66 (709.26 - 1544.35)	-40.71	36.18 (28.77 - 45.26)	16.15 (11.96 - 25.34)	-3.24 (-3.44 - -3.04)
Equatorial Guinea	Both	117.78 (77.56 - 176.01)	94.82 (60.28 - 144.07)	-19.49	34.17 (24.88 - 48.63)	9.3 (5.91 - 13.94)	-5.52 (-5.98 - -5.06)
Eritrea	Both	2018.98 (944.54 - 2892.55)	3209.51 (2097.8 - 4781.47)	58.97	80.26 (43.66 - 112.45)	58.97 (39.2 - 86.53)	-1.55 (-1.87 - -1.23)
Estonia	Both	588.1 (393.35 - 671.2)	232.24 (163.59 - 324.29)	-60.51	35.04 (23.66 - 39.97)	15.11 (10.86 - 22.74)	-3.57 (-3.95 - -3.19)
Ethiopia	Both	49469.66 (34095.25 - 64349.85)	46719.17 (29206.39 - 78303.53)	-5.56	108.07 (76.05 - 141.39)	50.89 (31.78 - 83.45)	-3.05 (-3.22 - -2.87)
Federated States of Micronesia	Both	13.69 (10.09 - 18.93)	11.17 (7.09 - 15.43)	-18.41	16.99 (12.63 - 23.01)	11.65 (7.38 - 16)	-1.42 (-1.5 - -1.33)
Fiji	Both	62.96 (46.82 - 76.92)	96.47 (50.58 - 131.62)	53.22	9.35 (7.15 - 11.43)	10.67 (5.67 - 14.44)	0.93 (0.65 - 1.2)
Finland	Both	1315.37 (999.98 - 1533.92)	827.53 (705.28 - 1070.56)	-37.09	22.75 (17.17 - 26.63)	12.16 (10.35 - 15.88)	-2.08 (-2.36 - -1.79)
France	Both	15746.16 (13080.04 - 20351.59)	9866.32 (8257.39 - 14042.2)	-37.34	24.24 (20 - 31.28)	12.5 (10.27 - 17.69)	-2.19 (-2.34 - -2.05)
Gabon	Both	164.63 (126.1 - 215.48)	210.28 (155.62 - 290.85)	27.73	20.15 (15.56 - 26.72)	14.04 (10.47 - 19.44)	-1.25 (-1.51 - -0.99)
Georgia	Both	3082.1 (2108.63 - 3604.09)	1504.5 (1259.16 - 1977.76)	-51.19	51.77 (35.62 - 60.76)	33.54 (28.09 - 45.6)	-2.15 (-2.43 - -1.87)
Germany	Both	27465.82 (18223.17 - 30734.6)	12321.58 (10153.16 - 18125.02)	-55.14	28.09 (18.96 - 31.45)	10.97 (8.7 - 17.19)	-3.6 (-4 - -3.2)
Ghana	Both	727.29 (558.19 - 1062.27)	1073.51 (816.28 - 1614.68)	47.60	5.72 (4.42 - 8.35)	4.23 (3.39 - 5.97)	-1.02 (-1.07 - -0.97)
Greece	Both	4649.56 (4064.26 - 6627.95)	4403.46 (3608.77 - 5163.71)	-5.29	36.67 (31.36 - 54.3)	29.98 (26.12 - 39.33)	-0.73 (-1.17 - -0.29)
Greenland	Both	13.23 (7.27 - 16.88)	5.34 (4.09 - 6.49)	-59.64	23.98 (12.14 - 30.72)	8.09 (6.41 - 10.09)	-4.38 (-4.52 - -4.24)
Grenada	Both	15.32 (12.54 - 20.38)	11.22 (9.15 - 16.44)	-26.76	20.23 (16.57 - 26.36)	9.32 (7.59 - 13.54)	-2.56 (-2.79 - -2.34)
Guam	Both	9.32 (7.1 - 13.68)	12.52 (9.99 - 16.27)	34.33	7.37 (5.66 - 11.06)	7.24 (5.73 - 9.38)	0.54 (0.09 - 0.99)
Guatemala	Both	2055.28 (1572.83 - 2996.65)	1924.15 (1563.61 - 2816.85)	-6.38	27.47 (22.98 - 39.1)	12.89 (10.65 - 17.91)	-2.77 (-3.08 - -2.45)
Guinea	Both	2175.58 (1330.77 - 2897.79)	2687.8 (1739.4 - 3540.76)	23.54	34.67 (23.44 - 42.85)	25.44 (17.61 - 31.79)	-1.08 (-1.21 - -0.94)
Guinea-Bissau	Both	269.23 (176.49 - 358.26)	299.48 (227.35 - 410.64)	11.24	30.7 (21.49 - 39.68)	19.73 (15.56 - 25.51)	-1.64 (-1.69 - -1.59)
Guyana	Both	55.73 (29.6 - 144.73)	110.2 (80.59 - 132.92)	97.74	8.51 (4.69 - 21.77)	14.92 (10.96 - 17.95)	2.16 (0.98 - 3.35)
Haiti	Both	2586.52 (2014.97 - 3639.1)	3411.99 (2226.13 - 5009.74)	31.91	49.22 (37.23 - 73.21)	33.64 (21.61 - 50.34)	-1.36 (-1.43 - -1.29)
Honduras	Both	307.4 (210.53 - 445.39)	351.29 (244.96 - 456.67)	14.28	8.31 (5.32 - 10.51)	4.73 (2.91 - 6.4)	-2.05 (-2.12 - -1.98)
Hungary	Both	4586.61 (3399.73 - 5100.33)	1540.12 (1309.46 - 2220.37)	-66.42	38.35 (28.13 - 42.85)	12.22 (10.32 - 17.48)	-4.3 (-4.43 - -4.17)
Iceland	Both	46.11 (38.28 - 57.08)	34.06 (28.4 - 51.78)	-26.13	17.19 (14.29 - 21.29)	8.64 (7.1 - 13.23)	-2.23 (-2.36 - -2.1)
India	Both	299265.42 (188045.44 - 375199.04)	214721.83 (176112.23 - 273747.14)	-28.25	35.75 (23.3 - 43.8)	15.81 (12.98 - 20.07)	-3.08 (-3.23 - -2.92)
Indonesia	Both	41299.97 (29213.38 - 51013.13)	35402.54 (27284.04 - 45565.87)	-14.28	24.64 (17.88 - 30.81)	13.85 (10.63 - 17.83)	-2.03 (-2.14 - -1.93)
Iran	Both	8275.21 (6290.86 - 13954.86)	11477.3 (7895.63 - 12943.84)	38.69	16.57 (12.79 - 27.51)	13.81 (9.45 - 15.58)	0.71 (0.11 - 1.31)
Iraq	Both	4237.58 (2072.73 - 5925.69)	3311.12 (2676.78 - 4818.67)	-21.86	29.03 (14.77 - 39.71)	8.31 (6.73 - 12.25)	-4.89 (-5.94 - -3.82)
Ireland	Both	943.86 (740.34 - 1068.38)	723.44 (568.72 - 917.9)	-23.35	25.3 (19.82 - 28.72)	13.1 (10.37 - 16.43)	-2.3 (-2.42 - -2.17)

Israel	Both	1183.12 (1020.57 - 1447.53)	1335.19 (1103.33 - 1806.27)	12.85	24.43 (21.05 - 29.8)	14.51 (11.91 - 19.49)	-2.36 (-2.62 - -2.09)
Italy	Both	21340.48 (16892.72 - 24260.47)	12393.34 (10097.14 - 16373.82)	-41.93	31.65 (24.8 - 35.26)	16.19 (13.06 - 21.15)	-2.11 (-2.36 - -1.86)
Jamaica	Both	250.01 (122.91 - 309.37)	286.21 (180.27 - 382.38)	14.48	11.58 (5.92 - 14.18)	9.68 (6.13 - 12.86)	-0.46 (-0.98 - 0.07)
Japan	Both	5534.98 (4720.13 - 7124.12)	5866.23 (4190.54 - 6677.63)	5.98	3.7 (3.16 - 4.7)	3.18 (2.35 - 3.66)	-0.09 (-0.26 - 0.07)
Jordan	Both	284.5 (184.16 - 361.92)	347.92 (263.08 - 428.08)	22.29	9.47 (5.56 - 12.2)	3.68 (2.71 - 4.49)	-3.88 (-4.12 - -3.65)
Kazakhstan	Both	4235.75 (3274.12 - 5631.97)	3202.71 (2681.42 - 3911.04)	-24.39	25.83 (19.93 - 34.31)	17.18 (14.38 - 21.02)	-1.82 (-2.4 - -1.23)
Kenya	Both	4038.31 (3120.84 - 5410.46)	8206.1 (6083.74 - 9636.07)	103.21	20.25 (16.36 - 27.02)	18.91 (13.87 - 22.18)	-0.07 (-0.32 - 0.18)
Kiribati	Both	5.44 (4.4 - 7.63)	8.98 (6.73 - 11.3)	65.07	8.52 (6.97 - 11.97)	8.33 (6.44 - 10.64)	-0.22 (-0.42 - -0.02)
Kuwait	Both	201.81 (137.7 - 235.77)	205.9 (171.81 - 261.31)	2.03	12.53 (8.69 - 14.37)	4.92 (4.16 - 6.24)	-2.71 (-3.26 - -2.15)
Kyrgyzstan	Both	864.84 (526.44 - 1043.09)	534.9 (448.15 - 735.8)	-38.15	21.45 (13.16 - 25.52)	9.05 (7.61 - 12.3)	-2.72 (-3.05 - -2.38)
Laos	Both	1645.25 (1057.38 - 2059.4)	1289.11 (907.07 - 1760.57)	-21.65	45.49 (30.53 - 57.1)	20.49 (14.56 - 28.4)	-3.05 (-3.2 - -2.9)
Latvia	Both	884.65 (588.89 - 1031.06)	443 (262.43 - 552.19)	-49.92	30.34 (20.38 - 35.47)	19.44 (11.23 - 24.8)	-1.9 (-2.12 - -1.67)
Lebanon	Both	3085.23 (2271.56 - 3917.22)	3776.71 (2683.64 - 4657.47)	22.41	84.69 (64.19 - 107.59)	44.27 (31.41 - 53.81)	-2.48 (-2.76 - -2.2)
Lesotho	Both	276.58 (220.14 - 375.14)	382.37 (280.75 - 510.41)	38.25	19.64 (15.73 - 26.8)	22.46 (16.55 - 29.77)	1.21 (0.69 - 1.73)
Liberia	Both	400.96 (278.66 - 509.33)	455.29 (355.35 - 609.44)	13.55	20.8 (15.31 - 25.49)	11.87 (9.14 - 15.34)	-2.4 (-2.76 - -2.04)
Libya	Both	2568.24 (1857.66 - 3303.57)	3952.35 (2751.79 - 5040.26)	53.89	71.57 (53.67 - 92.31)	56.9 (40.46 - 71.38)	-0.83 (-1.08 - -0.58)
Lithuania	Both	1304.58 (949.64 - 1503)	565.72 (487.88 - 724.25)	-56.64	32.71 (23.81 - 37.65)	16.81 (14.3 - 22)	-3 (-3.38 - -2.61)
Luxembourg	Both	96.31 (74.28 - 122.03)	75.11 (57.26 - 123.86)	-22.01	21.04 (16.08 - 27.28)	10.46 (7.89 - 17.72)	-2.84 (-3.04 - -2.64)
Macedonia	Both	631.33 (372.7 - 796.77)	517.67 (326.11 - 636.88)	-18.00	30.28 (18.08 - 38.07)	19.83 (12.54 - 24.52)	-2.26 (-2.62 - -1.89)
Madagascar	Both	6974.3 (5373.46 - 8847.47)	7682.69 (5558.37 - 9942.79)	10.16	57.71 (45.43 - 71.75)	32.86 (24.02 - 41.87)	-2.31 (-2.63 - -1.98)
Malawi	Both	2472.44 (755.56 - 3768.71)	3165.72 (2349.31 - 4199.72)	28.04	26.71 (9.72 - 38.46)	19.79 (15.13 - 25.09)	-1.71 (-2.06 - -1.35)
Malaysia	Both	3153.05 (1762.95 - 4242.03)	3257.74 (1661.73 - 4474.39)	3.32	20.55 (12.07 - 27.21)	10.44 (5.33 - 14.22)	-2.6 (-2.89 - -2.3)
Maldives	Both	18.61 (13.38 - 24.4)	12.69 (9.66 - 17.62)	-31.81	11.37 (8.38 - 14.47)	2.95 (2.36 - 3.89)	-5.15 (-5.35 - -4.95)
Mali	Both	4078.12 (2410.48 - 5355.7)	4638.54 (3112.74 - 6247.12)	13.74	46.46 (31.21 - 57.08)	23.4 (17.45 - 29.52)	-2.6 (-2.83 - -2.36)
Malta	Both	116.72 (102.61 - 151.13)	104.48 (89.48 - 143.67)	-10.49	29.05 (25.39 - 37.77)	18.68 (15.74 - 25.86)	-1.6 (-1.73 - -1.47)
Marshall Islands	Both	5.35 (4 - 6.48)	6.93 (4.38 - 8.79)	29.53	16.28 (12.26 - 19.7)	13.55 (8.68 - 16.98)	-0.56 (-0.75 - -0.37)
Mauritania	Both	318.01 (234.52 - 402.16)	329.51 (246.17 - 474.6)	3.62	18.03 (13.7 - 22.06)	10.03 (7.57 - 14.05)	-2.23 (-2.31 - -2.14)
Mauritius	Both	145.5 (103.47 - 165.52)	148.25 (90.69 - 175.37)	1.89	13.76 (10.03 - 15.54)	10.19 (6.1 - 12.15)	-1.57 (-1.99 - -1.15)
Mexico	Both	22872.26 (17911.12 - 25811.37)	20333.61 (17411.48 - 26434.58)	-11.10	31.21 (24.61 - 35.7)	16.36 (13.99 - 21.21)	-2.35 (-2.64 - -2.05)
Moldova	Both	2340.43 (1682.95 - 3011.52)	1164.99 (976.77 - 1585.48)	-50.22	51.71 (36.99 - 65.8)	27.38 (21.93 - 37.58)	-2.8 (-3.07 - -2.53)
Mongolia	Both	145.87 (109.29 - 225.04)	187.31 (154.18 - 241.91)	28.41	9.68 (7.91 - 13.18)	5.98 (4.85 - 7.47)	-2.42 (-2.82 - -2.02)
Montenegro	Both	314.07 (261.8 - 407.57)	225.32 (186.5 - 309.68)	-28.26	48.13 (40.18 - 62.48)	29.93 (24.74 - 41.06)	-2.24 (-2.58 - -1.89)
Morocco	Both	17680.13 (11485.67 - 22406.2)	16113.76 (10935.44 - 20434.11)	-8.86	72.55 (49.94 - 87.89)	44.68 (30.49 - 56.5)	-1.76 (-1.82 - -1.7)
Mozambique	Both	9527.43 (7216.91 - 12334.84)	11907.23 (8571.47 - 15149.26)	24.98	68.64 (54.42 - 89.35)	43.69 (30.88 - 56.36)	-1.63 (-1.89 - -1.38)
Myanmar	Both	17865.79 (11843.94 - 22379.68)	10921.45 (8363.83 - 14083.42)	-38.87	49.01 (33.55 - 61.38)	20.76 (15.92 - 26.53)	-3.29 (-3.55 - -3.03)
Namibia	Both	402.77 (267.67 - 508.15)	421.95 (262.3 - 571.01)	4.76	34.92 (25.05 - 42.22)	19.85 (13.37 - 25.67)	-2.33 (-2.87 - -1.8)
Nepal	Both	7730.65 (5123.25 - 11554.27)	5005.03 (2981.11 - 9214.02)	-35.26	44.88 (31.49 - 67.27)	17.49 (10.5 - 32.11)	-3.52 (-3.82 - -3.21)
Netherlands	Both	3908.94 (3024.06 - 4395.08)	2679.83 (2260.54 - 3430.8)	-31.44	22.55 (17.48 - 25.4)	12.28 (10.54 - 16.48)	-2.2 (-2.34 - -2.06)
New Zealand	Both	590.97 (404.77 - 670.02)	521.87 (333.59 - 615.03)	-11.69	15.88 (10.99 - 17.99)	10.23 (6.66 - 12.12)	-1.5 (-1.69 - -1.32)
Nicaragua	Both	498.26 (330.61 - 601.58)	478.49 (306.69 - 591.32)	-3.97	14.94 (10.66 - 17.74)	8.3 (5.28 - 10.22)	-2.2 (-2.32 - -2.08)
Niger	Both	2078.9 (1248.85 - 2970.33)	2318.19 (1762.6 - 3106.44)	11.51	26.27 (18.77 - 33.35)	13.88 (10.75 - 18.02)	-2.78 (-2.99 - -2.57)
Nigeria	Both	99151.8 (58088.59 - 146692.94)	141039.33 (91984.07 - 199040.19)	42.25	111.3 (71.08 - 158.34)	71.74 (49.32 - 102.22)	-2.02 (-2.19 - -1.85)
North Korea	Both	2229.34 (1433.19 - 2962.89)	2853.92 (1924.07 - 3542.54)	28.02	11.09 (7.18 - 14.69)	9.97 (6.72 - 12.32)	-0.31 (-0.55 - -0.07)
Northern Mariana Islands	Both	1.8 (1.34 - 2.74)	1.85 (1.39 - 2.26)	2.78	4.54 (3.47 - 6.57)	3.68 (2.83 - 4.52)	-0.36 (-0.66 - -0.06)
Norway	Both	887.41 (793.51 - 1184.76)	608.85 (524.53 - 901.89)	-31.39	17.94 (16.04 - 24.08)	9.52 (8.11 - 14.06)	-2.21 (-2.32 - -2.1)
Oman	Both	584.87 (340.39 - 798.47)	704.65 (423.97 - 938.28)	20.48	37 (22.23 - 49.57)	16.27 (9.61 - 21.14)	-3 (-3.45 - -2.54)
Pakistan	Both	89899.78 (65438.11 - 116689.65)	148991.24 (111698.32 - 195634.37)	65.73	85.39 (63.12 - 111.43)	69.12 (52.46 - 90.88)	-0.98 (-1.19 - -0.76)
Palestine	Both	297.12 (160.95 - 605.13)	619.43 (482.11 - 1084.15)	108.48	18.84 (10.9 - 36.31)	15.23 (12.24 - 25.14)	-0.77 (-1.06 - -0.47)
Panama	Both	224.04 (185.26 - 272.46)	370.86 (234.95 - 440.91)	65.53	10.3 (8.61 - 12.63)	9.44 (5.99 - 11.24)	0.21 (-0.17 - 0.6)
Papua New Guinea	Both	848.84 (639.72 - 1075.03)	1578.68 (1093.52 - 2005.11)	85.98	24.76 (19.22 - 31.78)	19.4 (13.61 - 24.68)	-0.62 (-0.76 - -0.48)
Paraguay	Both	641.83 (474.69 - 893.83)	917.29 (672.36 - 1165.03)	42.92	18.3 (13.67 - 25.49)	14.06 (10.17 - 17.82)	-0.78 (-0.91 - -0.64)
Peru	Both	3097.5 (2572.65 - 4308.34)	2290.18 (1693.89 - 2880.69)	-26.06	15.81 (13.07 - 21.64)	7.15 (5.25 - 8.93)	-2.85 (-2.96 - -2.74)
Philippines	Both	7507.41 (6258.27 - 11154.08)	6931.31 (4627.24 - 14343.09)	-7.67	13.29 (11.24 - 19.1)	7.2 (4.89 - 14.56)	-2.28 (-2.46 - -2.09)
Poland	Both	19415.06 (14032.57 - 21215.39)	8525.75 (7277.61 - 10867.16)	-56.09	46.26 (33.42 - 50.69)	17.6 (15.17 - 22.54)	-3.86 (-4.01 - -3.71)
Portugal	Both	2866.1 (2467.55 - 4176.61)	1658.42 (1394.08 - 2429.44)	-42.14	25.53 (22 - 36.52)	11.77 (9.75 - 17.21)	-3.16 (-3.31 - -3)
Puerto Rico	Both	918.43 (671.19 - 1060.02)	677.75 (470.37 - 791.79)	-26.21	25 (18.34 - 28.8)	15.6 (10.95 - 18.34)	-2.14 (-2.46 - -1.83)
Qatar	Both	27.16 (18.82 - 47.56)	135.55 (78.23 - 229.49)	399.08	8.08 (5.99 - 13.03)	5.3 (3.19 - 8.26)	-1.5 (-1.75 - -1.26)
Romania	Both	8966.33 (5808.35 - 10114.24)	3976.69 (3019.19 - 4582.52)	-55.65	36.24 (23.4 - 41.05)	16.98 (12.65 - 19.47)	-3.19 (-3.39 - -2.99)
Russian Federation	Both	61495.91 (38815.73 - 72815.55)	34625.28 (30102.56 - 42618.71)	-43.69	38.56 (24.04 - 45.79)	20.8 (18.27 - 25.94)	-2.77 (-3.23 - -2.3)
Rwanda	Both	3959.54 (2645.54 - 5052.35)	3171.37 (2320.35 - 4336.11)	-19.91	58.38 (40.43 - 73.61)	27.22 (20.31 - 36.4)	-3.63 (-4.05 - -3.21)
Saint Lucia	Both	28.44 (23.83 - 40.77)	34.31 (26.44 - 44.15)	20.64	24.46 (20.6 - 35.4)	17.45 (13.49 - 22.36)	-1.23 (-1.46 - -1.01)
Saint Vincent and the Grenadines	Both	11.39 (5.72 - 31.08)	26.52 (21.26 - 32.02)	132.84	12.08 (6.16 - 32.75)	21.7 (17.29 - 26.26)	1.1 (0.25 - 1.97)
Samoa	Both	31.53 (20.89 - 39.5)	25.31 (16.32 - 32.18)	-19.73	22.53 (15.71 - 27.46)	14.37 (9.56 - 18.03)	-1.77 (-1.92 - -1.63)
Sao Tome and Principe	Both	3.9 (2.59 - 7.18)	4.64 (3.06 - 8.17)	18.97	3.44 (2.47 - 6.1)	2.67 (1.8 - 4.34)	-0.92 (-1 - -0.83)
Saudi Arabia	Both	3947.63 (1259.56 - 6163.99)	6390.29 (1699.67 - 10070.18)	61.88	28.66 (9.99 - 44.79)	18.56 (5.05 - 28.75)	-1.01 (-1.37 - -0.64)
Senegal	Both	1376.57 (984.87 - 1690.49)	1762.54 (1346.16 - 2530.06)	28.04	20.06 (15.14 - 24.08)	14.49 (11.18 - 19.67)	-1.24 (-1.29 - -1.18)
Serbia	Both	4099.26 (2731.56 - 5167.51)	2452.96 (1528.9 - 3023.42)	-40.16	39.51 (26.09 - 50.04)	22.19 (13.62 - 28.09)	-2.09 (-2.3 - -1.87)
Seychelles	Both	14.24 (10.46 - 23.65)	16.17 (12.49 - 20.61)	13.55	21.8 (16.32 - 34.94)	14.66 (11.48 - 18.8)	-1.69 (-1.84 - -1.54)
Sierra Leone	Both	784.2 (514.54 - 1053.17)	924.49 (698.66 - 1178.87)	17.89	20.96 (14.79 - 26.73)	13.79 (10.27 - 17.14)	-1.57 (-1.63 - -1.5)
Singapore	Both	225.21 (178.59 - 270.94)	258.29 (180.64 - 311.88)	14.69	7.09 (5.75 - 8.88)	3.92 (2.74 - 4.72)	-1.49 (-1.8 - -1.18)
Slovakia	Both	1869.69 (1457.54 - 2207.52)	1259.17 (1048.62 - 1559.99)	-32.65	33.25 (26.08 - 39.17)	18.88 (15.81 - 23.64)	-1.88 (-2 - -1.77)
Slovenia	Both	609.99 (477.04 - 698.94)	334.01 (265.08 - 419.1)	-45.24	27.24 (21.39 - 31.34)	12.61 (10.4 - 16.5)	-3.14 (-3.27 - -3.02)
Solomon Islands	Both	46.05 (33.88 - 60.7)	76.18 (52.82 - 105.87)	65.43	18.2 (13.61 - 24.28)	14.28 (9.96 - 19.68)	-0.62 (-0.76 - -0.47)
Somalia	Both	3562.95 (995 - 5900.23)	8364.99 (4844.12 - 13577.48)	134.78	54.97 (20.85 - 88.44)	55.33 (33.57 - 86.5)	-0.4 (-0.69 - -0.11)
South Africa	Both	4059.21 (3173.47 - 4883.5)	4745.95 (3499.88 - 5761.35)	16.92	12.71 (9.89 - 15.19)	8.78 (6.47 - 10.6)	-1.53 (-2.31 - -0.73)
South Korea	Both	1624.28 (1279.71 - 2961.82)	1953.88 (1163.56 - 2445.11)	20.29	3.89 (3.09 - 6.96)	2.93 (1.81 - 3.63)	-0.42 (-0.62 - -0.22)
South Sudan	Both	2159.68 (710.13 - 3716.09)	3545.81 (2458.59 - 4835.82)	64.18	41.1 (16.96 - 67.67)	40.13 (28.02 - 53.63)	-0.35 (-0.62 - -0.08)
Spain	Both	12255.11 (9333.71 - 13698.11)	7143.05 (5650.23 - 9544.54)	-41.71	27.43 (20.52 - 30.55)	12.09 (9.51 - 16.18)	-2.95 (-3.06 - -2.85)
Sri Lanka	Both	4137.73 (3518.52 - 5608.99)	2818.82 (2219.65 - 3771.63)	-31.88	27.69 (23.35 - 36.51)	11.66 (9.18 - 15.74)	-3.59 (-3.94 - -3.24)
Sudan	Both	6234.79 (3910.98 - 9708.95)	7431.99 (5698.16 - 10079.14)	19.20	36.67 (24.7 - 56.04)	21.8 (16.27 - 29.9)	-1.82 (-1.89 - -1.76)
Suriname	Both	86.94 (72.86 - 123.73)	123.53 (98.17 - 152.18)	42.09	23.89 (20.13 - 34.08)	20.82 (16.62 - 25.69)	-0.42 (-0.75 - -0.08)
Swaziland	Both	110.36 (86.63 - 147.71)	171.82 (127.03 - 222.05)	55.69	19.87 (15.86 - 26.92)	18.55 (13.74 - 24.1)	0.56 (-0.17 - 1.3)
Sweden	Both	1397.08 (1188.55 - 1723.61)	1027.55 (869.75 - 1321.95)	-26.45	13.28 (11.49 - 16.93)	8.19 (7.04 - 10.69)	-1.61 (-1.76 - -1.46)
Switzerland	Both	1346.18 (1025.93 - 1591.25)	943.75 (744.67 - 1377.2)	-29.89	16.84 (12.74 - 19.62)	8.59 (6.88 - 13.11)	-2.39 (-2.5 - -2.28)
Syria	Both	405.23 (310.2 - 539.42)	464.81 (278.34 - 614.39)	14.70	4.44 (3.04 - 5.4)	2.91 (1.64 - 3.91)	-1.69 (-1.94 - -1.44)
Tajikistan	Both	369.5 (299.03 - 509.3)	595.79 (491.07 - 804.26)	61.24	9.24 (7.31 - 11.69)	7.73 (6.14 - 9.39)	-0.94 (-1.1 - -0.79)
Tanzania	Both	9353.57 (3995.51 - 13016.16)	15415.86 (10984.38 - 21676.13)	64.81	39.02 (19.65 - 53.57)	30.33 (21.85 - 41.18)	-1.19 (-1.36 - -1.02)
Thailand	Both	9328.78 (7309.94 - 13681.84)	5939.65 (4422.01 - 9695.73)	-36.33	17.52 (13.99 - 25.34)	7.02 (5.07 - 11.9)	-4.08 (-4.39 - -3.77)

The Bahamas	Both	45.73 (37.78 - 54.75)	54.84 (44.07 - 71.04)	19.92	18.88 (15.6 - 22.75)	13.6 (10.95 - 17.52)	-1.51 (-1.71 - -1.3)
The Gambia	Both	190.3 (131.22 - 256.15)	310.9 (222.27 - 448.88)	63.37	22.1 (15.99 - 29.02)	16.79 (12.58 - 23.53)	-0.78 (-0.92 - -0.64)
Timor-Leste	Both	221.02 (154.26 - 297.26)	195.44 (103.83 - 304.77)	-11.57	33.4 (22.51 - 48.34)	17.73 (9.52 - 28.02)	-2.55 (-2.69 - -2.41)
Togo	Both	634.37 (469.21 - 788.75)	811.13 (624.43 - 1050.43)	27.86	19.71 (15.22 - 24.21)	12.97 (9.81 - 16.58)	-1.71 (-1.88 - -1.54)
Tonga	Both	5.91 (4.42 - 8.02)	6.7 (4.59 - 9.14)	13.37	7.58 (5.67 - 10.27)	7.15 (4.9 - 9.77)	-0.07 (-0.17 - 0.03)
Trinidad and Tobago	Both	123.61 (99.63 - 154.57)	186.9 (100.77 - 252.95)	51.20	11.14 (9.07 - 13.98)	12.06 (6.41 - 16.29)	0.97 (0.53 - 1.42)
Tunisia	Both	3076.92 (2366.11 - 3894.66)	2796.19 (1971.83 - 3584.28)	-9.12	39.06 (30.87 - 50.53)	23.28 (16.43 - 29.76)	-1.85 (-2.03 - -1.67)
Turkey	Both	15529.18 (11233.9 - 26152.74)	9679.99 (7664.95 - 13800.22)	-37.67	28.93 (20.94 - 48.57)	11.43 (9.07 - 16.23)	-3.69 (-3.94 - -3.44)
Turkmenistan	Both	890.71 (598.73 - 1056.57)	1047.91 (671.27 - 1259.75)	17.65	26.07 (18.39 - 30.43)	20.56 (13.49 - 24.4)	-0.57 (-1 - -0.13)
Uganda	Both	6666.38 (4820.96 - 8801.53)	12337.35 (8628.49 - 17072.85)	85.07	40.92 (30.87 - 53.9)	35.29 (25.51 - 47.11)	-0.79 (-0.96 - -0.61)
Ukraine	Both	19095.48 (13745.45 - 24289.73)	20700.19 (16295.77 - 30438)	8.40	33.88 (24.19 - 42.85)	43.06 (32.94 - 65.22)	0.04 (-0.52 - 0.61)
United Arab Emirates	Both	390.07 (264.23 - 636.45)	1598.93 (1132.05 - 2345.54)	309.91	24.4 (17.84 - 37.56)	17.25 (12.06 - 25.8)	-1.17 (-1.27 - -1.07)
United Kingdom	Both	17537.55 (13237.04 - 19402.5)	11544.4 (9493.33 - 13741.65)	-34.17	26.34 (19.45 - 28.77)	14.2 (11.91 - 17.1)	-2.25 (-2.44 - -2.07)
United States	Both	64039.33 (46471.95 - 70172.27)	45881.61 (38942.39 - 66692.89)	-28.35	22.74 (16.5 - 24.94)	12.18 (10.08 - 18.21)	-2.49 (-2.62 - -2.37)
Uruguay	Both	1027.1 (748.11 - 1161.18)	733.66 (537.52 - 873.43)	-28.57	30.34 (22.11 - 34.31)	18.72 (13.66 - 22.4)	-1.6 (-1.8 - -1.4)
Uzbekistan	Both	4539.2 (3650.41 - 5947.4)	5849.75 (4896.66 - 7473.56)	28.87	23.82 (18.83 - 34.09)	18.56 (15.55 - 23.83)	-0.92 (-1.09 - -0.75)
Vanuatu	Both	29.22 (15.17 - 51.56)	59 (26.75 - 108.99)	101.92	24.82 (12.73 - 44.21)	24.03 (10.85 - 44.68)	-0.03 (-0.18 - 0.11)
Venezuela	Both	3874.7 (3021.7 - 4762.59)	5777.17 (4087.16 - 7011.1)	49.10	24.7 (18.9 - 29.75)	18.46 (13.2 - 22.35)	-0.91 (-1.04 - -0.77)
Vietnam	Both	13297.22 (9689.59 - 16646.09)	13289.83 (8116.8 - 16673.21)	-0.06	22.47 (16.83 - 28.19)	13.25 (8.12 - 16.6)	-1.81 (-1.92 - -1.7)
Virgin Islands, U.S.	Both	7.38 (5.7 - 12.63)	10.92 (8.1 - 13.43)	47.97	7.26 (5.63 - 12.32)	7.86 (5.76 - 9.48)	0.81 (0.37 - 1.25)
Yemen	Both	4039.21 (1587.2 - 6615.13)	6387.91 (4675.23 - 9078.54)	58.15	39.14 (18.2 - 62.52)	25.92 (18.82 - 37.16)	-1.68 (-1.79 - -1.56)
Zambia	Both	4035.78 (2248.59 - 5278.26)	5792.05 (4118.23 - 7968.59)	43.52	55.45 (35.29 - 70.67)	36.81 (26.68 - 49.11)	-1.97 (-2.3 - -1.64)
Zimbabwe	Both	1274.6 (789.65 - 1643.64)	3168.91 (1647.32 - 4349.3)	148.62	15.52 (11.02 - 18.99)	24.66 (14.3 - 32.31)	2.88 (1.76 - 4)
Afghanistan	Female	2237.99 (737.61 - 3911.37)	6685.72 (4232.99 - 9906.05)	198.74	54.76 (18.72 - 95.05)	49.82 (31.93 - 72.96)	-0.35 (-0.63 - -0.06)
Albania	Female	368.88 (293.89 - 519.32)	208.67 (140.5 - 330.39)	-43.43	24.05 (19.17 - 34.25)	13.55 (9.07 - 21.29)	-1.91 (-2.05 - -1.78)
Algeria	Female	5753.29 (3434.9 - 7719.66)	6134.4 (4078.57 - 7902.48)	6.62	49.13 (31.82 - 62.65)	30.22 (20.19 - 38.66)	-1.7 (-1.8 - -1.61)
American Samoa	Female	1.16 (0.81 - 1.8)	1.25 (0.93 - 1.98)	7.76	5.81 (4.05 - 9.38)	4.93 (3.68 - 7.75)	-0.45 (-0.72 - -0.17)
Andorra	Female	3.75 (2.66 - 5.1)	4.14 (3.07 - 6.02)	10.40	12.57 (8.95 - 16.91)	9.43 (7.03 - 13.64)	-1.04 (-1.19 - -0.88)
Angola	Female	798.2 (425.1 - 1391.38)	1256.4 (905.23 - 1866.24)	57.40	19.02 (11.39 - 30.81)	11.01 (7.68 - 16.08)	-2.12 (-2.4 - -1.84)
Antigua and Barbuda	Female	0.64 (0.52 - 0.96)	0.93 (0.68 - 1.1)	45.31	2.06 (1.69 - 3.07)	1.91 (1.44 - 2.25)	-0.06 (-0.28 - 0.17)
Argentina	Female	3118.97 (2609.88 - 4420.46)	2562.61 (1966.56 - 4357.47)	-17.84	18.33 (15.32 - 26.22)	10.35 (7.91 - 17.77)	-2.27 (-2.39 - -2.14)
Armenia	Female	59.63 (40.96 - 127.22)	87.47 (55.66 - 104.51)	46.69	3.42 (2.41 - 7.29)	4.94 (3.05 - 5.98)	1.77 (1 - 2.53)
Australia	Female	1168.07 (910.06 - 1352.85)	1091.19 (870.94 - 1364.98)	-6.58	12.44 (9.69 - 14.48)	7.89 (6.26 - 9.89)	-1.52 (-1.77 - -1.27)
Austria	Female	1154.7 (579.3 - 1355.56)	348.29 (275.61 - 621.42)	-69.84	22.41 (12.17 - 26.33)	6.21 (4.71 - 11.9)	-5.38 (-5.92 - -4.84)
Azerbaijan	Female	890.48 (419.31 - 1317.66)	899 (473.06 - 1313.6)	0.96	23.36 (11.38 - 33.54)	15.84 (8.47 - 22.89)	-1.8 (-2.19 - -1.4)
Bahrain	Female	28.47 (11.1 - 43.47)	23.29 (15.17 - 29.62)	-18.19	16.34 (6.37 - 24.84)	4.28 (2.85 - 5.36)	-5.54 (-6.01 - -5.08)
Bangladesh	Female	16893.07 (11948.95 - 23735.38)	9559.24 (6363.75 - 15909.62)	-43.41	33.45 (24.63 - 46.06)	11.46 (7.69 - 19.04)	-3.91 (-4.03 - -3.79)
Barbados	Female	18.95 (14.95 - 25.3)	16.43 (13.07 - 21.33)	-13.30	13.16 (10.41 - 17.47)	9.63 (7.61 - 12.52)	-1.18 (-1.28 - -1.09)
Belarus	Female	1528.41 (963.93 - 1821.65)	965.16 (651.82 - 1188.5)	-36.85	26.04 (16.53 - 31.47)	17.42 (11.36 - 22.08)	-1.31 (-1.55 - -1.07)
Belgium	Female	963.06 (654.68 - 1100.55)	536.12 (445.97 - 697.5)	-44.33	15.89 (11.21 - 18.39)	7.77 (6.42 - 10.44)	-2.42 (-2.54 - -2.3)
Belize	Female	10.79 (8.51 - 14.47)	25.05 (20.56 - 33.97)	132.16	14.18 (11.5 - 18.87)	13.01 (10.85 - 17.78)	-0.51 (-0.8 - -0.21)
Benin	Female	257.04 (179.54 - 344.16)	424.7 (291.95 - 627.69)	65.23	11.93 (8.69 - 15.84)	8.57 (5.92 - 12.44)	-1.49 (-1.67 - -1.3)
Bermuda	Female	3.83 (2.32 - 10.67)	6.04 (3.79 - 7.51)	57.70	11.65 (7.06 - 31.67)	14.46 (8.63 - 18.68)	-0.46 (-1.37 - 0.45)
Bhutan	Female	75.43 (51.89 - 105.14)	48.25 (25.1 - 94.93)	-36.03	29.63 (20.85 - 40.85)	10.34 (5.68 - 19.81)	-4.18 (-4.36 - -4.01)
Bolivia	Female	844.04 (442.3 - 1252.98)	730.75 (472.07 - 1013.08)	-13.42	29.47 (16.32 - 40.92)	13.64 (8.86 - 18.83)	-2.97 (-3.1 - -2.84)
Bosnia and Herzegovina	Female	411.21 (289.43 - 502.65)	291.08 (157.32 - 384.58)	-29.21	17.08 (12 - 20.96)	13.45 (7.38 - 17.88)	-1.37 (-1.69 - -1.06)
Botswana	Female	47.42 (26.33 - 83.59)	70.36 (51.48 - 102.07)	48.38	8.63 (5.05 - 14.81)	6.22 (4.6 - 9.09)	-0.32 (-0.84 - 0.21)
Brazil	Female	9560.7 (8360.46 - 12119.15)	10193.82 (8205.96 - 11832.01)	6.62	13.72 (11.8 - 16.8)	8.74 (7.07 - 10.11)	-1.66 (-1.73 - -1.59)
Brunei	Female	10.79 (5.63 - 23.06)	38.86 (25.12 - 50.22)	260.15	11.19 (5.74 - 24.21)	18.19 (11.7 - 23.3)	3.22 (2.66 - 3.78)
Bulgaria	Female	1093.93 (914.43 - 1318.07)	682.71 (566.55 - 858.28)	-37.59	22.31 (18.19 - 26.11)	16.86 (13.61 - 20.4)	-1.52 (-1.78 - -1.26)
Burkina Faso	Female	664.41 (449.03 - 881.17)	818.49 (581.83 - 1142.74)	23.19	15.81 (11.13 - 20.25)	9.02 (6.54 - 12.06)	-2.46 (-2.71 - -2.2)
Burundi	Female	1003.49 (582.63 - 1403.78)	1040.49 (678.23 - 1419.14)	3.69	39.03 (22.85 - 54.04)	21.17 (13.84 - 28.44)	-2.63 (-2.81 - -2.44)
Cambodia	Female	1078.26 (649.68 - 1432.53)	954.7 (658.5 - 1308.5)	-11.46	24.07 (15.01 - 31.14)	11.93 (8.25 - 16.41)	-2.85 (-3 - -2.7)
Cameroon	Female	558.48 (383.57 - 743.91)	1005.08 (705.6 - 1424.99)	79.97	12.33 (8.78 - 16.14)	8.49 (5.86 - 11.65)	-1.65 (-1.87 - -1.43)
Canada	Female	2368.44 (1565.3 - 2716.45)	1894.61 (1557.63 - 2410.51)	-20.01	15.39 (10.04 - 17.75)	9.25 (7.51 - 11.66)	-1.91 (-2.13 - -1.68)
Cape Verde	Female	4.19 (3.25 - 5.91)	5.52 (4.19 - 6.92)	31.74	2.56 (1.97 - 3.48)	2.06 (1.54 - 2.54)	-0.98 (-1.12 - -0.84)
Central African Republic	Female	226.96 (134.82 - 351.84)	352.01 (231.13 - 530.38)	55.10	20.31 (13.27 - 29.97)	18.11 (12.07 - 26.42)	-0.54 (-0.65 - -0.42)
Chad	Female	305.86 (199.83 - 425.78)	567.17 (398 - 789.28)	85.43	11.52 (7.9 - 15.71)	9.5 (6.78 - 13.05)	-0.73 (-0.9 - -0.57)
Chile	Female	1037.29 (890.02 - 1304.09)	888.67 (692.08 - 1102.12)	-14.33	15.63 (13.38 - 19.44)	8.68 (6.75 - 10.73)	-2 (-2.37 - -1.63)
China	Female	91185.84 (41815.83 - 118332.73)	29613.1 (21119.87 - 37160.17)	-67.52	16.33 (7.51 - 21.16)	3.63 (2.64 - 4.68)	-6.51 (-7.11 - -5.91)
Colombia	Female	2354.14 (2033.53 - 3275.56)	2374.6 (1945.34 - 3265.89)	0.87	16.1 (14.04 - 22.57)	8.77 (7.16 - 12.09)	-2.66 (-2.87 - -2.45)
Comoros	Female	94.33 (63.33 - 130.57)	95.16 (62.61 - 141.1)	0.88	43.15 (29.76 - 58.75)	26.82 (17.8 - 39.73)	-1.82 (-1.9 - -1.74)
Congo	Female	179.66 (119.76 - 281.84)	299.4 (187 - 489.82)	66.65	18.23 (12.93 - 27.78)	13.97 (9.18 - 21.96)	-1.31 (-1.59 - -1.02)
Costa Rica	Female	481.86 (186.07 - 603.41)	483.66 (233.43 - 625)	0.37	33.84 (14.34 - 41.64)	18.74 (9.31 - 24.16)	-2.13 (-2.44 - -1.82)
Cote d'Ivoire	Female	755.36 (459.13 - 1044.89)	1344.5 (804.27 - 1971.69)	77.99	14.4 (9.56 - 18.98)	11.92 (7.47 - 16.8)	-0.84 (-0.99 - -0.7)
Croatia	Female	623.74 (458.57 - 716.63)	282.92 (230.07 - 347.41)	-54.64	21.79 (16.06 - 25.22)	10.35 (8.3 - 12.75)	-2.04 (-2.39 - -1.69)
Cuba	Female	187.58 (115.62 - 539.6)	1186.49 (251.65 - 1658.68)	532.52	3.28 (2.07 - 9.1)	17.14 (3.92 - 24.02)	6.83 (4.43 - 9.29)
Cyprus	Female	100.22 (50.5 - 148.78)	67.18 (49.44 - 91.4)	-32.97	24.29 (12.1 - 36.48)	9.39 (6.76 - 12.64)	-3.68 (-4.01 - -3.35)
Czech Republic	Female	2075.15 (1516.66 - 2297.85)	872.67 (701.49 - 1397.81)	-57.95	34.4 (25.78 - 38.72)	13.67 (10.56 - 23.09)	-3.89 (-4.1 - -3.68)
Democratic Republic of the Congo	Female	2353.66 (1506.3 - 3580.24)	3621.49 (2575.58 - 5373.49)	53.87	15.25 (10.57 - 22.07)	11.45 (8.31 - 16.63)	-1.17 (-1.27 - -1.08)
Denmark	Female	438.59 (298.49 - 499.68)	319.06 (225.69 - 382.89)	-27.25	14.25 (9.81 - 16.28)	9.21 (6.67 - 11.21)	-1.89 (-2.09 - -1.68)
Djibouti	Female	65.98 (39.84 - 96.67)	109.32 (62.92 - 179.54)	65.69	31.68 (19.88 - 45.08)	21.45 (12.45 - 34.86)	-1.62 (-1.81 - -1.42)
Dominica	Female	3.82 (3.09 - 4.88)	3.19 (2.59 - 4.24)	-16.49	10.27 (8.35 - 13.2)	8.94 (7.21 - 11.83)	-0.61 (-0.75 - -0.48)
Dominican Republic	Female	97.18 (55.22 - 198.37)	331.02 (129.56 - 518.71)	240.63	2.97 (1.75 - 6.11)	6.35 (2.45 - 9.97)	2 (1.22 - 2.8)
Ecuador	Female	1131.88 (901.27 - 1628.67)	1024.79 (801.02 - 1650.57)	-9.46	23.73 (19.58 - 33.16)	12.42 (9.8 - 19.45)	-2.16 (-2.53 - -1.78)
Egypt	Female	12574.47 (8384.87 - 16259.45)	13439.31 (8014.79 - 18462.95)	6.88	49.87 (34.84 - 63.81)	29.5 (18.15 - 39.6)	-1.88 (-2.04 - -1.71)
El Salvador	Female	488.84 (374.54 - 653.84)	329.46 (226.51 - 530.48)	-32.60	21.38 (16.37 - 27.95)	10 (6.92 - 16.03)	-3.17 (-3.38 - -2.95)
Equatorial Guinea	Female	35.43 (21.95 - 59.21)	34.54 (18.96 - 55.89)	-2.51	19.77 (13.01 - 31.91)	6.75 (3.72 - 10.95)	-4.53 (-4.89 - -4.16)
Eritrea	Female	489.83 (257.48 - 716.46)	855.41 (492.64 - 1327.36)	74.63	39.48 (23.42 - 55.1)	31.31 (18.35 - 47.82)	-0.97 (-1.05 - -0.89)
Estonia	Female	201.15 (129.13 - 232.86)	84.43 (56.54 - 108.67)	-58.03	22.63 (14.91 - 26.46)	10.9 (7.66 - 14.96)	-2.99 (-3.35 - -2.63)
Ethiopia	Female	14374.6 (9631.88 - 19975.39)	15527.73 (8220.42 - 26615.69)	8.02	64.59 (43.61 - 90.1)	32.8 (17.55 - 54.95)	-2.82 (-2.98 - -2.67)
Federated States of Micronesia	Female	3.98 (2.64 - 5.5)	3.4 (1.97 - 4.72)	-14.57	10.51 (7.1 - 14.51)	7.26 (4.29 - 10.03)	-1.51 (-1.64 - -1.38)
Fiji	Female	17.64 (12.58 - 26.63)	22.24 (16.34 - 29.23)	26.08	5.54 (4.01 - 8.46)	5.04 (3.72 - 6.63)	-0.21 (-0.44 - 0.02)
Finland	Female	478.16 (300.78 - 545.94)	296.12 (214.13 - 357.53)	-38.07	15.92 (10.28 - 18.26)	9.23 (6.75 - 11.41)	-1.96 (-2.29 - -1.63)
France	Female	4912.31 (4168.85 - 5932.26)	3234.58 (2671.94 - 4512.98)	-34.15	14.81 (12.31 - 17.76)	8.43 (6.83 - 12.06)	-1.85 (-2.05 - -1.65)
Gabon	Female	47.54 (35.64 - 68.48)	64.3 (43.93 - 96.85)	35.25	11.56 (8.64 - 17.02)	8.06 (5.47 - 12.21)	-1.32 (-1.63 - -1.01)
Georgia	Female	956.04 (488.32 - 1215.72)	420.55 (283.32 - 496.52)	-56.01	30.03 (15.69 - 38.09)	18.06 (12.57 - 21.47)	-2.66 (-3.33 - -1.99)
Germany	Female	10524.87 (5865.16 - 11934.14)	4119.83 (3287.98 - 5729.07)	-60.86	20.4 (12.1 - 23.25)	7.51 (5.79 - 11.61)	-3.89 (-4.28 - -3.5)

Ghana	Female	299.13 (208.23 - 444.14)	445.54 (320.9 - 681.18)	48.95	4.82 (3.52 - 6.66)	3.26 (2.46 - 4.69)	-1.61 (-1.75 - -1.47)
Greece	Female	1555.36 (1328.38 - 2402.66)	1739.64 (1361.91 - 2036.25)	11.85	24.13 (20.02 - 40.25)	23.56 (19.6 - 30.98)	-0.07 (-0.57 - 0.44)
Greenland	Female	2.33 (1.63 - 2.9)	1.15 (0.91 - 1.6)	-50.64	9.02 (5.9 - 11.11)	4.06 (3.22 - 5.64)	-3.06 (-3.31 - -2.8)
Grenada	Female	3.43 (2.52 - 4.7)	2.83 (2.23 - 4.45)	-17.49	8.52 (6.31 - 11.76)	4.79 (3.72 - 7.56)	-1.85 (-2.02 - -1.69)
Guam	Female	1.13 (0.88 - 1.51)	1.71 (1.15 - 2.11)	51.33	1.93 (1.48 - 2.54)	2.04 (1.38 - 2.53)	0.4 (0.17 - 0.64)
Guatemala	Female	739.84 (488.07 - 1213.24)	690.86 (518.5 - 1178.77)	-6.62	18.6 (13.48 - 31.63)	8.75 (6.67 - 14.44)	-2.58 (-2.96 - -2.2)
Guinea	Female	676.43 (392.25 - 949.58)	959.53 (545.51 - 1483.47)	41.85	22.72 (14.42 - 29.63)	17.12 (10.6 - 24.44)	-1.05 (-1.14 - -0.95)
Guinea-Bissau	Female	77.1 (50.38 - 107.2)	96.68 (66.49 - 141.65)	25.40	17.39 (11.68 - 24.06)	12 (8.39 - 17.14)	-1.45 (-1.53 - -1.38)
Guyana	Female	16.9 (8.55 - 56.73)	43.63 (27.82 - 57.01)	158.17	4.77 (2.52 - 15.63)	11.41 (7.33 - 14.77)	3.12 (1.65 - 4.61)
Haiti	Female	763.13 (534.31 - 1018.2)	1156.94 (627.74 - 1742.44)	51.60	28.02 (20 - 36.08)	20.81 (11.38 - 31.27)	-1.07 (-1.16 - -0.98)
Honduras	Female	30.16 (22.25 - 37.32)	54.76 (41.19 - 71.71)	81.56	1.5 (1 - 1.92)	1.26 (0.93 - 1.68)	-0.53 (-0.64 - -0.42)
Hungary	Female	1750.91 (878.96 - 2048.27)	512.4 (426.74 - 684.73)	-70.74	28.27 (14.22 - 33.53)	8.18 (6.78 - 10.81)	-4.69 (-4.86 - -4.53)
Iceland	Female	18.58 (14.78 - 22.1)	11.16 (8.94 - 19.37)	-39.94	13.73 (10.86 - 16.38)	5.93 (4.68 - 10.46)	-2.76 (-2.84 - -2.68)
India	Female	100108.79 (52258.3 - 140513.96)	75389.88 (57062.18 - 103254.42)	-24.69	24.16 (13.03 - 32.99)	11.09 (8.37 - 15.08)	-3.09 (-3.22 - -2.95)
Indonesia	Female	14041.2 (8202.52 - 17067.32)	11863.7 (8193.73 - 14431.48)	-15.51	16.28 (9.94 - 19.49)	9.15 (6.37 - 11.16)	-2.15 (-2.24 - -2.06)
Iran	Female	3052.06 (2227.27 - 5174.94)	4352.85 (2942.22 - 5080.61)	42.62	12.37 (9.4 - 20.62)	10.62 (7.07 - 12.31)	0.86 (0.23 - 1.49)
Iraq	Female	1583.12 (536.96 - 2748.57)	1209.14 (887.26 - 1678.16)	-23.62	21.64 (7.52 - 37.21)	6.04 (4.51 - 8.34)	-5 (-6.18 - -3.81)
Ireland	Female	328.91 (266.99 - 388.71)	287.87 (208.93 - 358.96)	-12.48	17.24 (14.2 - 20.45)	10.46 (7.73 - 13.33)	-1.65 (-1.92 - -1.38)
Israel	Female	545.25 (442.86 - 642.23)	590.28 (457.91 - 740.29)	8.26	21.75 (17.66 - 25.5)	12.84 (9.82 - 16.16)	-2.32 (-2.51 - -2.12)
Italy	Female	8292.05 (5988.54 - 9098.28)	4754.53 (3864.46 - 6096.84)	-42.66	24.24 (17.35 - 26.78)	12.9 (10.23 - 16.39)	-2.14 (-2.37 - -1.91)
Jamaica	Female	94.98 (43.02 - 118.83)	102.39 (53.49 - 143.65)	7.80	8.38 (3.92 - 10.46)	6.77 (3.61 - 9.41)	-0.12 (-0.83 - 0.59)
Japan	Female	1543.8 (1409.63 - 2095.55)	1725.32 (1181.97 - 1957.85)	11.76	2.11 (1.93 - 2.85)	1.99 (1.46 - 2.28)	0.31 (0.14 - 0.48)
Jordan	Female	94.14 (45.2 - 138.64)	106.2 (61.42 - 143.83)	12.81	6.56 (2.84 - 9.84)	2.35 (1.32 - 3.18)	-4.43 (-5 - -3.85)
Kazakhstan	Female	1623.72 (1216.91 - 2155.47)	1282.76 (1029.8 - 1576.99)	-21.00	18.85 (14.23 - 24.75)	13.23 (10.54 - 16.26)	-1.81 (-2.49 - -1.12)
Kenya	Female	1334.43 (904.67 - 1813.53)	2421.25 (1582.06 - 2997.61)	81.44	13.48 (9.31 - 18.06)	10.59 (6.81 - 13.03)	-1 (-1.14 - -0.87)
Kiribati	Female	1.5 (1.03 - 2.63)	2.5 (1.67 - 3.88)	66.67	4.82 (3.44 - 8.22)	4.71 (3.23 - 7.23)	-0.24 (-0.44 - -0.05)
Kuwait	Female	70.44 (49.98 - 84.83)	71.85 (56.48 - 92.64)	2.00	10.38 (7.54 - 12.23)	3.74 (2.98 - 4.67)	-2.64 (-3.61 - -1.67)
Kyrgyzstan	Female	323.7 (156.59 - 402.89)	171.49 (142.97 - 226)	-47.02	15.38 (7.52 - 19.12)	5.58 (4.68 - 7.08)	-2.96 (-3.41 - -2.51)
Laos	Female	481.35 (250.06 - 713.42)	455.56 (293.61 - 619.05)	-5.36	26.27 (14.43 - 37.69)	13.7 (8.9 - 18.58)	-2.56 (-2.75 - -2.37)
Latvia	Female	299.83 (184.9 - 350.13)	154.23 (81.76 - 203.39)	-48.56	19.34 (12.18 - 22.62)	13.3 (7.08 - 17.86)	-1.57 (-1.8 - -1.34)
Lebanon	Female	1448.91 (951.08 - 1922.24)	1701.64 (1138.37 - 2209.1)	17.44	76.75 (51.22 - 100.52)	39.52 (26.71 - 50.74)	-2.71 (-2.98 - -2.44)
Lesotho	Female	79.73 (56.3 - 120.76)	109.15 (69.97 - 178.99)	36.90	10.47 (7.51 - 15.97)	11.74 (7.53 - 18.65)	1.23 (0.58 - 1.88)
Liberia	Female	108.91 (69.62 - 152.64)	152.87 (107.06 - 213.4)	40.36	12.43 (8.32 - 16.87)	7.75 (5.43 - 10.59)	-2.21 (-2.55 - -1.87)
Libya	Female	1211.53 (808.14 - 1676.09)	2082.68 (1389.23 - 2815.51)	71.90	70.42 (48.16 - 97.33)	61.26 (41.42 - 82.79)	-0.59 (-0.86 - -0.31)
Lithuania	Female	516.11 (378.9 - 593.3)	195.48 (160.77 - 296.24)	-62.12	24.72 (18.27 - 28.66)	11.19 (8.83 - 18.89)	-3.72 (-4.21 - -3.22)
Luxembourg	Female	34.69 (29.28 - 44.56)	30.07 (22.46 - 51.26)	-13.32	14.86 (12.26 - 19.76)	8.73 (6.45 - 14.86)	-2.12 (-2.31 - -1.93)
Macedonia	Female	170.14 (122.18 - 211.53)	189.33 (114.16 - 244.17)	11.28	16.36 (11.73 - 20.38)	15.69 (9.56 - 20.41)	-0.76 (-1.07 - -0.45)
Madagascar	Female	1915.65 (1274.26 - 2506.13)	2557.06 (1723.4 - 3468.51)	33.48	34.75 (23.12 - 44.2)	21.67 (14.48 - 29.09)	-1.86 (-2.06 - -1.66)
Malawi	Female	780.74 (264.18 - 1235.71)	913.03 (568.4 - 1361.85)	16.94	17.2 (6.82 - 25.7)	10.68 (6.85 - 15.78)	-2.7 (-3.14 - -2.27)
Malaysia	Female	1066.57 (441.94 - 1580.21)	998.42 (381.42 - 1626.3)	-6.39	13.79 (5.77 - 20.11)	6.6 (2.52 - 10.67)	-2.75 (-3.08 - -2.41)
Maldives	Female	4.18 (2.17 - 7.2)	2.87 (2.03 - 3.67)	-31.34	5.11 (2.46 - 8.78)	1.56 (1.13 - 1.94)	-4.61 (-4.85 - -4.38)
Mali	Female	1152.81 (639.86 - 1595.01)	1402.22 (804.04 - 2136.43)	21.63	27.88 (17.31 - 36.36)	14.67 (9.23 - 20.78)	-2.67 (-2.85 - -2.49)
Malta	Female	51.43 (42.85 - 64.39)	41.67 (34.9 - 57.11)	-18.98	24.89 (20.84 - 32.07)	15.38 (12.54 - 21.83)	-1.67 (-1.86 - -1.49)
Marshall Islands	Female	1.52 (1.07 - 1.96)	2.13 (1.21 - 3.28)	40.13	9.43 (6.6 - 12.16)	8.47 (4.88 - 12.79)	-0.44 (-0.72 - -0.15)
Mauritania	Female	101.7 (71.79 - 133.58)	118.25 (86.86 - 166.05)	16.27	11.35 (8.04 - 14.89)	6.94 (5 - 9.51)	-1.94 (-2.04 - -1.85)
Mauritius	Female	38.03 (30.65 - 44.96)	68.56 (25.92 - 88.31)	80.28	6.99 (5.67 - 8.19)	9.52 (3.64 - 12.31)	0.77 (0.13 - 1.41)
Mexico	Female	7809.59 (6038.04 - 8743.47)	7320.53 (6114.57 - 9113.13)	-6.26	21.11 (16.37 - 23.34)	11.39 (9.53 - 14.16)	-2.17 (-2.43 - -1.91)
Moldova	Female	812.23 (619.85 - 955.72)	450.64 (325.46 - 540.92)	-44.52	33.96 (25.81 - 39.91)	20.95 (14.42 - 25.97)	-2.18 (-2.49 - -1.88)
Mongolia	Female	47.16 (26.03 - 100.4)	69.85 (50.86 - 111.3)	48.11	5.7 (3.39 - 11.41)	4.31 (3.24 - 6.56)	-1.46 (-1.98 - -0.94)
Montenegro	Female	88.64 (68.02 - 118.95)	69.68 (54.92 - 101.26)	-21.39	26.95 (20.62 - 36.02)	19.16 (14.93 - 27.8)	-1.69 (-2.07 - -1.31)
Morocco	Female	7748.31 (4711.6 - 10344.64)	7557.07 (4673.08 - 10567.88)	-2.47	63.71 (40.42 - 82.35)	41.65 (26.16 - 57.89)	-1.43 (-1.5 - -1.36)
Mozambique	Female	3156.03 (2177.99 - 4208.93)	3341.37 (2190.48 - 4667.72)	5.87	45.2 (32.03 - 59.35)	22.9 (15.06 - 31.67)	-2.93 (-3.27 - -2.58)
Myanmar	Female	6851.94 (3743.81 - 9520.35)	4229.99 (2887.91 - 5650.42)	-38.27	36.12 (20.5 - 49.6)	15.06 (10.31 - 20.07)	-3.39 (-3.73 - -3.06)
Namibia	Female	148.76 (82.41 - 214.54)	148.18 (84.34 - 224.96)	-0.39	24.45 (14.04 - 33.7)	12.86 (7.6 - 19.17)	-2.89 (-3.52 - -2.26)
Nepal	Female	2366.09 (1542.06 - 3382.96)	1972.47 (1112.79 - 3729.52)	-16.64	26.96 (18.16 - 38.11)	12.51 (7.25 - 23.21)	-2.86 (-3.14 - -2.58)
Netherlands	Female	1437.56 (1120.28 - 1630.77)	1057.88 (883.15 - 1339.34)	-26.41	16.46 (12.96 - 18.81)	10.13 (8.37 - 13.81)	-1.94 (-2.17 - -1.71)
New Zealand	Female	243.98 (140.11 - 284.26)	212.22 (113.86 - 257.05)	-13.02	12.59 (7.43 - 14.67)	8.4 (4.56 - 10.33)	-1.13 (-1.46 - -0.8)
Nicaragua	Female	138.43 (97.47 - 194.78)	184.6 (107.31 - 249.49)	33.35	8.36 (6.18 - 11.06)	6.16 (3.58 - 8.29)	-0.75 (-0.98 - -0.52)
Niger	Female	508.02 (299.31 - 769.17)	689.73 (467.12 - 983.13)	35.77	14.37 (9.55 - 19.82)	8.41 (5.92 - 11.69)	-2.46 (-2.65 - -2.27)
Nigeria	Female	41243.34 (19035.09 - 72790.89)	66049.27 (36916.63 - 103540.62)	60.15	97.51 (49.1 - 164.95)	67.39 (40.23 - 103.03)	-1.67 (-1.87 - -1.48)
North Korea	Female	810.9 (442.85 - 1209.77)	883.17 (518.53 - 1208)	8.91	7.38 (4.05 - 10.96)	6.01 (3.51 - 8.24)	-0.74 (-0.93 - -0.54)
Northern Mariana Islands	Female	0.54 (0.38 - 0.76)	0.47 (0.33 - 0.61)	-12.96	2.7 (1.95 - 3.63)	2.1 (1.54 - 2.69)	-0.81 (-1.08 - -0.54)
Norway	Female	321.13 (267.75 - 379.79)	216.36 (191.62 - 292.6)	-32.63	12.91 (10.98 - 15.51)	7.09 (6.19 - 9.9)	-2.11 (-2.22 - -2)
Oman	Female	168.76 (75.23 - 265.72)	179.52 (86.49 - 262.87)	6.38	26.48 (11.98 - 40.45)	11.85 (5.76 - 17.19)	-3.03 (-3.52 - -2.54)
Pakistan	Female	25986.43 (16963.94 - 36593.34)	57268.48 (35117.48 - 87353.11)	120.38	52.27 (35.69 - 71.51)	51.59 (32.39 - 78.16)	-0.19 (-0.42 - 0.04)
Palestine	Female	83.97 (40.27 - 173.86)	206.74 (140.18 - 308.73)	146.21	10.23 (5.14 - 20.67)	10.24 (7.31 - 14.29)	-0.1 (-0.39 - 0.19)
Panama	Female	69.71 (58.28 - 96.63)	132.82 (80.43 - 161.52)	90.53	6.57 (5.57 - 8.96)	6.76 (4.1 - 8.23)	0.82 (0.36 - 1.28)
Papua New Guinea	Female	224.92 (150.59 - 310.93)	428.5 (275.66 - 612.47)	90.51	13.93 (9.59 - 19.05)	10.97 (7.13 - 15.48)	-0.68 (-0.79 - -0.57)
Paraguay	Female	189.48 (145.33 - 250.63)	271.95 (184.08 - 366.07)	43.52	10.55 (8.05 - 14.31)	8.27 (5.43 - 11.15)	-0.55 (-0.88 - -0.22)
Peru	Female	709.02 (548.02 - 997.39)	707.94 (427.85 - 951.43)	-0.15	7.61 (5.79 - 9.92)	4.37 (2.6 - 5.87)	-1.9 (-2.06 - -1.74)
Philippines	Female	2284.29 (1881.73 - 3345.8)	2241.07 (1471.44 - 4989.04)	-1.89	7.97 (6.67 - 11.47)	4.6 (3.07 - 9.92)	-2.11 (-2.33 - -1.89)
Poland	Female	6693.07 (4896.19 - 7441.86)	2986.9 (2516.61 - 4053.65)	-55.37	31.2 (22.88 - 34.72)	12.75 (10.6 - 17.12)	-3.67 (-3.82 - -3.52)
Portugal	Female	918.48 (676.7 - 1043.98)	547.02 (349.08 - 661.45)	-40.44	15.61 (11.47 - 17.96)	7.53 (4.93 - 9.42)	-3 (-3.23 - -2.78)
Puerto Rico	Female	374.56 (209.64 - 455.56)	235.65 (159.92 - 286.25)	-37.09	19.41 (10.83 - 23.68)	10.78 (7.02 - 13.62)	-2.94 (-3.37 - -2.51)
Qatar	Female	2.84 (2.11 - 4.26)	9.3 (5.93 - 12.47)	227.46	2.8 (2.15 - 3.89)	1.74 (1.07 - 2.37)	-1.77 (-2.07 - -1.47)
Romania	Female	3157.89 (2133.15 - 3563.52)	1387.35 (1047.45 - 1630.62)	-56.07	25.44 (17.1 - 28.75)	12.24 (9.02 - 14.46)	-3.03 (-3.25 - -2.81)
Russian Federation	Female	24026.95 (14400.28 - 27708.43)	13956.9 (12104.42 - 17485.81)	-41.91	29.36 (17.33 - 34.13)	16.55 (14.77 - 21.41)	-2.62 (-3.08 - -2.16)
Rwanda	Female	1333.58 (834.79 - 1791.63)	1172.15 (728.21 - 1744.64)	-12.11	39.15 (24.77 - 51.79)	18.94 (11.84 - 27.58)	-3.3 (-3.64 - -2.95)
Saint Lucia	Female	4.91 (3.8 - 7.47)	5.33 (4.31 - 6.89)	8.55	7.67 (6.04 - 11.68)	5.45 (4.43 - 7)	-1.07 (-1.29 - -0.86)
Saint Vincent and the Grenadines	Female	2.42 (1.04 - 9.4)	7.58 (6.16 - 9.19)	213.22	4.9 (2.12 - 18.68)	13 (10.56 - 15.76)	2.17 (0.94 - 3.41)
Samoa	Female	10.97 (5.86 - 15.94)	9.47 (4.67 - 13.93)	-13.67	15.84 (9.46 - 21.9)	10.98 (5.63 - 16.05)	-1.45 (-1.57 - -1.34)
Sao Tome and Principe	Female	1.21 (0.83 - 2.04)	1.69 (1.14 - 2.69)	39.67	2.09 (1.48 - 3.47)	1.79 (1.22 - 2.76)	-0.49 (-0.64 - -0.35)
Saudi Arabia	Female	1192.64 (276.43 - 2249.09)	2127.98 (435.91 - 3789.33)	78.43	19.77 (4.74 - 36.64)	14.31 (2.99 - 24.57)	-0.68 (-1.07 - -0.28)
Senegal	Female	383.48 (263.86 - 528.93)	559.34 (407.59 - 817.72)	45.86	11.18 (8.06 - 15.12)	8.73 (6.54 - 12.41)	-1.06 (-1.17 - -0.96)
Serbia	Female	1289.9 (869.64 - 1650.95)	839.36 (487.62 - 1077.15)	-34.93	25.6 (17.2 - 32.74)	15.45 (8.87 - 20.3)	-1.74 (-1.97 - -1.51)
Seychelles	Female	6.04 (3.52 - 12.51)	7.74 (5.26 - 10.27)	28.15	17.37 (10.33 - 34.87)	14.91 (10.33 - 20.15)	-0.61 (-0.89 - -0.33)
Sierra Leone	Female	211.22 (135.86 - 294.2)	304.65 (206.45 - 426.13)	44.23	11.72 (8.14 - 15.85)	8.95 (6.11 - 12.36)	-0.96 (-1.05 - -0.86)
Singapore	Female	66.69 (44.24 - 78.33)	88.53 (46.81 - 116.75)	32.75	4.13 (2.82 - 4.77)	2.68 (1.47 - 3.51)	-0.61 (-1.15 - -0.07)

Slovakia	Female	757.99 (570.73 - 978.74)	531.95 (410.68 - 786.35)	-29.82	26.17 (19.78 - 34.08)	16.67 (12.77 - 24.35)	-1.78 (-1.96 - -1.6)
Slovenia	Female	215.88 (149.35 - 248.32)	107.12 (83.57 - 129.58)	-50.38	18.44 (12.93 - 21.35)	8.22 (6.68 - 10.53)	-3.22 (-3.54 - -2.89)
Solomon Islands	Female	13.32 (8.87 - 18.69)	23.45 (14.95 - 32.88)	76.05	11.11 (7.53 - 15.24)	9.01 (5.84 - 12.57)	-0.59 (-0.69 - -0.49)
Somalia	Female	1062.53 (372.11 - 1760.35)	2662.3 (1391.05 - 4381.67)	150.56	36.74 (15.42 - 58.58)	36.38 (19.34 - 59.99)	-0.43 (-0.66 - -0.2)
South Africa	Female	1504.16 (1070.06 - 1987.06)	1621.47 (1114.74 - 2056.88)	7.80	8.36 (6.02 - 10.77)	5.54 (3.82 - 7.01)	-1.52 (-2.63 - -0.4)
South Korea	Female	438.95 (326.93 - 929.33)	596.93 (348.76 - 733.12)	35.99	2 (1.51 - 4.17)	1.99 (1.21 - 2.46)	0.59 (0.39 - 0.8)
South Sudan	Female	668.1 (245.17 - 1181.6)	1028.82 (680.37 - 1420.21)	53.99	26.95 (11.37 - 44.75)	23.68 (15.6 - 33.19)	-0.77 (-1.07 - -0.47)
Spain	Female	3826.76 (2633.07 - 4294.74)	2455.28 (1696.68 - 2915.13)	-35.84	16.68 (11.51 - 18.79)	8.58 (6.18 - 10.45)	-2.45 (-2.54 - -2.37)
Sri Lanka	Female	1030.53 (802.7 - 1382.62)	842.22 (578.11 - 1275.11)	-18.27	13.94 (10.62 - 18.37)	6.71 (4.62 - 10.32)	-3.5 (-3.98 - -3.02)
Sudan	Female	1937.18 (1188.88 - 3090.76)	2524.76 (1716.36 - 3434.84)	30.33	22.52 (14.4 - 34.24)	14.38 (9.91 - 19.55)	-1.56 (-1.62 - -1.5)
Suriname	Female	27.83 (22.07 - 41.27)	39.42 (30.91 - 48.88)	41.65	14.96 (12.01 - 22.1)	13.22 (10.29 - 16.44)	-0.26 (-0.57 - 0.05)
Swaziland	Female	36.44 (24.81 - 55.76)	48.11 (30.35 - 79.71)	32.03	11.37 (7.99 - 17.16)	9.21 (5.85 - 14.52)	-0.07 (-0.76 - 0.62)
Sweden	Female	511.62 (413.23 - 586.06)	400.91 (310.81 - 481.52)	-21.64	9.62 (8.18 - 11.83)	6.56 (5.25 - 8.3)	-1.49 (-1.63 - -1.35)
Switzerland	Female	516.46 (362.07 - 603.1)	359.33 (285.58 - 492.25)	-30.42	13.04 (8.8 - 15.44)	6.98 (5.58 - 9.91)	-2.25 (-2.4 - -2.1)
Syria	Female	92.64 (58.17 - 122.41)	125.42 (65.69 - 188.23)	35.38	2.03 (1.13 - 2.77)	1.57 (0.78 - 2.39)	-0.93 (-1.12 - -0.74)
Tajikistan	Female	156.21 (121.53 - 218.28)	262.21 (202.4 - 381.9)	67.86	7.51 (5.48 - 9.74)	6.54 (4.87 - 8.69)	-0.78 (-0.91 - -0.65)
Tanzania	Female	3476.77 (1726.31 - 4964.17)	5518.45 (3427.05 - 8053.88)	58.72	29.12 (16.24 - 40.29)	20.96 (13.25 - 30.07)	-1.67 (-1.9 - -1.44)
Thailand	Female	2205.92 (1462.84 - 3696.54)	1324.32 (788.69 - 2581.98)	-39.97	7.69 (5.16 - 12.96)	3.29 (1.99 - 6.31)	-3.57 (-3.85 - -3.29)
The Bahamas	Female	18.91 (15.55 - 24.03)	22.75 (17.85 - 29.75)	20.31	14.43 (12.06 - 18.3)	10.92 (8.55 - 14.26)	-1.2 (-1.37 - -1.03)
The Gambia	Female	51.59 (30.84 - 72.15)	99.41 (64.02 - 145.95)	92.69	11.8 (7.45 - 15.79)	10.17 (7.02 - 14.33)	-0.38 (-0.53 - -0.23)
Timor-Leste	Female	64.56 (35.21 - 95.48)	60.98 (30 - 85.69)	-5.55	20.36 (11.99 - 27.49)	10.97 (5.8 - 15.31)	-2.53 (-2.66 - -2.4)
Togo	Female	209.88 (142.89 - 287.44)	269.76 (190.58 - 380.65)	28.53	12.83 (9.24 - 16.95)	7.98 (5.62 - 11.18)	-2.07 (-2.26 - -1.88)
Tonga	Female	2.04 (1.4 - 2.83)	1.94 (1.23 - 2.86)	-4.90	5.24 (3.61 - 7.18)	4.16 (2.61 - 6.14)	-0.91 (-1.01 - -0.8)
Trinidad and Tobago	Female	32.79 (25.5 - 38.86)	58.74 (20.64 - 89.05)	79.14	5.7 (4.44 - 6.71)	8.16 (2.73 - 12.43)	2.5 (1.69 - 3.32)
Tunisia	Female	1492.35 (1068.43 - 1913.71)	1303.43 (864.47 - 1811.06)	-12.66	38.41 (28.38 - 49.32)	21.48 (14.34 - 29.65)	-2.23 (-2.32 - -2.15)
Turkey	Female	5279.28 (3766.41 - 8331.29)	2947.47 (2267.79 - 4271.52)	-44.17	19.05 (13.82 - 29.67)	6.96 (5.36 - 10.03)	-3.82 (-3.95 - -3.69)
Turkmenistan	Female	394.5 (266.63 - 481.07)	392.02 (232.76 - 485.33)	-0.63	22.66 (16.05 - 27.18)	15.74 (9.47 - 19.44)	-1.15 (-1.57 - -0.72)
Uganda	Female	2772.78 (1832.36 - 3679.28)	4302.19 (2729.46 - 6857.88)	55.16	36.12 (25.07 - 46.04)	23.36 (14.92 - 36.83)	-2.07 (-2.3 - -1.84)
Ukraine	Female	7618.51 (5189.85 - 9642.67)	7817.69 (5257.04 - 12030.3)	2.61	26.16 (17.31 - 33.06)	33.25 (21.56 - 53.52)	0 (-0.56 - 0.57)
United Arab Emirates	Female	72.19 (49.18 - 109.82)	215.31 (138.33 - 314.86)	198.25	13.48 (9.39 - 19.45)	9.32 (6.06 - 13.67)	-1.41 (-1.49 - -1.34)
United Kingdom	Female	6092.33 (4339.18 - 6548.44)	4216.34 (3381.12 - 4821.49)	-30.79	17.99 (12.61 - 19.45)	10.71 (8.69 - 12.55)	-1.78 (-2.04 - -1.52)
United States	Female	26257.94 (18813.63 - 28620.06)	19123.51 (16511.77 - 29500.26)	-27.17	18.09 (12.9 - 19.77)	10.05 (8.48 - 15.92)	-2.43 (-2.55 - -2.32)
Uruguay	Female	340.36 (226.6 - 406.24)	238.85 (169.05 - 300.55)	-29.82	20.18 (13.14 - 24.4)	12.09 (8.25 - 15.34)	-1.59 (-1.95 - -1.22)
Uzbekistan	Female	1319.22 (1012.24 - 1858.85)	2173.8 (1679.02 - 2724.26)	64.78	14.07 (10.73 - 20.64)	13.22 (10.19 - 16.53)	-0.24 (-0.45 - -0.04)
Vanuatu	Female	8.73 (4.06 - 15.43)	18.15 (6.94 - 33.56)	107.90	15.03 (6.98 - 26.7)	14.56 (5.58 - 26.61)	-0.14 (-0.29 - 0.02)
Venezuela	Female	1289.71 (886.19 - 1581.11)	2085.42 (1009.33 - 2801.61)	61.70	16.3 (11.18 - 19.6)	13.09 (6.54 - 17.43)	-0.45 (-0.68 - -0.22)
Vietnam	Female	4432.19 (2743.2 - 5966.06)	3468.18 (2232.75 - 4736.72)	-21.75	13.81 (8.93 - 18.27)	6.68 (4.27 - 9.19)	-2.75 (-2.8 - -2.7)
Virgin Islands, U.S.	Female	1.77 (1.31 - 3.27)	2.48 (1.92 - 3.11)	40.11	3.31 (2.47 - 6.07)	3.83 (2.93 - 4.95)	1.13 (0.62 - 1.65)
Yemen	Female	1174.72 (558.37 - 1904.55)	2238.12 (1560.18 - 3139.34)	90.52	22.82 (11.78 - 35.62)	17.46 (12.17 - 24.52)	-1.06 (-1.16 - -0.96)
Zambia	Female	1500.6 (849.35 - 2109.37)	1914.25 (1268.88 - 2653.38)	27.57	41.56 (24.57 - 56.54)	23.68 (15.66 - 31.85)	-2.48 (-2.72 - -2.25)
Zimbabwe	Female	442.75 (248.21 - 611.84)	877.79 (448.08 - 1367.48)	98.26	10.25 (6.31 - 13.38)	12.88 (7 - 19.19)	2.18 (1.25 - 3.11)
Afghanistan	Male	3962.72 (1225.86 - 7014.93)	8278.12 (4927.03 - 13335.14)	108.90	90.95 (27.75 - 162.14)	69.31 (42.65 - 111.09)	-1 (-1.44 - -0.54)
Albania	Male	565.91 (427.49 - 1070.37)	363.24 (244.84 - 644.1)	-35.81	36.37 (27.57 - 70.3)	23.02 (15.53 - 40.87)	-1.99 (-2.3 - -1.69)
Algeria	Male	8362.26 (5528.01 - 11076.79)	7492.33 (4890.89 - 9409.52)	-10.40	69.04 (48 - 90.93)	37.12 (24.19 - 46.35)	-2.16 (-2.33 - -2)
American Samoa	Male	1.02 (0.79 - 1.6)	1.19 (0.81 - 1.48)	16.67	5.38 (4.19 - 8.09)	4.62 (3.11 - 5.74)	-0.22 (-0.5 - 0.07)
Andorra	Male	8.36 (6.1 - 11.23)	7.99 (5.93 - 11.81)	-4.43	24.82 (18.3 - 33.05)	15.12 (11.27 - 22.32)	-1.81 (-1.97 - -1.64)
Angola	Male	1893.51 (993.37 - 2934.45)	2346.41 (1717.03 - 3243.61)	23.92	43.65 (26.02 - 63.12)	23.55 (16.77 - 33.12)	-2.37 (-2.61 - -2.14)
Antigua and Barbuda	Male	3.5 (2.84 - 5.29)	4.41 (3.53 - 6.1)	26.00	13.12 (10.71 - 19.71)	9.42 (7.55 - 12.94)	-1.11 (-1.36 - -0.86)
Argentina	Male	5583.28 (4648.91 - 7992.73)	4965.34 (3877.28 - 7332.46)	-11.07	35.51 (29.57 - 50.64)	21.66 (16.94 - 31.99)	-1.97 (-2.15 - -1.8)
Armenia	Male	120.58 (87.3 - 230.95)	187.99 (83.62 - 239.92)	55.90	7.85 (5.77 - 14.54)	11.05 (5.08 - 14)	1.3 (0.4 - 2.21)
Australia	Male	1909.59 (1280.51 - 2245.31)	1712.81 (1267.3 - 2201.91)	-10.30	21.09 (14.17 - 24.77)	12.03 (8.96 - 15.87)	-1.94 (-2.14 - -1.74)
Austria	Male	1551.21 (895.94 - 1798.41)	714.93 (563.28 - 1217.83)	-53.91	35.74 (20.73 - 41.46)	12.36 (9.65 - 21.97)	-4.15 (-4.66 - -3.64)
Azerbaijan	Male	1655.3 (1133.96 - 2085.21)	1692.2 (1125.66 - 2178.67)	2.23	47.83 (33.86 - 60.93)	32.48 (21.63 - 41.43)	-1.35 (-1.6 - -1.09)
Bahrain	Male	100.89 (51.34 - 137.23)	110 (75.94 - 143.15)	9.03	49.8 (22.9 - 67.45)	12.63 (8.51 - 15.57)	-6.32 (-6.88 - -5.76)
Bangladesh	Male	40374.01 (26122.61 - 67160.9)	15513.9 (9935.28 - 30257.77)	-61.57	77.52 (51.59 - 130.79)	20.11 (12.94 - 39.35)	-5.09 (-5.19 - -5)
Barbados	Male	32.94 (25.58 - 43.03)	26.89 (21.23 - 37.29)	-18.37	26.38 (20.55 - 34.52)	15.88 (12.57 - 21.9)	-2.16 (-2.27 - -2.04)
Belarus	Male	2494.86 (1479.9 - 3206.11)	1464.22 (956.91 - 1924.42)	-41.31	48.17 (28.6 - 61.02)	27.09 (17.71 - 35.74)	-1.9 (-2.28 - -1.52)
Belgium	Male	1958.03 (1404.09 - 2492.31)	1114.48 (876.17 - 1836.76)	-43.08	33.43 (24.49 - 43.28)	15.54 (11.93 - 26.56)	-2.81 (-2.92 - -2.71)
Belize	Male	22.97 (15.77 - 32.23)	39.56 (27.58 - 50.01)	72.22	29.03 (20.21 - 41.33)	21.48 (15.38 - 27.1)	-1.55 (-1.84 - -1.26)
Benin	Male	662.36 (442.83 - 883.12)	829.82 (592.97 - 1145.93)	25.28	31.05 (21.98 - 39.44)	18.63 (13.45 - 24.78)	-1.94 (-2.05 - -1.83)
Bermuda	Male	7.12 (4.83 - 11.9)	6.57 (5.11 - 8.89)	-7.72	23.22 (15.81 - 38.73)	16.34 (12.53 - 23.31)	-1.28 (-1.43 - -1.13)
Bhutan	Male	178.22 (115.33 - 290.69)	102.02 (52.46 - 215.92)	-42.76	66.61 (42.16 - 111.43)	21.22 (11.29 - 44.76)	-4.34 (-4.43 - -4.25)
Bolivia	Male	1886.4 (1200.08 - 2375.74)	1386.26 (929.7 - 2059.99)	-26.51	66.72 (42.97 - 84.8)	27.05 (18.25 - 39.7)	-3.36 (-3.41 - -3.32)
Bosnia and Herzegovina	Male	600.18 (414.93 - 728.5)	359 (207.85 - 457.13)	-40.18	26.37 (17.97 - 32.01)	16.28 (9.85 - 20.66)	-1.97 (-2.33 - -1.6)
Botswana	Male	107.44 (74.6 - 159.3)	96.78 (71.42 - 143.46)	-9.92	24.47 (17.49 - 36.12)	10.6 (7.94 - 15.5)	-3.21 (-3.42 - -2.99)
Brazil	Male	20684.16 (16681.88 - 28107.1)	17373.48 (12873.55 - 24318.77)	-16.01	31.87 (25.34 - 42.45)	15.85 (11.81 - 22.14)	-2.45 (-2.52 - -2.39)
Brunei	Male	18.16 (12.56 - 33.89)	42.54 (30.22 - 54.52)	134.25	17.3 (12.12 - 31.88)	19.23 (13.55 - 24.14)	1.25 (0.85 - 1.64)
Bulgaria	Male	2486.6 (1985.07 - 3404.17)	1439.24 (1111.42 - 1890.84)	-42.12	50.66 (39.32 - 67.12)	33 (25.15 - 42.26)	-1.76 (-1.9 - -1.62)
Burkina Faso	Male	1341.58 (931.65 - 1832.31)	1738.65 (1202.51 - 2385.32)	29.60	31.42 (22.09 - 43.62)	20.83 (14.15 - 27.13)	-1.73 (-2.01 - -1.46)
Burundi	Male	2076.77 (1342.78 - 2818.35)	2281.87 (1580.02 - 3093.41)	9.88	84.68 (56.9 - 114.75)	46.81 (32.13 - 62.69)	-2.47 (-2.64 - -2.3)
Cambodia	Male	2053.89 (1401.85 - 2712.08)	1730.5 (1214.91 - 2585.07)	-15.75	57.54 (38.98 - 79.76)	26.06 (18.5 - 37.85)	-3.02 (-3.1 - -2.94)
Cameroon	Male	1278.17 (857 - 1664.08)	2202.89 (1496.12 - 3054.35)	72.35	28.43 (19.65 - 36.32)	19.96 (13.16 - 26.92)	-1.48 (-1.7 - -1.27)
Canada	Male	4377.27 (3331.33 - 5666.96)	3427.79 (2786.42 - 5384.72)	-21.69	29.66 (22.39 - 38.02)	16.45 (13.26 - 25.48)	-2.05 (-2.83 - -1.28)
Cape Verde	Male	7.49 (5.44 - 9.55)	10.34 (6.15 - 13.13)	38.05	6.07 (3.81 - 7.85)	4.12 (2.33 - 5.28)	-1.66 (-1.87 - -1.44)
Central African Republic	Male	476.61 (272.5 - 705.01)	782.05 (530.09 - 1140.19)	64.09	45.28 (28.61 - 67.3)	41.24 (28.89 - 59.14)	-0.61 (-0.72 - -0.5)
Chad	Male	755.82 (504.19 - 1017.14)	1412.61 (1033.56 - 1898.63)	86.90	27.98 (20.07 - 37.13)	23.06 (16.38 - 30.93)	-0.64 (-0.88 - -0.4)
Chile	Male	1843.18 (1304.48 - 2474.33)	1339.6 (1053.57 - 1913.3)	-27.32	30.72 (22.37 - 41.67)	13.5 (10.67 - 19.13)	-2.72 (-2.99 - -2.45)
China	Male	164713.04 (90250.56 - 198484.04)	61285.03 (40996.51 - 73271.46)	-62.79	28.46 (15.8 - 34.33)	7.23 (4.95 - 8.69)	-5.91 (-6.48 - -5.34)
Colombia	Male	5676.37 (4108.14 - 7500.55)	4240.2 (3329.62 - 6136.46)	-25.30	39.08 (29.07 - 50.22)	16.84 (13.19 - 24.37)	-3.45 (-3.67 - -3.22)
Comoros	Male	157.04 (116.05 - 227.17)	140.49 (95.08 - 214.7)	-10.54	73.72 (54.78 - 105.59)	41.94 (28.75 - 62.72)	-2.19 (-2.34 - -2.04)
Congo	Male	363.62 (258.64 - 515.95)	454.67 (292.57 - 711.8)	25.04	39.45 (29.68 - 54.43)	22.54 (14.53 - 35.26)	-2.45 (-2.69 - -2.21)
Costa Rica	Male	831.49 (375.12 - 1053.62)	735.29 (424.69 - 918.35)	-11.57	61.81 (30.23 - 76.77)	31.06 (18.29 - 38.84)	-2.86 (-3.18 - -2.53)
Cote d'Ivoire	Male	1628.4 (1025.35 - 2143.49)	2671.16 (1700.6 - 3855.24)	64.04	29.28 (19.57 - 37.31)	23.27 (15.67 - 32.34)	-1.06 (-1.26 - -0.87)
Croatia	Male	1101.89 (668.23 - 1279.3)	454.86 (363.36 - 617.3)	-58.72	40.58 (24.42 - 47.1)	16.55 (13.44 - 23.43)	-2.97 (-3.19 - -2.74)
Cuba	Male	466.47 (295.06 - 1073.28)	1794.99 (475.77 - 2633.83)	284.80	8.31 (5.27 - 19.1)	25.74 (6.76 - 37.77)	4.31 (2.38 - 6.27)
Cyprus	Male	108.18 (72.12 - 142.36)	105.91 (80.06 - 145.24)	-2.10	26.77 (17.88 - 35.08)	13.42 (10.33 - 18.86)	-2.89 (-3.28 - -2.49)
Czech Republic	Male	3506.54 (2387.79 - 4299.77)	1529.4 (1227.27 - 2433.72)	-56.38	63.16 (43.25 - 77.3)	21.97 (17.27 - 37.48)	-3.72 (-3.91 - -3.54)
Democratic Republic of the Congo	Male	4551.77 (3049.57 - 6693.95)	7427.56 (5281.76 - 10936.63)	63.18	30.58 (20.54 - 45.92)	24.17 (16.92 - 35.7)	-0.84 (-0.93 - -0.75)

Denmark	Male	902.51 (636 - 1220.23)	633.58 (507.67 - 910.14)	-29.80	30.03 (21.19 - 40.23)	16.36 (13.12 - 26)	-2.29 (-2.44 - -2.15)
Djibouti	Male	143.95 (72.5 - 214.39)	219.52 (143.14 - 345.59)	52.50	57.44 (33.36 - 84.34)	39.47 (26.15 - 60.84)	-1.72 (-2.02 - -1.43)
Dominica	Male	14.72 (11.03 - 22.05)	11.6 (9.33 - 16.01)	-21.20	43.46 (32.99 - 65.81)	30.41 (24.1 - 41.63)	-1.5 (-1.73 - -1.26)
Dominican Republic	Male	235.36 (165.64 - 445.72)	520.19 (233.72 - 722.56)	121.02	7.64 (5.48 - 14.95)	10.31 (4.51 - 14.4)	0.05 (-0.73 - 0.84)
Ecuador	Male	1621.77 (1295.23 - 2567.92)	1664.28 (1281.73 - 2754.58)	2.62	35.15 (28.78 - 56.41)	20.7 (16.12 - 33.72)	-1.6 (-1.84 - -1.36)
Egypt	Male	26308.73 (17849.33 - 35436.35)	27167.05 (17079.2 - 35182.27)	3.26	102.38 (71.92 - 140.17)	59.61 (37.53 - 76.89)	-1.63 (-1.8 - -1.47)
El Salvador	Male	1136.46 (850.19 - 1471.21)	634.2 (427.69 - 1089.13)	-44.20	52.29 (38.5 - 67.88)	23.76 (16.18 - 39.21)	-3.13 (-3.35 - -2.91)
Equatorial Guinea	Male	82.35 (51.18 - 126.22)	60.28 (38.63 - 92.87)	-26.80	50.91 (35.38 - 74.37)	12.34 (8.07 - 18.51)	-6.05 (-6.55 - -5.55)
Eritrea	Male	1529.15 (622.37 - 2345.07)	2354.11 (1418.17 - 3784.72)	53.95	125.89 (63.33 - 187.51)	89.57 (55.06 - 140.57)	-1.74 (-2.17 - -1.31)
Estonia	Male	386.94 (239.91 - 459.48)	147.81 (97.99 - 236.62)	-61.80	49.68 (30.69 - 58.75)	19.9 (13.15 - 34.49)	-3.93 (-4.35 - -3.5)
Ethiopia	Male	35095.06 (22667.52 - 47487.13)	31191.45 (19203.59 - 54044.23)	-11.12	151.3 (100.39 - 202.22)	68.41 (41.65 - 115.98)	-3.16 (-3.34 - -2.98)
Federated States of Micronesia	Male	9.71 (6.64 - 14.6)	7.77 (4.44 - 11.44)	-19.98	23.17 (15.57 - 35.29)	16.04 (9.39 - 23.53)	-1.32 (-1.38 - -1.26)
Fiji	Male	45.32 (28.3 - 58.7)	74.23 (30.62 - 109.02)	63.79	13.11 (8.49 - 16.6)	16.19 (6.84 - 23.63)	1.34 (0.97 - 1.71)
Finland	Male	837.21 (533.37 - 1170.6)	531.41 (430.44 - 789.58)	-36.53	30.27 (19.08 - 41.8)	15.16 (12.24 - 22.87)	-2.21 (-2.5 - -1.93)
France	Male	10833.84 (8608.2 - 15251.55)	6631.74 (5312.83 - 10374.1)	-38.79	34.36 (26.86 - 48.33)	16.82 (13.21 - 26.64)	-2.38 (-2.51 - -2.25)
Gabon	Male	117.09 (85.21 - 160.61)	145.98 (99.99 - 208.49)	24.67	29.33 (21.74 - 40.1)	20.22 (14.1 - 28.74)	-1.27 (-1.51 - -1.04)
Georgia	Male	2126.06 (1384.83 - 2533.26)	1083.94 (854.52 - 1596.9)	-49.02	77.85 (50.62 - 92.86)	51.15 (40.78 - 76.46)	-2 (-2.2 - -1.79)
Germany	Male	16940.95 (9949.15 - 19291.66)	8201.75 (6390.9 - 13490.74)	-51.59	36.59 (21.51 - 41.75)	14.52 (10.95 - 25.04)	-3.48 (-3.9 - -3.07)
Ghana	Male	428.16 (317.03 - 701.93)	627.97 (457.88 - 1014.41)	46.67	6.62 (4.8 - 11.27)	5.34 (3.96 - 8.12)	-0.5 (-0.62 - -0.38)
Greece	Male	3094.2 (2658.48 - 4687.55)	2663.83 (2030.27 - 3455.27)	-13.91	50.08 (42.38 - 78.73)	36.81 (29.94 - 52.42)	-1.13 (-1.53 - -0.72)
Greenland	Male	10.9 (5.39 - 14.44)	4.19 (2.93 - 5.16)	-61.56	37.23 (16.95 - 49.49)	11.52 (8.47 - 14.43)	-4.79 (-4.97 - -4.61)
Grenada	Male	11.89 (8.7 - 16.51)	8.38 (6.66 - 13.02)	-29.52	33.28 (24.54 - 44.24)	13.58 (10.77 - 21.02)	-3.01 (-3.25 - -2.77)
Guam	Male	8.2 (6.04 - 12.44)	10.82 (8.44 - 14.69)	31.95	12.27 (9.09 - 19.02)	12.13 (9.5 - 16.51)	0.65 (0.16 - 1.14)
Guatemala	Male	1315.43 (998.66 - 1975.69)	1233.29 (983.97 - 1823.11)	-6.24	36.49 (30.4 - 52.78)	17.66 (13.91 - 24.38)	-2.75 (-3.04 - -2.46)
Guinea	Male	1499.15 (851.26 - 2070.37)	1728.27 (1026 - 2298.84)	15.28	46.73 (31.12 - 60.51)	34.07 (21.56 - 45.41)	-1.08 (-1.23 - -0.93)
Guinea-Bissau	Male	192.13 (115.83 - 270.52)	202.79 (142.03 - 293.52)	5.55	45.02 (29.02 - 62.35)	28.29 (20.63 - 38.67)	-1.69 (-1.74 - -1.64)
Guyana	Male	38.82 (20.1 - 104.72)	66.56 (42.07 - 84.39)	71.46	12.38 (6.69 - 33.08)	18.63 (11.82 - 23.66)	1.67 (0.63 - 2.72)
Haiti	Male	1823.39 (1321.48 - 2915.57)	2255.05 (1351.46 - 3589.77)	23.67	71.16 (48.79 - 121.1)	47.95 (27.94 - 77.37)	-1.4 (-1.46 - -1.34)
Honduras	Male	277.24 (186.18 - 418.1)	296.53 (196.73 - 400.45)	6.96	15.28 (9.66 - 19.51)	8.52 (4.89 - 12)	-2.14 (-2.22 - -2.05)
Hungary	Male	2835.7 (1951.42 - 3678.57)	1027.72 (833.86 - 1651.92)	-63.76	49.74 (33.31 - 63.88)	16.82 (13.61 - 26.93)	-4.05 (-4.2 - -3.91)
Iceland	Male	27.53 (20.56 - 38.55)	22.9 (18.71 - 38.16)	-16.82	20.72 (15.46 - 29.01)	11.31 (9.17 - 18.89)	-1.93 (-2.12 - -1.73)
India	Male	199156.63 (126286.25 - 243459.88)	139331.95 (111342.38 - 189317.57)	-30.04	46.43 (29.49 - 55.65)	20.48 (16.29 - 27.79)	-3 (-3.19 - -2.81)
Indonesia	Male	27258.77 (18722.07 - 36095.81)	23538.84 (17095.66 - 33616.2)	-13.65	33.22 (22.79 - 45.14)	18.69 (13.55 - 26.49)	-1.97 (-2.09 - -1.85)
Iran	Male	5223.15 (3896.34 - 9523.38)	7124.45 (4494.13 - 8235.27)	36.40	20.47 (15.39 - 37.37)	16.96 (10.69 - 19.65)	0.66 (0.08 - 1.25)
Iraq	Male	2654.46 (1425.4 - 3562.61)	2101.98 (1617.62 - 3350)	-20.81	36.25 (20.23 - 47.9)	10.47 (8.12 - 16.68)	-4.85 (-5.83 - -3.86)
Ireland	Male	614.95 (428.66 - 728.36)	435.57 (332.93 - 623.1)	-29.17	33.67 (23.53 - 39.77)	15.8 (12.11 - 22.3)	-2.71 (-2.8 - -2.61)
Israel	Male	637.87 (519.24 - 869.32)	744.92 (588.65 - 1164.87)	16.78	27.66 (22.45 - 37.79)	16.35 (12.88 - 25.62)	-2.44 (-2.82 - -2.05)
Italy	Male	13048.43 (9460.67 - 16266.66)	7638.81 (5684.96 - 11447.35)	-41.46	39.78 (28.09 - 49.07)	19.72 (14.6 - 29.53)	-2.12 (-2.39 - -1.84)
Jamaica	Male	155.04 (71.62 - 201.3)	183.82 (88.89 - 262.96)	18.56	14.97 (7.24 - 19.12)	12.69 (6.2 - 18.14)	-0.66 (-1.14 - -0.17)
Japan	Male	3991.18 (3123.48 - 5300.69)	4140.91 (2816.01 - 4845.88)	3.75	5.47 (4.29 - 7.23)	4.41 (3.08 - 5.22)	-0.35 (-0.53 - -0.18)
Jordan	Male	190.36 (129.95 - 253.79)	241.73 (180.92 - 317.2)	26.99	12.12 (7.69 - 16.08)	4.84 (3.55 - 6.16)	-3.63 (-3.75 - -3.51)
Kazakhstan	Male	2612.03 (1873.19 - 3756.17)	1919.95 (1512.45 - 2598.69)	-26.50	34.4 (24.7 - 49.64)	21.87 (17.23 - 29.48)	-1.85 (-2.37 - -1.32)
Kenya	Male	2703.88 (1990.44 - 3948.1)	5784.85 (4089.59 - 7111.37)	113.95	27.1 (21.19 - 40.06)	27.55 (19.38 - 34.16)	0.36 (0.04 - 0.68)
Kiribati	Male	3.94 (3.09 - 5.51)	6.48 (4.22 - 8.5)	64.47	12.42 (10.05 - 17.78)	12.2 (8.28 - 15.95)	-0.19 (-0.41 - 0.03)
Kuwait	Male	131.36 (77.91 - 162.13)	134.04 (106.96 - 183.25)	2.04	14.19 (8.71 - 17.08)	5.76 (4.68 - 8.04)	-2.75 (-3.24 - -2.26)
Kyrgyzstan	Male	541.15 (298.67 - 668.06)	363.41 (290.01 - 540.91)	-32.84	28.4 (16.39 - 34.51)	12.89 (10.32 - 18.7)	-2.58 (-2.9 - -2.26)
Laos	Male	1163.89 (754.24 - 1509.9)	833.55 (545.52 - 1304.19)	-28.38	65.82 (43.18 - 88.84)	27.48 (17.88 - 43.08)	-3.31 (-3.44 - -3.18)
Latvia	Male	584.82 (367.17 - 703.43)	288.77 (159.15 - 378.4)	-50.62	43.35 (27.08 - 51.86)	26.51 (14.38 - 35)	-2.08 (-2.34 - -1.82)
Lebanon	Male	1636.32 (1142.29 - 2327.76)	2075.06 (1332.78 - 2704.36)	26.81	93.83 (67.7 - 131.08)	49.4 (31.68 - 63.04)	-2.29 (-2.58 - -2)
Lesotho	Male	196.84 (149.73 - 274.83)	273.22 (173.74 - 387.38)	38.80	29.78 (22.99 - 42.23)	35.46 (23.19 - 49.63)	1.32 (0.85 - 1.8)
Liberia	Male	292.05 (195.6 - 384.71)	302.42 (213.52 - 430.18)	3.55	28.61 (19.84 - 36.9)	15.86 (11.06 - 21.88)	-2.45 (-2.83 - -2.08)
Libya	Male	1356.72 (898.09 - 1920.09)	1869.67 (1140.64 - 2560.24)	37.81	72.5 (49.81 - 103.02)	52.78 (32.93 - 71.38)	-1.04 (-1.3 - -0.78)
Lithuania	Male	788.46 (506.7 - 950.09)	370.24 (281.31 - 487.46)	-53.04	42.14 (26.99 - 50.85)	23.31 (17.56 - 31.22)	-2.59 (-2.94 - -2.24)
Luxembourg	Male	61.62 (41.51 - 82.04)	45.03 (32.71 - 88.32)	-26.92	27.67 (18.47 - 37.2)	12.17 (8.7 - 24.87)	-3.35 (-3.57 - -3.13)
Macedonia	Male	461.2 (222.54 - 613.72)	328.34 (190.09 - 427.54)	-28.81	44.42 (21.74 - 58.76)	24.01 (13.82 - 31.25)	-3.03 (-3.49 - -2.58)
Madagascar	Male	5058.65 (3757.94 - 6890.81)	5125.63 (3518.8 - 7206.82)	1.32	80.62 (62.08 - 108.49)	44.28 (30.51 - 61.23)	-2.49 (-2.88 - -2.09)
Malawi	Male	1691.7 (466.89 - 2680.44)	2252.69 (1577.77 - 3155.87)	33.16	36.68 (12.44 - 56.2)	29.92 (20.95 - 39.29)	-1.21 (-1.57 - -0.85)
Malaysia	Male	2086.48 (1224.58 - 2841.45)	2259.32 (1210.77 - 3066.54)	8.28	27.33 (17.05 - 36.06)	14.09 (7.65 - 19.02)	-2.58 (-2.87 - -2.3)
Maldives	Male	14.43 (10.53 - 20.49)	9.82 (7.05 - 14.44)	-31.95	16.58 (12.49 - 22.37)	4.04 (3.13 - 5.66)	-5.31 (-5.45 - -5.17)
Mali	Male	2925.31 (1646.11 - 3981.53)	3236.32 (2079.4 - 4458.08)	10.63	65.12 (42.29 - 83.5)	31.96 (22.48 - 43.01)	-2.59 (-2.87 - -2.31)
Malta	Male	65.29 (54.9 - 93.82)	62.81 (51.33 - 93.69)	-3.80	33.77 (28.38 - 48.44)	21.96 (17.85 - 33.07)	-1.62 (-1.79 - -1.46)
Marshall Islands	Male	3.84 (2.86 - 4.96)	4.79 (2.97 - 6.25)	24.74	22.78 (17.07 - 29.28)	18.46 (11.61 - 23.75)	-0.59 (-0.74 - -0.43)
Mauritania	Male	216.31 (153.28 - 286.84)	211.26 (139.8 - 329.46)	-2.33	25 (18.01 - 32.46)	13.2 (8.81 - 20.21)	-2.39 (-2.5 - -2.29)
Mauritius	Male	107.47 (68.86 - 126.67)	79.69 (49.59 - 98.29)	-25.85	20.83 (13.67 - 24.19)	10.96 (6.81 - 13.55)	-2.89 (-3.22 - -2.56)
Mexico	Male	15062.67 (10824.78 - 18067.34)	13013.07 (10380.29 - 18186.32)	-13.61	41.83 (30.53 - 51.26)	21.74 (17.3 - 30.53)	-2.42 (-2.73 - -2.1)
Moldova	Male	1528.2 (993.71 - 2180.42)	714.35 (593.07 - 1099.6)	-53.26	71.8 (46.92 - 101.59)	34.78 (28.24 - 53.75)	-3.16 (-3.44 - -2.89)
Mongolia	Male	98.71 (76.38 - 142.96)	117.46 (85.17 - 148.35)	19.00	13.9 (8.58 - 18.66)	7.95 (5.49 - 9.96)	-2.8 (-3.16 - -2.44)
Montenegro	Male	225.43 (176.42 - 305.59)	155.64 (122.21 - 225.02)	-30.96	70.62 (55.34 - 95.99)	41.3 (32.51 - 59.72)	-2.47 (-2.81 - -2.13)
Morocco	Male	9931.82 (6092.36 - 13014.58)	8556.69 (5355.85 - 11643.57)	-13.85	81.29 (52.85 - 103.3)	47.63 (30 - 64.56)	-2.03 (-2.11 - -1.95)
Mozambique	Male	6371.4 (4544.55 - 9272)	8565.86 (6043.79 - 11342.06)	34.44	93.4 (71.21 - 138.39)	67.43 (45.28 - 89.58)	-0.98 (-1.22 - -0.73)
Myanmar	Male	11013.85 (7188.54 - 14682.85)	6691.46 (4653.6 - 9626)	-39.25	62.68 (42.76 - 84.87)	27.48 (19.35 - 39.19)	-3.13 (-3.34 - -2.92)
Namibia	Male	254.01 (166.53 - 329.69)	273.76 (157.77 - 378.72)	7.78	46.38 (33.87 - 59.24)	28.05 (18.21 - 37.16)	-1.97 (-2.47 - -1.48)
Nepal	Male	5364.56 (3346.34 - 8725.41)	3032.55 (1696.8 - 6033.83)	-43.47	62.42 (40.53 - 103.52)	22.81 (12.72 - 45.33)	-3.77 (-4.1 - -3.44)
Netherlands	Male	2471.38 (1702.78 - 2899.84)	1621.95 (1271.41 - 2290.41)	-34.37	29.13 (20.14 - 34.24)	14.49 (11.28 - 21.4)	-2.41 (-2.54 - -2.29)
New Zealand	Male	346.98 (194.99 - 404.01)	309.65 (178.33 - 374.15)	-10.76	19.4 (10.95 - 22.57)	12.24 (7.13 - 14.8)	-1.74 (-1.87 - -1.61)
Nicaragua	Male	359.82 (225.25 - 450.03)	293.9 (182.3 - 374.23)	-18.32	21.87 (13.98 - 27.01)	10.65 (6.65 - 13.49)	-2.86 (-3.02 - -2.71)
Niger	Male	1570.88 (902.44 - 2369.56)	1628.46 (1174.49 - 2305.89)	3.67	37.75 (25.83 - 50.27)	19.54 (13.88 - 27.19)	-2.83 (-3.06 - -2.6)
Nigeria	Male	57908.47 (35793.52 - 80417.25)	74990.06 (50038.97 - 106326.95)	29.50	124.66 (83.97 - 168.92)	75.84 (53.27 - 107.33)	-2.3 (-2.5 - -2.1)
North Korea	Male	1418.44 (891.18 - 1992.32)	1970.76 (1260.98 - 2588.38)	38.94	15.94 (9.96 - 22.17)	14.4 (9.47 - 18.73)	-0.26 (-0.53 - 0.01)
Northern Mariana Islands	Male	1.26 (0.83 - 2.19)	1.38 (0.95 - 1.77)	9.52	5.82 (4.12 - 9.4)	5.18 (3.62 - 6.7)	0.05 (-0.25 - 0.35)
Norway	Male	566.28 (484.67 - 851.4)	392.49 (325.23 - 664.66)	-30.69	23.23 (20.01 - 34.82)	11.93 (9.79 - 20.47)	-2.33 (-2.46 - -2.2)
Oman	Male	416.11 (246.47 - 571.57)	525.13 (319.41 - 720.83)	26.20	45.58 (27.67 - 60.99)	19.56 (11.35 - 26.23)	-3.01 (-3.46 - -2.56)
Pakistan	Male	63913.35 (44070.8 - 89380.5)	91722.77 (65489.46 - 124433.38)	43.51	115.2 (81.46 - 159.16)	85.4 (61.84 - 115.59)	-1.33 (-1.56 - -1.11)
Palestine	Male	213.14 (115.19 - 450.89)	412.7 (300.51 - 822.58)	93.63	28.29 (16.27 - 56.07)	20.15 (15.29 - 36.93)	-1.18 (-1.5 - -0.85)
Panama	Male	154.33 (113.61 - 190.44)	238.04 (133.56 - 295.88)	54.24	13.91 (10.47 - 17.4)	12.08 (6.81 - 14.99)	-0.06 (-0.42 - 0.3)
Papua New Guinea	Male	623.91 (452.34 - 838.34)	1150.18 (735.99 - 1559.36)	84.35	34.71 (25.16 - 46.9)	27.3 (17.77 - 36.68)	-0.58 (-0.73 - -0.43)
Paraguay	Male	452.35 (300.84 - 694.83)	645.35 (437.33 - 871.21)	42.67	26.14 (17.8 - 39.96)	19.84 (13.23 - 26.55)	-0.89 (-0.99 - -0.78)
Peru	Male	2388.49 (1916.19 - 3474.3)	1582.25 (1157.89 - 2183.53)	-33.76	24.12 (19.36 - 34.83)	9.94 (7.21 - 13.54)	-3.23 (-3.33 - -3.13)

Philippines	Male	5223.12 (4180.89 - 8363.01)	4690.24 (2942.26 - 10458.15)	-10.20	18.68 (15.33 - 29.35)	9.89 (6.34 - 21.52)	-2.32 (-2.51 - -2.14)
Poland	Male	12721.99 (7947.08 - 14282.2)	5538.85 (4333.46 - 7283)	-56.46	63.16 (40.07 - 70.87)	22.95 (18.32 - 30.92)	-4 (-4.16 - -3.84)
Portugal	Male	1947.62 (1574.69 - 3314.83)	1111.4 (872.18 - 1849.72)	-42.94	36.29 (29.53 - 60.81)	16.4 (12.65 - 27.2)	-3.21 (-3.35 - -3.08)
Puerto Rico	Male	543.86 (366.04 - 681.99)	442.09 (267.97 - 531.51)	-18.71	31.35 (21.09 - 39.36)	20.99 (12.97 - 25.26)	-1.69 (-1.99 - -1.38)
Qatar	Male	24.32 (16.3 - 43.53)	126.25 (68.82 - 221.02)	419.12	10.75 (7.69 - 18.4)	6.45 (3.82 - 10.06)	-1.8 (-2.06 - -1.54)
Romania	Male	5808.44 (3335.69 - 6740.4)	2589.34 (1809.5 - 3107.94)	-55.42	47.53 (27.04 - 55.31)	21.99 (15 - 26.39)	-3.27 (-3.47 - -3.06)
Russian Federation	Male	37468.96 (21774.04 - 46769.81)	20668.38 (16458.12 - 27644.51)	-44.84	49.94 (29.04 - 61.83)	25.95 (20.75 - 35.12)	-2.86 (-3.33 - -2.39)
Rwanda	Male	2625.96 (1728.48 - 3536.47)	1999.21 (1357.63 - 2950.75)	-23.87	79.64 (55.15 - 107.49)	37.12 (25.74 - 52.22)	-3.71 (-4.18 - -3.24)
Saint Lucia	Male	23.54 (19.25 - 35.09)	28.98 (21.55 - 39.21)	23.11	43.15 (35.76 - 64.98)	29.62 (21.75 - 39.78)	-1.41 (-1.65 - -1.17)
Saint Vincent and the Grenadines	Male	8.97 (4.59 - 25.65)	18.95 (14.14 - 24.3)	111.26	19.7 (10.25 - 55.1)	29.94 (22.23 - 38.24)	0.6 (-0.13 - 1.33)
Samoa	Male	20.56 (13.59 - 26.81)	15.85 (10.47 - 21.88)	-22.91	29.05 (19.91 - 37.8)	17.76 (11.93 - 23.93)	-1.94 (-2.09 - -1.78)
Sao Tome and Principe	Male	2.7 (1.68 - 5.48)	2.95 (1.82 - 5.91)	9.26	4.87 (3.33 - 9.39)	3.59 (2.28 - 6.46)	-1.14 (-1.22 - -1.07)
Saudi Arabia	Male	2754.99 (905.84 - 4371.45)	4262.31 (1145.45 - 6844.94)	54.71	35.23 (13.03 - 55.7)	21.48 (6.07 - 33.22)	-1.2 (-1.54 - -0.86)
Senegal	Male	993.09 (689.75 - 1279.45)	1203.21 (845.36 - 1788.27)	21.16	29.19 (20.81 - 36.93)	20.5 (14.38 - 29.08)	-1.3 (-1.36 - -1.25)
Serbia	Male	2809.35 (1701.05 - 3683.06)	1613.6 (947.01 - 2046.41)	-42.56	54.11 (32.65 - 70.75)	29.12 (16.75 - 37.64)	-2.29 (-2.51 - -2.06)
Seychelles	Male	8.2 (6.45 - 12.57)	8.43 (6.67 - 11.69)	2.80	26.76 (21.09 - 39.87)	14.53 (11.5 - 20.14)	-2.62 (-2.78 - -2.46)
Sierra Leone	Male	572.98 (346.93 - 811.7)	619.85 (434.22 - 817.9)	8.18	30.34 (20.07 - 40.97)	18.62 (12.76 - 24.24)	-1.86 (-1.93 - -1.79)
Singapore	Male	158.52 (120.08 - 216.33)	169.76 (123.5 - 216.62)	7.09	10.05 (7.79 - 14.22)	5.24 (3.78 - 6.58)	-1.81 (-2.08 - -1.54)
Slovakia	Male	1111.71 (809.27 - 1393.75)	727.22 (500.89 - 887.54)	-34.59	41.34 (30.09 - 51.62)	21.56 (14.79 - 26.75)	-1.97 (-2.12 - -1.82)
Slovenia	Male	394.11 (286.7 - 507.16)	226.89 (169.78 - 309.71)	-42.43	37.42 (26.99 - 47.34)	17.05 (13.07 - 24.01)	-3.25 (-3.43 - -3.06)
Solomon Islands	Male	32.73 (22.28 - 46.98)	52.73 (32.94 - 78.36)	61.11	24.51 (16.88 - 35.14)	19.35 (12.13 - 28.84)	-0.56 (-0.72 - -0.4)
Somalia	Male	2500.43 (611.55 - 4376.9)	5702.68 (3223.59 - 9952.2)	128.07	72.37 (24.35 - 124.71)	74.03 (44.09 - 121.14)	-0.36 (-0.68 - -0.03)
South Africa	Male	2555.05 (1887.81 - 3317.03)	3124.49 (2144.04 - 3966.87)	22.29	17.8 (13.3 - 22.75)	12.7 (8.7 - 15.81)	-1.48 (-2.11 - -0.84)
South Korea	Male	1185.33 (927.41 - 2162.39)	1356.95 (760.49 - 1768.09)	14.48	6.09 (4.87 - 10.91)	3.92 (2.24 - 5.09)	-0.99 (-1.2 - -0.79)
South Sudan	Male	1491.58 (448.99 - 2643.97)	2517 (1628.03 - 3647.85)	68.75	52.85 (20.27 - 89.53)	55.57 (37.48 - 78.1)	-0.06 (-0.33 - 0.22)
Spain	Male	8428.35 (5820.81 - 10159.46)	4687.78 (3687.96 - 6931.93)	-44.38	38.71 (26.32 - 46.45)	15.76 (12.41 - 23.91)	-3.23 (-3.36 - -3.1)
Sri Lanka	Male	3107.19 (2550.41 - 4485.6)	1976.59 (1458.86 - 2732.57)	-36.39	41.2 (33.27 - 57.9)	17.28 (12.92 - 23.9)	-3.45 (-3.78 - -3.12)
Sudan	Male	4297.61 (2507.03 - 7375.46)	4907.23 (3417.67 - 7329.07)	14.19	50.2 (30.21 - 83.65)	28.58 (19.53 - 42.71)	-1.99 (-2.07 - -1.91)
Suriname	Male	59.11 (47.24 - 90.06)	84.12 (61.75 - 109.21)	42.31	32.87 (26.61 - 49.43)	28.82 (21.12 - 37.05)	-0.44 (-0.8 - -0.08)
Swaziland	Male	73.92 (55.58 - 101.58)	123.7 (80.09 - 164.52)	67.34	29.72 (22.64 - 41.37)	29.64 (19.25 - 39.56)	0.84 (0.07 - 1.62)
Sweden	Male	885.46 (710.87 - 1224.68)	626.64 (520.02 - 888.87)	-29.23	17.11 (13.79 - 24.15)	9.81 (8.16 - 14.12)	-1.73 (-1.92 - -1.53)
Switzerland	Male	829.72 (536.44 - 1108.97)	584.42 (433.31 - 947.21)	-29.56	20.95 (13.25 - 27.63)	10.27 (7.74 - 18.12)	-2.52 (-2.64 - -2.39)
Syria	Male	312.6 (237.93 - 453.18)	339.39 (201.34 - 469.52)	8.57	6.7 (4.63 - 8.37)	4.16 (2.32 - 5.81)	-1.93 (-2.2 - -1.66)
Tajikistan	Male	213.29 (159.94 - 336.55)	333.58 (263.23 - 475.36)	56.40	11.18 (8.2 - 15.49)	8.92 (6.61 - 11.34)	-1.15 (-1.35 - -0.96)
Tanzania	Male	5876.8 (2134.6 - 9175.94)	9897.41 (6663.36 - 15172.95)	68.41	49.07 (20.88 - 75.05)	40.12 (27.75 - 59.02)	-0.89 (-1.04 - -0.75)
Thailand	Male	7122.86 (5600.44 - 10513.86)	4615.33 (3415.96 - 7587.8)	-35.20	27.94 (22.54 - 40.03)	11.03 (7.99 - 19.17)	-4.21 (-4.54 - -3.88)
The Bahamas	Male	26.82 (20.12 - 34.12)	32.1 (22.64 - 43.94)	19.69	23.86 (17.98 - 30.41)	16.51 (11.59 - 22.64)	-1.74 (-1.98 - -1.5)
The Gambia	Male	138.72 (91.94 - 198.69)	211.48 (132.72 - 353.15)	52.45	31.72 (21.74 - 44.77)	23.6 (16 - 37.28)	-0.8 (-0.94 - -0.67)
Timor-Leste	Male	156.46 (102.17 - 234.55)	134.46 (69.73 - 232.37)	-14.06	45.69 (28.01 - 73.4)	24.39 (12.57 - 43.04)	-2.51 (-2.66 - -2.36)
Togo	Male	424.49 (291.96 - 563.22)	541.37 (376.58 - 745.49)	27.53	27.13 (19.52 - 35.1)	18.74 (12.72 - 25.33)	-1.45 (-1.63 - -1.26)
Tonga	Male	3.87 (2.74 - 5.69)	4.76 (3.04 - 6.89)	23.00	10.05 (7.11 - 14.36)	10.23 (6.54 - 14.83)	0.3 (0.15 - 0.45)
Trinidad and Tobago	Male	90.82 (71.12 - 121.43)	128.16 (71.42 - 179.23)	41.11	16.68 (13.14 - 22.17)	15.96 (9.02 - 22.26)	0.29 (-0.09 - 0.66)
Tunisia	Male	1584.57 (1144.71 - 2206.81)	1492.75 (893.43 - 2061.12)	-5.79	39.57 (29.04 - 56.3)	25.06 (14.98 - 34.35)	-1.49 (-1.77 - -1.21)
Turkey	Male	10249.9 (7048.99 - 18605.65)	6732.52 (5015.88 - 10467)	-34.32	38.92 (26.98 - 69.11)	16.03 (11.94 - 25.18)	-3.61 (-3.93 - -3.29)
Turkmenistan	Male	496.21 (283.6 - 622.52)	655.89 (339.82 - 831.09)	32.18	29.89 (18.46 - 36.72)	25.41 (13.74 - 31.79)	-0.21 (-0.66 - 0.24)
Uganda	Male	3893.6 (2621.12 - 5723.48)	8035.16 (5097.19 - 11658.64)	106.37	45.42 (30.57 - 69.67)	48.57 (31.52 - 66.5)	0.12 (-0.14 - 0.37)
Ukraine	Male	11476.97 (7705.38 - 15796.36)	12882.5 (9922.93 - 20773.13)	12.25	43.47 (29.33 - 59.42)	54.52 (41.87 - 91.01)	0.05 (-0.5 - 0.61)
United Arab Emirates	Male	317.88 (202.04 - 558.28)	1383.62 (931.86 - 2111)	335.26	30.63 (21.31 - 50.64)	21 (13.89 - 32.84)	-1.23 (-1.37 - -1.09)
United Kingdom	Male	11445.22 (7938.8 - 13512.78)	7328.07 (5545.01 - 9419.05)	-35.97	35.22 (24.09 - 40.85)	17.79 (13.56 - 23.41)	-2.56 (-2.7 - -2.41)
United States	Male	37781.39 (23592.24 - 41906.84)	26758.1 (22177.62 - 41945.96)	-29.18	27.74 (17.43 - 30.76)	14.4 (11.63 - 23.29)	-2.55 (-2.71 - -2.4)
Uruguay	Male	686.73 (471.72 - 803.68)	494.81 (324.3 - 623.31)	-27.95	41.8 (29.07 - 48.99)	26.23 (17.25 - 33.03)	-1.6 (-1.74 - -1.45)
Uzbekistan	Male	3219.98 (2436.5 - 4483.6)	3675.95 (2954.05 - 5265.03)	14.16	34.55 (26.14 - 55.72)	24.41 (19.62 - 35.38)	-1.27 (-1.43 - -1.11)
Vanuatu	Male	20.5 (10.36 - 39.36)	40.84 (18.1 - 82.33)	99.22	33.65 (17.07 - 65.69)	33.04 (14.39 - 67.1)	0.08 (-0.08 - 0.23)
Venezuela	Male	2584.98 (1984.14 - 3361.17)	3691.76 (2174.94 - 4941.35)	42.82	33.44 (25 - 42.6)	23.98 (14.36 - 32.34)	-1.16 (-1.3 - -1.03)
Vietnam	Male	8865.03 (6301.23 - 12115.56)	9821.65 (5628.01 - 12759.33)	10.79	32.75 (23.92 - 45.74)	20.51 (11.82 - 26.35)	-1.5 (-1.65 - -1.35)
Virgin Islands, U.S.	Male	5.61 (4.17 - 10.2)	8.44 (5.86 - 10.76)	50.45	11.62 (8.75 - 20.89)	12.42 (8.52 - 15.69)	0.72 (0.29 - 1.16)
Yemen	Male	2864.49 (1016.93 - 5022.11)	4149.79 (2930.65 - 6658.35)	44.87	55.27 (23.26 - 98.62)	34.42 (24 - 54.79)	-1.94 (-2.05 - -1.82)
Zambia	Male	2535.19 (1249.88 - 3649.83)	3877.79 (2578.77 - 5724.65)	52.96	68.98 (40.14 - 96.98)	50.28 (33.95 - 71.25)	-1.67 (-2.12 - -1.22)
Zimbabwe	Male	831.85 (487.4 - 1105.32)	2291.12 (1077.9 - 3220.17)	175.42	20.87 (14.13 - 27.52)	38.43 (20.63 - 51.74)	3.33 (2.11 - 4.57)

DALY, disability adjusted life-year; CI, confidence interval; EAPC, estimated annual percentage change; UI, uncertainty interval.

Supplementary Table S6. Age distribution of incidence (per 100,000) for Hodgkin lymphoma in different countries in 2017.

country	Under 5	5 to 9	10 to 14	15 to 19	20 to 24	25 to 29	30 to 34	35 to 39	40 to 44	45 to 49	50 to 54	55 to 59	60 to 64	65 to 69	70 to 74	75 to 79	80 to 84	85 to 89	90 to 94	95 plus
Afghanistan	0.200132	0.337511	0.472548	0.93228	1.574619	2.12365	2.015984	1.734358	1.714445	1.860676	2.505189	3.40262	3.7182	4.520876	5.592304	4.853602	5.208476	5.31825	3.532109	3.367222
Albania	0.634586	1.129193	1.189655	2.572771	4.941477	4.553514	3.44748	2.172863	1.819021	1.395601	1.319513	1.364421	1.314399	1.406982	1.660487	1.356712	1.147701	1.791924	1.285965	0.941286
Algeria	0.210099	0.969251	1.332313	2.037779	2.75938	2.608007	2.356297	1.806877	1.353707	1.266035	1.372573	1.559617	1.885931	2.465717	2.927014	2.864736	3.138911	3.330561	1.870006	1.380776
American Samoa	0.042814	0.058768	0.043251	0.121448	0.434336	0.384727	0.429411	0.1813	0.168165	0.245861	0.261831	0.292883	0.415798	0.418805	0.685906	0.577307	0.507328	0.842492	1.102584	0.967196
Andorra	0.3161	0.366999	0.794838	3.392219	7.449156	7.96771	7.204855	5.774006	3.631428	3.868819	3.917463	3.756208	4.466229	4.084154	4.450277	3.055244	2.135184	2.272661	1.950047	1.490297
Angola	0.114858	0.091078	0.103891	0.208546	0.385437	0.513967	0.497621	0.456654	0.460904	0.582235	0.779453	1.050562	1.324746	1.631367	1.866881	1.583571	1.509169	1.487532	0.955433	0.619465
Antigua and Barbuda	0.134605	0.148409	0.134085	0.374529	0.706744	0.514052	0.518829	0.380496	0.270496	0.296112	0.362067	0.450464	0.532228	0.559817	0.603401	0.691725	0.542369	0.554179	0.351945	0.269017
Argentina	0.089251	0.162697	0.228665	0.584307	1.198177	1.155322	1.100725	0.887915	0.712913	0.825456	1.207638	1.478503	1.840461	2.099734	2.572911	1.779524	2.096192	1.877361	1.351017	0.703124
Armenia	0.140309	0.243754	0.234842	0.676171	1.287575	0.712415	1.22413	0.563478	0.550748	0.668588	0.792706	0.806567	0.904749	0.79905	0.724736	0.871523	0.612642	0.58526	0.383281	0.280279
Australia	0.341863	0.460784	1.318504	3.296578	5.662448	6.857624	5.76255	4.823457	3.307175	2.960553	3.117906	3.026896	3.475463	3.859117	3.966114	3.391046	3.432269	3.824865	2.214295	1.503012
Austria	0.287571	0.315096	0.42874	1.755537	3.566312	4.37577	3.846409	3.314157	2.468719	2.383032	2.052616	2.465435	3.256713	2.989258	3.314685	2.355049	1.818585	2.21006	1.933323	1.060233
Azerbaijan	0.303125	0.649237	0.92183	1.842612	3.325677	2.550159	2.419312	1.810529	1.356814	1.307257	1.408756	1.556623	1.587825	1.809888	1.490746	1.067367	0.751947	1.474876	1.417362	1.190745
Bahrain	0.088274	0.177065	0.293416	0.557322	1.105464	0.938743	0.707593	0.607236	0.513378	0.479802	0.679806	0.983084	1.603497	1.679134	1.617744	1.029104	1.190589	0.965216	0.65366	0.572477
Bangladesh	0.099952	0.361858	0.38414	0.403111	0.523745	0.656667	0.485602	0.443967	0.397357	0.422863	0.55349	0.688772	0.798665	0.972421	1.045927	1.0373	1.250402	1.529136	0.647328	0.416454
Barbados	0.102768	0.120674	0.382741	0.845215	1.897443	1.515281	1.510747	1.114354	0.789276	0.927659	0.884084	0.93036	1.097954	1.188974	1.194711	1.368022	1.326313	1.641681	1.417754	1.63271
Belarus	0.38305	0.73169	1.187794	4.263045	8.63574	7.530414	7.546174	6.233708	4.460739	3.840093	3.371741	3.429169	4.062078	3.665843	3.72506	2.57735	1.75835	1.647293	1.136648	0.778033
Belgium	0.325612	0.388575	0.567793	2.772336	4.913159	6.41252	6.014946	4.561386	3.290685	3.592981	3.658176	3.550895	4.220176	3.833896	3.720274	2.838003	2.191071	2.423806	2.024656	1.636216
Belize	0.085422	0.080646	0.120328	0.419562	0.895389	0.992091	1.007616	0.779868	0.692531	0.717903	0.83513	1.067786	1.204267	1.374438	1.39569	1.450633	1.430511	1.481039	0.922775	0.975582
Benin	0.175517	0.078227	0.107375	0.205467	0.270861	0.343411	0.320201	0.321145	0.37855	0.409899	0.539833	0.816332	0.962133	1.258023	1.841686	1.430544	1.34805	1.355372	0.637221	0.45998
Bermuda	0.661263	0.531458	0.713499	3.326824	6.840622	4.504442	3.768326	2.761423	2.293062	2.264179	2.305908	2.272037	2.595905	2.46629	2.587931	3.270253	2.927201	3.809318	2.8635	3.485921
Bhutan	0.071206	0.272957	0.267459	0.413251	0.533958	0.63427	0.552865	0.470113	0.415079	0.453639	0.537475	0.773315	0.943438	1.326583	1.623147	1.607163	1.778059	1.789973	0.664958	0.378499
Bolivia	0.19895	0.274657	0.239327	0.35264	0.459031	0.475952	0.464107	0.454399	0.490454	0.630508	0.988499	1.390886	1.840274	2.462935	3.02846	3.454964	3.68327	3.361286	2.630258	1.398352
Bosnia and Herzegovina	0.120731	0.202157	0.451574	1.639346	2.802282	2.215734	2.06166	1.364132	1.208987	1.101906	1.146102	1.286621	1.419661	1.512271	1.81057	1.651029	1.74017	1.982474	1.333202	0.806397
Botswana	0.039964	0.081777	0.080521	0.1382	0.183834	0.284124	0.293336	0.270841	0.279728	0.359686	0.414728	0.585127	0.778002	0.834014	1.155507	0.814655	1.200649	1.281704	1.192059	0.387355
Brazil	0.083445	0.14988	0.269522	0.632891	1.059152	1.040134	0.926128	0.673512	0.527599	0.586747	0.700223	0.878205	1.02196	1.13325	1.371118	1.10854	1.104433	1.184713	1.041313	0.726906
Brunei	0.230314	0.251566	0.422668	2.004098	3.557282	2.731	2.085175	1.448971	1.189922	1.246494	1.750745	2.212807	2.878449	3.674095	4.09199	3.339283	2.629167	2.669005	2.133288	0.982599
Bulgaria	0.52724	0.987439	1.45586	3.634295	6.391271	6.383443	5.792887	4.27979	3.21796	3.641237	3.641278	3.529165	3.952144	3.366545	2.694629	1.728531	1.297469	1.393568	1.000491	0.902017
Burkina Faso	0.253956	0.076068	0.10697	0.246731	0.320136	0.418852	0.397765	0.397173	0.45844	0.463688	0.580729	0.828875	0.964197	1.282685	1.784932	1.420804	1.3129	1.331116	0.62667	0.466518
Burundi	0.406498	0.409505	0.376047	0.521321	0.689533	1.038397	0.97154	0.901865	0.873463	0.929498	1.093625	1.512081	1.790174	2.365197	2.76262	2.518635	2.609183	2.346713	0.833296	0.491618
Cambodia	0.080542	0.085569	0.136315	0.266866	0.40084	0.563496	0.43054	0.474827	0.527201	0.740037	1.084968	1.286889	1.614148	2.242846	2.729224	1.765958	1.678626	1.410404	0.980431	0.842655
Cameroon	0.142162	0.084911	0.12311	0.229126	0.281772	0.358788	0.344256	0.347936	0.403455	0.425786	0.554793	0.837284	1.00609	1.345445	1.913209	1.490159	1.415042	1.444197	0.67552	0.504394
Canada	0.364954	0.35032	1.043918	4.211792	8.715183	8.865219	7.873198	5.778408	3.859687	3.576029	3.574909	2.954801	3.366177	3.244782	3.457759	3.549574	2.791745	3.102917	2.399578	1.70435
Cape Verde	0.050728	0.023911	0.02883	0.063283	0.08741	0.118677	0.122505	0.133084	0.13743	0.138287	0.143443	0.210298	0.1952	0.252862	0.401453	0.332672	0.345952	0.403845	0.250348	0.213267
Central African Republic	0.240495	0.148906	0.159783	0.300609	0.541222	0.761176	0.789015	0.789559	0.85872	1.094122	1.42839	1.825712	2.163951	2.638046	2.871142	2.398622	2.20929	2.066778	1.310047	0.853795
Chad	0.21802	0.124693	0.153442	0.249859	0.308446	0.381844	0.363597	0.373218	0.436297	0.477731	0.630772	0.971651	1.154294	1.549854	2.268549	1.689926	1.575156	1.608563	0.673344	0.544384
Chile	0.149343	0.245491	0.477496	1.076336	1.94506	1.726377	1.378791	1.13813	0.902684	0.890726	1.061899	1.235066	1.668731	1.987236	2.41913	1.741544	1.81913	2.309232	1.665527	1.899959
China	0.896575	0.988207	0.92807	0.819108	1.144468	1.286128	1.474132	0.959553	0.767966	0.949703	1.232374	1.41493	2.411005	2.661397	2.342621	1.921223	1.492546	1.465667	1.11227	0.991886
Colombia	0.333636	0.414344	0.429908	0.649569	1.0757	1.012959	0.873091	0.729304	0.635912	0.71086	0.828002	0.949227	1.315308	1.459613	1.925416	1.7388	1.506238	1.745171	1.335231	1.207849
Comoros	0.259182	0.441986	0.442651	0.620178	0.873594	1.250469	1.109295	1.051054	0.904834	0.913134	1.053923	1.41387	1.634923	2.136623	2.509233	2.312583	2.403772	2.156281	0.743955	0.419285
Congo	0.096777	0.087146	0.105671	0.218254	0.404827	0.543039	0.52809	0.490668	0.516991	0.638031	0.845051	1.164675	1.439183	1.803137	2.106863	1.863991	1.809569	1.858841	1.190706	0.813789
Costa Rica	0.307525	0.605319	0.754043	2.328839	3.259633	3.641517	2.845196	2.554549	1.698814	2.022181	2.030305	2.247965	2.820457	3.14189	3.740021	4.041712	3.639639	4.356297	3.561328	2.263487
Cote d'Ivoire	0.23644	0.170323	0.210352	0.309275	0.394791	0.526834	0.503231	0.471401	0.487178	0.486219	0.607215	0.895918	1.004769	1.315805	1.792346	1.421199	1.278861	1.227189	0.484002	0.333759
Croatia	0.395443	0.582012	1.119412	3.515237	6.86605	6.430917	6.609111	4.664944	3.566646	2.772599	2.78089	2.736491	3.29808	3.456024	3.528341	1.771062	1.497246	1.673814	1.00494	1.147856
Cuba	0.285237	0.320812	0.514721	1.808587	3.838442	3.786982	3.32725	2.567655	2.115573	2.269194	2.268785	2.189183	2.572303	2.754166	2.929366	3.124555	2.820172	2.897612	2.118451	2.563063
Cyprus	0.159842	0.293108	0.415159	2.781879	8.489445	5.521256	4.208638	3.225004	1.996414	2.044296	1.891383	2.916154	2.671466	3.161375	4.012624	2.995656	2.004556	0.93422	0.641131	0.47299
Czech Republic	0.413198	0.581725	1.725842	5.219099	10.54081	9.652166	8.746562	6.582261	4.304377	3.806461	3.259172	2.724581	2.47813	2.065849	2.181457	1.920545	2.061537	2.11626	1.638905	1.72874
Democratic Republic of the Congo	0.154923	0.081243	0.096088	0.178411	0.348999	0.478377	0.484894	0.453814	0.472553	0.629341	0.845917	1.144328	1.421614	1.755982	2.001258	1.754623	1.68947	1.6886	1.155062	0.76714
Denmark	0.328435	0.517087	0.87533	3.008991	5.975237	6.692953	5.727883	4.019671	2.911807	3.037873	3.784931	3.997941	4.871856	3.762047	4.449711	3.481002	2.816582	3.30824	2.478033	1.98309
Djibouti	0.286456	0.454355	0.444744	0.515592	0.670882	0.995847	0.92757	0.892653	0.819171	0.866044	1.013962	1.43533	1.675841	2.13976	2.585637	2.246788	2.310163	2.049453	0.693179	0.388185
Dominica	0.233676	0.181136	0.388606	0.763989	1.673166	1.316476	1.319326	0.885477	1.079894	0.874577	0.981902	1.0515	1.320996	1.520855	1.746087	1.781111	1.555749	1.601511	1.093628	0.916173
Dominican Republic	0.115508	0.164596	0.129014	0.262878	0.42349	0.316734	0.371167	0.307939	0.383221	0.396099	0.553671	0.688464	0.868326	0.9193	0.933457	0.932699	0.950691	1.259591	0.588125	1.035465
Ecuador	0.670312	0.407378	0.401596	0.641408	0.785481	0.876011	0.783612	0.594887	0.596716	0.550849	0.83309	1.065482	1.330118	1.470915	1.82793	2.535128	2.964438	3.178643	4.192686	3.584608
Egypt	0.250523	0.70269	1.091805	1.819188	2.384667	2.47985	2.265582	1.946961	1.702674	1.682831	1.918743	2.2776	2.853991	3.923684	5.037565	4.450209	4.636206	4.255484	1.959352	1.256501
El Salvador	0.185207	0.222262	0.36096	0.642217	0.721349	0.706421	0.753309	0.74668	0.599481	0.765417	1.026877	1.394801	1.828553	2.025002	2.798591	2.652598	2.771874	2.992686	3.329845	2.923824
Equatorial Guinea	0.070495	0.066251	0.075406	0.151682	0.273104	0.327023	0.323154	0.285593	0.273285	0.345871	0.44624	0.612026	0.791365	0.979461	1.163711	1.057082	1.017528	1.025453	0.663452	0.43529
Eritrea	0.592993	0.639782	0.600548	0.89353	1.260381	2.019137	1.972192	1.979454	1.899875	1.83339	1.85971	2.29181	2.402378	3.655473	3.314749	2.977695	2.885317	2.349126	1.012941	0.450077
Estonia	0.363326	0.518324	0.792455	6.071551	9.664561	9.075003	8.211025	5.833763	4.319451	3.502312	3.388195	3.571764	4.107729	3.831959	3.14596	2.506843	2.513221	2.503616	2.444976	1.076077
Ethiopia	0.460142	0.660354	0.643027	0.863613	1.168974	1.748657	1.558669	1.429687	1.226558	1.307951	1.580894	2.243019	2.737965	3.730368	4.651609	4.116973	4.315899	3.996912	1.184873	0.758488
Federated States of Micronesia	0.030068	0.130605	0.065893	0.134745	0.324531	0.469581	0.549356	0.360414	0.321944	0.493788	0.629239	0.554854	0.763551	0.904176	1.255738	1.1681	0.946302	1.380522	1.593384	0.95707
Fiji	0.08046	0.283029	0.151477	0.181756	0.365312	0.439625	0.491049	0.395825	0.307599	0.450276	0.531221	0.364291	0.52493	0.599921	0.865759	0.93672	0.710277	1.281255	1.485844	0.58938
Finland	0.31786	0.489657	1.375569	3.999274	7.882721	7.659542	6.696441	5.586547	3.576308	3.676696	4.05245	3.998267	4.148035	4.282892	3.922763	3.475238	2.972538	3.53838	2.825618	1.421536
France	0.350774	0.350982	0.566256	2.267691	5.857574	7.391402	6.606191	4.612837	3.50851	3.631402	3.393032	3.516887	3.886847	3.31505	3.469654	2.429693	1.93495	2.353195	1.868796	1.619663
Gabon	0.08213	0.072869	0.088364	0.171088	0.282305	0.395576	0.415111	0.399221	0.411133	0.519457	0.701536	0.969703	1.20621	1.45941	1.615985	1.355845	1.32893	1.357678	0.912781	0.599582
Georgia	0.720278	0.427663	0.768426	1.240171	3.492985	2.690114	3.292118	2.584658	2.530497	2.874071	2.920412	3.511542	3.184181	3.400892	3.541439	3.281297	1.700021	2.929712	1.85965	0.989339
Germany	0.380544	0.400688	1.012093	3.386534	5.710756	5.888553	6.398421	5.073152	3.541602	3.450685	3.435355	3.788728	4.268899	3.909465	4.834425	3.482598	2.909988	3.204682	2.236848	1.522032
Ghana	0.065122	0.026748	0.045903	0.068066	0.076351	0.094611	0.093857	0.095438	0.140753	0.11914	0.163862	0.285325	0.308426	0.420521	0.811124	0.542713	0.521221	0.679373	0.672954	0.84687
Greece	0.541436	0.965008	1.467361	5.07374	11.30354	10.93736	10.70055	8.777884	6.458328	7.678007	8.786623	8.759807	11.15367	9.903956	10.48284	9.477403	7.311763	8.557264	8.86494	7.648683
Greenland	0.042246	0.053742	0.06301	0.180483	0.425779	0.374969	0.518065	0.501094	0.4495	0.382902	0.502413	0.845698	1.058131	1.40054	1.726943	1.360646	1.217942	0.796209	0.558308	0.459402
Grenada	0.047514	0.065165	0.098745	0.185227	0.340115	0.594865	0.50058	0.471625	0.399505	0.415285	0.634401	0.691087	0.79686	0.894324	0.837128	0.812871	0.687036	0.80855	0.62734	0.776823
Guam	0.140506	0.306728	0.155848	0.419731	0.507908	0.854751	0.957432	0.634784	0.455127	0.511503	0.762091	0.42171	0.545109	0.564464	0.806925	0.657489	0.434792	0.705488	0.826016	0.198664
Guatemala	0.367796	0.206925	0.168503	0.197394	0.275598	0.289804	0.327928	0.331452	0.351608	0.488819	0.679272	0.78394	1.035338	1.398121	1.871716	1.519118	1.685053	2.1096	2.128153	4.310762
Guinea	0.421335	0.231829	0.279115	0.412378	0.541898	0.735718	0.691859	0.637962	0.660523	0.666155	0.839168	1.242117	1.439482	1.917897	2.631835	2.098947	1.845227	1.671402	0.67293	0.469461
Guinea-Bissau	0.183877	0.128269	0.180483	0.322309	0.411221	0.530659	0.526298	0.521139	0.629998	0.629821	0.781378	1.131489	1.302246	1.749417	2.38639	1.761045	1.640427	1.646841	0.815688	0.659383
Guyana	0.051707	0.071944	0.096156	0.250337	0.612543	0.7408	0.681628	0.582375	0.551735	0.608496	0.727888	0.889405	1.007832	1.145627	1.196729	1.212046	1.085584	1.078669	0.715218	0.790066
Haiti	0.17264	0.142739	0.157437	0.358588	0.772441	0.877721	0.937884	0.913297	1.021689	1.196424	1.707302	2.244315	2.855681	3.676935	3.944142	4.131024	3.745917	4.067145	2.701815	2.283413
Honduras	0.047658	0.044261	0.032153	0.044016	0.072405	0.080519	0.091169	0.095516	0.103658	0.182121	0.310262	0.47451	0.721154	0.861262	0.980075	0.4489	0.363434	0.365574	0.252184	0.193406
Hungary	0.33888	0.467326	1.025995	2.570907	4.711322	4.033821	3.244006	2.376832	1.715815	1.731585	1.868658	1.577422	1.404905	1.322837	1.290112	1.148076	1.082426	1.190351	0.715583	0.481114
Iceland	0.339533	0.363146	1.008195	2.809304	4.987191	5.022034	5.582225	4.019625	2.888402	2.989989	3.136363	2.766643	3.308486	3.39344	3.753308	2.846144	2.002011	2.082354	1.556135	1.110033
India	0.058179	0.184292	0.192571	0.280228	0.438019	0.581191	0.624654	0.568808	0.525837	0.54115	0.648387	0.934354	0.968643	1.24185	1.544198	1.428911	1.723116	1.784696	0.708048	0.390476
Indonesia	0.089166	0.102122	0.167594	0.312965	0.41157	0.489105	0.347174	0.355186	0.379908	0.513603	0.748201	0.918209	1.222712	1.719452	2.15282	1.388588	1.405389	1.259262	0.895083	0.777493
Iran	0.375084	0.686664	1.052436	1.835062	2.430337	1.926079	1.689388	1.342581	1.013153	1.036591	1.21511	1.194549	1.308975	1.507531	1.757473	1.716561	1.950506	2.108324	1.843016	2.073169
Iraq	0.068709	0.155016	0.146673	0.205584	0.271636	0.330141	0.336261	0.289313	0.239295	0.294718	0.365467	0.397735	0.519102	0.583835	0.686988	0.569531	0.650401	0.895086	0.763887	0.975563
Ireland	0.325184	0.385726	1.443922	3.642532	7.30017	8.656657	7.043966	5.50406	3.934643	4.072845	4.429445	4.199659	4.566786	4.333787	4.654645	3.674986	2.804776	2.971792	2.310876	1.723356
Israel	0.236755	0.399386	0.640619	2.269498	4.219212	5.099966	4.568748	3.477097	2.344218	2.027902	2.255199	2.283893	2.626714	2.465662	2.692735	2.495466	2.182322	2.425388	2.140016	1.969169
Italy	0.349452	0.64483	1.2272	4.040414	9.550754	9.022949	7.74323	5.296036	3.088669	4.403603	4.760168	4.194951	4.81079	3.787695	4.499024	2.664153	2.413192	2.596227	2.069521	1.606546
Jamaica	0.085331	0.054158	0.102979	0.29957	0.979262	0.700891	0.794247	0.706948	0.479427	0.350592	0.509261	0.668698	0.880424	0.938233	0.849385	0.931928	0.8007	0.802742	0.391702	0.289961
Japan	0.31897	0.321394	0.404668	0.611794	1.291921	1.256968	1.29323	1.079668	0.976999	1.014859	1.200946	1.170475	1.474715	1.623156	1.612367	1.266248	1.11346	1.166083	0.88847	0.735085
Jordan	0.081107	0.08434	0.091088	0.201446	0.41971	0.366228	0.386034	0.277008	0.219988	0.238523	0.245676	0.274306	0.348608	0.462894	0.498206	0.542876	0.520254	0.733127	0.498135	0.322902
Kazakhstan	0.247852	0.360944	0.557435	1.324642	2.749068	2.284511	2.65495	2.18841	1.448112	1.314134	1.233899	1.402764	1.37984	1.354245	1.169024	1.219513	0.786834	0.772633	0.539771	1.479392
Kenya	0.128043	0.230831	0.246861	0.377346	0.528499	0.75703	0.721836	0.661179	0.584231	0.593088	0.685651	0.930879	1.05913	1.375491	1.6852	1.423113	1.408105	1.248038	0.435417	0.247587

Kiribati	0.030311	0.117139	0.051947	0.084801	0.185063	0.300754	0.358259	0.277863	0.217945	0.291563	0.358985	0.347949	0.470253	0.482071	0.875359	0.713816	0.629319	0.839966	0.940311	0.645374
Kuwait	0.184783	0.3505	0.404154	0.75847	0.821983	1.024852	0.907869	0.61512	0.638944	0.672939	0.745681	0.697109	1.005827	0.901337	0.648189	0.71831	0.438709	1.373179	2.28455	0.404547
Kyrgyzstan	0.088646	0.197549	0.259782	0.341419	0.4896	0.429973	0.495217	0.545972	0.466001	0.43961	0.430264	0.934415	0.761584	0.928918	0.824045	0.973104	0.500078	0.373092	0.218249	0.18219
Laos	0.1724	0.162516	0.215504	0.326275	0.46063	0.652794	0.499807	0.510533	0.526288	0.711166	1.044497	1.263605	1.637531	2.216105	2.80872	1.654708	1.635084	1.402448	0.932823	0.809373
Latvia	0.295697	0.408758	0.906487	3.474341	8.827978	8.779446	8.452795	6.099358	4.03227	3.634103	3.184905	3.243144	4.237381	3.251808	3.195865	2.503995	1.486355	1.273502	0.973459	0.409343
Lebanon	0.736178	3.420157	6.470656	19.28804	26.34561	20.70788	16.49582	10.96622	7.491761	6.527016	6.212777	5.83782	6.448213	6.471679	6.768314	6.027859	5.360966	5.192834	2.93882	2.189111
Lesotho	0.043178	0.142455	0.132939	0.233054	0.301535	0.564184	0.675802	0.77749	0.835979	1.048931	1.108028	1.551806	1.865282	1.813219	2.221623	1.463322	2.08948	2.128418	1.965966	0.662295
Liberia	0.123706	0.067907	0.101958	0.17259	0.234976	0.304795	0.290331	0.290211	0.328749	0.349436	0.475097	0.708445	0.840643	1.104661	1.785162	1.268039	1.252674	1.402219	0.569483	0.498312
Libya	0.5888	1.517662	2.501495	5.258034	8.559793	7.435164	6.986481	4.705752	3.645004	3.359366	3.119109	3.345992	4.388256	4.909107	5.667734	5.001008	5.497527	5.242686	3.31015	3.260717
Lithuania	0.365664	0.537488	1.413237	3.223356	6.493688	7.097272	6.634288	4.961496	3.353624	2.892673	2.709524	2.748792	3.615329	3.249087	3.058314	2.432056	1.789295	1.726856	1.449739	1.155043
Luxembourg	0.294247	0.324681	0.714709	3.259617	6.445317	6.322908	5.589848	5.743386	3.060622	3.412388	3.869162	3.43634	4.136341	3.911778	4.18345	3.205045	2.267719	2.513438	2.371249	1.987121
Macedonia	0.232049	0.58846	0.664112	2.45169	5.610376	5.017979	4.010825	2.317771	2.037583	1.760712	1.928649	1.539224	1.667809	1.385142	1.726947	1.38769	0.934872	0.925375	0.320494	0.095018
Madagascar	0.286001	0.364021	0.367078	0.510757	0.72273	1.144597	1.048045	0.987506	0.888063	0.911283	1.068351	1.460672	1.595974	1.999685	2.336571	2.012493	2.038828	1.823792	0.630974	0.413024
Malawi	0.256305	0.265496	0.254434	0.337566	0.472383	0.682448	0.613084	0.569699	0.509496	0.536402	0.589989	0.794855	0.903742	1.176746	1.447491	1.278819	1.245606	1.072884	0.352897	0.216885
Malaysia	0.093181	0.2314	0.451963	1.057552	1.43474	1.313413	0.82557	0.688547	0.543754	0.617104	0.791342	0.809488	1.042722	1.292636	1.703066	1.172082	1.125116	0.84975	0.617233	0.604006
Maldives	0.168063	0.125304	0.135473	0.165143	0.294221	0.298407	0.230875	0.237413	0.188416	0.208789	0.247883	0.278589	0.413572	0.51194	0.649322	0.325505	0.317562	0.369674	0.302787	0.286882
Mali	0.748355	0.310481	0.299129	0.362555	0.459914	0.625798	0.588739	0.558876	0.553637	0.55065	0.678806	0.975934	1.112968	1.488663	2.114647	1.7355	1.628256	1.669333	0.592458	0.457971
Malta	0.334619	0.623331	1.398066	3.565696	9.057558	9.092246	9.263032	6.357885	4.108312	3.838734	3.855145	4.104054	4.965652	4.726047	5.447822	4.442865	3.454765	3.65097	2.77836	1.7582
Marshall Islands	0.029664	0.121311	0.08258	0.166856	0.414957	0.558075	0.569445	0.452864	0.435249	0.580962	0.678824	0.690122	0.965466	1.220387	1.382721	1.330057	0.971333	1.468937	1.508604	0.781008
Mauritania	0.10135	0.097822	0.128076	0.204767	0.235854	0.28076	0.27157	0.254642	0.294701	0.300304	0.40245	0.596859	0.737855	1.044075	1.541227	1.189788	1.149225	1.240039	0.551782	0.427057
Mauritius	0.216801	0.289379	0.558753	1.186022	1.635339	2.247752	0.74558	0.585187	0.672793	0.673004	0.923438	0.601456	0.891272	1.138978	1.337428	0.646774	0.526386	0.447874	0.376908	0.632373
Mexico	0.392063	0.366381	0.395251	0.664858	1.151547	1.005558	0.915691	0.822135	0.741653	0.926672	1.102949	1.393276	1.766572	2.054185	2.600215	2.884911	3.020134	3.238593	2.555888	2.416916
Moldova	0.507108	0.747465	1.169544	2.532062	4.356124	3.96926	3.172196	2.532151	2.037924	2.132087	2.046932	2.193045	2.842073	3.03817	2.668063	1.588925	1.295923	1.007557	0.576055	0.948655
Mongolia	0.034363	0.051145	0.071717	0.117488	0.211164	0.217502	0.257795	0.269294	0.236043	0.325056	0.372408	0.439836	0.552697	0.685183	0.731357	0.457449	0.273037	0.329573	0.268275	0.252838
Montenegro	0.59051	1.451214	2.63467	5.071463	12.22955	10.36862	8.154585	5.447784	3.808574	3.583172	3.429751	3.244321	3.079388	2.961479	3.356963	2.780341	2.474071	2.663965	1.566369	1.253702
Morocco	0.219656	0.964003	1.202175	1.94332	2.621188	2.660181	2.567353	1.875921	1.446585	1.402557	1.542612	1.887923	2.261701	3.121771	3.970798	3.712116	4.126263	3.940733	2.305677	1.833144
Mozambique	0.456659	0.587149	0.526544	0.746588	0.967247	1.467788	1.37716	1.334226	1.246185	1.243642	1.363379	1.769443	1.949479	2.460839	2.936502	2.699075	2.751306	2.461965	0.837023	0.464408
Myanmar	0.161202	0.140937	0.203044	0.362164	0.522536	0.736394	0.552605	0.567469	0.586829	0.785328	1.135516	1.309497	1.678164	2.281062	2.750054	1.835139	1.764145	1.46139	0.991415	0.832884
Namibia	0.076072	0.191887	0.217495	0.394117	0.43494	0.647181	0.643928	0.614379	0.596752	0.711114	0.80761	1.12735	1.409986	1.541682	2.106721	1.537708	1.907786	1.777794	1.287908	0.41155
Nepal	0.053363	0.196523	0.208431	0.352297	0.47081	0.568643	0.513225	0.463928	0.449056	0.519257	0.642079	0.96727	1.148526	1.563584	1.928748	1.93481	2.119236	1.999641	0.742286	0.372546
Netherlands	0.33687	0.306986	0.685113	3.310886	7.004234	7.273546	7.882673	6.36638	3.581755	3.830723	4.14427	4.194616	5.101336	4.879287	5.129281	3.985147	3.133954	3.889573	4.426765	3.930358
New Zealand	0.308402	0.73219	0.900448	2.693831	5.142823	7.676865	5.470027	3.766238	3.069512	2.865288	2.528155	2.646061	3.273684	3.193321	3.513402	3.339591	3.311177	3.582678	2.189512	1.884015
Nicaragua	0.09803	0.154505	0.173881	0.263753	0.452114	0.362842	0.318505	0.254572	0.290072	0.43064	0.458952	0.628457	0.92905	0.965868	1.161926	1.289547	1.40085	1.709287	0.856817	1.067637
Niger	0.210552	0.08163	0.108524	0.205425	0.266175	0.347203	0.334	0.343527	0.379871	0.399611	0.532783	0.83619	0.985613	1.296957	1.833067	1.44324	1.391832	1.446337	0.636186	0.502764
Nigeria	1.731492	1.161562	1.192942	1.42397	1.943205	2.522479	2.056662	1.70803	1.702784	1.5742	1.989057	2.789183	3.409875	4.721574	7.0397	6.212835	6.152479	6.416994	2.892498	1.793206
North Korea	0.311105	0.197725	0.163752	0.348283	0.547843	0.442398	0.458723	0.362627	0.36023	0.45441	0.618124	0.874154	1.194079	1.304326	1.272104	1.118218	0.919746	0.836196	0.603755	0.603258
Northern Mariana Islands	0.171318	0.200196	0.142871	0.270302	0.630685	0.51681	0.532565	0.396187	0.234196	0.400156	0.318371	0.359785	0.434726	0.56147	0.698752	0.65799	0.394473	0.610368	0.57543	0.244247
Norway	0.267666	0.311994	1.105709	3.278251	5.188719	5.810532	5.728314	4.587808	2.849634	3.00236	3.301305	3.012828	3.658022	3.831342	4.078737	3.160985	2.666805	3.021409	2.31705	1.581
Oman	0.299587	0.764601	1.17131	2.148756	3.1207	2.316662	2.058588	1.465064	1.243969	1.36419	1.472091	1.717767	1.953296	2.137487	2.305213	1.989731	1.996857	1.887657	0.955909	0.600249
Pakistan	0.352954	1.393045	1.369058	1.838258	2.504753	3.022013	2.098121	1.905892	1.453245	1.385102	1.786277	2.200882	2.958014	3.414582	4.336985	3.782777	4.677401	5.140395	2.011011	1.42027
Palestine	0.066177	0.118852	0.178713	0.359302	0.611003	0.956938	0.776059	0.611926	0.566387	0.835535	0.870562	0.923584	1.124848	1.447778	1.773025	1.726831	1.489042	1.580524	1.267991	0.461172
Panama	0.463894	0.465554	0.370664	0.626737	0.56383	0.732206	0.748636	0.501971	0.366857	0.439389	0.605897	0.696455	0.881081	1.037206	1.231892	1.314547	1.222807	1.452956	0.560592	0.387533
Papua New Guinea	0.045376	0.202767	0.098627	0.182455	0.45546	0.700032	0.955946	0.715015	0.636295	0.826542	1.03418	0.83436	1.072847	1.304829	1.614893	1.47754	0.971015	1.300697	1.255297	0.627614
Paraguay	0.031443	0.173328	0.258493	0.426015	0.511799	0.536885	0.642373	0.469775	0.450731	0.616714	0.771404	1.054267	1.253033	1.577089	1.896425	1.421873	1.330234	1.385998	1.102338	0.40452
Peru	0.096223	0.184948	0.177721	0.239349	0.341146	0.311631	0.312014	0.284086	0.265588	0.368443	0.513489	0.607197	0.770534	0.961354	1.138574	1.25212	1.266858	1.182599	1.071101	0.609935
Philippines	0.094405	0.079767	0.129521	0.191337	0.245864	0.342991	0.218312	0.213935	0.207156	0.297413	0.385651	0.425279	0.591177	0.799923	1.01617	0.599956	0.645596	0.711726	0.70007	0.695843
Poland	0.367614	0.730253	1.1749	3.510237	7.228162	6.428201	4.756004	3.578377	2.805282	2.100862	2.20072	1.94573	1.992881	1.754402	1.917917	1.429743	1.234077	1.15231	0.845114	0.611232
Portugal	0.399894	0.267378	0.57752	1.988667	4.658062	4.489266	4.661953	3.812688	2.494598	2.457734	2.640849	2.359794	2.726544	2.468241	2.581752	2.122619	1.451502	2.037271	1.700204	1.315884
Puerto Rico	0.278572	0.219315	0.537747	2.682537	7.300437	4.561016	5.141119	3.655039	2.474967	2.092048	2.192605	2.068916	2.366531	1.993227	1.973838	2.147574	1.774175	2.055494	1.008163	0.423003
Qatar	0.160021	0.438929	0.319074	0.761136	2.377892	0.904658	0.663701	0.434352	0.453217	0.42848	0.682663	0.880436	1.030063	0.954656	0.905307	0.617444	0.546442	0.621996	0.515384	0.521182
Romania	0.468257	0.829283	1.303475	2.806945	5.207827	4.551663	4.041766	2.820602	2.120774	2.004899	1.964275	1.775404	1.53312	1.423237	1.337451	1.139522	0.913192	0.84973	0.541684	0.314332
Russian Federation	0.406577	0.721852	1.566367	3.974335	8.526457	8.762563	7.767468	6.075618	4.347221	2.971293	2.554004	2.789049	2.879888	2.770295	2.682166	2.068087	1.496346	1.206183	0.87517	0.590866
Rwanda	0.357925	0.371038	0.363947	0.476757	0.622893	0.894573	0.813735	0.759758	0.667833	0.706868	0.846562	1.181859	1.39199	1.819216	2.236883	2.069854	2.145131	1.936588	0.692581	0.369095
Saint Lucia	0.091781	0.136756	0.226069	0.513119	1.209359	1.30875	1.350979	0.959067	0.95985	0.923113	1.132245	1.277663	1.329905	1.493165	1.596745	1.672372	1.387479	1.578457	1.281168	1.581285
Saint Vincent and the Grenadines	0.081187	0.141196	0.262026	0.567142	1.085064	1.10644	1.411336	0.986208	0.973621	1.019941	1.142436	1.194925	1.474292	1.800764	1.910118	2.037886	1.492032	1.34159	0.941941	1.088999
Samoa	0.046415	0.174436	0.152244	0.340853	0.606624	0.623498	0.662657	0.439504	0.389522	0.541629	0.677187	0.703263	0.974836	1.231351	1.630781	1.494941	1.13764	1.464194	1.300779	0.692083
Sao Tome and Principe	0.043839	0.030122	0.02463	0.046743	0.065167	0.072806	0.062124	0.06088	0.064409	0.076164	0.110732	0.168266	0.204044	0.261561	0.340233	0.252751	0.241691	0.32297	0.343054	0.694723
Saudi Arabia	0.299269	0.590431	1.017878	3.323752	4.337555	3.571792	2.561819	2.033392	1.776479	1.845862	1.936397	1.94484	2.023783	2.183395	2.643672	2.581011	2.574106	2.61607	1.24014	0.746361
Senegal	0.144304	0.091862	0.127047	0.22554	0.264518	0.329883	0.314818	0.3246	0.401247	0.420645	0.581256	0.915086	1.086898	1.506291	2.11226	1.619877	1.547042	1.564271	0.680655	0.526006
Serbia	0.200842	0.386757	0.718929	2.220855	5.246057	6.49394	5.4692	3.618571	3.059022	3.195899	3.40764	3.766747	3.066751	2.094377	2.271612	2.031471	1.71929	1.723647	1.102891	0.57377
Seychelles	0.281533	0.285486	0.352394	0.851402	1.381152	1.40337	1.015316	0.841912	0.785532	0.964897	1.208365	1.239239	1.628419	2.083063	2.298474	2.054149	2.014849	1.570165	1.633059	1.760659
Sierra Leone	0.197703	0.083216	0.116288	0.217206	0.285912	0.363732	0.343911	0.337224	0.384102	0.393253	0.496023	0.743708	0.872856	1.192298	1.770413	1.398198	1.370086	1.431683	0.636428	0.525434
Singapore	0.183747	0.29548	0.312986	0.943741	2.083029	1.757312	1.302298	0.900608	0.825433	0.838101	1.01249	1.183906	1.481127	1.587055	1.73153	1.299067	1.048885	1.052301	0.871145	0.626734
Slovakia	0.470407	0.69856	1.183389	4.387265	10.59119	10.17265	9.239769	5.274288	3.914851	3.223974	3.039913	2.600925	2.179945	1.871109	1.923377	1.648541	1.633733	1.783743	1.105264	0.626096
Slovenia	0.32817	0.454466	2.449601	5.13469	8.000628	5.985889	5.768902	4.845902	3.276628	3.017109	2.862614	2.069282	1.78073	1.42164	1.495968	1.411838	1.59485	1.869484	1.394812	0.864729
Solomon Islands	0.02812	0.124332	0.070585	0.140978	0.328265	0.46884	0.610906	0.462455	0.442361	0.606378	0.777026	0.677693	0.898755	1.053818	1.449459	1.390117	1.013886	1.480172	1.488149	0.642417
Somalia	0.621427	0.665235	0.643648	0.814211	1.137531	1.788801	1.665678	1.583836	1.499756	1.538188	1.788647	2.384565	2.674666	3.443507	4.077258	3.531673	3.45356	3.135223	1.066899	0.708659
South Africa	0.056105	0.059292	0.040321	0.104394	0.200424	0.339553	0.384067	0.361769	0.347852	0.431552	0.495739	0.706364	0.844218	0.805829	0.951014	0.596313	0.894723	0.990521	1.015925	0.340852
South Korea	0.316935	0.310053	0.312096	0.57739	1.085469	0.944709	0.920984	0.659662	0.664096	0.643517	0.788778	0.840412	1.070786	1.237958	1.391617	1.188514	0.986936	1.09286	0.985905	0.537828
South Sudan	0.550059	0.503742	0.464248	0.577536	0.789323	1.223903	1.17231	1.155837	1.070423	1.101777	1.294553	1.820181	2.138862	2.499612	3.121024	2.72146	2.763301	2.569862	0.763632	0.480664
Spain	0.346708	0.448252	0.945541	3.048637	5.917991	6.56708	6.664805	5.029338	3.221313	3.440451	3.880447	3.525085	4.125365	3.488347	3.74618	2.883049	2.655669	2.827045	2.356325	1.940518
Sri Lanka	0.254977	0.271703	0.458328	0.901472	1.309845	1.230665	0.909892	0.84386	0.831626	1.006668	1.247678	1.25155	1.58942	1.875699	2.634947	1.580096	1.619303	1.586787	1.244995	1.107676
Sudan	0.16322	0.273018	0.311883	0.443662	0.65972	0.731232	0.698727	0.606899	0.56149	0.649913	0.869005	1.204628	1.43174	2.075823	2.644927	2.329417	2.58757	2.641398	1.613619	1.290503
Suriname	0.111269	0.099124	0.195079	0.493378	0.838853	1.082761	1.046462	0.907584	0.80306	0.805984	1.057329	1.258038	1.420708	1.775502	1.755023	1.973814	1.81287	1.941131	1.191349	1.18435
Swaziland	0.045162	0.11502	0.110855	0.202543	0.258416	0.471659	0.561215	0.641403	0.717026	0.902917	0.920159	1.276528	1.492131	1.475161	1.754499	1.109531	1.585491	1.590736	1.478719	0.521261
Sweden	0.347674	0.410998	0.616408	1.893797	4.261607	4.701831	4.180709	3.237683	1.997792	2.080697	2.157741	2.186256	2.819507	2.9001	3.062275	2.849839	2.640641	3.034887	2.296956	1.529854
Switzerland	0.327363	0.386579	0.875702	2.67549	4.908696	4.861025	5.04538	3.481779	2.431125	2.903728	2.783076	2.70477	3.25432	3.147975	3.845679	2.824181	2.26914	2.431678	1.821599	0.920467
Syria	0.056559	0.046139	0.057994	0.112545	0.181795	0.19987	0.169577	0.144893	0.146956	0.160823	0.215979	0.287154	0.326707	0.414211	0.490735	0.415686	0.353778	0.403903	0.24999	0.167515
Tajikistan	0.054033	0.052886	0.055788	0.119346	0.252471	0.230215	0.292398	0.36946	0.323642	0.292693	0.492829	0.652967	0.766644	0.865318	1.15299	0.694037	0.503548	1.952123	1.022531	0.232896
Tanzania	0.511539	0.473672	0.449863	0.53549	0.707118	1.046138	0.940634	0.856801	0.71647	0.751802	0.897416	1.246694	1.454988	1.896393	2.333615	2.121855	2.15388	0.515499	0.375535	0.370785
Thailand	0.343401	0.240794	0.287998	0.565123	0.796393	0.834631	0.626294	0.501647	0.396563	0.515082	0.615538	0.629871	0.775702	0.957859	1.240505	0.61548	0.556415	1.264368	0.652321	0.251775
The Bahamas	0.106132	0.113227	0.16436	0.494877	1.301959	1.498099	1.429272	1.058066	0.783775	0.755049	0.812021	0.880186	0.919788	1.133862	1.15102	1.252323	1.149983	1.178283	0.338649	0.924346
The Gambia	0.197525	0.15154	0.180811	0.272513	0.342077	0.455783	0.447371	0.428512	0.46303	0.471924	0.604502	0.888434	1.002756	1.39239	1.922187	1.46234	1.3029	1.622914	0.838292	0.324152
Timor-Leste	0.123011	0.143691	0.206037	0.340755	0.449079	0.531838	0.38868	0.415851	0.458818	0.637537	1.004232	1.232075	1.626851	2.11316	2.552242	1.727683	1.733654	1.252212	0.485556	0.874466
Togo	0.110723	0.063408	0.098517	0.212028	0.271661	0.345286	0.329242	0.331158	0.407428	0.42714	0.553535	0.814888	0.916369	1.221318	1.57032	1.264447	1.279551	0.970709	0.99227	0.435032
Tonga	0.043101	0.065063	0.048057	0.139476	0.2895	0.316724	0.336334	0.263057	0.205426	0.312058	0.404511	0.366959	0.493929	0.631611	0.871393	0.81526	0.644828	0.500934	0.608679	0.608369
Trinidad and Tobago	0.094487	0.071891	0.132653	0.501842	1.125184	1.127225	0.905291	0.822413	0.520907	0.725473	0.807934	0.730282	0.673462	0.757942	0.726679	1.359614	0.992746	3.747744	1.112593	0.186648
Tunisia	0.292143	0.816331	1.308693	2.312935	3.355919	3.278978	3.004877	2.107033	1.563681	1.430131	1.505968	1.7929	2.097171	2.628555	3.049635	2.789065	3.407803	0.971429	0.262456	1.438158
Turkey	0.29241	0.471712	0.70213	1.279183	2.16594	1.795279	1.465002	0.95885	0.822546	0.86818	0.956587	1.030155	1.229374	1.431882	1.708108	1.365238	1.10634	0.689707	2.01627	0.326698
Turkmenistan	0.271041	0.216268	0.499021	1.080741	2.452691	2.189032	1.785384	1.577903	1.100671	0.99836	0.846154	1.175189	1.097092	1.26104	0.981214	0.859265	0.542121	2.379164	0.485028	0.438976
Uganda	0.433561	0.458928	0.435633	0.608405	0.819583	1.196784	1.099133	0.987056	0.876283	0.925422	1.062767	1.478046	1.739447	2.233989	2.767274	2.634786	2.722087	0.922983	0.502454	0.452171
Ukraine	0.472327	0.662919	1.406922	5.786817	12.15526	10.00545	8.839858	5.792775	4.467062	3.083104	2.407832	2.472272	2.613207	2.610576	3.169143	2.034066	1.051766	1.601638	0.867565	0.437764

United Arab Emirates	0.252594	0.36371	0.69253	1.707756	1.806434	1.456126	1.18676	0.972012	0.923967	1.001534	1.236021	1.547993	2.110493	2.269002	2.012174	1.38758	1.449268	3.187109	0.64542	0.926507
United Kingdom	0.297869	0.294958	0.695485	3.088356	6.762679	7.98301	7.014333	6.855071	4.771069	4.613893	4.717958	4.674536	5.696952	5.87495	5.604465	4.078442	2.856479	4.097229	1.041205	2.113621
United States	0.298296	0.281554	0.499986	1.880868	5.693277	6.983414	6.690629	4.963163	3.395664	3.11976	3.112239	2.738534	3.086984	3.096387	3.699727	4.07194	3.731751	2.025453	2.614719	2.235845
Uruguay	0.080888	0.146776	0.285863	0.910884	1.998174	1.83106	1.859708	1.229435	1.155448	1.141483	1.515125	1.75001	2.406081	2.706326	2.954585	1.598792	1.675075	1.004545	3.216185	0.308371
Uzbekistan	0.409013	0.698806	0.728547	0.921881	1.514458	1.287785	1.08423	1.045623	1.008903	1.044288	1.204108	1.434643	1.508382	1.563515	1.520721	1.334668	0.871579	2.122144	0.853421	0.634036
Vanuatu	0.031647	0.189214	0.092744	0.200635	0.548805	0.752203	1.028127	0.776794	0.739487	0.989775	1.303254	1.123593	1.502117	1.801483	2.330686	2.220315	1.516338	2.121983	0.736751	1.052357
Venezuela	0.220538	0.298057	0.406827	1.196345	1.814192	1.755724	1.370315	1.009073	0.823021	1.073471	1.295085	1.500741	2.018801	1.89645	2.368771	2.179772	2.357427	1.040789	2.11145	1.794737
Vietnam	0.131424	0.165525	0.308654	0.747274	0.966874	0.888295	0.5884	0.536286	0.524332	0.665682	0.859627	0.96505	1.143483	1.451777	1.869691	1.215584	1.21679	0.899501	2.053376	0.398193
Virgin Islands, U.S.	0.230362	0.188922	0.156515	0.419566	0.805095	0.684505	0.760396	0.711943	0.713747	0.828464	0.95257	0.940618	1.061123	1.113039	1.136858	1.107267	0.877253	2.882253	0.577158	0.602696
Yemen	0.081172	0.2444	0.30197	0.483844	0.732574	0.890224	0.866602	0.772787	0.756467	0.858021	1.113435	1.469523	1.716904	2.639323	2.974686	2.746843	3.002455	2.395174	0.583045	1.306409
Zambia	0.450652	0.4659	0.445157	0.596094	0.832556	1.225372	1.133795	1.066286	0.995671	1.017484	1.171056	1.561327	1.809097	2.350715	2.799735	2.577977	2.632053	1.424998	1.690321	0.496888
Zimbabwe	0.07508	0.246761	0.258341	0.415206	0.477377	0.778905	0.816276	0.874431	0.797536	0.912154	0.955202	1.195095	1.481816	1.576447	2.108393	1.458822	1.599566	1.053134	0.826209	0.388337

Supplementary Table S7. Age distribution of death rate (per 100,000) for Hodgkin lymphoma in different countries in 2017.

country	Under 5	5 to 9	10 to 14	15 to 19	20 to 24	25 to 29	30 to 34	35 to 39	40 to 44	45 to 49	50 to 54	55 to 59	60 to 64	65 to 69	70 to 74	75 to 79	80 to 84	85 to 89	90 to 94	95 plus
Afghanistan	0.116471	0.220447	0.36696	0.744221	1.084239	1.266462	1.34784	1.327098	1.445053	1.615964	2.251877	3.136335	3.594737	4.677458	6.115	5.648183	6.884853	7.026104	4.662678	4.46006
Albania	0.039544	0.074727	0.088878	0.190859	0.315831	0.446844	0.418543	0.366632	0.445744	0.455388	0.588934	0.892326	1.06348	1.406621	1.804184	1.575314	1.518713	2.371503	1.701657	1.24564
Algeria	0.04242	0.227427	0.370458	0.57806	0.675118	0.734212	0.774127	0.738908	0.696715	0.720844	0.885278	1.168329	1.516054	2.326201	2.984796	3.158709	4.057117	4.314838	2.423977	1.792139
American Samoa	0.00736	0.013016	0.011073	0.030045	0.088313	0.099196	0.146277	0.085869	0.099984	0.158239	0.190851	0.233045	0.348509	0.400547	0.687282	0.640145	0.658251	1.092839	1.429594	1.252357
Andorra	0.009098	0.010797	0.019817	0.089886	0.18766	0.250408	0.239635	0.250941	0.244104	0.300282	0.381797	0.56	0.770727	1.084644	1.520074	1.327143	1.675579	1.746616	1.484307	1.13418
Angola	0.061977	0.059436	0.079695	0.167347	0.264594	0.311105	0.335592	0.347748	0.393281	0.511496	0.708871	0.990808	1.302844	1.701745	2.049115	1.845072	1.997541	1.967291	1.262556	0.818303
Antigua and Barbuda	0.017541	0.022773	0.024143	0.069065	0.110818	0.101128	0.120079	0.113716	0.103187	0.130346	0.19273	0.296442	0.385011	0.497917	0.5879	0.737076	0.689257	0.704151	0.447539	0.341763
Argentina	0.016007	0.034305	0.055993	0.146553	0.258591	0.285407	0.316351	0.323572	0.331371	0.418244	0.69447	0.990923	1.309137	1.780759	2.391024	1.798548	2.583075	2.311795	1.661656	0.86654
Armenia	0.010809	0.021337	0.027651	0.070864	0.126027	0.103234	0.198034	0.122945	0.171152	0.258782	0.38885	0.520059	0.639082	0.728747	0.734808	0.955682	0.795832	0.757892	0.494916	0.360428
Australia	0.009805	0.012972	0.027119	0.074853	0.12922	0.204593	0.186113	0.189398	0.201842	0.219208	0.259277	0.39279	0.535237	0.827686	1.21876	1.341811	1.818741	2.025525	1.173441	0.795143
Austria	0.00895	0.010387	0.013101	0.057484	0.109474	0.172079	0.184151	0.181872	0.198751	0.234986	0.324899	0.490106	0.667485	0.925214	1.305434	1.179807	1.618889	1.995488	1.767306	0.987163
Azerbaijan	0.039406	0.101161	0.171162	0.33831	0.510751	0.503992	0.557886	0.55422	0.550557	0.64162	0.825563	1.158247	1.311108	1.773203	1.567888	1.210836	0.988685	1.943794	1.868846	1.56895
Bahrain	0.009012	0.022101	0.043075	0.080882	0.141918	0.162298	0.140194	0.155403	0.173191	0.186229	0.324081	0.609939	1.109955	1.454555	1.532941	1.073014	1.507962	1.222137	0.827119	0.719838
Bangladesh	0.041553	0.178257	0.229491	0.253669	0.274262	0.336189	0.282855	0.297692	0.306584	0.341686	0.4728	0.624508	0.761673	0.999706	1.135978	1.202316	1.652827	2.024358	0.856906	0.551964
Barbados	0.013226	0.016738	0.060844	0.140643	0.262562	0.277746	0.32197	0.297914	0.281472	0.380205	0.438024	0.574449	0.741942	1.016656	1.126705	1.414463	1.66606	2.0597	1.786098	2.052082
Belarus	0.014826	0.033105	0.05504	0.199758	0.361827	0.449725	0.525486	0.601138	0.578575	0.653523	0.763881	1.018392	1.241949	1.540713	1.763031	1.409676	1.465159	1.350947	0.887601	0.623972
Belgium	0.009682	0.011911	0.016388	0.083554	0.135822	0.222138	0.22575	0.229433	0.246849	0.310048	0.408808	0.589966	0.799437	1.137444	1.400462	1.356239	1.855005	2.015192	1.659574	1.306223
Belize	0.029098	0.030783	0.0528	0.184904	0.340846	0.402011	0.465276	0.427275	0.45859	0.513097	0.648008	0.905617	1.086285	1.371829	1.481711	1.649906	1.872668	1.939442	1.209225	1.277548
Benin	0.096348	0.049953	0.080522	0.164812	0.18474	0.215381	0.225158	0.250631	0.324402	0.359279	0.48086	0.758526	0.937705	1.290958	1.994342	1.639268	1.754052	1.76398	0.833944	0.603178
Bermuda	0.026235	0.024327	0.037325	0.175004	0.309315	0.283307	0.27753	0.273713	0.318713	0.382167	0.510302	0.698821	0.929592	1.384808	1.720528	2.54612	3.181218	4.122462	3.098654	3.714309
Bhutan	0.03012	0.139877	0.159633	0.248434	0.271998	0.315283	0.30999	0.31222	0.317519	0.364876	0.458122	0.70079	0.898018	1.363718	1.76474	1.863253	2.352187	2.370415	0.877998	0.500089
Bolivia	0.08121	0.13541	0.138133	0.205634	0.227631	0.230656	0.252707	0.294597	0.371428	0.512392	0.847221	1.267131	1.753474	2.536942	3.28883	3.996698	4.859866	4.43336	3.4618	1.840337
Bosnia and Herzegovina	0.007659	0.016553	0.041942	0.160659	0.24122	0.269329	0.304258	0.279849	0.364828	0.435045	0.574278	0.905163	1.180603	1.526346	1.968448	1.918354	2.303066	2.623879	1.764803	1.067229
Botswana	0.015935	0.03781	0.043236	0.076635	0.084727	0.128989	0.149244	0.165144	0.201738	0.275825	0.337934	0.516769	0.724477	0.849347	1.245296	0.934304	1.576653	1.683548	1.564797	0.508714
Brazil	0.015984	0.035107	0.070603	0.16244	0.233191	0.266623	0.279331	0.254497	0.252432	0.313755	0.427974	0.629956	0.791919	1.045463	1.375126	1.200381	1.414375	1.516325	1.329638	0.934713
Brunei	0.024022	0.026133	0.052168	0.250758	0.390647	0.380932	0.35929	0.320733	0.33986	0.400004	0.647391	0.992672	1.345726	2.190656	2.76745	2.484474	2.587253	2.629773	2.10203	0.96783
Bulgaria	0.026128	0.055735	0.096065	0.253117	0.380394	0.518023	0.547039	0.62313	0.637607	0.761162	1.025381	1.381631	1.588649	1.862942	1.923287	1.646248	1.663651	1.786854	1.28289	1.156722
Burkina Faso	0.134336	0.0467	0.0766	0.183727	0.20486	0.242112	0.256155	0.290678	0.377816	0.395357	0.507576	0.757064	0.92766	1.305732	1.919605	1.629935	1.71064	1.737364	0.82125	0.612287
Burundi	0.223705	0.271837	0.298349	0.460919	0.533956	0.691431	0.698157	0.728115	0.778739	0.843099	1.020093	1.452289	1.783411	2.498961	3.053629	2.952226	3.459722	3.109195	1.103141	0.651158
Cambodia	0.036853	0.04548	0.08652	0.171375	0.219942	0.300538	0.264284	0.339552	0.428434	0.627544	0.959557	1.191458	1.561008	2.314057	2.955252	2.045218	2.216858	1.862985	1.294762	1.112259
Cameroon	0.07653	0.05424	0.092919	0.19197	0.201659	0.226078	0.2385	0.272218	0.347757	0.376961	0.501142	0.785171	0.987434	1.392499	2.081834	1.716121	1.846529	1.883092	0.883712	0.660957
Canada	0.010749	0.010399	0.027092	0.115145	0.225703	0.293489	0.283702	0.271229	0.272945	0.294023	0.360227	0.446145	0.576693	0.816339	1.123788	1.429505	1.862161	2.05762	1.568518	1.105641
Cape Verde	0.015665	0.008913	0.012867	0.030741	0.03527	0.050477	0.059306	0.077538	0.093566	0.101588	0.113027	0.181171	0.177949	0.253666	0.427462	0.378809	0.452754	0.528777	0.327639	0.279096
Central African Republic	0.140207	0.104908	0.132523	0.267767	0.417653	0.525105	0.593364	0.657323	0.77591	0.99756	1.332417	1.750151	2.153749	2.771213	3.165545	2.804467	2.929183	2.737226	1.733457	1.129688
Chad	0.121133	0.081226	0.118656	0.209881	0.221856	0.246018	0.259786	0.29447	0.377418	0.421351	0.568636	0.909202	1.131944	1.600297	2.467472	1.947289	2.054528	2.097386	0.881364	0.7138
Chile	0.012451	0.023729	0.053197	0.122602	0.191752	0.207022	0.198192	0.212845	0.227875	0.25435	0.370273	0.540948	0.783396	1.254859	1.760172	1.418739	1.99746	2.532792	1.808606	2.045788
China	0.032819	0.038949	0.033594	0.030558	0.038748	0.05485	0.072654	0.06415	0.084883	0.133345	0.203219	0.262527	0.441724	0.685026	0.802604	0.904614	1.059058	1.052329	0.831113	0.794565
Colombia	0.046915	0.069698	0.08357	0.132235	0.18621	0.215719	0.218778	0.233606	0.260102	0.330675	0.452172	0.62112	0.934037	1.292854	1.8581	1.837436	1.9132	2.211797	1.690589	1.534077
Comoros	0.138054	0.28446	0.336488	0.498248	0.597918	0.756659	0.736774	0.796135	0.771789	0.804677	0.965954	1.342119	1.614971	2.246491	2.76705	2.704648	3.185584	2.854525	0.983118	0.553502
Congo	0.051979	0.056729	0.080959	0.174905	0.277828	0.335753	0.360672	0.377718	0.442562	0.561994	0.77015	1.100035	1.416213	1.882106	2.312546	2.17295	2.39621	2.461527	1.575533	1.076891
Costa Rica	0.028165	0.067256	0.092509	0.287753	0.348972	0.499437	0.477454	0.55494	0.493052	0.698059	0.862077	1.210628	1.687216	2.551883	3.383837	4.03046	4.492118	5.363288	4.351339	2.740422
Cote d'Ivoire	0.129695	0.10872	0.157773	0.255541	0.279842	0.334947	0.350738	0.365381	0.41691	0.425265	0.539376	0.826504	0.974618	1.343023	1.931055	1.625637	1.660837	1.594186	0.632366	0.436956
Croatia	0.011546	0.016909	0.027157	0.091721	0.168735	0.238079	0.263358	0.261946	0.314111	0.330043	0.457864	0.769122	0.990226	1.33294	1.457852	1.507128	1.897286	2.118123	1.278545	1.472997
Cuba	0.0218	0.029486	0.055636	0.193936	0.351686	0.450712	0.473107	0.477301	0.543432	0.674122	0.834356	1.052281	1.41737	2.065498	2.488839	3.003238	3.416393	3.510563	2.569509	3.10863
Cyprus	0.00506	0.010307	0.015418	0.104436	0.284867	0.224445	0.199767	0.20216	0.183221	0.220305	0.265587	0.577503	0.60666	1.1323	1.785296	1.658176	1.914234	0.859999	0.570817	0.413221
Czech Republic	0.01202	0.017113	0.039684	0.12835	0.257639	0.31996	0.326028	0.337869	0.346998	0.439681	0.567265	0.963564	1.333806	1.888593	2.303699	2.200994	2.725071	2.796764	2.165941	2.284603
Democratic Republic of the Congo	0.085919	0.054028	0.075281	0.149221	0.250153	0.303042	0.336869	0.354375	0.410278	0.559983	0.774408	1.083716	1.402249	1.833803	2.197491	2.045194	2.23586	2.233648	1.527245	1.014298
Denmark	0.009699	0.015517	0.022249	0.093949	0.165025	0.2296	0.235337	0.212188	0.232421	0.284854	0.429902	0.681307	0.994233	1.281021	1.95691	1.845545	2.413279	2.801191	2.069839	1.623142
Djibouti	0.14791	0.286863	0.333822	0.415972	0.460253	0.612678	0.624696	0.684315	0.696317	0.760926	0.927539	1.36082	1.654724	2.24959	2.852134	2.627407	3.061764	2.712254	0.915641	0.512457
Dominica	0.057896	0.049123	0.12307	0.244386	0.45437	0.405429	0.478503	0.398261	0.603353	0.537412	0.684005	0.835792	1.123222	1.476576	1.817094	1.993459	2.021274	2.080802	1.421507	1.187119
Dominican Republic	0.029339	0.046633	0.040943	0.081676	0.11562	0.09874	0.137114	0.138531	0.214403	0.244177	0.384864	0.544075	0.732461	0.889405	0.967006	1.038792	1.231934	1.631394	0.764507	1.333861
Ecuador	0.143199	0.10735	0.122185	0.1974	0.207981	0.264604	0.268756	0.258914	0.321649	0.330719	0.563157	0.817989	1.092222	1.408913	1.874895	2.805639	3.834169	4.113645	5.420815	4.642717
Egypt	0.0678	0.222239	0.411556	0.695379	0.778767	0.886914	0.940612	0.996175	1.057698	1.130647	1.42671	1.883864	2.524154	3.879487	5.320345	5.041252	6.060093	5.563675	2.557842	1.639118
El Salvador	0.038344	0.052575	0.100687	0.181208	0.170556	0.195558	0.246839	0.304877	0.303551	0.43378	0.659977	1.036274	1.470472	1.907469	2.842735	2.915874	3.575267	3.861194	4.290805	3.758217
Equatorial Guinea	0.029032	0.032718	0.043625	0.090349	0.138901	0.162737	0.180712	0.187572	0.207379	0.275466	0.375813	0.54982	0.746382	1.001479	1.258045	1.217751	1.339678	1.348842	0.871337	0.571414
Eritrea	0.326817	0.432471	0.483038	0.766364	0.911902	1.30246	1.410072	1.600668	1.699071	1.667788	1.743492	2.206362	2.396536	3.875201	3.67387	3.495717	3.832811	3.117024	1.343919	0.596226
Estonia	0.011169	0.016005	0.020975	0.166003	0.258027	0.333901	0.332277	0.328661	0.331051	0.341642	0.450814	0.670925	0.825836	1.08565	1.171383	1.183196	1.6393	1.599107	1.444352	0.591438
Ethiopia	0.252288	0.439599	0.50722	0.696032	0.797856	1.059708	1.044241	1.080241	1.039461	1.150046	1.441257	2.120715	2.702536	3.908815	5.126078	4.819247	5.732462	5.305244	1.569145	1.003222
Federated States of Micronesia	0.013899	0.070976	0.042537	0.085779	0.172015	0.247383	0.332086	0.257312	0.260459	0.415815	0.555285	0.511362	0.735023	0.931643	1.358842	1.352428	1.249734	1.824204	2.106521	1.265721
Fiji	0.030596	0.12691	0.079556	0.094825	0.156029	0.204252	0.269624	0.264134	0.235532	0.362513	0.452089	0.327154	0.492322	0.610414	0.922947	1.075655	0.935024	1.687118	1.955957	0.774797
Finland	0.009219	0.014325	0.034218	0.107095	0.198912	0.236519	0.242442	0.240395	0.223725	0.252531	0.357582	0.502602	0.718571	0.971247	1.244603	1.368791	1.787574	2.073536	1.608162	0.850323
France	0.011031	0.011226	0.018	0.075407	0.173527	0.273758	0.271616	0.253483	0.288478	0.343776	0.415588	0.630399	0.801759	1.052524	1.407251	1.232451	1.707227	2.041975	1.607651	1.35203
Gabon	0.041724	0.044149	0.062824	0.130201	0.191327	0.23639	0.266651	0.292742	0.340669	0.44748	0.630726	0.90833	1.177251	1.517986	1.768588	1.575605	1.756209	1.792806	1.203935	0.790734
Georgia	0.078689	0.060926	0.131217	0.202454	0.459999	0.489617	0.695326	0.755685	0.934187	1.327693	1.646019	2.538395	2.493276	3.26317	3.666091	3.64176	2.223132	3.815709	2.419372	1.282006
Germany	0.010941	0.011293	0.020363	0.076868	0.130728	0.169778	0.185475	0.192092	0.215836	0.279444	0.389313	0.576132	0.770305	1.087606	1.49279	1.460361	1.90988	2.116053	1.494747	1.023584
Ghana	0.032954	0.015361	0.030213	0.046196	0.045201	0.053521	0.059323	0.068846	0.114005	0.100368	0.142759	0.261393	0.296962	0.430265	0.87494	0.620534	0.678166	0.885849	0.883027	1.113804
Greece	0.017309	0.032881	0.052254	0.18886	0.367401	0.445568	0.495505	0.554931	0.59013	0.813984	1.210164	1.683659	2.403452	3.373288	4.503174	5.111501	6.644843	7.751235	8.083915	6.924367
Greenland	0.007687	0.011658	0.01618	0.04804	0.097339	0.096307	0.157067	0.190065	0.21616	0.200045	0.300185	0.588346	0.779083	1.219798	1.63866	1.396936	1.518585	0.990405	0.692408	0.568712
Grenada	0.013676	0.021347	0.038395	0.070958	0.109935	0.217702	0.210917	0.244064	0.248363	0.280152	0.476107	0.57563	0.706607	0.883986	0.881249	0.91793	0.896046	1.05458	0.818577	1.011593
Guam	0.013311	0.035558	0.020731	0.059227	0.056377	0.149667	0.231075	0.206746	0.190128	0.244544	0.433308	0.284626	0.39029	0.482489	0.699535	0.675227	0.550257	0.896756	1.052845	0.25089
Guatemala	0.135771	0.092483	0.087516	0.104836	0.12639	0.133619	0.171565	0.205658	0.253404	0.375028	0.556014	0.689668	0.961924	1.419178	2.012312	1.742306	2.214042	2.770427	2.791725	5.660466
Guinea	0.232297	0.15065	0.214623	0.350434	0.393282	0.469433	0.478915	0.496074	0.563859	0.5866	0.757143	1.163622	1.412234	1.983144	2.863685	2.419617	2.409122	2.181063	0.881517	0.615702
Guinea-Bissau	0.103373	0.084387	0.135105	0.251145	0.280856	0.330348	0.363904	0.403508	0.537168	0.554386	0.705746	1.059442	1.273952	1.802812	2.587115	2.022019	2.132626	2.145877	1.069796	0.866828
Guyana	0.022415	0.03424	0.05293	0.141572	0.29615	0.355105	0.368951	0.370058	0.408842	0.476015	0.606868	0.791591	0.944441	1.165822	1.28957	1.393062	1.42751	1.418221	0.940771	1.038346
Haiti	0.100207	0.092826	0.11843	0.278024	0.519035	0.51985	0.614746	0.683099	0.857616	1.04108	1.547422	2.116902	2.813317	3.838503	4.334999	4.822339	4.964388	5.389582	3.575629	3.022326
Honduras	0.021462	0.020836	0.01628	0.020164	0.029231	0.032422	0.04277	0.054944	0.072017	0.139785	0.25967	0.430639	0.691935	0.891438	1.070394	0.52078	0.479766	0.482934	0.333024	0.25776
Hungary	0.01126	0.017534	0.041057	0.102829	0.159853	0.215894	0.209928	0.234999	0.256197	0.362368	0.560656	0.823838	0.986784	1.282823	1.388243	1.326056	1.43131	1.573812	0.946184	0.635974
Iceland	0.009649	0.010309	0.020724	0.065389	0.115663	0.147366	0.165392	0.147985	0.17209	0.203187	0.26605	0.367163	0.520051	0.789292	1.160833	1.114159	1.435521	1.477719	1.048456	0.732084
India	0.026835	0.096582	0.116917	0.176764	0.239765	0.300776	0.362521	0.382366	0.405701	0.435739	0.551652	0.845203	0.917908	1.270827	1.671284	1.646107	2.27037	2.352577	0.931433	0.514295
Indonesia	0.034208	0.044984	0.088539	0.165736	0.18458	0.226173	0.19133	0.23473	0.292293	0.416941	0.640989	0.833807	1.161666	1.756095	2.299805	1.597496	1.851741	1.660165	1.179488	1.024486
Iran	0.032443	0.062945	0.111709	0.201609	0.233231	0.250591	0.255857	0.261451	0.271619	0.321407	0.469936	0.622623	0.751888	1.183439	1.567216	1.705028	2.422582	2.637147	2.32393	2.59576
Iraq	0.029452	0.070531	0.079178	0.11147	0.126506	0.154453	0.17953	0.181401	0.175134	0.228661	0.301266	0.351077	0.48462	0.592858	0.739166	0.653894	0.855245	1.177734	1.00609	1.286536
Ireland	0.009451	0.011289	0.035237	0.096479	0.184654	0.273784	0.23494	0.239332	0.262396	0.312029	0.43037	0.61414	0.775635	1.120434	1.570151	1.588661	2.200246	2.255106	1.687569	1.247995
Israel	0.010525	0.020538	0.038568	0.140666	0.226473	0.337131	0.347304	0.359337	0.346084	0.348597	0.485987	0.669385	0.818018	1.177431	1.578694	1.752156	2.265948	2.502174	2.198688	2.04036
Italy	0.010402	0.019892	0.036285	0.125607	0.268732	0.362424	0.332895	0.333987	0.33084	0.37303	0.487228	0.669986	0.938856	1.321449	1.975574	2.034135	2.75368	2.907333	2.260505	1.723575
Jamaica	0.01855	0.013803	0.030122	0.080971	0.226464	0.185728	0.251549	0.281584	0.243907	0.202697	0.342256	0.516438	0.72557	0.896553	0.878129	1.034798	1.035075	1.038037	0.506983	0.374633
Japan	0.009417	0.009494	0.009318	0.015879	0.032753	0.04114	0.049166	0.050043	0.066332	0.079573	0.111681	0.162794	0.23473	0.343742	0.45712	0.459185	0.559584	0.586521	0.446719	0.370532
Jordan	0.00956	0.011476	0.014859	0.033773	0.06228	0.067623	0.082875	0.077335	0.08119	0.099139	0.125751	0.176015	0.243418	0.398941	0.477195	0.566948	0.658478	0.931497	0.635007	0.410744
Kazakhstan	0.023003	0.03966	0.072607	0.169467	0.303	0.347933	0.477334	0.515593	0.459191	0.53162	0.617171	0.925124	1.008655	1.255149	1.175007	1.329422	1.022396	1.001326	0.698714	1.905634

Kenya	0.058355	0.129659	0.164609	0.25719	0.299145	0.395917	0.415519	0.444686	0.455275	0.486782	0.596428	0.857492	1.024961	1.429041	1.846462	1.655763	1.862432	1.648238	0.573113	0.325644
Kiribati	0.017938	0.076631	0.039962	0.066544	0.123795	0.180397	0.241109	0.212048	0.185547	0.256699	0.326717	0.327496	0.461844	0.501819	0.95829	0.831378	0.832646	1.111433	1.244312	0.854019
Kuwait	0.008705	0.019951	0.026636	0.047262	0.048037	0.076896	0.082739	0.081025	0.110948	0.140036	0.19905	0.273397	0.434526	0.578716	0.527304	0.628108	0.505912	1.668429	2.78668	0.487429
Kyrgyzstan	0.016555	0.044279	0.071458	0.092103	0.110807	0.119392	0.164496	0.228749	0.23729	0.268459	0.299061	0.754127	0.6678	0.92508	0.874325	1.104482	0.656938	0.49023	0.286554	0.238724
Laos	0.083737	0.091492	0.145156	0.224114	0.269512	0.359113	0.313694	0.371598	0.435195	0.611249	0.936233	1.181423	1.597935	2.299459	3.057293	1.922068	2.161472	1.85303	1.230708	1.066997
Latvia	0.01029	0.016246	0.041604	0.152314	0.329033	0.470898	0.550944	0.516501	0.466511	0.548831	0.650138	0.883638	1.177332	1.246341	1.408965	1.295959	1.133203	1.007418	0.67014	0.309887
Lebanon	0.024675	0.124294	0.252387	0.754321	0.886601	0.953697	0.903363	0.826626	0.826488	0.876496	1.082965	1.49956	1.891735	3.027501	4.047228	4.307891	5.600459	5.451553	3.053474	2.259296
Lesotho	0.026744	0.09881	0.106868	0.189762	0.206979	0.341055	0.455235	0.599785	0.724372	0.934326	1.01521	1.471586	1.838817	1.892575	2.439406	1.706026	2.764272	2.817123	2.603423	0.877398
Liberia	0.065991	0.043432	0.077721	0.146569	0.1677	0.191811	0.19984	0.22495	0.282747	0.312549	0.431434	0.666119	0.826038	1.145326	1.948257	1.463944	1.6379	1.832556	0.745665	0.653672
Libya	0.068896	0.211368	0.404505	0.87045	1.208468	1.307105	1.460196	1.268415	1.313946	1.370112	1.511489	2.003372	2.815853	4.080931	5.263204	5.167528	6.91146	6.598281	4.170217	4.102401
Lithuania	0.012623	0.020545	0.064607	0.141151	0.244741	0.386734	0.432086	0.448248	0.407346	0.443964	0.539321	0.770277	1.019093	1.249038	1.375019	1.292303	1.416701	1.390436	1.05339	0.792796
Luxembourg	0.00847	0.009245	0.015877	0.078305	0.152921	0.190062	0.172674	0.224989	0.189131	0.244956	0.349036	0.471813	0.665514	0.947495	1.322258	1.293171	1.655131	1.796468	1.640995	1.361775
Macedonia	0.012273	0.03936	0.04741	0.187377	0.349881	0.471984	0.485117	0.412152	0.530093	0.609164	0.915467	1.035588	1.381855	1.393821	1.879572	1.61257	1.237605	1.224623	0.423911	0.125424
Madagascar	0.156096	0.240246	0.288594	0.444019	0.544656	0.749773	0.74801	0.791263	0.783596	0.821364	0.99712	1.403217	1.587666	2.110801	2.583181	2.356557	2.702464	2.414077	0.834555	0.546132
Malawi	0.137366	0.171493	0.194167	0.273244	0.330042	0.423975	0.422008	0.441983	0.440227	0.475674	0.541671	0.753575	0.890854	1.230744	1.589534	1.489721	1.645601	1.417513	0.465886	0.286789
Malaysia	0.008208	0.024985	0.060605	0.139567	0.159021	0.211686	0.185596	0.210944	0.221931	0.288706	0.433345	0.540282	0.735716	1.099737	1.479809	1.204597	1.418563	1.07558	0.777833	0.763243
Maldives	0.011462	0.010323	0.014047	0.017319	0.027416	0.041676	0.04474	0.063752	0.067494	0.086675	0.123206	0.174878	0.276693	0.419773	0.525997	0.324152	0.397551	0.464243	0.378768	0.359256
Mali	0.393409	0.19333	0.219752	0.289313	0.314415	0.386502	0.397747	0.424117	0.465807	0.479077	0.6062	0.909548	1.087259	1.538	2.299196	2.000977	2.125644	2.177706	0.775159	0.600749
Malta	0.011177	0.021913	0.052046	0.1368	0.304569	0.375039	0.450523	0.412036	0.391332	0.414304	0.552185	0.799477	1.082537	1.614274	2.373437	2.443224	3.156576	3.310071	2.492519	1.571191
Marshall Islands	0.013578	0.0635	0.05266	0.104386	0.210251	0.285462	0.337677	0.317725	0.347518	0.48537	0.595488	0.635165	0.929288	1.257459	1.492121	1.538915	1.282209	1.93964	1.992904	1.031307
Mauritania	0.048706	0.05324	0.079705	0.129558	0.129112	0.148251	0.162332	0.176871	0.232196	0.247166	0.345198	0.542187	0.709025	1.068593	1.660233	1.362681	1.490483	1.610652	0.720215	0.558961
Mauritius	0.018262	0.025028	0.06207	0.144054	0.160291	0.31552	0.150938	0.16686	0.249939	0.296805	0.469723	0.385648	0.59506	0.953577	1.154625	0.65966	0.661562	0.564885	0.473691	0.794709
Mexico	0.062846	0.072509	0.090683	0.151386	0.221805	0.230989	0.246788	0.281183	0.319982	0.452876	0.624414	0.94647	1.292653	1.838173	2.55091	3.095902	3.859573	4.137205	3.263542	3.092198
Moldova	0.055872	0.094524	0.161317	0.355455	0.525479	0.651063	0.601956	0.634459	0.657029	0.837333	0.96939	1.232352	1.647135	2.116879	2.050517	1.389993	1.482111	1.14973	0.664708	1.010623
Mongolia	0.009202	0.016834	0.028957	0.045719	0.071319	0.085239	0.117341	0.148368	0.155015	0.239153	0.296767	0.386247	0.512323	0.697656	0.789718	0.527573	0.360205	0.434642	0.353698	0.333116
Montenegro	0.021529	0.060527	0.120477	0.218101	0.484268	0.593302	0.584086	0.592167	0.609001	0.826146	1.150735	1.854663	2.328916	2.916658	3.626227	3.2198	3.273687	3.524618	2.07249	1.658459
Morocco	0.062841	0.313391	0.46709	0.774257	0.904565	0.980792	1.093782	0.967625	0.901393	0.943179	1.138161	1.558113	1.98152	3.066255	4.170513	4.187757	5.390073	5.153685	3.016773	2.400351
Mozambique	0.246741	0.384896	0.40686	0.616656	0.690868	0.934298	0.96451	1.048469	1.086212	1.10878	1.260131	1.686481	1.929879	2.58608	3.237687	3.157503	3.646784	3.262813	1.108208	0.614674
Myanmar	0.068854	0.070061	0.121396	0.221956	0.273614	0.38017	0.331083	0.398592	0.471953	0.659172	0.997351	1.208237	1.616592	2.349799	2.971481	2.122624	2.328634	1.928587	1.306857	1.097412
Namibia	0.036602	0.114841	0.15154	0.28313	0.261244	0.361041	0.399378	0.445503	0.493541	0.607031	0.714373	1.047641	1.362337	1.595344	2.298684	1.782748	2.515853	2.34448	1.69745	0.542762
Nepal	0.026682	0.116738	0.146226	0.255693	0.28875	0.321614	0.32192	0.333636	0.367054	0.442633	0.572099	0.902012	1.119789	1.62703	2.115684	2.256468	2.810142	2.651042	0.981342	0.492285
Netherlands	0.009708	0.008701	0.01506	0.079913	0.165748	0.21809	0.242219	0.249165	0.221449	0.270848	0.367143	0.577985	0.820678	1.15594	1.634993	1.612831	2.270131	2.785019	3.093001	2.64095
New Zealand	0.009195	0.022402	0.023563	0.076383	0.132965	0.261963	0.203938	0.18396	0.221876	0.238755	0.256806	0.395091	0.544747	0.771085	1.153411	1.336106	1.964934	2.119818	1.288219	1.101025
Nicaragua	0.022262	0.04164	0.054773	0.083945	0.122149	0.11211	0.117853	0.114394	0.161062	0.261631	0.315449	0.49276	0.773894	0.925355	1.195372	1.436184	1.81709	2.213307	1.108098	1.376488
Niger	0.115398	0.052963	0.083197	0.169342	0.185648	0.21742	0.232737	0.266573	0.326495	0.352375	0.479632	0.78205	0.966322	1.341095	1.995619	1.665139	1.816384	1.885014	0.832024	0.658463
Nigeria	0.834398	0.673549	0.81408	0.990701	1.106306	1.344991	1.254474	1.214695	1.360565	1.314423	1.736068	2.573087	3.288598	4.8721	7.660898	7.173881	8.067207	8.392946	3.789699	2.351246
North Korea	0.067551	0.05121	0.049374	0.102714	0.142545	0.128927	0.163661	0.165459	0.212179	0.303587	0.440461	0.635688	0.807054	1.032109	1.108855	1.13355	1.161879	1.063201	0.771325	0.775206
Northern Mariana Islands	0.008574	0.01395	0.011558	0.022478	0.04184	0.05337	0.079304	0.090585	0.067371	0.143176	0.138226	0.200513	0.250851	0.43109	0.532809	0.626739	0.482642	0.752038	0.708283	0.296454
Norway	0.007892	0.0091	0.02415	0.081297	0.126777	0.171901	0.175588	0.173011	0.172947	0.210839	0.289929	0.426317	0.582426	0.877355	1.243015	1.216723	1.641612	1.828903	1.371012	0.920357
Oman	0.022404	0.068313	0.122956	0.222129	0.284365	0.276345	0.290273	0.270318	0.313235	0.402432	0.555116	0.872812	1.108595	1.631073	1.99609	1.930187	2.442559	2.300435	1.154423	0.722138
Pakistan	0.179207	0.857195	0.991705	1.307342	1.449998	1.607244	1.276345	1.371088	1.198704	1.189652	1.602618	2.058718	2.889105	3.552166	4.754051	4.409855	6.194676	6.811201	2.663715	1.884832
Palestine	0.019326	0.039942	0.07076	0.146927	0.214981	0.367521	0.331035	0.321842	0.358444	0.57365	0.656496	0.768682	0.986577	1.424224	1.867543	1.952462	1.945645	2.068799	1.661588	0.601406
Panama	0.062674	0.07783	0.074678	0.127338	0.093527	0.147507	0.184896	0.150121	0.145635	0.200851	0.321814	0.462007	0.621812	0.916568	1.193445	1.39119	1.552882	1.840749	0.710951	0.491198
Papua New Guinea	0.026368	0.129666	0.074397	0.134099	0.274309	0.394397	0.608805	0.531131	0.533453	0.717633	0.935058	0.783496	1.051426	1.357842	1.765386	1.720959	1.285253	1.721384	1.661548	0.831063
Paraguay	0.00985	0.060855	0.104864	0.177776	0.185417	0.206978	0.284452	0.251025	0.290735	0.432282	0.590186	0.886328	1.122659	1.57117	2.013733	1.611476	1.737017	1.809305	1.435501	0.528161
Peru	0.018723	0.042631	0.047985	0.064871	0.078974	0.083269	0.097008	0.112136	0.132021	0.204353	0.322004	0.446759	0.613159	0.902167	1.153835	1.374978	1.630441	1.523324	1.374808	0.784461
Philippines	0.028934	0.028201	0.05557	0.081622	0.088764	0.137113	0.108343	0.129412	0.149088	0.228816	0.316684	0.375874	0.546157	0.803532	1.066819	0.683706	0.847111	0.933817	0.918033	0.912608
Poland	0.011665	0.02569	0.042727	0.127865	0.234654	0.37191	0.387563	0.392554	0.429565	0.507729	0.686896	1.030038	1.31763	1.755841	2.076227	1.623864	1.632589	1.52416	1.117736	0.808418
Portugal	0.013079	0.009498	0.021812	0.077672	0.159602	0.216473	0.200525	0.263728	0.281946	0.362516	0.514148	0.646329	0.822259	1.123785	1.441405	1.442639	1.503827	2.084348	1.741175	1.339204
Puerto Rico	0.012117	0.010325	0.029707	0.150729	0.343313	0.296962	0.403354	0.38698	0.370424	0.371556	0.529693	0.698831	0.921464	1.201245	1.420373	1.769681	1.966765	2.275056	1.139463	0.469246
Qatar	0.006682	0.022567	0.01931	0.049153	0.17348	0.070242	0.060231	0.052921	0.076109	0.086643	0.183572	0.350461	0.477526	0.650733	0.705622	0.544436	0.63902	0.734558	0.609672	0.615462
Romania	0.019234	0.041088	0.071938	0.155013	0.24266	0.328154	0.367651	0.374296	0.420174	0.548249	0.741071	1.072713	1.164468	1.406028	1.446445	1.32015	1.208519	1.124617	0.716929	0.416143
Russian Federation	0.016359	0.036258	0.067654	0.173129	0.345704	0.506549	0.591708	0.65479	0.572226	0.562779	0.631043	0.818766	0.99063	1.183706	1.404646	1.265406	1.326549	1.050162	0.73853	0.519257
Rwanda	0.185542	0.232752	0.270787	0.376792	0.424569	0.54551	0.545412	0.579041	0.572025	0.624614	0.778	1.126157	1.37807	1.918769	2.473296	2.426756	2.847781	2.567116	0.914941	0.48752
Saint Lucia	0.020906	0.033405	0.064545	0.146681	0.299555	0.391042	0.461553	0.407131	0.502297	0.5391	0.75275	0.988178	1.108735	1.435542	1.647778	1.86516	1.800106	2.047355	1.663363	2.05297
Saint Vincent and the Grenadines	0.026434	0.049887	0.105452	0.233477	0.383057	0.422848	0.627599	0.525011	0.627467	0.702532	0.869578	1.004984	1.321288	1.790831	2.025978	2.314234	1.951387	1.75303	1.231003	1.420413
Samoa	0.017499	0.077555	0.079169	0.174183	0.258879	0.28457	0.356818	0.288833	0.296882	0.434006	0.57784	0.632712	0.919304	1.254445	1.740158	1.718039	1.497763	1.92823	1.712926	0.910483
Sao Tome and Principe	0.022783	0.01746	0.016806	0.032117	0.03778	0.039203	0.037957	0.043291	0.052173	0.064715	0.097473	0.155881	0.198008	0.270499	0.371058	0.293208	0.319009	0.425812	0.453219	0.918389
Saudi Arabia	0.019126	0.043403	0.088367	0.303432	0.339532	0.361346	0.318711	0.344752	0.40138	0.495221	0.656077	0.895724	1.056514	1.604685	2.252829	2.471348	3.125199	3.178145	1.500456	0.917562
Senegal	0.078007	0.058756	0.095934	0.193169	0.194401	0.21569	0.224329	0.25666	0.347457	0.371638	0.524136	0.855493	1.06464	1.554489	2.290051	1.862034	2.011617	2.034313	0.888877	0.688341
Serbia	0.008803	0.021504	0.048165	0.141248	0.289559	0.52683	0.537246	0.506028	0.573898	0.717767	0.912518	1.279689	1.697487	2.000605	2.447251	2.355871	2.274301	2.280761	1.459794	0.759478
Seychelles	0.028873	0.035085	0.052414	0.122841	0.176711	0.24326	0.245312	0.279837	0.349883	0.490842	0.707582	0.870341	1.205045	1.834527	2.110037	2.161636	2.558413	1.996557	2.072095	2.23049
Sierra Leone	0.107029	0.053379	0.088408	0.186018	0.207649	0.236069	0.241596	0.266446	0.331658	0.347443	0.448768	0.698443	0.856396	1.234868	1.928488	1.613595	1.79057	1.869126	0.833725	0.689597
Singapore	0.006054	0.00952	0.010212	0.032089	0.061747	0.070268	0.066149	0.059681	0.073659	0.086252	0.128373	0.203904	0.271729	0.408447	0.550126	0.498934	0.579784	0.581472	0.482219	0.346717
Slovakia	0.014582	0.024376	0.037217	0.143101	0.311401	0.396261	0.418064	0.353949	0.412471	0.487712	0.683004	0.950807	1.306374	1.762514	2.053496	1.899586	2.159768	2.358419	1.462325	0.828292
Slovenia	0.009312	0.012661	0.051101	0.117705	0.182694	0.183737	0.192878	0.218683	0.240964	0.303759	0.446579	0.697822	0.932609	1.284523	1.570917	1.613578	2.106617	2.46913	1.842283	1.142216
Solomon Islands	0.015546	0.079978	0.054342	0.105692	0.209283	0.274032	0.40211	0.348149	0.373022	0.529229	0.702787	0.635549	0.879757	1.096071	1.584074	1.619864	1.341371	1.958074	1.968562	0.849434
Somalia	0.353865	0.463161	0.531323	0.711753	0.842218	1.156885	1.175685	1.255201	1.31221	1.393334	1.677786	2.300437	2.678324	3.651867	4.528276	4.154315	4.594893	4.164933	1.415181	0.940704
South Africa	0.021716	0.026516	0.020474	0.053387	0.088221	0.151298	0.196256	0.226969	0.253712	0.330501	0.402247	0.621865	0.784626	0.818711	1.022856	0.682767	1.174155	1.300172	1.333536	0.447733
South Korea	0.009943	0.010198	0.010312	0.019877	0.033363	0.039001	0.047831	0.044886	0.060541	0.06821	0.101595	0.146379	0.196959	0.325553	0.443034	0.461242	0.549941	0.609551	0.550169	0.300328
South Sudan	0.303379	0.335317	0.366869	0.49367	0.597253	0.808877	0.81891	0.894801	0.923331	0.981516	1.198936	1.741562	2.126298	2.63319	3.448186	3.186439	3.663307	3.401266	1.009458	0.635281
Spain	0.010288	0.013541	0.025931	0.089326	0.15912	0.22021	0.226761	0.224032	0.235121	0.322232	0.428991	0.6049	0.818018	1.084684	1.492415	1.437751	2.289783	2.399997	1.966675	1.583693
Sri Lanka	0.017754	0.022746	0.047955	0.094215	0.114803	0.156936	0.158385	0.205943	0.284471	0.407345	0.604425	0.758166	1.038961	1.519032	2.141326	1.566645	2.020218	1.987722	1.554242	1.378393
Sudan	0.073506	0.139143	0.189566	0.27778	0.357966	0.379873	0.402697	0.403273	0.430287	0.52661	0.74704	1.095667	1.368342	2.134798	2.875143	2.69721	3.416647	3.488163	2.130696	1.706094
Suriname	0.042086	0.04032	0.092058	0.236145	0.348998	0.462495	0.513964	0.527803	0.555436	0.595296	0.844693	1.08832	1.301236	1.785797	1.872597	2.253619	2.376869	2.544596	1.561674	1.551867
Swaziland	0.02433	0.075049	0.084621	0.163068	0.175073	0.288189	0.380704	0.500939	0.624378	0.799671	0.834417	1.203487	1.45962	1.53537	1.921466	1.289854	2.094014	2.101192	1.953412	0.689195
Sweden	0.010596	0.013163	0.018423	0.061269	0.123253	0.171989	0.162048	0.160163	0.153439	0.173174	0.21081	0.338977	0.493221	0.747684	1.051818	1.107873	1.646209	1.905009	1.452155	0.962311
Switzerland	0.009453	0.011114	0.019583	0.066213	0.118249	0.148077	0.160023	0.141247	0.153386	0.211139	0.255428	0.383886	0.543372	0.770965	1.24954	1.182735	1.714612	1.830631	1.308896	0.637549
Syria	0.008048	0.008276	0.012482	0.024755	0.034205	0.045031	0.045365	0.049538	0.064954	0.07967	0.125721	0.201541	0.251619	0.380446	0.491692	0.45217	0.454856	0.521049	0.322964	0.216756
Taiwan	0.011757	0.012217	0.014373	0.028431	0.0333	0.035686	0.055291	0.056316	0.064298	0.100627	0.126427	0.196361	0.226033	0.307828	0.424166	0.424264	0.491543	0.589093	0.556746	0.196709
Tajikistan	0.015949	0.019875	0.024576	0.052406	0.094976	0.095773	0.138421	0.216774	0.223387	0.218272	0.387082	0.564419	0.701235	0.882451	1.255794	0.804171	0.665358	0.813677	0.495837	0.307071
Tanzania	0.267674	0.297019	0.330717	0.402349	0.450139	0.603134	0.607749	0.636136	0.602161	0.655595	0.816815	1.178245	1.433515	1.988243	2.56914	2.478936	2.852788	2.584396	0.862183	0.489941
Thailand	0.027619	0.022746	0.033134	0.065477	0.077347	0.120842	0.126246	0.140324	0.148912	0.228191	0.321044	0.410202	0.540566	0.804146	1.029512	0.62232	0.698497	0.648691	0.424838	0.315707
The Bahamas	0.018517	0.022881	0.038214	0.119836	0.270678	0.360449	0.40362	0.374288	0.355313	0.386934	0.481093	0.614789	0.697752	1.031169	1.137158	1.349739	1.469017	1.615018	1.070969	1.177974
The Gambia	0.103617	0.094096	0.133244	0.231469	0.255769	0.303278	0.319043	0.337985	0.396223	0.414809	0.544801	0.833104	0.981404	1.43864	2.090408	1.680349	1.696705	1.530072	0.632037	0.422592
Timor-Leste	0.05162	0.070552	0.121323	0.202269	0.223374	0.26946	0.23056	0.283754	0.363164	0.534068	0.882311	1.140418	1.570338	2.179796	2.754488	2.001587	2.291283	2.145746	1.30925	1.15351
Togo	0.059929	0.040827	0.073862	0.166632	0.18557	0.215521	0.228338	0.257913	0.347876	0.375735	0.498476	0.765144	0.899961	1.268173	1.714114	1.458171	1.669406	1.63471	0.798124	0.571541
Tonga	0.014559	0.026122	0.022865	0.068061	0.113353	0.139555	0.175763	0.168837	0.152353	0.244341	0.340368	0.326422	0.459606	0.639414	0.923526	0.933117	0.847458	1.275981	1.462244	0.799339
Trinidad and Tobago	0.020614	0.016975	0.03444	0.133228	0.261771	0.330753	0.306741	0.332451	0.261273	0.403481	0.52226	0.564498	0.560442	0.721934	0.746416	1.49232	1.277465	0.647259	0.339022	0.240543
Tunisia	0.036964	0.108868	0.190238	0.300252	0.364108	0.445331	0.50395	0.469296	0.483399	0.536597	0.701054	1.060133	1.369411	2.189032	2.837954	2.860784	4.259202	4.712972	2.540293	1.814719
Turkey	0.023719	0.044139	0.077828	0.144316	0.210151	0.228473	0.226553	0.192314	0.223527	0.275431	0.385013	0.544606	0.720707	1.116923	1.518019	1.347281	1.364018	1.19307	0.593579	0.39306
Turkmenistan	0.044745	0.044745	0.120975	0.255439	0.501512	0.567613	0.535817	0.601862	0.536263	0.570395	0.548662	0.910128	0.912225	1.242073	1.037896	0.972973	0.711871	0.903939	0.657633	0.573661

Uganda	0.23286	0.298213	0.336392	0.49348	0.568184	0.731922	0.745204	0.760749	0.757726	0.826754	0.985659	1.414265	1.730237	2.361024	3.065209	3.091573	3.614278	3.153263	1.14629	0.596985
Ukraine	0.035605	0.062644	0.145087	0.591195	1.081121	1.372274	1.369738	1.313179	1.139839	1.127618	1.136514	1.344497	1.470905	1.6695	1.6948	1.329487	1.041016	0.899995	0.60478	0.402871
United Arab Emirates	0.031831	0.052428	0.117336	0.294065	0.26668	0.275939	0.266952	0.285243	0.357922	0.440645	0.654705	1.023019	1.536852	2.013245	1.945019	1.463905	1.836851	2.033164	1.319296	1.168558
United Kingdom	0.009626	0.009848	0.021036	0.103315	0.209844	0.291571	0.289762	0.301681	0.309427	0.345924	0.417663	0.628196	0.864531	1.279021	1.713486	1.642437	2.072716	2.263549	1.826984	1.447595
United States	0.009834	0.010115	0.018857	0.073494	0.196643	0.286932	0.313787	0.288809	0.269076	0.299515	0.359522	0.478547	0.604942	0.8433	1.186414	1.599002	2.099569	2.291421	1.791977	1.240967
Uruguay	0.011985	0.025032	0.056681	0.183109	0.344835	0.366942	0.444769	0.377895	0.4544	0.493964	0.757681	1.062301	1.546866	2.160128	2.60147	1.53727	2.00682	2.429877	1.016953	0.367654
Uzbekistan	0.064961	0.133491	0.165622	0.19856	0.277795	0.307456	0.304238	0.374703	0.463416	0.571754	0.751677	1.086852	1.262089	1.527092	1.605571	1.521532	1.14679	1.320003	0.967264	0.830573
Vanuatu	0.017988	0.125255	0.07368	0.158458	0.361715	0.472044	0.710182	0.623809	0.650518	0.889264	1.204057	1.069469	1.48709	1.889913	2.560697	2.600275	2.014685	2.815773	2.799941	1.395592
Venezuela	0.034017	0.05361	0.08459	0.246441	0.318838	0.375662	0.353562	0.332041	0.343679	0.505344	0.72139	1.000982	1.47376	1.708126	2.315606	2.320951	2.99614	2.702856	2.612763	2.298532
Vietnam	0.02278	0.033666	0.075886	0.187758	0.205423	0.242725	0.204036	0.244577	0.299871	0.425249	0.605496	0.769802	0.964322	1.381359	1.830754	1.336168	1.573741	1.346292	0.743211	0.512731
Virgin Islands, U.S.	0.021074	0.02066	0.019718	0.052696	0.08657	0.099524	0.126138	0.159153	0.211961	0.289707	0.41821	0.530193	0.670136	0.921325	1.047986	1.121774	1.08596	1.108868	0.716996	0.734533
Yemen	0.040254	0.131881	0.19462	0.320514	0.414319	0.465697	0.502578	0.521812	0.594875	0.712222	0.973166	1.351234	1.654818	2.726942	3.242413	3.187354	3.967287	3.807599	2.232465	1.727338
Zambia	0.244395	0.305439	0.344372	0.486205	0.581676	0.756664	0.773914	0.822038	0.856274	0.902185	1.078138	1.485212	1.790806	2.471623	3.086554	3.015034	3.485006	3.170788	1.09346	0.657228
Zimbabwe	0.04226	0.162212	0.197688	0.333301	0.328593	0.480897	0.562056	0.667877	0.682702	0.803829	0.86849	1.126366	1.457424	1.645694	2.318042	1.704901	2.116329	1.884744	1.392897	0.513737

Supplementary Table S8. Age distribution of DALYs rate (per 100,000) for Hodgkin lymphoma in different countries in 2017.

country	Under 5	5 to 9	10 to 14	15 to 19	20 to 24	25 to 29	30 to 34	35 to 39	40 to 44	45 to 49	50 to 54	55 to 59	60 to 64	65 to 69	70 to 74	75 to 79	80 to 84	85 to 89	90 to 94	95 plus
Afghanistan	10.08151	17.91282	27.84805	52.71519	71.61921	77.52247	75.69937	67.88776	66.79254	66.86289	82.48744	100.1579	98.21154	106.8377	113.4479	82.0598	75.00789	55.47131	25.63869	14.75303
Albania	3.745898	6.618444	7.38391	14.88582	23.50404	29.63818	25.22821	19.77743	21.35029	19.35568	21.88729	28.60628	29.02716	31.96673	33.28162	22.65414	16.30698	18.46627	9.244937	3.927774
Algeria	3.751322	18.80008	28.58308	41.74338	45.67483	45.76197	44.12614	38.22941	32.41694	29.97158	32.53032	37.33001	41.41193	53.03039	55.01514	45.36813	43.55976	33.53087	13.13794	5.706725
American Samoa	0.656996	1.078439	0.856541	2.178121	6.019258	6.199361	8.328609	4.42114	4.636808	6.568569	7.003396	7.456321	9.536179	9.14623	12.72594	9.259408	7.125237	8.574157	7.819018	4.129742
Andorra	0.949263	1.067665	1.954227	8.275829	16.54681	19.74189	17.44458	16.03485	13.2073	14.47495	16.02585	19.73212	23.22989	26.51252	29.81005	20.11644	18.25242	13.87478	8.320895	3.611912
Angola	5.362721	4.829952	6.048432	11.85369	17.46473	19.02049	18.84187	17.79823	18.18912	21.17374	25.97817	31.64527	35.59429	38.86035	37.99639	26.75437	21.68644	15.47474	6.912148	2.683265
Antigua and Barbuda	1.579818	1.912831	1.891808	5.062546	7.661928	6.407349	6.943504	5.964438	4.855125	5.466745	7.094471	9.500454	10.5663	11.36498	10.85195	10.64713	7.440011	5.495076	2.422098	1.026931
Argentina	1.422586	2.845794	4.336749	10.62256	17.60258	17.89664	18.1401	16.81617	15.46656	17.44133	25.55108	31.73464	35.87923	40.71638	44.32176	25.97275	27.81906	18.04569	9.039996	2.756184
Armenia	1.003071	1.852539	2.214585	5.375036	8.995437	6.65108	11.68914	6.520972	8.108532	10.90431	14.398	16.69941	17.53557	16.64036	13.5872	13.79182	8.603093	5.941545	2.706783	1.178448
Australia	1.026334	1.293345	2.80166	7.176212	11.72926	16.36015	13.61262	12.3696	11.1164	10.66567	11.14232	14.11279	16.36679	20.69957	24.18127	20.63245	20.39755	16.85053	7.037744	2.694238
Austria	0.923064	1.007404	1.236106	5.05654	9.204032	12.91135	12.43403	11.07715	10.48587	10.94535	12.87692	16.76245	19.72825	22.30706	25.38438	17.57568	17.52175	15.71881	9.780901	3.161473
Azerbaijan	3.544349	8.492051	13.38496	24.8302	35.31915	31.87994	32.25169	28.94169	25.82513	26.77488	30.40348	37.03471	35.80902	40.49092	29.0954	17.54622	10.75039	15.40508	10.29643	5.220781
Bahrain	0.820719	1.873808	3.406265	6.008739	9.917649	10.34793	8.183802	8.196092	8.180926	7.836953	12.00676	19.48841	30.35952	33.14479	28.35321	15.48916	16.24908	9.520387	4.481522	2.211821
Bangladesh	3.600517	14.51371	17.44649	18.03682	18.17198	20.61165	15.91437	15.23578	14.17796	14.10309	17.31363	19.9221	20.77956	22.81416	21.0367	17.32512	17.77644	15.78243	4.651729	1.759751
Barbados	1.195779	1.411946	4.797123	10.37135	18.25239	17.64724	18.71023	15.70934	13.2609	16.00881	16.23762	18.44746	20.35915	23.2144	20.85325	20.38757	17.90584	16.01551	9.687635	6.563648
Belarus	1.476341	3.061744	4.827831	16.49625	28.6717	31.51184	33.50661	33.9837	28.983	28.90239	29.50676	33.92929	35.61509	36.34226	33.80527	21.18723	16.08802	10.83062	5.041697	2.099037
Belgium	1.006634	1.169348	1.563487	7.486179	11.70549	17.10958	16.02633	14.21908	13.13423	14.69307	16.82814	20.51705	23.86459	27.54794	27.2567	20.32595	20.13642	15.95323	9.280793	4.239172
Belize	2.542644	2.512194	4.023684	13.1757	22.69374	24.70739	26.27569	21.97148	21.2597	21.29406	23.75068	28.93406	29.72936	31.30495	27.40923	23.75147	20.14886	15.10448	6.512668	3.807258
Benin	8.327969	4.060015	6.112181	11.6769	12.20114	13.17375	12.63884	12.82416	14.99893	14.86517	17.61281	24.21222	25.61079	29.45568	36.95095	23.75722	19.03208	13.86694	4.56567	1.976003
Bermuda	2.606541	2.247931	3.2247	14.20588	24.1924	19.77062	17.58334	15.4299	15.86794	16.88689	19.70159	23.1707	26.25618	32.01428	32.04181	36.88877	34.16992	32.0755	16.82721	11.27614
Bhutan	2.609669	11.38217	12.12767	17.64369	18.02254	19.33981	17.44119	15.99233	14.6842	15.09046	16.76464	22.34058	24.48053	31.0516	32.59391	26.87063	25.37715	18.51851	4.774052	1.604534
Bolivia	7.063373	11.02361	10.49792	14.60528	15.08885	14.15183	14.22129	15.09272	17.17929	21.19279	31.00768	40.40152	47.82247	57.79195	60.77712	57.70042	52.51049	34.69747	18.87434	5.946091
Bosnia and Herzegovina	0.721506	1.442602	3.419103	12.22905	17.39391	17.53763	18.0493	14.91304	17.29549	18.30517	21.24425	28.96191	32.28956	34.72905	36.27062	27.63323	24.8551	20.55031	9.632857	3.463765
Botswana	1.385108	3.079734	3.288071	5.437562	5.614209	7.906456	8.399964	8.465729	9.349232	11.45326	12.42475	16.54237	19.7956	19.3709	23.03227	13.49264	17.04029	13.1739	8.533512	1.638149
Brazil	1.417496	2.901613	5.455036	11.75455	15.85183	16.67982	15.98047	13.20796	11.7841	13.0884	15.75812	20.17914	21.68752	23.86369	25.42858	17.31521	15.22372	11.80532	7.173254	2.830192
Brunei	2.199666	2.24198	4.170265	18.7917	27.61395	24.58986	21.11381	17.01572	16.19351	17.00637	24.2779	32.26742	37.48089	50.46305	51.70938	36.32524	28.17271	20.77218	11.63496	3.11263
Bulgaria	2.525318	5.016269	8.064576	19.90022	28.61728	35.02348	33.61659	33.91981	30.89419	33.10454	39.01741	45.24694	44.69061	43.2561	35.96214	23.84446	17.97911	14.00081	7.01133	3.779844
Burkina Faso	11.61639	3.79686	5.817293	13.02303	13.53807	14.8226	14.39072	14.88125	17.47345	16.36205	18.59989	24.18323	25.34887	29.81751	35.59287	23.65592	18.5951	13.68545	4.505235	2.020329
Burundi	19.32809	22.08859	22.64405	32.64594	35.25498	42.28199	39.18108	37.21758	35.97868	34.8788	37.36978	46.37799	48.73641	57.09676	56.67702	42.9002	37.6643	24.53987	6.062043	2.158211
Cambodia	3.196524	3.700734	6.572155	12.15947	14.56106	18.41469	14.84905	17.3718	19.80012	25.9608	35.13987	38.02377	42.61943	52.79825	54.74675	29.58394	24.02198	14.64279	7.08212	3.60696
Cameroon	6.615245	4.408506	7.053871	13.60244	13.30906	13.81262	13.38269	13.92902	16.09136	15.61645	18.37497	25.0848	26.97818	31.79496	38.59319	24.88926	20.05104	14.81959	4.839388	2.168353
Canada	1.119869	1.02707	2.648703	10.52117	19.80848	22.86907	20.31368	17.04852	14.65457	14.04426	15.04546	15.72661	17.38866	20.04999	22.18907	21.90466	20.48626	16.54404	8.928131	3.557167
Cape Verde	1.365564	0.727958	0.980286	2.188837	2.344228	3.099608	3.337791	3.977856	4.337995	4.219406	4.146588	5.796299	4.870028	5.763019	7.890933	5.453409	4.855182	4.109961	1.770863	0.863871
Central African Republic	12.13067	8.521465	10.06163	18.97188	27.57135	32.08991	33.2887	33.61243	35.88632	41.33495	48.90247	56.03103	58.98875	63.49158	58.95892	40.91715	32.07125	21.72111	9.575099	3.746398
Chad	10.47163	6.601386	9.007004	14.86785	14.64639	15.03954	14.57785	15.06931	17.45648	17.44479	20.84155	29.03735	30.92753	36.53278	45.75604	28.26702	22.34056	16.52498	4.840142	2.357139
Chile	1.149663	2.043515	4.278682	9.239225	13.65435	13.50465	11.7887	11.4088	10.91563	10.88336	13.9375	17.60897	21.79277	28.84472	32.77106	20.58099	21.54109	19.80878	9.860278	6.307619
China	3.311061	3.669315	3.069053	2.63601	3.200139	4.059156	4.888924	3.806136	4.318375	5.986747	8.06188	9.047975	13.22358	16.88739	15.88594	13.79069	11.81963	8.634326	4.816204	2.656748
Colombia	4.222935	5.832618	6.530835	9.651807	12.79993	13.62157	12.64164	12.22541	12.22537	13.86298	16.69104	19.89527	25.57682	29.48023	34.31834	26.44735	20.5137	17.13915	9.041331	4.407377
Comoros	11.92392	23.11491	25.53181	35.299	39.49964	46.30628	41.37469	40.72116	35.6627	33.26236	35.34633	42.79935	44.05738	51.22867	51.23163	39.14017	34.5393	22.43108	5.372767	1.812959
Congo	4.495462	4.610148	6.145347	12.39611	18.343	20.52377	20.25187	19.32906	20.46641	23.27065	28.23229	35.14381	38.70058	42.98432	42.88988	31.54282	26.08054	19.2446	8.65905	3.578177
Costa Rica	2.586936	5.738089	7.392545	21.55808	24.71699	32.29312	28.12334	29.49605	23.46504	29.59738	32.13648	39.03373	46.48198	58.32281	62.57663	58.16556	48.29459	41.72754	23.42955	8.037026
Cote d'Ivoire	11.20708	8.836709	11.97705	18.09423	18.46622	20.47179	19.68599	18.70001	19.28438	17.61241	19.77911	26.40816	26.63888	30.67201	35.80621	23.58385	18.04408	12.54911	3.463362	1.435829
Croatia	1.203855	1.674279	2.682167	8.462227	15.01372	18.13044	18.40888	15.94138	16.38499	15.04714	18.0818	25.59447	28.27095	31.60426	28.13686	21.81236	20.41838	16.54898	6.973517	4.809436
Cuba	2.023267	2.548312	4.482188	14.68166	25.22124	29.40176	28.15835	25.5808	25.96784	28.74021	31.28351	34.0491	39.19521	47.32666	46.15425	43.45664	36.82905	27.39247	13.91255	9.436128
Cyprus	0.520408	0.988756	1.400736	8.950655	23.54259	16.7466	13.49777	12.06418	9.496243	10.16262	10.6397	19.77106	17.75618	26.95719	34.14158	24.47973	20.7702	6.775178	3.157896	1.29354
Czech Republic	1.252931	1.691217	3.980077	12.03885	22.95487	24.92408	23.0962	20.82244	18.25225	20.09613	22.26426	31.60702	36.95965	43.08753	42.57027	31.62912	29.22249	21.74229	11.74564	7.310105
Democratic Republic of the Congo	7.432561	4.38994	5.713579	10.57066	16.51986	18.53399	18.90922	18.1281	18.96512	23.1675	28.3665	34.60926	38.30896	41.88128	40.77238	29.68488	24.31326	17.60187	8.379325	3.349179
Denmark	1.011065	1.530073	2.179184	8.331809	14.23008	17.74516	16.34272	13.02488	12.27829	13.37118	17.64009	23.66051	29.47334	30.65717	37.4592	27.37045	26.26989	22.22131	11.56491	5.272469
Djibouti	12.7776	23.31471	25.33423	29.48869	30.40432	37.45088	35.04653	35.0009	32.20715	31.50397	33.97936	43.42447	45.1594	51.30554	52.81413	38.03193	33.19266	21.31501	5.004869	1.676935
Dominica	5.116908	4.04314	9.463053	17.56122	30.63607	25.18295	27.21871	20.50134	28.01804	22.36486	25.12204	26.69403	30.71268	33.63221	33.57862	28.82392	21.84091	16.25536	7.733517	3.807011
Dominican Republic	2.588109	3.836236	3.147926	5.876944	7.787701	6.12713	7.782676	7.144483	9.964868	10.14357	14.1556	17.39482	20.0431	20.29317	17.90493	15.00796	13.2281	12.66297	4.136712	4.155045
Ecuador	12.70832	8.845579	9.403631	14.20839	14.0309	16.45325	15.30998	13.37772	14.96257	13.74746	20.69723	26.15613	29.87086	32.12056	34.60069	40.35301	41.164	31.80401	29.24884	15.07769
Egypt	5.928845	18.20748	31.4764	49.79059	52.18302	54.76251	53.14364	51.16466	49.01449	46.86356	52.33608	60.16014	68.9726	88.4852	98.59689	73.21261	65.88169	43.86147	14.04904	5.377148
El Salvador	3.413423	4.344237	7.766567	13.07607	11.55523	12.18274	14.07203	15.78943	14.14296	18.0611	24.28568	33.19498	40.28668	43.54477	52.56342	42.0705	38.44746	30.04982	23.21932	11.54492
Equatorial Guinea	2.5193	2.664169	3.317874	6.420395	9.18677	9.959764	10.16403	9.618563	9.609765	11.42707	13.7953	17.57216	20.39149	22.85046	23.29848	17.61842	14.50138	10.5761	4.754093	1.850843
Eritrea	28.21556	35.13629	36.65403	54.25471	60.20838	79.64762	79.13703	81.83483	78.53939	69.03797	63.89854	70.51215	65.52721	88.55866	68.19637	50.78435	41.76929	24.63212	7.391009	1.988292
Estonia	1.154315	1.567346	2.038584	15.21423	22.48765	25.43971	23.19983	19.98027	17.61754	15.97592	18.24394	23.13753	24.56172	26.50116	22.92291	17.93217	18.31256	13.19555	8.676659	2.062833
Ethiopia	21.79082	35.71469	38.48544	49.3413	52.73736	64.86621	58.63946	55.27352	48.04071	47.55952	52.76294	67.6282	73.73571	89.07845	94.83023	69.66193	62.06186	41.6175	8.565384	3.270425
Federated States of Micronesia	1.20779	5.773356	3.230388	6.079448	11.37767	15.15085	18.66879	13.17143	12.04619	17.2133	20.35253	16.33901	20.08799	21.29527	25.17888	19.59962	13.58046	14.38118	11.57892	4.243876
Fiji	2.663451	10.33965	6.053064	6.740188	10.37118	12.54183	15.16641	13.52059	10.88223	14.98985	16.56235	10.44458	13.46425	13.95425	17.12014	15.63509	10.1601	13.28123	10.76103	2.505488
Finland	0.962146	1.419061	3.369227	9.820066	17.5406	18.75356	17.27744	15.3904	12.25541	12.41666	15.21339	18.06493	21.6511	24.17262	24.67637	21.08215	19.93027	17.17604	9.731046	2.905301
France	1.137482	1.093916	1.684257	6.595459	14.73789	20.81614	18.85083	15.44966	15.18307	16.11637	16.92654	21.78868	23.71786	25.32469	27.20526	18.33989	18.43166	16.01525	8.894162	4.276989
Gabon	3.612334	3.589023	4.770024	9.230791	12.64079	14.46316	14.97382	14.97691	15.74997	18.52103	23.10836	29.00245	32.15159	34.64807	32.76253	22.80014	19.02106	14.0683	6.579273	2.554054
Georgia	7.124136	5.132015	10.30226	14.90364	32.11207	31.06718	40.34485	39.58706	43.94521	55.59016	60.71954	81.341	68.34342	74.40348	67.77898	52.68961	24.1079	29.95641	13.24754	4.186426
Germany	1.143954	1.125765	2.123795	7.377382	11.84229	13.64898	13.97108	12.6092	11.87466	13.35085	15.97302	20.26937	23.12637	26.54659	29.70855	22.32675	21.02733	17.10419	8.561266	3.378645
Ghana	2.854973	1.249532	2.295286	3.276686	2.98873	3.274699	3.332109	3.524282	5.274891	4.155967	5.233637	8.350811	8.114745	9.823569	16.2113	8.996049	7.360855	6.966529	4.830634	3.628098
Greece	1.778907	3.172116	4.796603	16.16817	30.56394	33.24236	33.65846	33.0881	30.60922	37.57564	48.6683	57.87993	70.79181	80.7365	86.48255	75.32382	71.88313	61.05966	44.84417	22.37461
Greenland	0.683633	0.966771	1.251883	3.476342	6.611938	6.030731	8.988723	9.853101	10.07017	8.338563	11.03673	18.83755	21.35164	27.90116	30.41224	20.26941	16.45812	7.75066	3.769985	1.789969
Grenada	1.199997	1.747948	2.934306	5.089826	7.341553	13.42321	11.91401	12.53252	11.53444	11.60147	17.44616	18.40821	19.32708	20.18959	16.32346	13.27026	9.647012	8.193687	4.439123	3.253323
Guam	1.223994	3.027008	1.648091	4.403122	3.97792	9.523134	13.36621	10.79012	8.918751	10.22943	15.97706	9.132697	10.72562	11.03217	13.00353	9.794141	5.937012	7.003895	5.738788	0.79039
Guatemala	11.87334	7.535384	6.658305	7.44486	8.379165	8.200801	9.664405	10.55654	11.7437	15.54956	20.40936	22.03785	26.26543	32.34906	37.19144	25.07012	23.77255	21.56983	15.18458	18.72213
Guinea	20.07187	12.24474	16.29229	24.81577	25.95697	28.69928	26.88719	25.38493	26.07568	24.27516	27.73872	37.16608	38.59611	45.30887	53.14014	35.1634	26.22653	17.21702	4.844724	2.040213
Guinea-Bissau	8.929759	6.85831	10.2593	17.80033	18.54189	20.18674	20.43112	20.65534	24.85741	22.96502	25.88202	33.87186	34.83774	41.23498	48.06614	29.40444	23.24942	16.96447	5.882758	2.861313
Guyana	1.951612	2.7881	4.024911	10.04394	19.60198	21.77418	20.78172	18.98143	18.92407	19.72248	22.24213	25.28499	25.83969	26.62195	23.89901	20.16456	15.44669	11.0956	5.098795	3.288912
Haiti	8.700416	7.542162	8.988361	19.69692	34.27576	31.80767	34.53291	34.96271	39.65535	43.07626	56.67349	67.57272	76.82175	87.58697	80.33014	69.94157	53.94606	42.44295	19.6004	9.958126
Honduras	1.872603	1.696767	1.238562	1.432021	1.939821	1.993446	2.412961	2.822773	3.34028	5.794338	9.515279	13.73959	18.88042	20.30006	19.78916	7.513769	5.168786	3.765297	1.800967	0.789895
Hungary	1.147758	1.663506	3.678122	8.733132	13.17467	15.38933	13.50679	13.23367	12.63793	15.73964	21.21038	26.64921	27.16621	29.32208	25.67997	19.09003	15.41628	12.26336	5.122871	2.004998
Iceland	1.010348	1.026431	2.149551	6.201037	10.42769	11.76713	12.40775	9.789989	9.513647	10.0161	11.37364	13.11716	15.81359	19.51893	23.05974	17.14446	15.71125	11.82935	5.96236	2.35396
India	2.325443	7.860318	8.885638	12.53855	15.87663	18.43446	20.38602	19.58481	18.77275	18.04003	20.18474	26.99675	25.09247	28.96054	30.96196	23.79363	24.56968	18.53022	5.111472	1.70975
Indonesia	2.973448	3.667164	6.735424	11.78604	12.26683	13.89257	10.76616	12.01797	13.51003	17.24483	23.45771	26.5954	31.70407	40.04803	42.58387	23.0989	20.05373	13.04145	6.453578	3.358906
Iran	2.994321	5.435017	9.009878	15.23117	16.66512	16.25266	15.15279	13.96638	12.95519	13.67122	17.55506	20.08837	20.72741	27.06641	28.98093	24.59045	26.08783	20.55717	12.64459	8.499273
Iraq	2.558473	5.741351	6.027112	7.937311	8.397329	9.484968	10.11649	9.31406	8.113553	9.461988	11.0601	11.17795	13.21527	13.51462	13.64667	9.414668	9.158615	9.111821	5.386832	3.648686
Ireland	0.985928	1.117627	3.501731	8.875524	16.26981	21.53923	17.09711	15.27838	14.20973	15.05341	18.06514	21.68467	23.37144	27.42594	30.82845	24.09594	23.98751	17.97765	9.546497	4.077679
Israel	1.030932	1.872101	3.277235	11.21637	17.23896	23.28483	21.88345	20.12349	17.12355	15.34433	18.77332	22.24482	23.31838	27.44477	29.50393	25.46261	24.40505	19.54755	12.01572	6.287888
Italy	1.080523	1.951049	3.43571	11.11174	23.12021	27.13759	22.89505	19.91206	16.84978	17.72203	20.27933	23.42398	27.87417	31.46162	37.7573	29.47511	29.49958	22.58803	12.30444	5.383196
Jamaica	1.637531	1.138117	2.317485	5.842864	15.33479	11.57103	14.32926	14.55524	11.35084	8.429049	12.56871	16.51791	19.83964	20.44287	16.25698	14.95544	11.15761	8.091078	2.739045	1.149949
Japan	0.9798	0.938684	0.93823	1.474052	2.89284	3.22098	3.479446	3.151921	3.591291	3.835005	4.727583	5.788261	7.151112	8.62834	9.180196	7.178036	6.398635	4.998712	2.779936	1.251102
Jordan	0.865979	0.96938	1.169296	2.490518	4.316887	4.296189	4.81445	4.062658	3.822788	4.165348	4.645865	5.636595	6.660849	9.101774	8.820047	8.169305	7.07465	7.240578	3.438947	1.270807
Kazakhstan	2.106587	3.389032	5.783406	12.67777	21.39511	22.29252	27.96993	27.30345	21.78253	22.41046	22.85052	29.71911	27.66098	28.70623	21.76834	19.20943	11.07052	7.851965	3.811968	6.066646

Kenya	5.054542	10.55002	12.50862	18.25211	19.80043	24.26144	23.38412	22.79434	21.07966	20.18112	21.8837	27.40218	28.00213	32.61721	34.21623	23.98539	20.20296	12.96339	3.135767	1.072081
Kiribati	1.558922	6.226691	3.033128	4.709288	8.177279	11.03812	13.54192	10.84951	8.580213	10.62887	11.97768	10.4672	12.62786	11.47709	17.78227	12.0819	9.077125	8.791462	6.855501	2.860585
Kuwait	0.847225	1.795845	2.237338	3.768913	3.625585	5.251563	5.125727	4.453784	5.433971	6.101473	7.599216	8.941891	12.15289	13.29529	9.771227	9.066819	5.420533	12.94961	15.03235	1.449674
Kyrgyzstan	1.471312	3.667446	5.515799	6.662478	7.518022	7.437041	9.361277	11.81463	11.05428	11.16388	10.97399	24.09333	18.25073	21.10395	16.18595	15.95444	7.114492	3.848137	1.551048	0.731989
Laos	7.2702	7.44267	11.02327	15.89296	17.82682	22.00027	17.62589	19.01915	20.11694	25.27773	34.27339	37.69741	43.62616	52.4697	56.65495	27.82546	23.42307	14.55466	6.725673	3.477364
Latvia	1.041847	1.528746	3.651039	12.72954	26.64162	33.48007	35.44179	29.62965	23.66131	24.52041	25.31393	29.61568	34.04461	29.65275	27.0978	19.51433	12.44238	8.072258	3.8422	1.031543
Lebanon	2.513038	11.83479	22.74973	64.22924	73.27085	69.68339	59.68239	48.03364	41.99548	39.49527	42.59431	50.32475	54.25657	70.69009	75.95599	62.77925	60.62823	42.87697	16.81442	7.25095
Lesotho	2.324002	8.028324	8.114984	13.43905	13.64679	20.84788	25.58743	30.74092	33.5574	38.7666	37.31674	47.14659	50.35912	43.35867	45.40877	24.80647	30.1086	22.2371	14.29946	2.903328
Liberia	5.705149	3.52998	5.898972	10.38259	11.07204	11.72799	11.21588	11.50567	13.07252	12.93779	15.80744	21.26205	22.55934	26.13369	36.09198	21.21438	17.77826	14.41267	4.084251	2.141547
Libya	6.21596	17.81726	31.88514	64.20262	84.03885	83.23197	84.95649	66.69612	61.93748	57.70998	56.11803	64.22722	77.30285	93.3107	97.60091	74.79716	74.66416	51.67767	22.74019	13.18384
Lithuania	1.279449	1.945429	5.677393	11.80667	19.74799	27.43875	27.78102	25.51982	20.54718	19.81664	21.0521	25.74996	29.44837	29.70613	26.43827	19.40419	15.50654	11.10477	6.007585	2.713044
Luxembourg	0.884984	0.919805	1.610969	7.397813	13.73505	15.15816	12.75057	14.65141	10.39873	11.92665	14.80122	16.7846	20.21347	23.38222	26.18969	19.811	18.11515	14.38886	9.336803	4.477522
Macedonia	1.178341	3.488025	3.953595	14.59299	26.17098	31.42554	29.19328	22.12177	25.26967	25.76475	33.93472	33.20418	37.76724	31.73243	34.69119	23.236	13.33387	9.556538	2.306782	0.414782
Madagascar	13.48878	19.52003	21.89966	31.43318	35.94618	45.84779	41.97933	40.46158	36.20637	33.96809	36.50648	44.79333	43.36966	48.18618	47.90383	34.19705	29.3864	19.02041	4.57808	1.805011
Malawi	11.86577	13.93858	14.74502	19.38471	21.8182	25.92036	23.69251	22.61917	20.36324	19.6955	19.8695	24.08742	24.34249	28.10258	29.47287	21.60129	17.86643	11.15236	2.548398	0.941478
Malaysia	0.756068	2.136946	4.818144	10.42439	11.24404	13.55136	10.75725	11.03537	10.41263	12.08163	15.98192	17.30379	20.1692	25.10705	27.50345	17.46176	15.36602	8.419032	4.230003	2.47382
Maldives	1.074672	0.899717	1.134746	1.313606	1.964945	2.692653	2.609669	3.353962	3.181657	3.637307	4.555149	5.5937	7.580106	9.592517	9.769415	4.676958	4.273193	3.599814	2.032898	1.046778
Mali	34.00385	15.71683	16.68663	20.50991	20.76075	23.63753	22.33116	21.70926	21.54758	19.83223	22.21351	29.02472	29.68276	35.07395	42.5682	28.96921	23.04687	17.11143	4.246776	1.966395
Malta	1.140943	2.102869	4.730791	11.6436	25.18512	27.85659	30.31134	24.50123	20.18728	19.06924	22.08514	27.40267	31.76893	38.52269	45.3926	36.04532	34.26673	26.16556	13.86272	5.035866
Marshall Islands	1.181032	5.166704	4.000482	7.392212	13.91661	17.51061	18.99182	16.25332	16.06931	20.11397	21.81495	20.2779	25.42917	28.817	27.72275	22.36104	13.98239	15.33528	10.98959	3.473209
Mauritania	4.213147	4.331227	6.055496	9.195728	8.546372	9.085363	9.126722	9.057421	10.73632	10.2235	12.63766	17.28938	19.33697	24.35164	30.70046	19.69281	16.12481	12.62591	3.931608	1.809333
Mauritius	1.682244	2.175294	4.995528	10.80543	11.43347	20.34412	8.793966	8.7535	11.76198	12.46254	17.39354	12.37104	16.3352	21.81024	21.4492	9.552622	7.127586	4.406115	2.576755	2.533664
Mexico	5.624149	6.026184	7.039414	11.00838	15.15916	14.52052	14.18698	14.64552	14.99222	18.93845	23.03197	30.35039	35.43282	42.00278	47.20795	44.65744	41.52397	32.18303	17.64892	9.584701
Moldova	5.058718	8.013768	12.81027	26.45691	36.83811	41.55786	35.10667	33.42667	31.06308	35.28003	35.9984	39.7371	45.54084	48.74304	38.28802	20.21147	16.09621	9.080255	3.635556	3.056428
Mongolia	0.806832	1.378921	2.210695	3.270838	4.770266	5.245058	6.616013	7.610321	7.18049	9.910869	10.8829	12.35413	14.00325	15.93867	14.68188	7.640837	3.890511	3.403154	1.937153	1.120582
Montenegro	2.162618	5.650658	10.58097	18.20383	38.73291	41.91744	37.06479	33.0644	29.92338	35.66571	43.27003	58.94203	63.85434	66.43852	66.98368	46.43358	35.33879	27.58188	11.31274	5.381074
Morocco	5.495313	25.66148	35.70119	55.35401	60.51733	60.51627	61.80135	49.71284	41.79283	39.11981	41.72579	49.71572	54.10536	69.89955	77.09935	60.42069	58.17428	40.31066	16.44042	7.731161
Mozambique	21.31914	31.28402	30.89769	43.70358	45.55751	57.07427	54.18177	53.71399	50.29955	45.9587	46.24032	53.93706	52.77344	59.14009	60.1519	45.88706	39.71049	25.74466	6.082387	2.013562
Myanmar	5.973506	5.70411	9.227596	15.7607	18.12156	23.30133	18.61437	20.40946	21.82531	27.27674	36.52725	38.56966	44.14024	53.60952	55.04558	30.70411	25.20592	15.14518	7.138805	3.560427
Namibia	3.170265	9.337233	11.5104	20.07759	17.26523	22.07415	22.42874	22.81692	22.85747	25.16769	26.22797	33.49883	37.21855	36.41149	42.58119	25.76975	27.19039	18.33415	9.226132	1.730425
Nepal	2.310135	9.489097	11.09784	18.12785	19.10054	19.69705	18.08419	17.06867	16.95959	18.29886	20.92961	28.75867	30.54904	37.09084	39.14973	32.64411	30.46402	20.82493	5.364617	1.609903
Netherlands	1.015071	0.866672	1.533334	7.519909	14.87246	17.36814	17.98566	16.26297	12.15383	13.24217	15.59604	20.55426	24.92418	28.56248	32.38981	24.71892	24.83454	22.31184	17.61852	8.833189
New Zealand	0.955281	2.199579	2.29041	6.922124	11.66695	20.30594	14.5022	11.47894	11.89129	11.38334	10.71692	13.93355	16.47652	19.02647	22.71489	20.48272	21.72736	17.22216	7.466031	3.689021
Nicaragua	1.967262	3.427847	4.207009	6.031535	8.236632	6.956093	6.68776	5.897821	7.4842	10.87019	11.58644	15.75983	21.16268	21.09872	22.0818	20.67285	19.47808	17.10255	5.94329	4.182842
Niger	9.972637	4.304375	6.315102	11.99792	12.25995	13.29982	13.06296	13.63949	15.09514	14.58111	17.56895	24.96106	26.38953	30.59521	36.96615	24.14069	19.72563	14.83133	4.562548	2.16602
Nigeria	72.15391	54.78741	61.83672	70.27837	73.07953	82.27806	70.49053	62.21913	63.00469	54.50494	63.71397	82.18417	89.77025	110.9962	141.5346	103.5406	87.08843	65.6637	20.64307	7.543279
North Korea	5.976462	4.220607	3.799083	7.404184	9.630722	8.028476	9.296838	8.527988	9.842231	12.5878	16.16188	20.34789	22.1618	23.65333	20.61837	16.43977	12.58808	8.342161	4.215294	2.496787
Northern Mariana Islands	0.827832	1.2316	0.952453	1.735904	3.110213	3.530463	4.709809	4.803009	3.206365	6.059673	5.151409	6.456177	6.921166	9.873829	9.918491	9.086549	5.20934	5.880058	3.858353	0.933184
Norway	0.822059	0.901514	2.457213	7.612805	11.29091	13.72216	13.03186	11.37064	9.528872	10.30508	12.33499	15.09684	17.69868	21.76434	24.7259	18.80425	18.30679	15.10564	8.220144	3.168003
Oman	2.082881	5.91465	9.92815	16.83458	20.3886	18.04906	17.28435	14.48255	14.9959	17.17745	20.78328	28.16113	30.5353	37.35357	37.0701	27.93272	26.38213	17.97993	6.271751	2.249767
Pakistan	15.49519	69.68612	75.30237	92.7205	95.93841	98.62641	71.7622	70.15426	55.50468	49.11529	58.74248	65.5539	78.88579	81.11762	88.13395	63.83292	66.85922	53.39596	14.54747	6.152146
Palestine	1.690729	3.2672	5.403194	10.50386	14.37591	22.64606	18.708	16.52441	16.60731	23.75949	24.04939	24.48935	26.9067	32.44063	34.47109	28.1561	20.99849	16.1452	9.022866	1.873136
Panama	5.632708	6.515683	5.825868	9.302619	6.44263	9.329947	10.68788	7.869715	6.846623	8.420508	11.8844	14.80955	17.04294	20.90708	22.05245	20.02362	16.62961	14.22694	3.813669	1.472153
Papua New Guinea	2.292191	10.53867	5.648395	9.495411	18.12951	24.14428	34.21126	27.19203	24.68303	29.73532	34.30692	25.07264	28.78398	31.10184	32.847	25.08586	14.0684	13.67254	9.186729	2.798405
Paraguay	0.869418	4.973222	8.002108	12.68452	12.38892	12.75193	16.05947	12.89384	13.46802	17.91569	21.63447	28.31202	30.65636	35.80233	37.23686	23.22526	18.68222	14.08766	7.746143	1.610013
Peru	1.664553	3.526533	3.704621	4.69006	5.354472	5.199887	5.545812	5.811388	6.153437	8.506902	11.84252	14.29517	16.75703	20.55908	21.27497	19.78072	17.4696	11.79042	7.352867	2.274258
Philippines	2.52293	2.306348	4.239253	5.822642	5.92296	8.439974	6.104221	6.63736	6.901669	9.478672	11.60421	12.00361	14.91571	18.3397	19.78272	9.889732	9.168083	7.342347	5.055054	3.067835
Poland	1.198258	2.4637	3.902699	11.0035	19.52598	26.19086	24.21316	21.90601	21.18434	21.88064	25.93404	33.28264	36.28127	40.07213	38.37561	23.37403	17.52418	11.82724	6.033914	2.527095
Portugal	1.339528	0.90976	1.975545	6.616307	13.12184	15.67852	13.79594	15.49504	14.28886	16.18501	20.05725	21.63705	23.53893	26.29183	27.07812	21.00231	16.19261	16.31242	9.571117	4.298727
Puerto Rico	1.190372	0.948683	2.553992	12.15064	26.72206	20.63345	25.36313	21.66773	18.37555	16.3863	20.38429	23.11036	25.96634	27.73978	26.47311	25.67086	21.18301	17.76646	6.169861	1.394327
Qatar	0.659914	2.056147	1.637796	3.90205	12.74328	4.777521	3.732927	2.925512	3.738292	3.772695	6.992992	11.43604	13.26187	14.93502	13.12885	7.888928	6.88605	5.710854	3.300451	1.881568
Romania	1.900848	3.755419	6.170585	12.52206	18.89708	22.45152	22.70495	20.51911	20.37208	23.47212	27.74014	34.57021	31.97144	32.07666	26.73472	19.01989	13.03238	8.785446	3.898032	1.333953
Russian Federation	1.620824	3.309574	6.00315	14.47055	27.53931	35.66049	37.31704	36.67942	28.71291	24.72874	24.27081	27.3137	28.20258	27.90056	26.75511	18.76157	14.53939	8.37801	4.145762	1.709907
Rwanda	16.02991	18.91781	20.55394	26.70792	28.05343	33.38	30.62828	29.61685	26.43853	25.83082	28.48322	35.92922	37.60272	43.75086	45.79356	35.11849	30.86623	20.16349	4.99864	1.596582
Saint Lucia	1.852379	2.75758	4.973809	10.56643	20.22559	24.29902	26.30429	21.02899	23.36001	22.4268	27.62543	31.62606	30.37068	32.7458	30.43625	26.90578	19.4112	15.95004	8.978445	6.288139
Saint Vincent and the Grenadines	2.315976	4.076757	8.054686	16.67064	25.59747	26.02904	35.41266	26.99478	29.07965	29.16778	31.94144	32.1163	36.08837	40.85468	37.43351	33.46743	21.0815	13.69945	6.751005	4.52315
Samoa	1.522792	6.317733	6.020474	12.38574	17.21429	17.47407	20.08422	14.79396	13.72362	17.95453	21.14267	20.18417	25.08633	28.59786	32.18728	24.84289	16.21366	15.12694	9.353761	2.96576
Sao Tome and Principe	1.97707	1.420259	1.276057	2.276835	2.499597	2.402514	2.132783	2.21621	2.413142	2.674445	3.569214	4.972796	5.40357	6.174886	6.864586	4.239773	3.459731	3.348316	2.482391	3.03057
Saudi Arabia	1.802275	3.815533	7.244777	23.26058	24.75147	23.91984	19.13458	18.58645	19.31482	21.23222	24.71414	29.08358	29.24453	36.75697	41.79087	35.76652	33.73057	24.84925	8.177278	2.927251
Senegal	6.740192	4.775146	7.280121	13.68071	12.83504	13.18637	12.58891	13.12497	16.05702	15.36889	19.18706	27.29288	29.05689	35.46595	42.4184	26.9684	21.83769	15.99918	4.863839	2.25377
Serbia	0.86321	1.940075	4.036466	11.19922	21.97993	35.53915	32.88294	27.64198	27.87961	31.06027	34.80471	42.23657	46.80236	45.67319	45.17148	33.91358	24.58824	17.90962	7.98628	2.476293
Seychelles	2.636193	2.980253	4.145811	9.122135	12.36149	15.48106	14.17702	14.59592	16.35432	20.49488	26.06346	27.89943	33.01128	41.9522	39.06089	31.19335	27.63493	15.60718	11.25906	7.036458
Sierra Leone	9.25667	4.338675	6.711537	13.17548	13.70299	14.42659	13.55908	13.63497	15.34071	14.38084	16.44295	22.30087	23.39683	28.18941	35.75123	23.41935	19.46907	14.73261	4.579554	2.27702
Singapore	0.620379	0.926943	0.950504	2.808635	5.238654	5.271228	4.430755	3.542868	3.845851	4.010438	5.199163	7.07272	8.154272	10.01377	10.89346	7.722215	6.461951	4.749437	2.799267	1.106396
Slovakia	1.503236	2.34193	3.486209	12.59197	26.59624	29.82487	28.55273	20.88934	21.0535	21.71264	26.33575	31.211	36.04181	40.22884	37.92638	27.30805	23.1995	18.38116	7.951877	2.670732
Slovenia	0.974925	1.265089	5.236515	11.24821	16.59519	14.55515	14.01665	13.85675	12.87176	14.0935	17.72117	22.98131	25.86377	29.30801	28.99279	23.13	22.5398	19.11778	9.92159	3.522687
Solomon Islands	1.348292	6.498594	4.123109	7.484542	13.82538	16.76788	22.58567	17.8127	17.24883	21.90502	25.74644	20.30357	24.04713	25.04559	29.36974	23.5065	14.60652	15.46202	10.8376	2.853321
Somalia	30.56343	37.61715	40.32801	50.42469	55.61652	70.76381	66.00982	64.20394	60.65099	57.64509	61.45881	73.47979	73.21071	83.46949	84.09749	60.43695	50.12926	32.92158	7.789381	3.136812
South Africa	1.893974	2.161212	1.558872	3.793242	5.850703	9.278226	11.05637	11.65111	11.77195	13.72752	14.78327	19.90889	21.47163	18.72092	18.97918	9.847474	12.62122	10.12838	7.239408	1.437913
South Korea	1.023451	0.989218	0.958431	1.73697	2.82278	2.912886	3.198324	2.655029	3.15299	3.163354	4.133095	5.097468	5.908675	7.957844	8.770017	7.138319	6.11264	4.979151	3.209527	1.008081
South Sudan	26.21064	27.23683	27.85207	34.98672	39.43167	49.4561	45.98663	45.79683	42.70194	40.63439	43.93244	55.6269	58.10573	60.14108	63.99699	46.28306	39.87658	26.82784	5.544811	2.109515
Spain	1.069445	1.332483	2.501476	8.05468	13.8206	17.0908	16.39229	14.21855	12.56211	15.0972	17.68714	21.0208	24.31257	26.12862	28.82321	21.37856	24.77841	18.91049	10.93466	5.039445
Sri Lanka	1.659714	1.978501	3.871341	7.134693	8.255031	10.20961	9.311029	10.87999	13.43323	17.15172	22.41556	24.35334	28.5298	34.69384	39.76268	22.64279	21.7665	15.53549	8.460268	4.362758
Sudan	6.371803	11.32461	14.40513	19.72275	23.69279	23.27751	22.64853	20.66461	19.9103	21.79604	27.3519	34.94154	37.33103	48.65714	53.1683	38.97027	36.93968	27.33227	11.62346	5.532027
Suriname	3.676782	3.287669	7.010498	16.82492	23.19408	28.39417	28.97179	27.09953	25.75328	24.66455	30.95337	34.79144	35.54764	40.75918	34.65231	32.53163	25.6654	19.8874	8.465425	4.739519
Swaziland	2.11579	6.099288	6.426728	11.55155	11.5461	17.60863	21.38953	25.65556	28.9138	33.16397	30.64817	38.53641	39.93569	35.1445	35.71331	18.71276	22.73859	16.53	10.69212	2.254633
Sweden	1.097427	1.282911	1.749334	5.401341	10.50945	13.09338	11.41006	9.966307	8.147148	8.266624	8.84559	11.86951	14.79987	18.31393	20.63412	17.04262	18.15177	15.40257	8.354076	3.229065
Switzerland	0.987668	1.104224	1.988115	6.203283	10.56862	11.76645	11.75228	9.126162	8.373564	10.27315	10.78667	13.57888	16.41629	18.97607	24.64518	17.96054	18.65079	14.51888	7.385964	2.081896
Syria	0.719056	0.689593	0.973505	1.808329	2.34734	2.839224	2.614609	2.585459	3.044171	3.330914	4.633752	6.454333	6.893924	8.696344	9.109866	6.535971	4.907392	4.079272	1.77112	0.705135
Taiwan	1.229066	1.212241	1.452858	2.672837	2.988911	2.83154	4.054871	3.655136	3.515336	5.015344	5.60351	7.172556	7.019918	7.766775	8.518737	6.547677	5.63104	5.119481	3.538835	0.64481
Tajikistan	1.39325	1.623418	1.87378	3.736412	6.334884	5.889733	7.803518	11.121	10.34136	9.03997	14.18786	18.00745	19.15121	20.10982	23.25298	11.63854	7.194857	6.367083	2.697916	0.97669
Tanzania	23.1267	24.14346	25.11024	28.54358	29.75029	36.90216	34.1355	32.56894	27.85674	27.13814	29.93428	37.60521	39.12675	45.33842	47.5415	35.85426	30.88922	20.2659	4.703903	1.595578
Thailand	2.561176	1.962065	2.658983	4.930033	5.523622	7.780077	7.359622	7.378513	7.020749	9.590744	11.87845	13.16595	14.82408	18.37772	19.11986	8.975181	7.517578	5.038424	2.273205	0.922616
The Bahamas	1.651188	1.901369	2.966111	8.683995	18.41901	22.60315	23.17391	19.4995	16.62342	16.15843	17.7323	19.73251	19.11176	23.54304	21.0625	19.49262	15.8295	12.58761	5.797121	3.600909
The Gambia	8.951526	7.648677	10.11383	16.39199	16.87581	18.52529	17.90188	17.28907	18.32469	17.17392	19.962	26.60063	26.80355	32.84852	38.75592	24.36567	18.43841	12.05882	3.466143	1.388655
Timor-Leste	4.478524	5.744622	9.220193	14.36228	14.80422	16.52336	12.96237	14.52816	16.78847	22.08627	32.2892	36.3648	42.84006	49.6992	50.97466	28.93678	24.81015	16.84633	7.159736	3.764344
Togo	5.179795	3.318135	5.6074	11.81289	12.26176	13.17683	12.81334	13.19403	16.09192	15.55641	18.27047	24.43522	24.58588	28.9574	31.7682	21.14221	18.12782	12.86574	4.369323	1.873325
Tonga	1.270138	2.130191	1.740779	4.84128	7.554986	8.58046	9.898024	8.648787	7.04545	10.11488	12.46457	10.42123	12.55333	14.59304	17.09215	13.50177	9.171279	10.01042	7.98358	2.570965
Trinidad and Tobago	1.826565	1.403241	2.663263	9.631048	17.74425	20.59431	17.49283	17.2237	12.16813	16.7937	19.1949	18.05445	15.33144	16.4837	13.80949	21.56447	13.774	5.042899	1.831806	0.733473
Tunisia	3.326963	9.200665	15.05176	22.43988	25.7222	28.70923	29.59506	24.83604	22.88106	22.62521	25.95298	34.01524	37.54025	49.95667	52.46027	41.22502	45.82784	36.75797	13.8102	5.74587
Turkey	2.193697	3.807645	6.259872	10.87517	15.02107	14.84305	13.40993	10.26827	10.656	11.70458	14.3701	17.53959	19.85429	25.52787	28.13403	19.47562	14.71934	9.308815	3.202125	1.165639
Turkmenistan	3.985049	3.714974	9.376377	18.54721	34.16428	35.47834	30.63745	31.20874	24.99142	23.75492	20.16592	29.11678	24.97471	28.36027	19.25957	14.08167	7.711294	7.078623	3.565465	1.823372

Uganda	20.11449	24.23731	25.53765	34.9924	37.52114	44.76416	41.86065	38.93895	35.0417	34.20217	36.10946	45.15593	47.2382	53.86939	56.77924	44.77508	39.19738	24.76922	6.26015	1.953487
Ukraine	3.307625	5.402368	11.73003	44.89219	77.72212	88.61064	80.98143	69.67493	54.6409	47.79134	42.28111	43.42025	40.67864	38.50259	32.1447	19.59962	11.37361	7.145977	3.389021	1.340194
United Arab Emirates	2.864666	4.414721	9.220848	21.67624	18.50327	17.48985	15.46928	14.931	16.80471	18.46204	24.15085	32.63696	42.10415	46.12738	36.21212	21.23769	19.8268	15.92911	7.223276	3.767754
United Kingdom	0.986803	0.952472	1.986018	9.03264	17.61119	22.19993	20.08974	19.16467	16.86036	16.77002	17.77341	22.4095	26.46398	31.96768	34.09768	25.24827	22.69586	18.18116	10.4315	4.76234
United States	1.004773	0.967331	1.711263	6.241107	16.1343	21.38862	21.27777	17.46966	14.24858	14.0275	14.77077	16.62165	18.03416	20.61519	23.52613	24.71957	23.53385	18.94304	10.6434	4.172818
Uruguay	1.074744	2.092374	4.423573	13.37962	23.70352	23.1888	25.70605	19.76596	21.35276	20.74425	28.0208	34.09057	42.4825	49.4109	48.2893	22.23567	21.61903	18.96206	5.522295	1.151136
Uzbekistan	5.796242	11.11874	12.86592	14.47618	19.01408	19.28534	17.45694	19.47852	21.65275	23.80083	27.65037	34.71701	34.53329	34.87889	29.75304	22.07216	12.49436	10.41334	5.265396	2.698211
Vanuatu	1.561699	10.17593	5.589966	11.21738	23.8956	28.86888	39.87017	31.90345	30.0725	36.80231	44.11182	34.16922	40.65137	43.21648	47.51581	37.77468	21.95596	22.26821	15.43571	4.706714
Venezuela	3.044867	4.473407	6.591888	17.96629	21.89952	23.70668	20.40549	17.34598	16.12324	21.14893	26.60786	32.08393	40.39997	38.99979	42.82747	33.48468	32.23401	21.03514	14.09402	6.997093
Vietnam	2.023806	2.796274	5.876624	13.60491	13.99242	15.16046	11.61272	12.59982	13.90867	17.63849	22.2151	24.60734	26.37024	31.54054	33.94539	19.31301	17.00425	10.53493	4.038098	1.621235
Virgin Islands, U.S.	1.930651	1.764778	1.573568	3.945772	6.134846	6.413716	7.433801	8.447289	10.07223	12.27945	15.58694	17.08194	18.45909	21.1056	19.4701	16.27663	11.75831	8.687994	3.912976	2.338156
Yemen	3.487881	10.72188	14.79305	22.76408	27.43367	28.55569	28.27944	26.73709	27.52003	29.47028	35.63488	43.11387	45.15943	62.18253	60.02481	46.12854	43.01293	29.92241	12.22165	5.666202
Zambia	21.10793	24.82322	26.14667	34.46836	38.40621	46.25236	43.45521	42.08272	39.61716	37.35413	39.53458	47.45996	48.91705	56.43344	57.22542	43.72252	37.86568	24.95958	5.986365	2.164202
Zimbabwe	3.661336	13.18265	15.01249	23.616	21.69967	29.40617	31.55361	34.17588	31.58125	33.29341	31.87733	36.03028	39.85674	37.63721	43.05468	24.76458	23.02706	14.85731	7.641904	1.683757

Sup Figure 1 The incident cases (A), age standardized incidence (B), death (C) and DALY (D) rates of Hodgkin lymphoma between 1990 and 2017 among sexes.

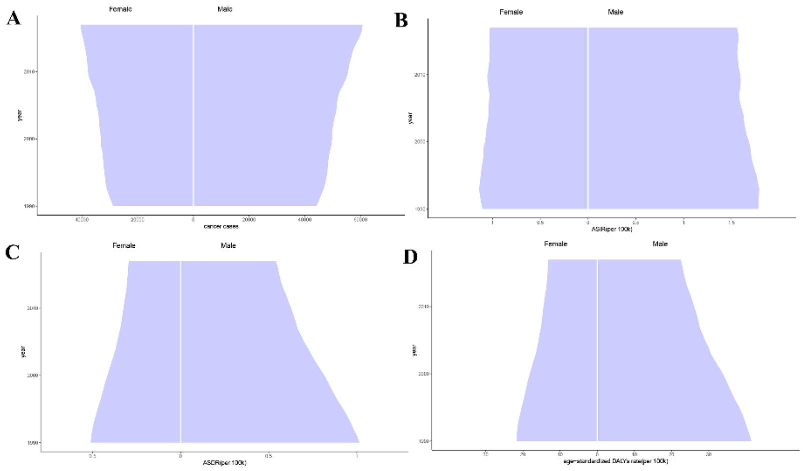

Sup Figure 2 The EAPC of Hodgkin lymphoma ASR from 1990 to 2017, by sex and region. A: The EAPC of ASIR; B: The EAPC of ASDR; C: The EAPC of age-standardized DALY rate.

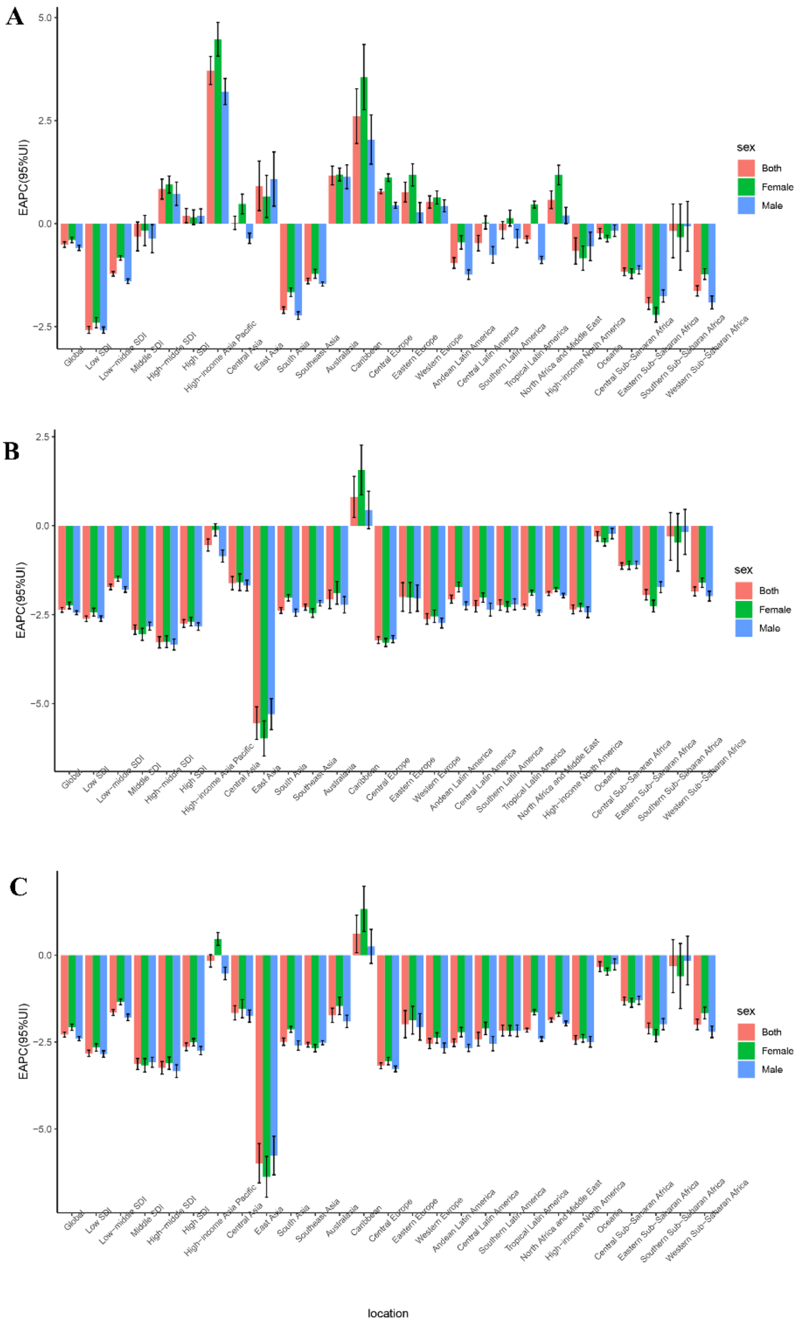

Sup Figure 3 The age standardized incidence (A), death (B) and DALY (C) rates of Hodgkin lymphoma per 100,000 population among regions based on SDI in 2017.

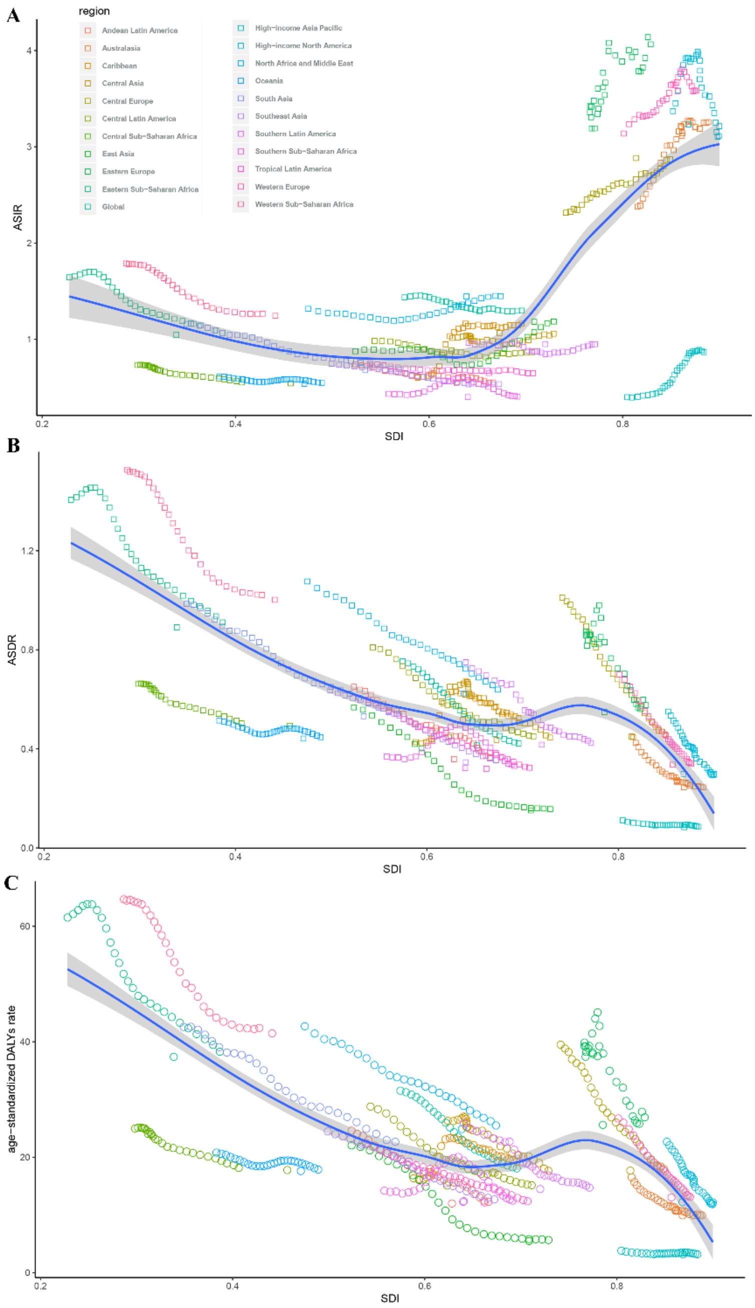

Sup Figure 4 The proportion of different ages in Hodgkin lymphoma incidence (A) and death (B) by years.

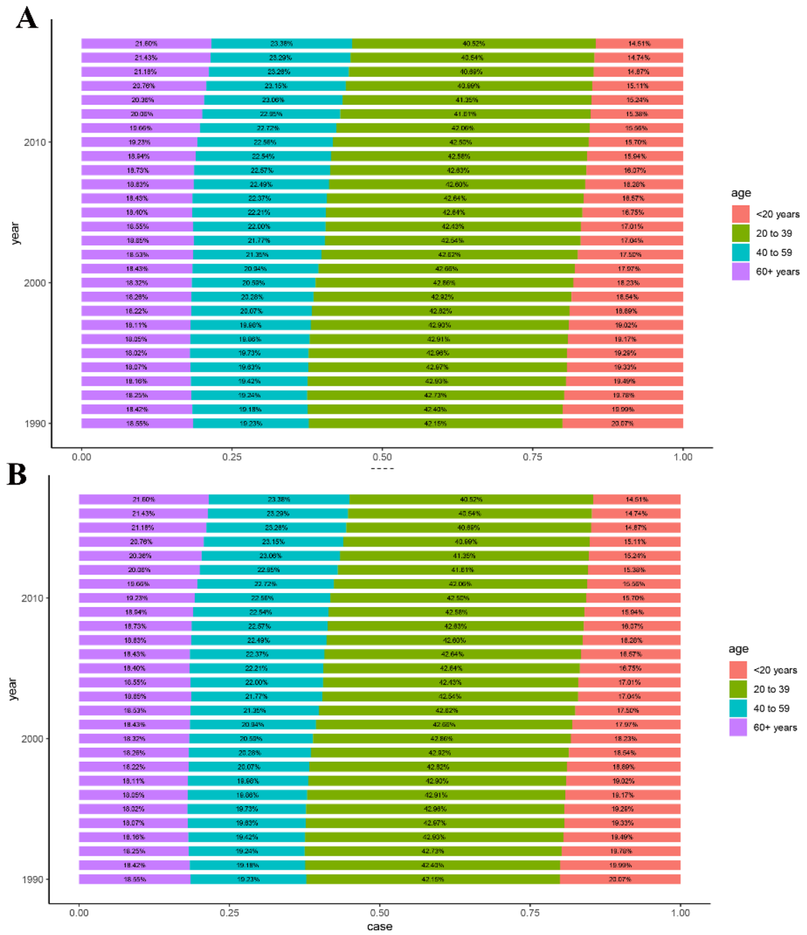

Sup Figure 5 Distribution of different ages in Hodgkin lymphoma incidence in global (A), high SDI (B), high-middle SDI (C), middle SDI (D), middle-low SDI (E), low SDI (F). SDI, socio-demographic index.

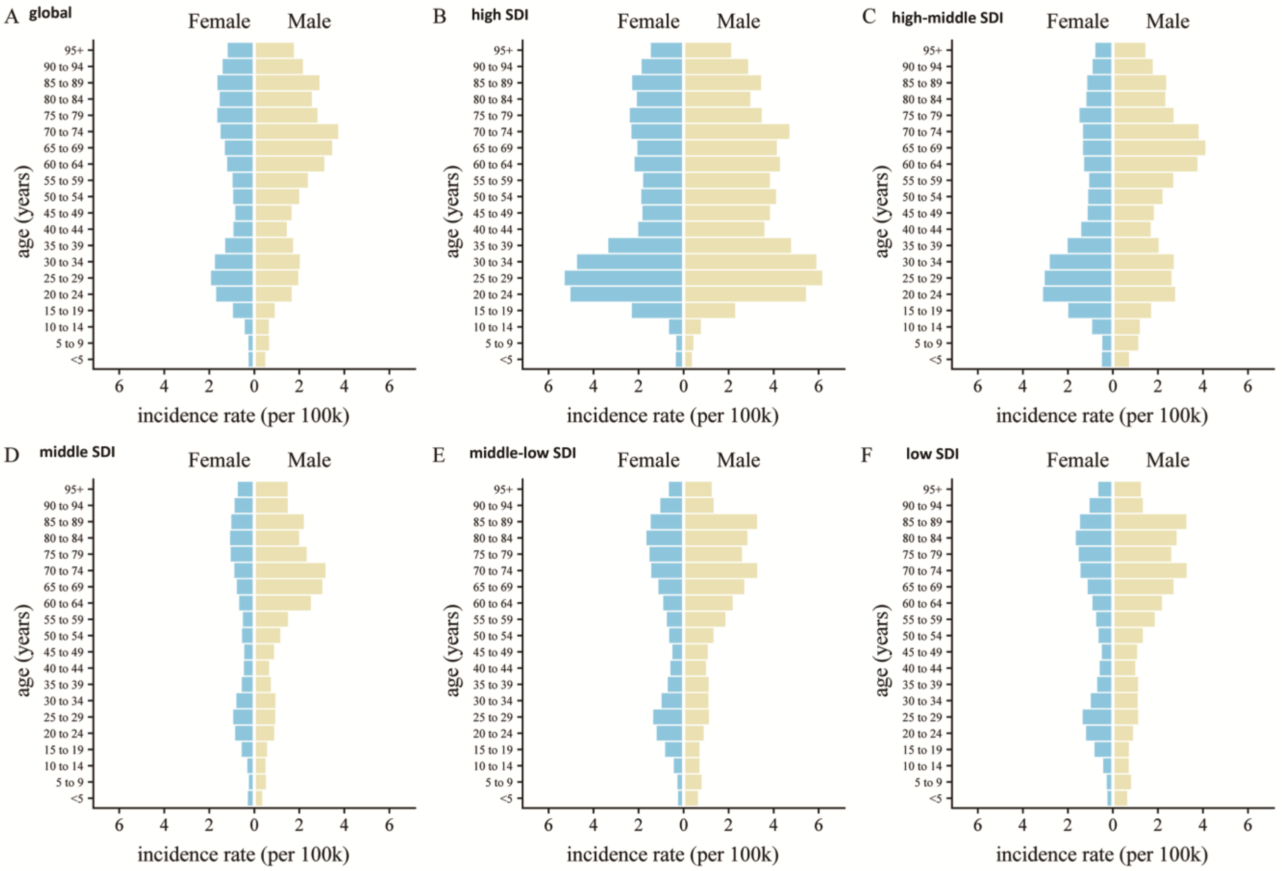

Sup Figure 6 The global EAPC of Hodgkin lymphoma for both sexes in 194 countries. A: The EAPC of ASIR; B: The EAPC of ASDR; C: The EAPC of age-standardized DALY rate

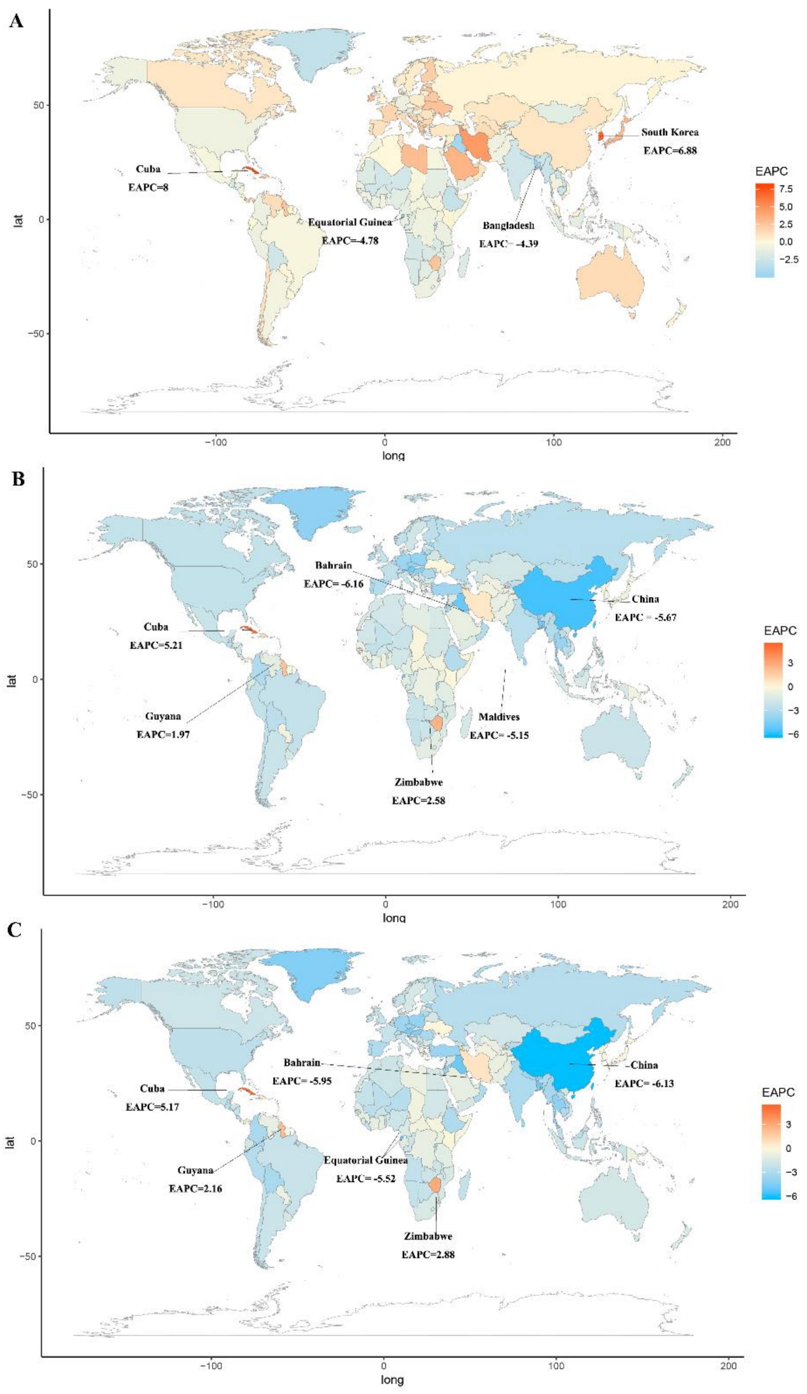

Sup Figure 7 The global EAPC of Hodgkin lymphoma for female in 194 countries. A: The EAPC of ASIR; B: The EAPC of ASDR; C: The EAPC of age-standardized DALY rate.

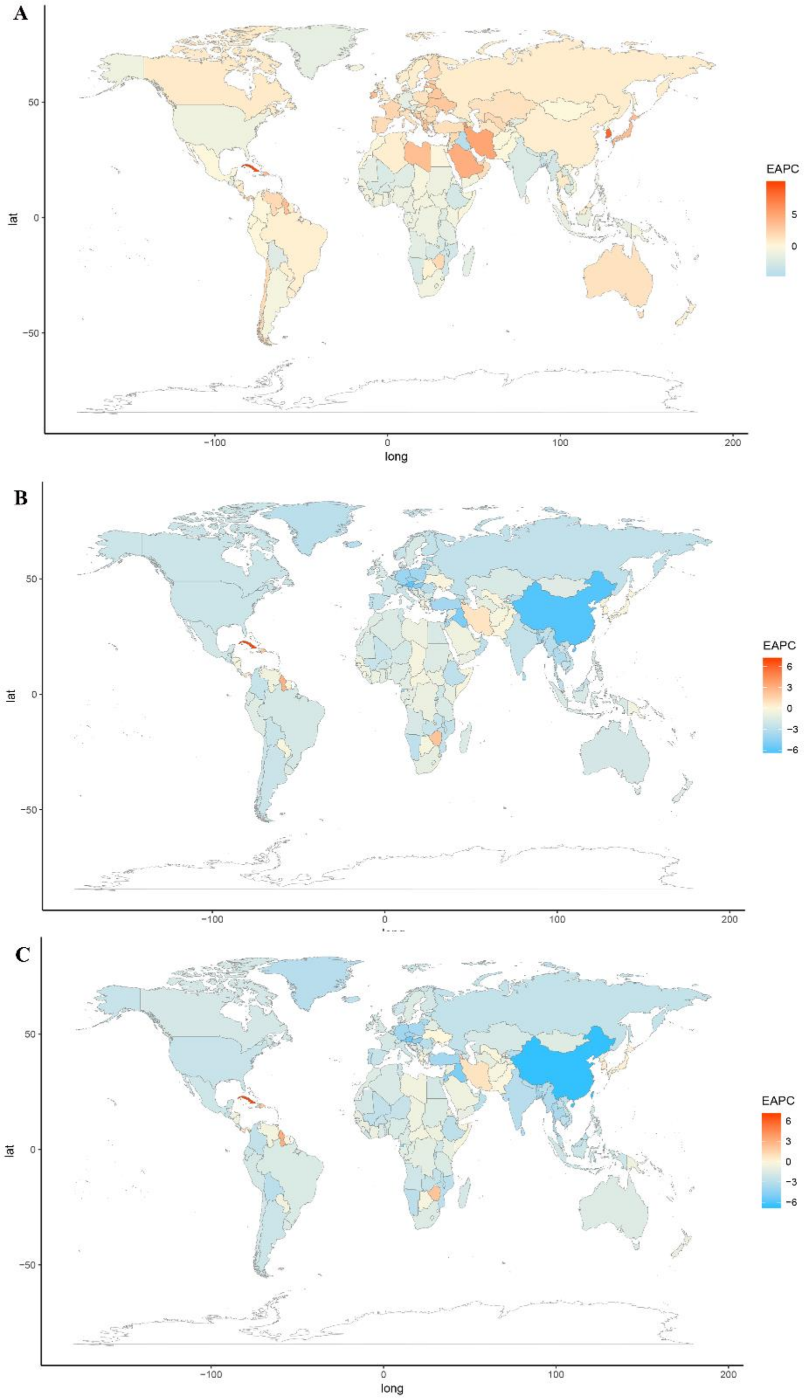

Sup Figure 8 The global EAPC of Hodgkin lymphoma for male in 194 countries. A: The EAPC of ASIR; B: The EAPC of ASDR; C: The EAPC of age-standardized DALY rate.

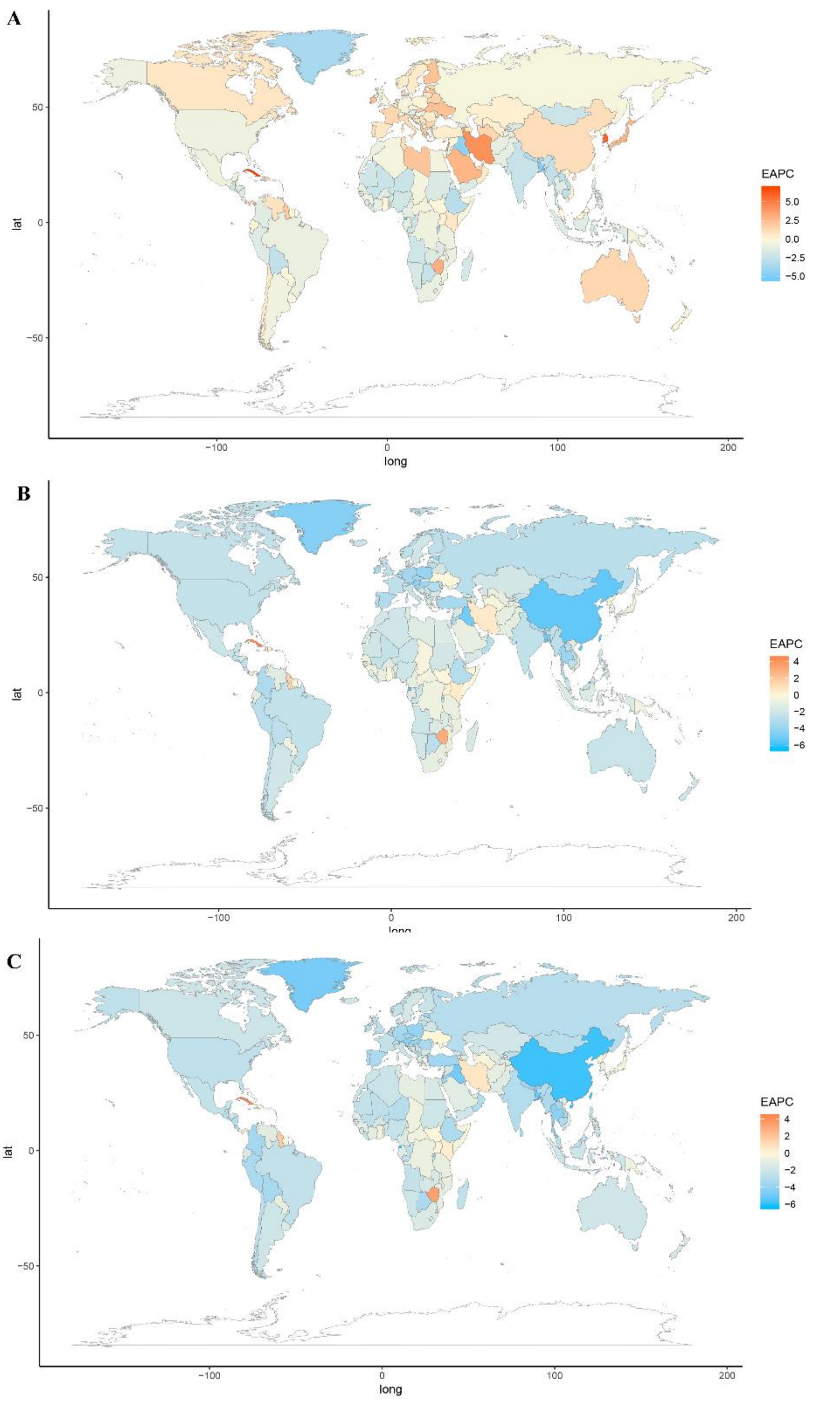

Sup Figure 9 The global disease burden of Hodgkin lymphoma for female in 194 countries. (A) The ASIR of Hodgkin lymphoma in 2017; (B) The ASDR of Hodgkin lymphoma in 2017; (C) The age standardized DALY rate of Hodgkin lymphoma in 2017. ASIR: age-standardized incidence rate; ASDR: age-standardized death rate.

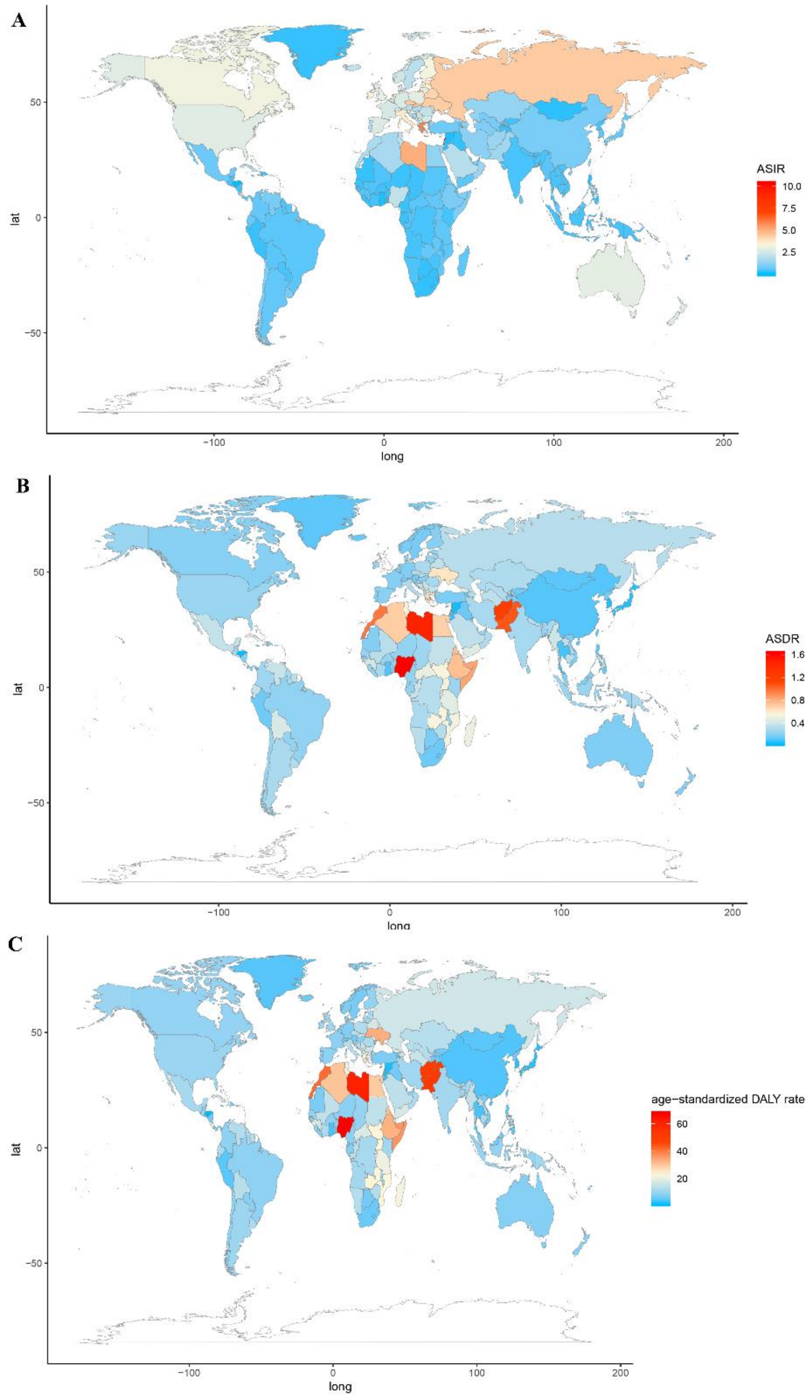

Sup Figure 10 The global disease burden of Hodgkin lymphoma for male in 194 countries. (A) The ASIR of Hodgkin lymphoma in 2017; (B) The ASDR of Hodgkin lymphoma in 2017; (C) The age standardized DALY rate of Hodgkin lymphoma in 2017. ASIR: age-standardized incidence rate; ASDR: age-standardized death rate.

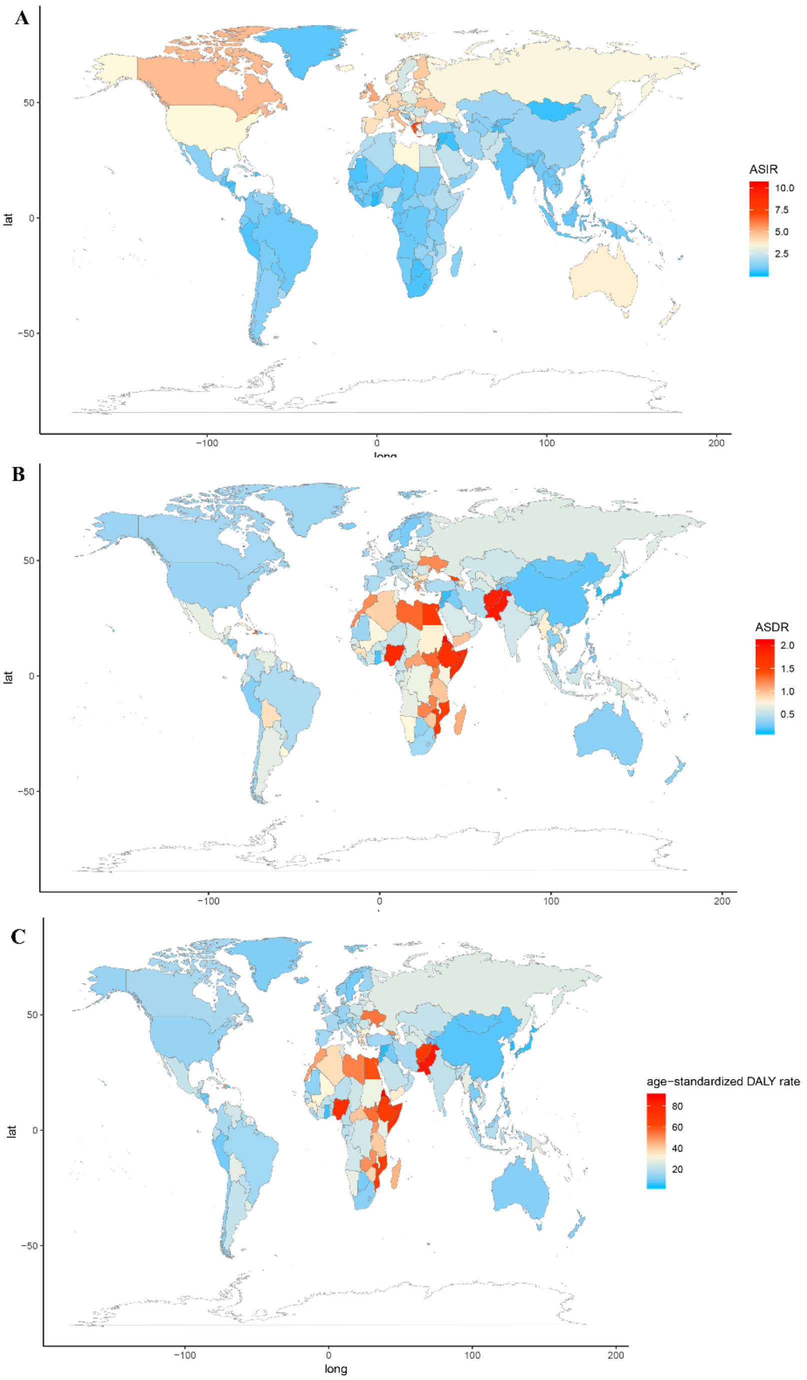

Sup Figure 11 Distribution of different ages in Hodgkin lymphoma death rate in global (A), high SDI (B), high-middle SDI (C), middle SDI (D), middle-low SDI (E), low SDI (F). SDI, socio-demographic index.

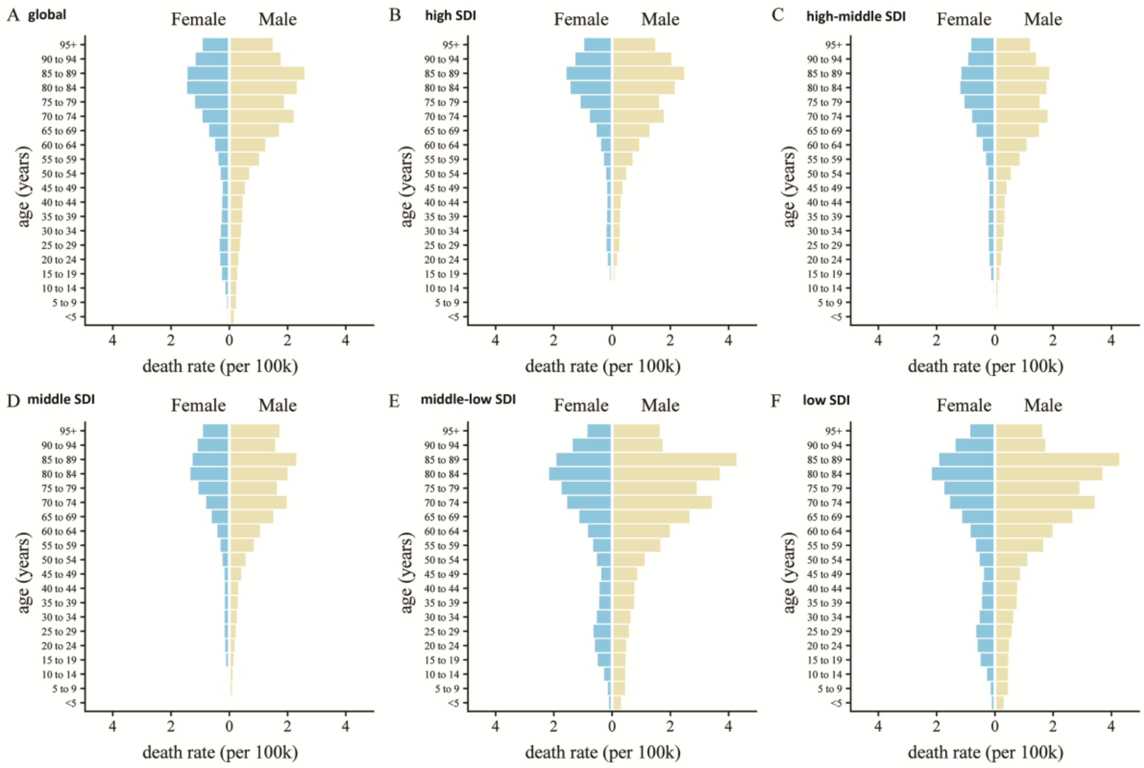

Sup Figure 12 The ratio of male to female ASDR among different age groups in global (A), high SDI (B), high-middle SDI (C), middle SDI (D), middle-low SDI (E), low SDI (F). SDI, socio-demographic index. ASDR, age-standardized death rate.

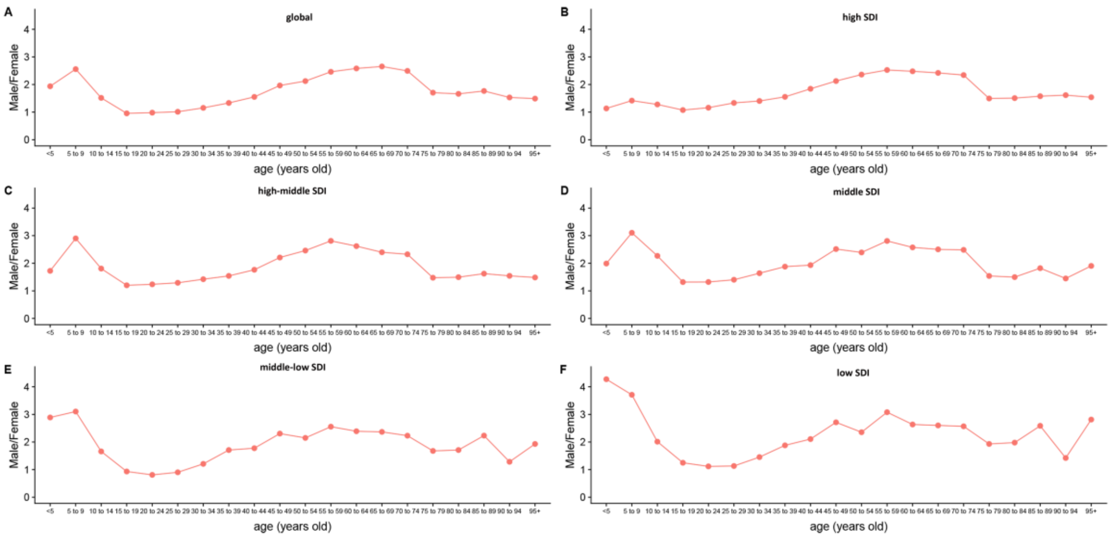

Sup Figure 13 Distribution of different ages in Hodgkin lymphoma DALYs rate in global (A), high SDI (B), high-middle SDI (C), middle SDI (D), middle-low SDI (E), low SDI (F). SDI, socio-demographic index.

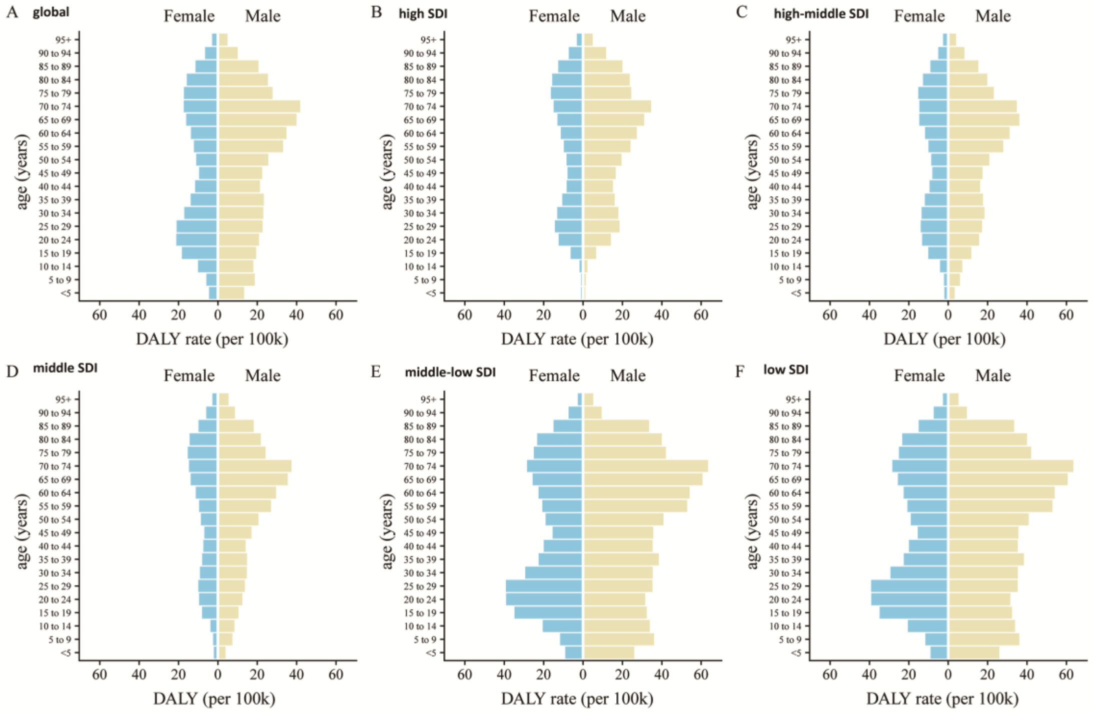

Sup Figure 14 The ratio of male to female age standardized DALY rate among different age groups in global (A), high SDI (B), high-middle SDI (C), middle SDI (D), middle-low SDI (E), low SDI (F). SDI, socio-demographic index.

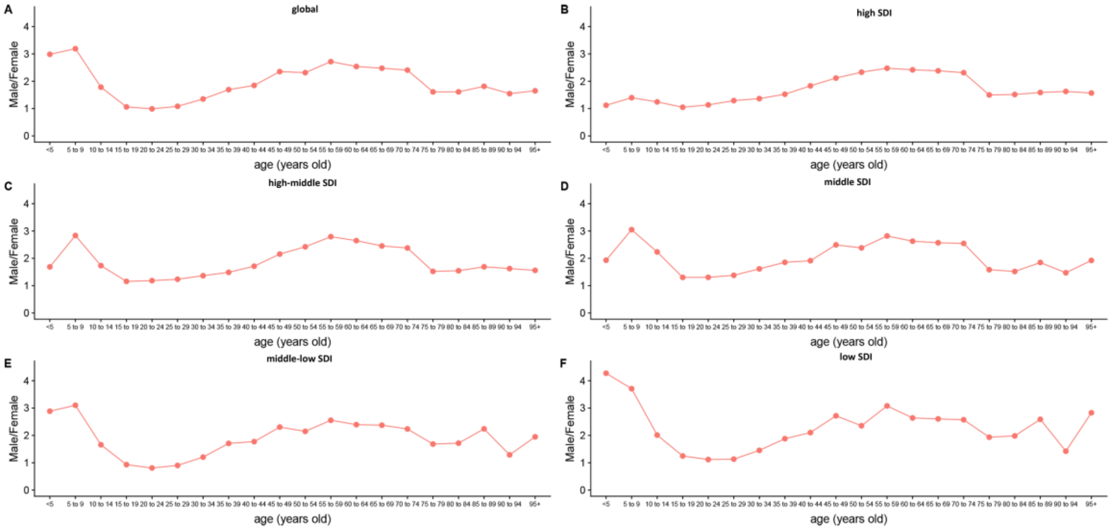

Supplement: Supplementary file 1 — Additional file 1. Supplementary tables and figures. [file 13045_2019_799_MOESM1_ESM.pdf]
